# ALOG domains: provenance of plant homeotic and developmental regulators from the DNA-binding domain of a novel class of DIRS1-type retroposons

**DOI:** 10.1186/1745-6150-7-39

**Published:** 2012-11-12

**Authors:** Lakshminarayan M Iyer, L Aravind

**Affiliations:** 1 National Center for Biotechnology Information, National Library of Medicine, National Institutes of Health, Bethesda, MD, 20894, USA

**Keywords:** DIRS1, Tyrosine recombinase, Plant development, DNA-binding, Retroposon, Transcription factor, Chromatin protein, Plant defense

## Abstract

Members of the *Arabidopsis* LSH1 and *Oryza* G1 (ALOG) family of proteins have been shown to function as key developmental regulators in land plants. However, their precise mode of action remains unclear. Using sensitive sequence and structure analysis, we show that the ALOG domains are a distinct version of the N-terminal DNA-binding domain shared by the XerC/D-like, protelomerase, topoisomerase-IA, and Flp tyrosine recombinases. ALOG domains are distinguished by the insertion of an additional zinc ribbon into this DNA-binding domain. In particular, we show that the ALOG domain is derived from the XerC/D-like recombinases of a novel class of DIRS-1-like retroposons. Copies of this element, which have been recently inactivated, are present in several marine metazoan lineages, whereas the stramenopile *Ectocarpus*, retains an active copy of the same. Thus, we predict that ALOG domains help establish organ identity and differentiation by binding specific DNA sequences and acting as transcription factors or recruiters of repressive chromatin. They are also found in certain plant defense proteins, where they are predicted to function as DNA sensors. The evolutionary history of the ALOG domain represents a unique instance of a domain, otherwise exclusively found in retroelements, being recruited as a specific transcription factor in the streptophyte lineage of plants. Hence, they add to the growing evidence for derivation of DNA-binding domains of eukaryotic specific TFs from mobile and selfish elements.

## Findings

Specific transcription factors (TFs) bind DNA sequences distinct from the promoter elements recognized by the basal TFs to activate or repress transcription [1]. In contrast to the basal TFs, which are highly conserved within each of the three superkingdoms of life, specific TFs show a great diversity in their structures and phyletic distributions [2]. The specific TFs of bacteria and archaea are dominated by DNA-binding domains (DBDs) displaying different versions of the helix-turn-helix (HTH) fold, several of which were already present in common ancestors of the two superkingdoms [3]. Although the basal transcriptional machinery of eukaryotes is similar in subunit composition and promoter-binding TFs to the archaeal counterpart [4], most of their specific TFs are unrelated to the prevalent prokaryotic families. They are also characterized by a greater variety in terms of the types of protein folds adopted by their DBDs, and great variability in their phyletic patterns and frequencies of occurrence in the organismal protein complements [2]. For example, specific TFs with the C2H2 finger and the homeodomain as their DBDs are the most frequently found ones in metazoans. In multicellular plants the MADS, VP1 (B3) and AP2 families are the most abundant specific TFs, whereas in fungi and the heterolobosean amoeboflagellate *Naegleria* the C6 finger is the dominant family [2]. The abundance and phylogenetic affinities of specific TF families can also vary between closely related lineages. For example, in amoebozoans, both dictyosteliids and *Entamoeba* have an abundance of specific TFs from the MYB family, which have arisen through independent lineage-specific expansions (LSEs), whereas the bZIP family is only expanded in the former subclade [2]. Indeed, LSEs appear to be the most striking evolutionary feature of eukaryotic transcription factors [2,5].

The lineage-specific diversity in eukaryotic specific TFs have posed interesting computational and evolutionary problems in terms of identifying these proteins from the genome sequences of non-model eukaryotes and elucidating the ultimate origins of the DBDs found in them. The principle of LSE as the main evolutionary trend in specific TFs, together with sensitive computational analyses, have aided in unearthing the principal specific TFs of diverse eukaryotes. These include the ApiAP2 family in apicomplexa, the C6 finger family in *Naegleria* , the MYB family in ciliates, *Entamoeba*, and *Trichomonas* and IBD family in *Trichomonas*[2,6,7]. In terms of the evolutionary origins of their DBDs, four principal sources can be identified: 1) DBDs that were acquired directly from prokaryotes, such as the MYB and the AP2 domains [8]. 2) DBDs, whose folds are found in prokaryotic TFs, but are not specifically related to any prokaryotic family. These could have evolved from prokaryotic precursors or eukaryotic paralogs via rapid sequence divergence. These include several families with HTH fold DBDs, such as the BRIGHT, homeodomain, HSF/ETS, TEA and FKH/Histone H1 domains [2,3]. 3) DBDs, such as the bHLH and bZIP, whose folds are uniquely found in eukaryotes, suggestive of their neomorphic innovation in eukaryotes [2]. 4) DBDs derived from transposases of mobile and selfish elements. In these cases the transposase activity is typically lost and a part or whole of the transposase domain is reused as a DBDs [2,9]. The last of these appears to have been a major contributor to the emergence of several eukaryotic specific TFs in different lineages. For example, in metazoans, the Paired, Pipsqueak, POU, THAP, and BED finger are derived from DBDs of various transposable elements [10-14]. Whereas in fungi, the Gcr1p family of TFs (e.g. Gcr1, Hot1, Ndc10, Msn1 and Sum1) is derived from the DBDs of transposases of crypton transposons [15], while in plants DBDs of the AP2 and B3/VP1 families are similarly derived from different mobile elements [16,17]. Across several eukaryotic lineages, the WRKY domain from transposases has been recruited as a DBD on multiple independent occasions [9]. Likewise, DBDs of the APSES family in fungi and the Dachshund family in metazoans can be traced to the KilA domain found in various DNA viruses (e.g. the nucleo-cytoplasmic DNA viruses) and a class of transposable elements related to DNA viruses [18,19].

The *Arabidopsis* LSH1 and *Oryza* G1 (ALOG) family of developmental regulators (corresponding to DUF640 in Pfam) were identified in the context of homeotic and developmental mutants in both eudicots and monocots [20-22]. In grasses, like rice, the morphology of the outer whorl of the typical angiosperm flower is drastically modified. As a result, there are two distinct structures, known as the lemma and the sterile lemma, which form outer bounding bract-like elements of a grass floret. In the *long sterile lemma1* or *g1* mutants of the cultivated rice a homeotic transformation of the sterile lemma into a regular lemma is observed [21]. The protein encoded by the *Oryza* G1 gene is homologous to the *Arabidopsis* LSH1 gene, which is involved in light-dependent regulation of hypocotyl length. Dominant mutants of the paralogous LSH3 and LSH4 genes suppress differentiation of leaves and disrupt the normal boundary regions between different floral organs [20,22]. The ALOG family of proteins encoded by these genes is present in multiple copies in land plants and was claimed to be absent outside of land plants [22]. Given the strong association between homeotic transformations and transcription regulators and chromatin proteins in both plants and animals, it has been suspected that the ALOG proteins might function as TFs [20,21]. This conjecture has been supported by their nuclear localization [21,22], but DNA-binding or relationship to any known TF has never been demonstrated for the ALOG family. In this study, using sensitive sequence and structure analysis, we provide evidence for the origin of the ALOG domain from the N-terminal DNA-binding domains of integrases belonging to the tyrosine recombinase superfamily encoded by a distinct type of DIRS1-like LTR retrotransposon found in several eukaryotes [23]. We also show that ALOG domains are additionally present in certain plant defense proteins.

### The ALOG domain belongs to the tyrosine recombinase/phage integrase N-terminal DBD superfamily

Members of the plant ALOG family are characterized by a single globular region flanked by short N- and C- terminal low-complexity segments [22]. We initiated iterative sequence profile searches using the PSI-BLAST and JACKHMMER programs with this globular region. For example, PSI-BLAST searches initiated with the globular region in *Arabidopsis* LSH1 (gi: 15241821, region 25–152) as a query recovered, in the first iteration, the previously reported ALOG family of proteins in multicellular plants, an AP-ATPase and TIR domain containing protein in *Arabidopsis* (gi: 240256009, E < 10^-9^) and proteins from the metazoans *Nematostella* and *Branchiostoma* (E between 10^-4^ and 10^-12^). Subsequent iterations of the search recovered similar proteins from other plants and converged in 3 iterations. Additionally, searches of the EST database with the TBLASTN program allowed us to recover an ALOG domain protein from *Spirogyra* which is a representative of the algal clade of Zygnematophyceae. These translating searches also led to the detection of versions of the ALOG domain in several other metazoans, such as the molluscs *Lottia* and *Crassostrea*, the starfish *Asterina pectinifera (Patiria pectinifera)*, the coral *Acropora digitifera* and the stramenopile brown alga *Ectocarpus siliculosus*. Iterative profile searches with the JACKHMMER program, further consistently recovered the N-terminal regions of XerC/D-like tyrosine recombinases at E-values below the significant threshold (e.g. *B. cereus* XerD, gi: 229025548, hsp region: 27–67, e-value 0.3-0.5).

**Figure 1:**
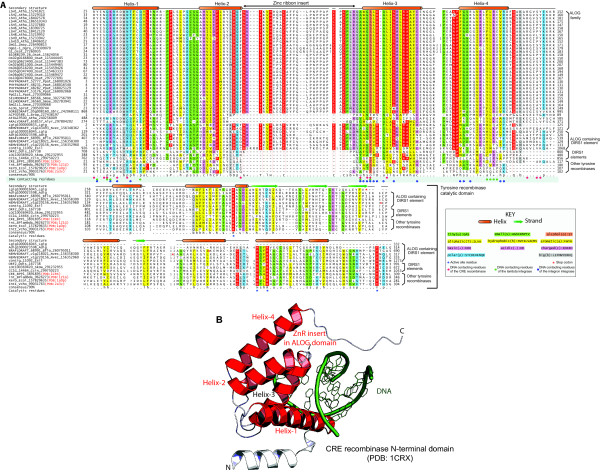
**(A) Multiple sequence alignment of the DNA-binding ALOG and catalytic tyrosine recombinase domains.** Proteins are labeled by their gene names, species abbreviations and Genbank index numbers separated by underscores. Sequences are colored based on their conservation at 90% consensus. The coloring scheme, consensus abbreviations and secondary structure representation are shown in the key. Absolutely conserved residues are shaded red. For residues encompassing the tyrosine recombinase N-terminal/ALOG domain, the consensus was computed based on the conservation of the alignment positions in ALOG domain-containing proteins. Also highlighted are the DNA-contacting residues derived from crystal structures of tyrosine recombinase DBDs, and the catalytic residues of the tyrosine recombinase catalytic domain. Species abbreviations are as follows. Adig : *Acropora digitifera*; Alyr : *Arabidopsis lyrata*; Atha : *Arabidopsis thaliana*; BPP1 : Enterobacteria phage P1; BPlambda : Enterobacteria phage lambda; Bflo : *Branchiostoma floridae*; Brap : *Brassica rapa*; Ccin : *Coprinopsis cinerea*; Ddis : *Dictyostelium discoideum*; Ecol : *Escherichia coli*; Esil : *Ectocarpus siliculosus*; Lgig : *Lottia gigantea*; Nvec : *Nematostella vectensis*; Ogra : *Oryza grandiglumis*; Osat : *Oryza sativa*; Ppat : *Physcomitrella patens*; Sbic : *Sorghum bicolor*; Skow : *Saccoglossus kowalevskii*; Smoe : *Selaginella moellendorffii*; Spra : *Spirogyra pratensis*; Vcho : *Vibrio cholerae*; Zmay : *Zea mays*. (**B**) Cartoon representation of the N-terminal DBD of the CRE recombinase (PDB: 1CRX) in complex with DNA illustrating the position of the predicted ALOG domain zinc ribbon. Helices in the DBD that are conserved in the ALOG domain are colored red.

In order to evaluate this relationship and to study the conservation patterns of the ALOG domain, we constructed a multiple alignment of these proteins using the Kalign2 program. Secondary structure predictions revealed an all-α helical domain with four conserved helices (Figure 1). Residues conserved across the alignment include a pair of aromatic and aliphatic residues in helices 1 and 2 with a “[FY][LMV]” signature, a conserved predicted Zinc-Ribbon (ZnR) insert between helices 2 and 3 with “HxxxC” and “CxC” motifs, a highly conserved basic residue towards the C-terminal end of the ZnR insert, a conserved aspartate and an +xR motif (where + is H, K, R) in helix-3, and two basic residues and a conserved Q in helix-4. Of these, the conserved basic residue (typically arginine) in helix-4 was observed as being mutated in the naturally occurring homeotic mutant of *Oryza*. We then ran a profile-profile comparison using the HHPRED program with a HMM derived from the ALOG domain alignment against a panel of HMMs derived using PDB structures as search seeds. This search recovered the N-terminal DBD of several members of the tyrosine recombinase clade prototyped by the XerC/D recombinases with significant scores (e.g. the *Haloarcula* XerC/D-like recombinase [PDB: 3nrw]: probability 95% and p=10^-6^; CRE recombinase [PDB 1x0O]: probability 93% and p=10^-5^; *Escherichia coli* XerD recombinase [PDB: 1a0p]: probability 82% and p=10^-4^). The profile-profile alignments completely covered the conserved core of four helices in the N-terminal DBD of the XerC/D-like clade of tyrosine recombinases by precisely skipping the central Zn-ribbon (ZnR) insert in the ALOG domain. A comparison of the conservation profiles of the tyrosine recombinase N-terminal DBD and the ALOG domain revealed a shared pattern of hydrophobic residues in all the four helices, which are critical for the stabilization of the core tetrahelical fold (Figure 1). These observations supported the ALOG domain being a version of the N-terminal DBD of the XerC/D clade tyrosine recombinases, with a ZnR inserted into the core tetrahelical structure.

### Radiation of ALOG domains occurred in the streptophyte clade of the plant lineage

**Figure 2:**
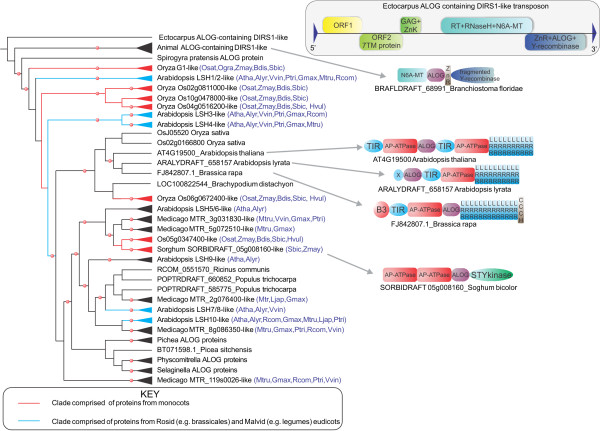
**(B). Phylogenetic tree of the ALOG domain, domain architectures, and structure of the ALOG containing DIRS-1 transposon.** The tree was reconstructed using an approximately maximum-likelihood method implemented in the FastTree 2.1 program (see Material and methods). Clades with boostrap values equal to or above 80% are marked with a red circle. Well-supported clades are collapsed and shown as triangles, which are colored based on their phyletic patterns (shown in the key below). The higher-order relationships should be viewed with caution due to the shortness of the alignment. Phyletic patterns of the collapsed clades are shown next to the clade name in brackets. Species abbreviations are as in Figure 1. For complete details, refer to the supplement. Also shown is the structure of the complete transposon extracted from the *Ectocarpus* genome. Domains in the architectures are not drawn to scale. X refers to an uncharacterized domain.

Most ALOG domains in multicellular plants exist as solo domains flanked by low-complexity extensions, and correspond to the originally described ALOG family of proteins. In the eudicots, *Arabidopsis and Brassica*, and in the monocot *Sorghum*, the ALOG domains are additionally fused to domains found in plant counter-pathogen defense proteins, such as the TIR, AP-ATPase, and LRR repeats, and in certain cases to a MAP-kinase-like module (Figure 2). In these proteins the ALOG domain is present either at the N-terminus (e.g. gi: 297804202; *Arabidopsis lyrata*) or in the middle of the protein (e.g. AT4G19500 of *Arabidopsis thaliana* and SORBIDRAFT_05g008160 of *Sorghum bicolor*) (Figure 2). The ALOG domains in these *Arabidopsis* and *Brassica* proteins lack the ZnR insert, whereas the *Sorghum* version retains it, similar to the solo ALOG proteins (Figure 1). In the green plant lineage, outside of the multicellular land plants, the only other organism with an ALOG domain was the alga *Spirogyra* belonging to the clade Zygnemophyceae. We did not observe any representatives of this domain in chlorophyte alga. This suggests that the ALOG domain was probably acquired at some point in course of the diversification of the streptophyte clade of plants that unites *Spirogyra* and the land plants. A phylogenetic tree of the ALOG domain (Figure 2) revealed that its evolutionary history is dominated by lineage-specific duplications. Within plants, these expansions appear to have occurred after the separation of the monocot and dicot lineages. In many instances, duplications appeared to have occurred very late, i.e. within particular terminal clades, such as within Brassicaceae or legumes. Within dicots, only 5 lineages namely, LSH1/2, LSH3, LSH4, LSH7/8 and LSH10 can be confidently recognized as being present in the common ancestor of the legumes and Brassicales, corresponding to the rosid and malvid clades of eudicots. Further, both sequence analysis and phylogenetic trees support the independent accretion of monocot and dicot ALOG domains to defense proteins with AP-ATPase domains.

### All other ALOG domains map to DIRS1-like retroposons or their remnants

Outside of the streptophyte clade, ALOG domains are sporadically present in several distant metazoan lineages and the brown alga *Ectocarpus*. In metazoans they are found in certain cnidarians, molluscs, echinoderms and the cephalochordate *Branchiostoma floridae.* However, in most metazoan groups for which genome data exists, these domains are observed as being absent in the sister lineages of those that possess them. Thus, ALOG domains are present in *Nematostella* and the coral *Acropora*, but are absent in *Hydra*. Likewise, while they are present in the starfish *Asterina pectinifera,* they appear to be absent in the sea urchin *Strongylocentrotus*. This phyletic pattern pointed to the possibility of active mobility of this domain across phylogenetically distant organisms living in the marine environments. To better understand this mobility, we investigated the domain architectures of these versions and found that they tended to be fused to a distinct, catalytically inactive N6-adenine methylase domain at the N-terminus, and a tyrosine recombinase catalytic domain at the C-terminus that is fragmented in the metazoan versions (Figure 2). These two domains have been previously noted as distinguishing features of the DIRS1 class of eukaryotic retroposons [24,25]. This observation, together with the relationship of the ALOG domain to the N-terminal DBD of the XerC/D-clade of tyrosine recombinases, suggested that these versions might be derived from DIRS1-like retroposons. To test this, we investigated genomic sequence flanking the ALOG-containing ORF to identify other features of these retroposons. Indeed, in all the above organisms our nucleotide searches detected regions homologous to the reverse transcriptase-RNaseH gene upstream of the ALOG-containing ORF. However, in most cases, barring the *Ectocarpus* genome, these appeared to be disrupted by multiple stop codons or fragmentation (Figure 1 and Additional file 1). In the *Ectocarpus* genome we were able identify two complete copies of the potential retroposon and one of them appeared to be active. This helped us define the structure of the intact version of these elements, which in *Ectocarpus* are little over 9Kb in length with terminal direct repeats of 159 bp (Additional File 1). It encodes a Gag and Zn-knuckle protein, a reverse transcriptase+RNAseH, an inactive adenine methylase, and a tyrosine recombinase protein with a N-terminal FCS-type ZnR, followed by the ALOG domain and finally the recombinase catalytic domain (Figure 2). It shares all these with other retroposons of the DIRS1 class [25], but additionally encodes multiple overlapping fast-diverging ORFs in its 5’ end including one for a predicted 7-transmembrane protein (Figure 2 and Additional file 1). The tyrosine recombinase with the ALOG domain distinguishes this element from all other previously described DIRS1 retroposons [25].

Searches with the complete active element from *Ectocarpus* revealed that the metazoan elements share a similar organization, although it is not clear if they share the fast-diverging 5’ORFs with the former (Additional file 1). Furthermore, these searches revealed that in each of the above-mentioned organisms, where the ALOG-containing DIRS1 elements could be detected, they had undergone proliferation to spawn numerous copies (Additional f.ile 1). However, except for a single copy in *Ectocarpus,* all other copies, both in this organism and all the metazoans, are inferred to be inactive on account of multiple stop codons disrupting one or more of the key functional ORFs. This suggests that there is a strong selective pressure, especially in metazoans, for the inactivation of these retroposons, probably due to the risks posed to genomic integrity by their tyrosine recombinases. However, in *Nematostella*, at least six of these retroposon remnants contain an ORF that specifies a complete ALOG domain (Figure 1, Additional file 1). An interesting possibility is that these solo ALOG domains might function as possible DNA-binding regulators in this organism, just as their plant counterparts. Thus, the above observations establish that the ALOG domain is indeed derived from the DBD of the tyrosine recombinase of a novel retroposon of the DIRS1 class. Complete versions of such elements are currently not known from the streptophyte clade of plants. This suggests that they were probably invaded early in their evolution by such an element followed by their inactivation and retention of the ALOG portion alone as a regulatory protein.

#### The predicted DNA-binding mode of the ALOG domain and its functional implications

Unification of the ALOG domain with the phage tyrosine recombinase N-terminal domain and the availability of multiple crystal structures of these enzymes in complexes with DNA [26-30] allowed us to reconstruct its DNA-binding properties. An examination of these structures revealed that all tyrosine recombinases share a common mode of interacting with DNA via a combination of contacts from the N-terminal region and the C-terminal catalytic domain. We were able to identify a conserved domain in the N-terminal regions of all tyrosine recombinases, which is comprised of three core α-helices that usually make a contact with the major groove of DNA (Figure 1B and Additional file 1). The catalytic domain binds diametrically opposite to this site with its active site dyad of arginines, histidine and tyrosine positioned in the minor groove (Additional file 1). Usually, further contacts are also made by the linker that connects the N-terminal DNA-binding regions to the C-terminal catalytic domain. In the simplest case, namely the XerC/D-like clade (includes in addition to the eponymous recombinases, the phage integrases, like those of Lambda and P1, the integron integrases and integrases of the classical DIRS1 elements) the three major groove-contacting helices of the N-terminal DBD are incorporated into a helical bundle with at least one additional conserved helix (Figure 1B and Additional file 1). The ALOG domain preserves this situation with three core DNA-contacting helices forming a bundle with a fourth C-terminal helix (Figure 1B). Thus, the ALOG domain is predicted to bind the major groove of the DNA by deeply inserting into it (Figure 1B). The ALOG domain differs from the simple DBDs of the XerC/D-like clade in possessing the Zn ribbon between the 2^nd^ and 3^rd^ helices that insert into the major groove. Based on the available structures, we predict that this Zn ribbon is suitably positioned to make additional DNA contacts that could extend to the adjacent minor groove (Figure 1). This situation, featuring additional contacts, is reminiscent of the embellishments frequently observed among the DBDs of tyrosine recombinases. These might occur in the form of fusion to additional N-terminal DBDs, such as the AP2 domain, which contacts distantly located DNA segments in the lambda integrase [6,27]. Alternatively, in the case of the topoisomerase IA, protelomerase and the Flp recombinase clades of tyrosine recombinases additional DBDs, respectively an all β-strand, an all α-helical and an α+β domain, are inserted between the N-terminal helical DBD and the C-terminal catalytic domain (Additional file 1). Moreover, the protelomerase clade shows an additional embellishment in the form of a winged HTH DBD C-terminal to the catalytic domain.

Based on the structure of the XerC/D-like clade, we infer that the helix-1 and helix-3 of the ALOG domains, which are orthogonally positioned with respect to each other (Figure 1B), are likely to make key backbone and base contacts in the major groove. Conserved positively charged residues from the Zn-ribbon are likely to provide additional contacts unique to the ALOG domains (Figure 1). Additionally, in the plant proteins there are several well-conserved positively charged residues in the region after helix-4, which based on the precedence of the tyrosine recombinase structures, are also likely to form accessory DNA-contacting sites (Figure 1). With the exception of an alcoholic-group residue in helix-3, which is conserved across much of the XerC/D-like clade (Figure 1), most of the other DNA-contacting residues show differences between the ALOG domain and other members of this clade, suggesting differences in target sequence specificity. Most plant ALOG domains are very similar in the inferred DNA-contacting positions suggesting that they are likely to bind similar target sequences (Figure 1). Thus, the above prediction of sequence-specific DNA-binding by ALOG domain is consonant with the standalone versions functioning as specific TFs in plants. However, based on the observed phenotypes, we also envisage a slightly distinct possibility. Both in *Arabidopsis* and *Oryza* the standalone ALOG proteins facilitate a phenotype consistent with large-scale gene repression, such as the suppression of default organ identity in the sterile lemma of rice by G1 [21], and the suppression of organ differentiation in boundary regions by LSH3 and LSH4 [20]. This raises the possibility that DNA-binding by the ALOG proteins might help nucleate repressive chromatin that facilitates these shifts in organ identity. The fusions to potential defense proteins with AP-ATPase domains suggest that in these contexts, the ALOG domain might also function as a sensor for invading DNA. The domain could potentially recognize replication and recombination intermediates of plant viruses such as ss-, ds DNA-, or pararetro- viruses or even invasive DIRS1-like elements and initiate a defense response via the AP-ATPase domains.

## General Conclusions

Thus, the ALOG domain joins the ranks of several other DNA-binding transcription regulators of eukaryotes that were derived from DBDs of mobile elements. To our knowledge, this is the first instance of a domain otherwise exclusively found in retroelements being recruited for such a function. We hope the findings presented here will help in guiding further laboratory studies on its DNA-binding specificity and mechanism of action.

## Methods

Iterative sequence profile searches were performed using the PSI-BLAST [31] and web version of the JACKHMMER (http://hmmer.janelia.org/search/jackhmmer) [32] programs, run against the non-redundant (NR) protein database of National Center for Biotechnology Information (NCBI). Multiple sequence alignments were built by the Kalign2 [33] and MUSCLE [34] programs, followed by manual adjustments on the basis of profile-profile and structural alignments. Similarity-based clustering for both classification and culling of nearly identical sequences was performed using the BLASTCLUST program (ftp://ftp.ncbi.nih.gov/blast/documents/blastclust.html). The HHpred program [35] was used for profile-profile comparisons. Structure similarity searches were performed using the DaliLite program [36]. Secondary structures were predicted using the JPred [37] program. For previously known domains the Pfam database [38] was used as a guide, though the profiles were augmented by addition of newly detected divergent members that were not detected by the original Pfam models. Phylogenetic analysis was conducted using an approximately-maximum-likelihood method implemented in the FastTree 2.1 program under default parameters [39]. Structural visualization and manipulations were performed using the PyMol (http://www.pymol.org) programs. The in-house TASS package, which comprises a collection of Perl scripts, was used to automate aspects of large-scale analysis of sequences, structures and genome context.

## Competing interests

The authors declare that they have no competing interests.

## Authors’ contributions

LMI and LA made the discovery and prepared the paper. Both authors read and approved the final manuscript.

## Reviewers' comments

Reviewer 1: Prof. Valerian Dolja (Oregon State University, USA)

This work by Iyer and Aravind presents a remarkable case of the Virus World incursion into evolution of the cellular organisms. Using sensitive bioinformatics approaches, the authors trace origins of the ALOG domain-containing regulators of plant development to a distinct lineage of the DIRS-1-like retrotransposons. Furthermore, investigation of the predicted folding pattern of the ALOG domains suggested that these domains function as the DNA-binding transcription factors thus enabling experimental inquiry into action mechanism of these enigmatic plant proteins. I am particularly impressed with the breadth and depth of this work spanning the areas of molecular and evolutionary biology of the selfish genetic elements and plants, exactly the areas of my own research interest. Is it not ironic that the retroelement-derived domain was recruited by flowering plants to not only modulate their development, but also to fortify their innate immune response via including DNA-sensing ALOG domain into counterdefensive TIR and LRR repeat proteins? I am certain that the students of both plant development and plant-pathogen interactions will be enthusiastic about testing the predictions of this study.

The manuscript is very well written and documented and is a pleasure to read. There are only a few stylistic inaccuracies that need to be corrected (one example is fourth sentence of the Abstract starting with Recently).

Authors’ response:

We thank the reviewer for his positive comments. We have revised the manuscript for stylistic, grammatical and typographic errors.

Reviewer 2: Dr. Gaspar Jekely (Max Planck Institute for developmental biology, Germany)

The paper by Iyer and Aravind convincingly demonstrates that the ALOG domain, first described as being part of certain plant developmental proteins, derives from a DNA-binding domain found in DIRS1-like mobile elements. This discovery suggests that the ALOG domain may also have a DNA-binding function in plants. The ALOG domain thus represents an addition to the growing list of DBDs derived from mobile elements. The careful sequence and structural analyses presented could guide future experiments in plant models. The authors also identify a putatively active, novel type of DIRS1-like retrotransposon from the brown alga *Ectocarpus*. The ALOG domain was also identified in several marine invertebrates, and the domain composition of these ALOG domain-containing proteins suggests that they also derive from mobile elements.

The widespread but patchy occurrence of the ALOG domain together with domains characteristic of mobile elements points to the active mobility of these elements in marine environments. However, given that all the elements seem to be degraded in animals, the alternative possibility is that the patchy distribution is due to a single early origin and linear descent combined with occasional losses (e.g. in Hydra). It would be interesting to see whether or not the animal part of the tree corresponds to the known phylogeny. A correspondence would support linear descent, the lack of it would rather suggest independent multiple origins. (The ALOG domain from the oyster *Crassostrea gigas* (EKC24824) that has recently been added to the database could also be added to the sequences.) If independent origins can be confirmed, this would suggest that with further sampling in marine organisms, an active version of the element may be found (as in *Ectocarpus*) or at least could be reconstructed from several recently inactivated ones. This could have potential practical uses in emerging marine models like *Nematostella*.

Authors’ response:

*We thank the reviewer for pointing out to us about the availability of the Crassostrea genome, data from which we have now included in this study. We recovered three, almost identical, copies of the element in its genome, but they all possess several stop codons and frame shifts in the coding sequences of the different ORFs* (see Additional file 1), *suggesting that, like the versions in other metazoans, they have been recently inactivated. We provide, in Additional file 1, several examples of reconstructed ALOG-containing DIRS1 elements from different metazoans andEctocarpus. Although the metazoan sequences group together in phylogenetic trees, their branching patterns fail to recapitulate even the reliable species relationships. Thus, while gene loss in sister species could have played a role, the available evidence supports the important role of lateral transfer of this element between various marine species.*

## Supplementary Material

Additional file 1 ALOG domains: provenance of plant homeotic and developmental regulators from the DNA-binding domains of a novel DIRS1-class retroposon. Click here for file
