# Supplementary material for: ALOG domains: provenance of plant homeotic and developmental regulators from the DNA-binding domain of a novel class of DIRS1-type retroposons
Source: Biol Direct. 2012 Nov 12;7:39. doi: 10.1186/1745-6150-7-39 (PMC3537659; doi:10.1186/1745-6150-7-39)
Supplement: Additional file 1 — ALOG domains: provenance of plant homeotic and developmental regulators from the DNA-binding domains of a novel DIRS1-class retroposon. [file 1745-6150-7-39-S1.html]

Supplementary material for the manuscript "ALOG domains: provenance of plant homeotic and developmental regulators from the DNA-binding domains of a novel DIRS1-class retroposon"


  
**Supplementary Material  
ALOG domains: provenance of plant homeotic and developmental regulators from the DNA-binding domains of a novel DIRS1-class retroposon**  
  
Lakshminarayan M. Iyer and L. Aravind\*,   
\* *Address for correspondence: L. Aravind (aravind@mail.nih.gov)*  
  
 *National Center for Biotechnology Information, National Library of Medicine, National Institutes of Health, Bethesda, MD 20894, USA*  
  

---

|  |
| --- |
|  |
| **Abstract** Paste abstract here |

---

|  |  |
| --- | --- |
|  | **Contents**- Comprehensive multiple sequence of the ALOG domain- Phyletic patterns and domain architectures of the ALOG domain- Approximately maximum-likelihood tree of the ALOG domains - **Structure of the ALOG domain-containing DIRS1-like transposons in eukaryotes**         - Structure of the ALOG domain-containing DIRS1-like transposons in Ectocarpus siliculosus         - Structure of the ALOG domain-containing DIRS1-like transposons in Branchiostoma floridae and coordinates of other fragmentary elements         - Structure of the ALOG domain-containing DIRS1-like transposons in Nematostella vectensis and coordinates of other fragmentary elements         - Structure of the ALOG domain-containing DIRS1-like transposons in Crassostrea gigas- **Structures of various tyrosine recombinases depicting the common structural core in their N-terminal DNA-binding domain**                ---            - **High resolution versions of the figures**              Figure 1. Multiple sequence alignment and structure of the N-terminal DBD of the CRE recombinase               Figure 2. Phylogenetic tree of the ALOG domain, domain architectures, and structure of the ALOG containing DIRS-1 transposon.              ---              - **Fasta sequences of sequences that were incomplete in genbank, cDNA sequences, and sequences from unfinished genomes assigned a temporary id can be accessed here.**                   ---                **- Comprehensive multiple sequence of the ALOG domain**                 ```               Domain_boundaries                    <--------------ALOG domain----------------------------------------------------------------------------------------------------------------------------------------------->-------<-----Tyrosine recombinase catalytic domain-------------------------------------------------------------------------------------------------------------------------------------------------------------------------------------------------------------->               Secondary_Structure                  HHHHHHHHHHHHHHHHHHHH------------HHHHHHHHHHHHH----------------------------------------------HHHHHHHHHHHHHHHHHH--------------------------------HHHHHHHHHHHHHHH--------------------------HHHHHHHHH---------------------HHHHHHHHHHH----HHHH-EEEEEEEE---------EEEEEE----------EEEEEE---------------HHHHHHHHHHH----------------------------------EEE---------------------HHHHHHHHHHHHH------------------HHHHHHHHHHHHHH--HHHHHH-------------------               LSH1_Atha_15241821                   RYENQKRRDWNTFCQYLRNHR-PPLSLPSCSGAHVLEFLRYLDQFGK---TKVHHQNCAFFG--LPNPPAP-------C-----PCPLRQAWGSLD-ALIGRLRAAYEENGGP-----------------------PEANPFGSRAVRLFLREVRDFQAKARGVSYEKKR-----------------------------------------------------------------------------------------------------------------------------------------------------------------------------------------------------------------------------------------------------------------               LSH2_Atha_15229310                   RYENQKRRDWNTFCQYLRNHH-PPLSLASCSGAHVLDFLRYLDQFGK---TKVHHQNCAFFG--LPNPPAP-------C-----PCPLRQAWGSLD-ALIGRLRAAYEENGGA-----------------------PETSPFGSRSVRIFLREVRDFQAKSRGVSYEKKR-----------------------------------------------------------------------------------------------------------------------------------------------------------------------------------------------------------------------------------------------------------------               LSH3_Atha_18402578                   RYENQKRRDWNTFGQYLRNHR-PPLSLSRCSGAHVLEFLRYLDQFGK---TKVHTNICHFYG--HPNPPAP-------C-----PCPLRQAWGSLD-ALIGRLRAAFEENGGK-----------------------PETNPFGARAVRLYLREVRDMQSKARGVSYEKKK-----------------------------------------------------------------------------------------------------------------------------------------------------------------------------------------------------------------------------------------------------------------               LSH4_Atha_186510343                  RYENQKRRDWNTFGQYLRNHR-PPLSLSRCSGAHVLEFLRYLDQFGK---TKVHTHLCPFFG--HPNPPAP-------C-----ACPLRQAWGSLD-ALIGRLRAAFEENGGS-----------------------PETNPFGARAVRLYLREVRDSQAKARGISYEKKK-----------------------------------------------------------------------------------------------------------------------------------------------------------------------------------------------------------------------------------------------------------------               LSH5_Atha_15237680                   RYESQKRRDWNTFLQYLRNHK-PPLNLSRCSGAHVLEFLKYLDQFGK---TKVHATACPFFG--QPNPPSQ-------C-----TCPLKQAWGSLD-ALIGRLRAAFEEIGGG----------------------lPESNPFAAKAVRIYLKEVRQTQAKARGIPYDKKK-----------------------------------------------------------------------------------------------------------------------------------------------------------------------------------------------------------------------------------------------------------------               LSH6_Atha_18390725                   RYESQKRRDWNTFLQYLKNHK-PPLALSRCSGAHVIEFLKYLDQFGK---TKVHVAACPYFG--HQQPPSP-------C-----SCPLKQAWGSLD-ALIGRLRAAYEENGGR-----------------------PDSNPFAARAVRIYLREVRESQAKARGIPYEKKK-----------------------------------------------------------------------------------------------------------------------------------------------------------------------------------------------------------------------------------------------------------------               LSH7_Atha_18412120                   RYESQKRRDWNTFCQYLRNQQ-PPVHISQCGSNHILDFLQYLDQFGK---TKVHIHGCVFFG--QVEPAGQ-------C-----NCPLKQAWGSLD-ALIGRLRAAFEENGGL-----------------------PERNPFAGGGIRVFLREVRDSQAKARGVPYKKRK-----------------------------------------------------------------------------------------------------------------------------------------------------------------------------------------------------------------------------------------------------------------               LSH8_Atha_15219952                   RYESQKSRDWNTFCQYLMTKM-PPVHVWECESNHILDFLQSRDQFGK---TKVHIQGCVFFG--QKEPPGE-------C-----NCPLKQAWGSLD-ALIGRLRAAYEENGGL-----------------------TEKNPFARGGIRIFLREVRGSQAKARGVLYKKKK-----------------------------------------------------------------------------------------------------------------------------------------------------------------------------------------------------------------------------------------------------------------               LSH9_Atha_15233942                   RYESQKRRDWNTFVQYLKSQN-PPLMMSQFDYTHVLSFLRYLDQFGK---TKVHHQACVFFG--QPDPPGP-------C-----TCPLKQAWGSLD-ALIGRLRAAYEEHGGGs----------------------PDTNPFANGSIRVHLREVRESQAKARGIPYRKKK-----------------------------------------------------------------------------------------------------------------------------------------------------------------------------------------------------------------------------------------------------------------               LSH10_Atha_18406012                  RYESQKRRDWNTFGQYLKNQR-PPVPMSHCSCNHVLDFLRYLDQFGK---TKVHVPGCMFYG--QPEPPAP-------C-----TCPLRQAWGSLD-ALIGRLRAAYEENGGP-----------------------PETNPFASGAIRVYLREVRECQAKARGIPYKKKK-----------------------------------------------------------------------------------------------------------------------------------------------------------------------------------------------------------------------------------------------------------------               OgG1-1_Ogra_270309070                RYESQKRRDWQTFTQYLAAHR-PPLELRRCSGAHVLEFLRYLDRFGK---TRVHESPCPAYGGRALSAAGPvvaa---C-----QCPLRQAWGSLD-ALVGRLRAAYDERQGRagepda-aaagavatdstssssaAAANPFAARAVGLYLRDVRDAQAMARGISYHKKK-----------------------------------------------------------------------------------------------------------------------------------------------------------------------------------------------------------------------------------------------------------------               G1_Osat_27260935                     RYESQKRRDWQTFTQYLAAHR-PPLELRRCSGAHVLEFLRYLDRFGK---TRVHEPPCPSYGGRSPSAAGPvaaaaaaC-----QCPLRQAWGSLD-ALVGRLRAAYDERHGRagepdavagagavatdstssssaAAANPFAARAVRLYLRDVRDAQAMARGISYHKKK-----------------------------------------------------------------------------------------------------------------------------------------------------------------------------------------------------------------------------------------------------------------               ZmG1_Zmay_226490815                  RYESQKRRDWQTFTRYLGAHR-PPLELCRCSGAHVLEFLRYLDRFGK---TRVHAPSCAAYG--GGGGGRPveaava-C-----QCPLRQAWGSLD-ALVGRLRAAFEERHGArg--iwtssqqpdggvgvgvgggDGANPFAARAVRLYLRDVRDAQSRARGISYSRKK-----------------------------------------------------------------------------------------------------------------------------------------------------------------------------------------------------------------------------------------------------------------               B1088C09.26_Osat_15624056            RYESQKRRDWNTFLQYLRNHR-PPLTLARCSGAHVIEFLRYLDQFGK---TKVHASGCAFYG--QPSPPGP-------C-----PCPLRQAWGSLD-ALIGRLRAAYEESGGT-----------------------PESNPFAARAVRIYLREVRDSQAKARGIPYEKKK-----------------------------------------------------------------------------------------------------------------------------------------------------------------------------------------------------------------------------------------------------------------               Os02g0166800_Osat_115444445          RYESQKRRDWQTFGQYLRNHR-PPLELSRCSGAHVLEFLRYLDQFGK---TKVHAHGCPFFG--HPSPPAP-------C-----PCPLRQAWGSLD-ALVGRLRAAFEEHGGR-----------------------PESNPFGARAVRLYLRDIRDTQSKARGIAYEKKR-----------------------------------------------------------------------------------------------------------------------------------------------------------------------------------------------------------------------------------------------------------------               Os02g0623400_Osat_115447383          RYEAQKRRDWNTFGQYLRNHR-PPLGLAQCSGAHVLEFLRYLDQFGK---TKVHTAACPFFG--HPNPPAP-------C-----PCPLRQAWGSLD-ALVGRLRAAFEENGGR-----------------------PESNPFAVRAVRLYLREVREHQARARGVSYEKKK-----------------------------------------------------------------------------------------------------------------------------------------------------------------------------------------------------------------------------------------------------------------               Os02g0811000_Osat_115449465          RYESQKRRDWNTFGQYLRNHR-PPLSLSRCSGAHVLEFLKYMDQFGK---TKVHTPVCPFYG--HPNPPAP-------C-----PCPLRQAWGSLD-ALIGRLRAAYEENGGT-----------------------PEMNPFGARAVRLYLREVRETQARARGISYEKKK-----------------------------------------------------------------------------------------------------------------------------------------------------------------------------------------------------------------------------------------------------------------               Os04g0516200_Osat_115459426          RYEAQKRRDWNTFGQYLRNHR-PPLSLAQCSGAHVLEFLRYLDQFGK---TKVHTAACPFFG--HPSPPAP-------C-----PCPLRQAWGSLD-ALVGRLRAAFEENGGR-----------------------PESNPFAARAVRLYLREVREHQARARGVSYEKKK-----------------------------------------------------------------------------------------------------------------------------------------------------------------------------------------------------------------------------------------------------------------               Os05g0347400_Osat_115463333          RYESQKRRDWNTFLQYLRNHK-PPLTLPRCSGAHVIEFLKYLDQFGK---TKVHADGCAYFG--EPNPPAP-------C-----ACPLRQAWGSLD-ALIGRLRAAYEESGGR-----------------------PESNPFAARAVRIYLREVREAQAKARGIPYEKKR-----------------------------------------------------------------------------------------------------------------------------------------------------------------------------------------------------------------------------------------------------------------               Os06g0672400_Osat_115469472          RYESQKRRDWHTFGQYLRNHR-PPLELSRCSGAHVLEFLRYLDQFGK---TKVHAAGCPFFG--HPSPPAP-------C-----PCPLRQAWGSLD-ALVGRLRAAFEEHGGR-----------------------PEANPFGARAVRLYLREVRDSQAKARGIAYEKKR-----------------------------------------------------------------------------------------------------------------------------------------------------------------------------------------------------------------------------------------------------------------               Os10g0478000_Osat_297727691          RYESQKRRDWNTFGQYLRNHR-PPLSLARCSGAHVLEFLRYLDQFGK---TKVHAPACPFFG--HPAPPAP-------C-----PCPLRQAWGSLD-ALVGRLRAAYEENGGR-----------------------PENNPFGARAVRLYLREVREHQARARGVSYEKKK-----------------------------------------------------------------------------------------------------------------------------------------------------------------------------------------------------------------------------------------------------------------               LOC100306082_Gmax_351722585          RYESQKRRDWNTFGQYLKNQT-PPVSLSQCNFNHVLEFLRYLDQFGK---TKVHLHGCIFFG--QPDPPAP-------C-----TCPLRQAWGSLD-ALIGRLRAAYEEHGGS-----------------------AETNPFGSGAIRVYLREVKECQAKARGIPYTKKK-----------------------------------------------------------------------------------------------------------------------------------------------------------------------------------------------------------------------------------------------------------------               LOC100306225_Gmax_351721022          RYESQKRRDWNTFGQYLKNQR-PPVPLSQCNCNHVLDFLRYLDQFGK---TKVHLQGCMFYG--QPEPPAP-------C-----TCPLRQAWGSLD-ALTGRLRAAYEENGGS-----------------------PETNPFASGSIRVYLKEIRECQAKARGIPYKKKK-----------------------------------------------------------------------------------------------------------------------------------------------------------------------------------------------------------------------------------------------------------------               LOC100527164_Gmax_351725703          RYENQKRRDWNTFGQYLKNHR-PPLSLSRCSGAHVLEFLRYLDQFGK---TKVHTPICPFYG--HPNPPAP-------C-----PCPLRQAWGSLD-ALIGRLRAAFEENGGK-----------------------PEANPFGARAVRLYLREVRDLQSKARGISYEKKK-----------------------------------------------------------------------------------------------------------------------------------------------------------------------------------------------------------------------------------------------------------------               LOC100527409_Gmax_351721177          RYENQKRRDWNTFGQYLKNHR-PPLSLSRCSGANVLEFLRYLDQFGK---TKVHTPICPFYG--HPNPPAP-------C-----PCPLRQAWGSLD-ALIGRLRAAFEENGGK-----------------------PETNPFGARAVRLYLREVRELQSKARGISYEKKK-----------------------------------------------------------------------------------------------------------------------------------------------------------------------------------------------------------------------------------------------------------------               LOC100527554_Gmax_351722645          RYESQKRRDWNTFGQYLRNQR-PPVALSQCSSNHVLEFLRYLDQFGK---TKVHSQGCLFFG--QTEPPGP-------C-----TCPLRQAWGSLD-ALIGRLRAAYEENGGL-----------------------PETNPFASGTIRVYLREVRDSQAKARGIPYKKKK-----------------------------------------------------------------------------------------------------------------------------------------------------------------------------------------------------------------------------------------------------------------               LOC100775798_Gmax_356537589          RYESQKRRDWNTFLQYLQNHR-PPLTLARCSGAHVIEFLKYLDQFGK---TKVHVTGCPYFG--HPNPPAP-------C-----TCPLKQAWGSLD-ALIGRLRAAYEENGGR-----------------------PESNPFGARAVRTCLREVREGQAKARGIPYEKKK-----------------------------------------------------------------------------------------------------------------------------------------------------------------------------------------------------------------------------------------------------------------               LOC100775838_Gmax_356510740          RYESQKRRDWNTFGQYLKNHR-PPLTLSRCSGAHVLEFLRYLDQFGK---TKVHAETCAYFG--NSHPPGP-------C-----ACPLRQAWGSLD-ALIGRLRAAFEENGGT-----------------------PEMNPFGTRAVRLYLREVRDAQAKARGIAYEKKK-----------------------------------------------------------------------------------------------------------------------------------------------------------------------------------------------------------------------------------------------------------------               LOC100778283_Gmax_356505027          RYENQKRRDWNTFGQYLKNHR-PPLSLSRCSGAHVLEFLRYLDQFGK---TKVHTPICPFYG--HPNPPAP-------C-----PCPLRQAWGSLD-ALIGRLRAAFEENGGK-----------------------PEANPFGARAVRLYLREVRDLQSKARGISYEKKK-----------------------------------------------------------------------------------------------------------------------------------------------------------------------------------------------------------------------------------------------------------------               LOC100778886_Gmax_356549228          RYESQKRRDWNTFGQYLKNQT-PPVSLSQCNFNHVLEFLRYLDQFGK---TKVHLHGCIFFG--QPDPPAP-------C-----TCPLRQAWGSLD-ALIGRLRAAYEERGGS-----------------------PETNPFGSGAIRVYLREVKECQAKARGIPYIKKK-----------------------------------------------------------------------------------------------------------------------------------------------------------------------------------------------------------------------------------------------------------------               LOC100780494_Gmax_356551892          RYESQKRRDWNTFLQYLRNHK-PPLTLARCSGAHVIEFLKYLDQFGK---TKVHILGCPYFG--HPNPPAP-------C-----ACPLKQAWGSLD-ALIGRLRAAFEENGGR-----------------------PESNPFATRAVRIYLREIREGQAKARGIPYEKKK-----------------------------------------------------------------------------------------------------------------------------------------------------------------------------------------------------------------------------------------------------------------               LOC100786135_Gmax_356571190          RYENQKRRDWNTFCQYLRNQR-PPLSLALCSGAHVLEFLQYLDQFGK---TKVHNPPCPFFG--LPNPPAP-------C-----PCPLRQAWGSLD-ALIGRLRAAYEENGGR-----------------------PETNPFGARAVRLYLHDVRDFQAKARGVSYEKKR-----------------------------------------------------------------------------------------------------------------------------------------------------------------------------------------------------------------------------------------------------------------               LOC100789834_Gmax_356535364          RYENQKRRDWNTFGQYLRNHR-PPLSLARCSGAHVLEFLRYLDQFGK---TKVHTQLCPFFG--HPNPPAA-------C-----PCPLRQAWGSLD-ALIGRLRAAFEENGGK-----------------------PEANPFGARAVRLYLREVRDSQAKARGISYEKKK-----------------------------------------------------------------------------------------------------------------------------------------------------------------------------------------------------------------------------------------------------------------               LOC100791424_Gmax_356535206          RYENQKRRDWNTFCQYLRNHR-PPLSLALCSGAHVLEFLHYLDQFGK---TKVHNHPCPFFG--LPNPPAP-------C-----PCPLRQAWGSLD-ALIGRLRAAYEENGGR-----------------------PETNPFGARAVRIYLRDVRDFQAKARGVSYEKKR-----------------------------------------------------------------------------------------------------------------------------------------------------------------------------------------------------------------------------------------------------------------               LOC100798406_Gmax_356524906          RYESQKRRDWNTFGQYLKNHR-PPLTLSRCSGAHVLEFLRYLDQFGK---TKVHAETCGYFG--NSHPPGP-------C-----ACPLRQAWGSLD-ALIGRLRAAFEENGGA-----------------------PEMNPFGTRAVRLYLREVRDAQAKARGIAYEKKK-----------------------------------------------------------------------------------------------------------------------------------------------------------------------------------------------------------------------------------------------------------------               LOC100802715_Gmax_356500896          RYESQKRRDWNTFLQYLRNHK-PPLTLARCSGAHVIEFLKYLDQFGK---TKVHIAGCPYFG--HPNPPAP-------C-----ACPLKQAWGSLD-ALIGRLRAAFEENGGR-----------------------PESNPFATRAVRIYLKEVREGQAKARGIPYEKKK-----------------------------------------------------------------------------------------------------------------------------------------------------------------------------------------------------------------------------------------------------------------               LOC100802937_Gmax_356543912          RYESQKRRDWNTFGQYLKNQR-PPVPLSQCNCNHVLDFLRYLDQFGK---TKVHLQGCMFYG--QPEPPAP-------C-----TCPLRQAWGSLD-ALIGRLRAAYEENGGS-----------------------PETNPFASGSIRVYLKEVRECQAKARGIPYKKKK-----------------------------------------------------------------------------------------------------------------------------------------------------------------------------------------------------------------------------------------------------------------               LOC100803605_Gmax_356537028          RYENQKRRDWNTFGQYLKNHR-PPLSLSRCSGAHVLEFLRYLDQFGK---TKVHTPICPFYG--HPNPPAP-------C-----PCPLRQAWGSLD-ALIGRLRAAFEENGGK-----------------------PETNPFGARAVRLYLREVRELQSKARGISYEKKK-----------------------------------------------------------------------------------------------------------------------------------------------------------------------------------------------------------------------------------------------------------------               LOC100803965_Gmax_356504125          RYENQKRRDWNTFCQYLRNQR-PPLSMAVCGGAHVLEFLQYLDQFGK---TKVHNPTCPFFG--LPNPPAP-------C-----PCPLRQAWGSLD-ALIGRLRAAYEENGGR-----------------------AETNPFGARAVRFYLHDVRDFQAKARGVSYEKKR-----------------------------------------------------------------------------------------------------------------------------------------------------------------------------------------------------------------------------------------------------------------               LOC100806134_Gmax_356543082          RYESQKRRDWNTFGQYLKNQR-PPVPLSQCNCNHVLDFLRYLDQFGK---TKVHLQGCMFYG--QPEPPAP-------C-----ACPLRQAWGSLD-ALIGRLRAAYEENGGS-----------------------PETNPFASGSIRVYLREVRECQAKARGIAYKKKK-----------------------------------------------------------------------------------------------------------------------------------------------------------------------------------------------------------------------------------------------------------------               LOC100806493_Gmax_356544090          RYESQKRRDWNTFGQYLRNQR-PPVALSQCSSNHVLEFLRYLDQFGK---TKVHSQGCLFFG--QTEPPGP-------C-----TCPLRQAWGSLD-ALIGRLRAAYEENGGL-----------------------PETNPFASGAIRVYLREVRDSQSKARGIPYKKKK-----------------------------------------------------------------------------------------------------------------------------------------------------------------------------------------------------------------------------------------------------------------               LOC100806497_Gmax_356576525          RYENQKRRDWNTFGQYLRNHR-PPLSLSRCSGAHVLEFLRYLDQFGK---TKVHTQLCPFFG--HPNPPAP-------C-----PCPLRQAWGSLD-ALIGRLRAAFEENGGK-----------------------PEANPFGARAVRLYLREVRDSQAKARGISYEKKK-----------------------------------------------------------------------------------------------------------------------------------------------------------------------------------------------------------------------------------------------------------------               LOC100810350_Gmax_356569498          RYESQKRRDWNTFLQYLQNHK-PPLTLARCSGAHVIEFLKYLDQFGK---TKVHITGCPYYG--YPNPPAP-------C-----ACPLKQAWGSLD-ALIGRLRAAYEENGGR-----------------------PESNPFGARAVRIYLREVREGQAKARGIPYEKKK-----------------------------------------------------------------------------------------------------------------------------------------------------------------------------------------------------------------------------------------------------------------               LOC100810655_Gmax_356525290          RYESQKRRDWNTFGQYLRNQS-PPVPLSQCNFNHVLDFLRYLDQFGK---TKVHLHGCIFFG--QPTPPAP-------C-----ACPLRQAWGSLD-ALIGRLRAAYEEHGGS-----------------------PETNPFGGGAIRVYLREVKECQAKARGIPYKKKK-----------------------------------------------------------------------------------------------------------------------------------------------------------------------------------------------------------------------------------------------------------------               LOC100815968_Gmax_356515072          RYESQKRRDWNTFGQYLKNQR-PPVPLSQCNCNQVLDFLRYLDQFGK---TKVHLQGCMFYG--QPEPPAP-------C-----TCPLRQAWGSLD-ALIGRLRAAYEENGGS-----------------------PETNPFASGSIRVYLREVRECQAKARGIPYKKKK-----------------------------------------------------------------------------------------------------------------------------------------------------------------------------------------------------------------------------------------------------------------               PHYPADRAFT_52777_Ppat_168001026      RYEAQKRRDWNTFGQYLRNHR-PPLPLARCTGAHVLEFMRYLDQFGK---TKVHIASCSFFG--LPHPPHP-------C-----PCPLRQAWGSLD-ALIGRLRAAFEENGGM-----------------------PESNPFGARQVRLYLREVREMQAKARGIAYEKKK-----------------------------------------------------------------------------------------------------------------------------------------------------------------------------------------------------------------------------------------------------------------               PHYPADRAFT_48231_Ppat_168016540      RYEAQKRRDWNTFGQYLRNHR-PPLALARCTGVHVLEFVRYLDQFGK---TKVHVQSCPFFG--LPHPPHP-------C-----PCPLRQAWGSLD-ALIGRLRAAFEENGGK-----------------------PESNPFGARQVRLYLREVREMQAKARGIAYEKKK-----------------------------------------------------------------------------------------------------------------------------------------------------------------------------------------------------------------------------------------------------------------               PHYPADRAFT_48282_Ppat_168025129      RYEAQKRRDWNTFGQYLRNHR-PPLALARCTGLHVLEFVRYLDQFGK---TKVHIASCSFFG--LPHPPHP-------C-----PCPLRQAWGSLD-ALIGRLRAAFEENGGK-----------------------PESNPFGARQVRLYLREVREMQAKARGIAYEKKK-----------------------------------------------------------------------------------------------------------------------------------------------------------------------------------------------------------------------------------------------------------------               PHYPADRAFT_53176_Ppat_168002068      RYEAQKRRDWNTFGQYLRNHR-PPLALARCTGVHVLEFVHYLDQFGK---TKVHVPSCPFFG--LPHPPHP-------C-----PCPLRQAWGSLD-ALIGRLRAAFEENGGK-----------------------PESNPFGARQVRLYLREVREMQAKARGIAYEKKK-----------------------------------------------------------------------------------------------------------------------------------------------------------------------------------------------------------------------------------------------------------------               PpG1L1_Ppat_270309066                RYEAQKRRDWNTFGQYLRNHR-PPLALARCTGVHVLEFVHYLDQFGK---TKVHVPSCPFFG--LPHPPHP-------C-----PCPLRQAWGSLD-ALIGRLRAAFEENGGK-----------------------PESNPFGARQVRLYLREVREMQAKARGIAYEKKK-----------------------------------------------------------------------------------------------------------------------------------------------------------------------------------------------------------------------------------------------------------------               SELMODRAFT_68566_Smoe_302756799      RYEAQKRRDWNTFGQYLRNHR-PPLTLPRCSGANVLEFLRYLDQFGK---TKIHAPACPFFG--IAHPPAP-------C-----ACPLRQAWGSLD-ALIGRLRAAFEEHGGK-----------------------PESNPFGARAVRLYLREVREMQAKARGIAYEKKK-----------------------------------------------------------------------------------------------------------------------------------------------------------------------------------------------------------------------------------------------------------------               SELMODRAFT_36560_Smoe_302783941      RYEAQKRRDWNTFGQYLKNHR-PPLALSRCSGAHVLEFLRYLDQFGK---TKIHAPACPFFG--LAHPPAP-------C-----ACPLRQAWGSLD-ALIGRLRAAFEEHGGK-----------------------PESNPFGARAVRLYLREVREMQAKARGIAYEKKK-----------------------------------------------------------------------------------------------------------------------------------------------------------------------------------------------------------------------------------------------------------------               SmG1L1_Smoe_270309068                RYEAQKRRDWNTFGQYLKNHR-PPLALSRCSGAHVLEFLRYLDQFGK---TKIHAPACPFFG--LAHPPAP-------C-----ACPLRQAWGSLD-ALIGRLRAAFEEHGGK-----------------------PESNPFGARAVRLYLREVREMQAKARGIAYEKKK-----------------------------------------------------------------------------------------------------------------------------------------------------------------------------------------------------------------------------------------------------------------               ALOG_Sprat_299509744                 RYESQKRRDWNTFGQYLKNHR-PPIAMNKCTSQHVVEFLRYLDQFGK---TKVHNEGCQFYG---TSGGNP-------C-----GCPMKQAWGSLD-SLVWRLRAAYEENGGK-----------------------SENNPFGTRIVRTFLRDVREHQAKARGIAYEKKK-----------------------------------------------------------------------------------------------------------------------------------------------------------------------------------------------------------------------------------------------------------------               SORBIDRAFT_05g008160_Sbic_242068111  RYESQKWRDWNTFLQYLQNHR-PPLKLACCTGAHVIEFLRYLDQFGK---TRVHLEGCDYFG--QPNPPVP-------C-----ACPLRQAWGSLD-ALIGRLRAGYEEFGGR-----------------------PESNPFMAKDVRIYLRDVREAQAKASGISYVKKKP----------------------------------------------------------------------------------------------------------------------------------------------------------------------------------------------------------------------------------------------------------------               ACP30588.1_Brap_227438197            ---------MEAFSEFLSHRQ-PPRSLFESTADDAIDFLLTLP---------------------------------------------PDRLNEDV-NQLSKATF-SEIDGVH------------------------KENPFNSPMVRTFLQERVNPGGGKSEITGRYHD-----------------------------------------------------------------------------------------------------------------------------------------------------------------------------------------------------------------------------------------------------------------               AT4G19500_Atha_240256009             PNRRHSNDDWCSFCEFLRNRI-PPLNPFKCSANDVIDFLRT-----------------------------------------------RQVLGSTE-ALVDRLIFSSEAFGIK-----------------------PEENPFRSQAVTSYLKAARDMTREKECILVFSCH-----------------------------------------------------------------------------------------------------------------------------------------------------------------------------------------------------------------------------------------------------------------               ARALYDRAFT_658157_Alyr_297804202     ITTTALNNDWDSFREFLNNRL-APLT-ISC-AKDVIDFLRM---------------------------------------------RQTSGMEAVE-ALAGYLKAMCEAHGIR-----------------------PMDNPFRSLAVTSYLKSASEMTREKECILVFSCN-----------------------------------------------------------------------------------------------------------------------------------------------------------------------------------------------------------------------------------------------------------------               Lgig1000000655_Lgig                  KYVHQKEKLTSQLTLFLMSVY-STLNLDNASPGVMREFLVWKDSTGK---TKVHLDSCVFRT---QSDKAS-------C-----KCPIRRAASSLD-TLIGQLRAIFRDHGRG-sdwnevl---------------GFGNPMAAPSIKRHLQAVTLEQSK---------------------------------------------------------------------------------------------------------------------------------------------------------------------------------------------------------------------------------------------------------------------------               NEMVEDRAFT_v1g143493_Nvec_156348362  -YKKQKSALERQLSVFLAALS-PPKDVSSASSVDIVKFLISKDAGGR---TTVHVQGCERRG-----R----------C-----ECPRRLASGTVD-SLLGKLRAIYNAIGRT-----------------------NDSNPVAHQVIKDYLKFIRASGVAVVPEQAVPLF-----------------------------------------------------------------------------------------------------------------------------------------------------------------------------------------------------------------------------------------------------------------               DB416162_Apect_93299263              -------------------------------------------------------------------RKPP-------C-----SCPRRLAFGTVV-TKTAQLKAIFQS-MGKqhdwpgd-----------------SGNPVCSDIVNAYVKQIKTEQSIGHTSQVQAKP-----------------------------------------------------------------------------------------------------------------------------------------------------------------------------------------------------------------------------------------------------------------               Lgig1000016045_Lgig                  KYVKQKDRLQTDVEKFLEEYS-KS-TIDKATPHDMRAFLVYKESCGK---TKVHRKDCLVV---SGTGCH--------C-----ECLLDMSANSVD-SLIGKLRAIFRDRGRG-skwdadl---------------GTGNPLASLSIKNHLKALKMEQLQADVIPIHAVPlfldkaaKLDRYLEFYLTRPLL----------IREEYLVRRD--KAFIKFICHSGDRAGDLANLRTDQIKQ--TEKGLLVRMTQ-GKTik------------------------QKLEYFV*---------------------------------------------------------------------------------------------------------------------------------------               Adig1000023598_Adig                  PYERRKSALEQQLFKFLGALS-PPRTMTSCTAQDIVKFLISKDRSGR---TVVHSLSCSKR----G------------C-----SCPKRLAAGSVD-STLGRLRAIFNK-LGR-a---------------------NDSNPASHPLVKDYLKFVREEQAGLAITPSQAV--------PIFFGKFQQLIAHLRDLCSSSVFLSSASKYILVRD--ATFFVVDFLIGDRASDLGRLQSCNVFRLRDREGFLLRFTL-TKNlr--kGPPRSVALIK--fahshv---CPVAWIQYYITVCQclkvpl------------------------DQGYFFRTAERsgsigsnpftgs-----AVNNRL-RKHLS-EAKLYAgetp-------HSFRVEGCQGLTTCGKRPDSVNDLRD--------PSYAELG               BRAFLDRAFT_68991_Bflo_260795011      DYGKKKTALENELVDFLGNSA-PPKDLVTASPKDVCAFLVWKDKGGK---TVVHKVNCKYFG---EKRKTG-------C-----GCPKRLAAGTVD-SIIGQLRAIFTI-SGRgrdwteav---------------CAGNPAAAPVVRQYLKVTKVEQANAMIQPKQAQ--------PVFFDKLTAVCTHITkkmkekevr-HTTLFALARDQLQAFLKIMFFAADRASDLARCKSEELAWLPNEEGILFNHTF-GKTlr--dGTVNAFPILA--gknksm---CPIQGLRLYIRVAQSlkicl------------------------dKGYLFRAINKaqevt-ndpftydAAQYRFKSVI*KEMDC-DEGETL-----------HSFRTGCAITLAATGVPEKQARQHIGWCSNRMP-DHYTGAS               NEMVEDRAFT_v1g218021_Nvec_156358300  --MPNKRLLRRELEEFLEALP-GYVSIATVTPRDICRFLVSKDKDGR---TQVHRLSCRFIV---KKGHFG-------C-----GCPTHLSYKTVD-SYIGKLRAIFHAMGRD-sewdkrl---------------GLGNPAVDKSLKDHLRLITAGQLQARMDKLS----------QLALHLDSEMNKAKR----------NIDRFIIVRD--QAYHKMAFFSEDRPSDLGQIKVAEMLRFPQNDGFLFNHIW-GKTlr--gGDGNVFGVRR--npqlei---CQIRGIEQYMEVARdigidl-------------------------RGYLFRPVT-pdlgv*dsplsssAAEARLKGYL-KDLKA-DEGETL-----------HGFRAGCAITLALTGAELSEIMDHIGWSNRHTA-LFYMQLE               NEMVEDRAFT_v1g220156_Nvec_156352960  PYQKRTSSLLKELERFLDSLT-PPKNLMSASPRDINRFLVWKDEGGR---TKIHKPTCTKYG---SAGSAR-------C-----RCPSRLAAGTVD-SIIGKLRAIFAE-AGRkgewneml---------------NIGNPSSHRSAKGYLTSIREEQAMAHVSPKQAT--------PIFFDKLAKLCRFLRnlvfvekat-SIQRHIHARD--LAFFCLDFFAGDRASGLGRVLTKEALASKDGETIWFRHTY-GKTlrg-gGDTNVFPIKKya*ldpva---CPVANLRLYIKLCDImkinl------------------------rEGYIFRATDGatkvs-dnpfvgsTIANRLKLHL-GKADI-LEGETM-----------HGFRSGCSITLSLLRVSTEDVARHVGWKSTSTA-DYYSQTG               CGI_10005117_Cgig_405958725          SLSKRRDTLKNSLNLFLPLLR-KNCTISNCTPDDSKAFLVWKDDFGK---TPVHKINCTFLG---TKEFGS-------G-----ECPRRFVAGTVS-VMVQNLSEVIYE-MGRGkfwders---------------DTGNPAAAHYIKQYLKPFQEEQEAAHVVP---M--------PMVLGKVKRIASYINNrev---------------------------------------------------------------------------------------------------------------------------------------------------------------------------------------------------------------------------------------               contig_11092_Esil_242186836          ASARSRSATWALFSNFLASRRNGSVSIENAQPKDVVEFLCWLDSCGSRRRTIVHAKHCEAVG---TKDLTA-------CstdkgECSLRYAFDSLRTNHVSKLSMVFEKEMGVvtpwsktm---------------RVGNPVKSELVAQYMAFTTSEQKQAGVLVKQA---------PVILRSHLEKIIFPMqirlqyass-DVERVTLARD--IAFFSVAFSTTKRGVELTNILIQRILRLPNRSGLMCNFQW-GKTqr--dGADHILTVPY-deeyvai---CPVRAVERFIAVGKQvgwdt------------------------tSGYLFPDISEsmqge-aqrgklpVATSRMSEAL-KRYAA-AVGETQgfsl-------HSFRSGGAVSRALAGDSLSTIMQKAYWKSPKTA-WRYMRLM               ORF2_Ddis_167738                     STLKVYSSSYTRFRNFCTLN---SLNPANITLVVFMDYLTHLFKHK-----------------------------------------PPLAFSTIN-GHRSMLNQLLLLRNQTd----------------------IVNDPFITRIMTGIHKLRP--SSAKY---------------KEIWDANQVFKHLSTik--------VIPKYTYTALLNKTLVLCKMFGLARSSDLVKWSFKGLIITP--DSIKGPVIN-AKEqr--sGVVSILELTSlddtnsqv---CPVRHLATYLRASKGrrkphs-----------------------gDSVFIKNEVN-------------RSKLMILTQI-VLSTLSKSGIDIvkfks------HSTRSAMASLLVSNNVPFHVVKKMGRWKSNDTVDTFYDKRI               LOC100369420_Skow_291232955          NTLSKYTGSWLRWKRWCQSNL-SAGAACPAKPLHIAIYLRSLLDN-------------------------------------------ANTVAPMD-SALYSIRWAHSMAGIEs----------------------PTCHPMVRATMEGCRRILA--KPRKA---------------KEPVYPEILATLVSQkn--------LS-SLSDLRL--LCLCLISYAGFMRIGELLSVKISDISFS---DSHMEIKLHKRKNdkfreGSSIIIARSP--knt------CPVRVTEMFISKLGVnvns--------------------------ESFLIRRLVHtkqglkp------HNHLGISYST-ARDLM-MKGIKPlvndisqfgt-HSFRAGGATAAANSNVNERCIARHGGWKSTSSK-DRYICDS               CC1G_14464_Ccin_299750223            STQETYATGLLVYHAFCDKRGIDEHQRAPASPVLLAAFVTSLVGL--------------------------------------------YSGKTIR-NYVFGVRAWHIMHGVRw----------------------VPNDTELEALLRAGERNAPPTSKKAK---------------RTPVTEDHIRRIHAQld--------PNNPL-HAAT--FACLTTTFYAAARLGEFTLPNLNAFDKTHVKPSDIRVEV--DRR-----GQEVTVFHLPrtkssmsgedvSWAQQNGTVDPKSALenhlqvnqpp-------------------pDGPLFAYRTK--ngkkethk-a-LTMKKFLEVV-NQAGA-RANIVKisg--------HCIRIGATLEYLLRGVSFEAMKAKGRWASDAF--LDYLREH               CRE_BPP1_3891895(PDB:1CRX)           HTWKMLLSVCRSWAAWCKLN---NRKWFPAEPEDVRDYLLYLQAR-------------------------------------------GLAVKTIQ-QHLGQLNMLHRRSGLPr----------------------PSDSNAVSLVMRRIRKEN-VD---AGERAKQALA-------FERTDFDQVRSLME-----------NSDRCQDIRN--LAFLGIAYNTLLRIAEIARIRVKDISRTD--GGRMLIHI--GRTktlvsTAGVEKALSL-----------GVTKLVERWISVSGVadd--------------------------pNNYLFCRVRKngvaapsatsq--LSTRALEGIF-EATHRLIYGAKDdsgqrylawsgHSARVGAARDMARAGVSIPEIMQAGGWTNVNIV-MNYIRNL               int_BPlambda_9626273(PDB:1Z1G)       KTLINYMSKIKAIRRGLPD-----APLEDITTKEIAAMLNGYIDE--------------------------------------------GKAASAK-LIRSTLSDAFREAIAEghit--------------------TNHVAATRAAKSEVRRSRL-------------------------TADEYLKIYQAa------------ESSPCWL--RLAMELAVVTGQRVGDLCEMKWSDIV-----DGYLYVEQ--SKT-----GVKIAIPTAL--hidalg---ISMKETLDKCKEILG------------------------------GETIIASTRRep-----------LSSGTVSRYF-MRARK-ASGLSFegdpptf----HELRSLSARLYEKQISD-KFAQHLLGHKSDTMA-SQYRDDR               XerD_Ecol_157829635(PDB:1a0p)        NTLNAYRRDLSMMVEWLHHR---GLTLATAQSDDLQALLAERLEG-------------------------------------------GYKATSSA-RLLSAVRRLFQYLYRE-----------------------KFREDDPSAHLASPKLPQRL---------------------PKDLSEAQVERLLQApl---------IDQPLELRD--KAMLEVLYATGLRVSELVGLTMSDISLR---QGVVRVI---GKG-----NKERLVPLGE-----------EAVYWLETYLEHGRPwllngvs-----------------------IDVLFPSQRAqq-----------MTRQTFWHRI-KHYAV-LAGIDSeklsp------HVLRHAFATHLLNHGADLRVVQMLLGHSDLSTT-QIYTHVA               IntI_Vcho_99031763(PDB:2a3v)         KTIEAYLHWITRYIHFHNK----KHPSLM-GDKEVEEFLTYLAVQ------------------------------------------GKVATKTQS-LALNSLSFLYKEI--------------------------LKTPLSLEIRFQRSQLERKL---------------------PVVLTRDEIRRLLEIv---------------DPKH--QLPIKLLYGSGLRLMECMRLRVQDIDFD---YGAIRIWQ--GKG-----GKNRTVTLAKel---------YPHLKEQIALAKRYYdrdlhqknyggvwlptalkekypnapyefrWHYLFPSFQLsldpesdvmrrhhMNETVLQKAV-RRSAQ-EAGIEKtvtc-------HTLRHSFATHLLEVGADIRTVQEQLGHTDVKTT-QIYTHSG               consensus/90%                        .Yc.pKpp.hpph.paL.s...PP..l.phssscllcFL..bDp.G+---TplH...C..hs................C-----.CP.+bAhGolD-uLlG+LRAhacp.u.........................s..NPhs...lp.aL+.h+..p.bscs..h..p............p.....p..........................bhhh.h.h.s..R.s-h..h..p.h.......u.h......s+......s....h.......................hh......................................lh...................s...h...h-........Gbp............HshR.u.u..h....ss.p.h.....a.ssp.....Y.p..               binding_residues_CRE                 *--**--*--*---------------------------------*---------------------------------------------*-**----*--------------------------------------------------------------*--**---------------------------------------------------------------------------------------------------------------------------------------------------------------------------------------------------------------------------------------------------------------------               binding_residues_lambda_integrase    -*--**--------*---------------------------------------------------------------------------*--*-------------------------------------------------------****----------------------------------------------------------------------------------------------------------------------------------------------------------------------------------------------------------------------------------------------------------------------------------               binding_residues_2a3v                -*---*-*---*------------------------------------------------------------------------------******-*--*------*---------------------------------**-*--------**--------------------------------------------------------------------------------------------------------------------------------------------------------------------------------------------------------------------------------------------------------------------------------                 Species abbreviations are as follows:               Adig : Acropora digitifera; Alyr : Arabidopsis lyrata; Atha : Arabidopsis thaliana; BPP1 : Enterobacteria phage P1; BPlambda : Enterobacteria phage lambda; Bflo : Branchiostoma floridae; Brap : Brassica rapa; Ccin : Coprinopsis cinerea; Cgig : Crassostrea gigas; Ddis : Dictyostelium discoideum; Ecol : Escherichia coli; Esil : Ectocarpus siliculosus; Gmax : Glycine max; Lgig : Lottia gigantea; Nvec : Nematostella vectensis; Ogra : Oryza grandiglumis;               Osat : Oryza sativa; Ppat : Physcomitrella patens; Ppec : Patiria pectinifera; Sbic : Sorghum bicolor; Skow : Saccoglossus kowalevskii; Smoe : Selaginella moellendorffii; Spra : Spirogyra pratensis; Vcho : Vibrio cholerae; Zmay : Zea mays               ```                Back to Contents                 ---                **- Phyletic patterns and domain architectures of the ALOG domain**                 ```               # 184;               293335351       ALOG                                              LOC100381724          246   eukaryota>viridiplantae         Zea mays                             uncharacterized protein LOC100381724 [Zea mays].               226495423       ALOG                                              LOC100276429          247   eukaryota>viridiplantae         Zea mays                             uncharacterized protein LOC100276429 [Zea mays].               226503287       ALOG                                              LOC100276793          305   eukaryota>viridiplantae         Zea mays                             uncharacterized protein LOC100276793 [Zea mays].               226506018       ALOG                                              LOC100275232          204   eukaryota>viridiplantae         Zea mays                             uncharacterized protein LOC100275232 [Zea mays].               226490815       ALOG                                              ZmG1                  256   eukaryota>viridiplantae         Zea mays                             uncharacterized protein LOC100278049 [Zea mays].               226509510       ALOG                                              LOC100278742          204   eukaryota>viridiplantae         Zea mays                             uncharacterized protein LOC100278742 [Zea mays].               239835738       ALOG                                              LOC100273939          247   eukaryota>viridiplantae         Zea mays                             uncharacterized protein LOC100273939 [Zea mays].               195639324       ALOG                                              -                     279   eukaryota>viridiplantae         Zea mays                             hypothetical protein [Zea mays].               219362509       ALOG                                              LOC100217086          210   eukaryota>viridiplantae         Zea mays                             uncharacterized protein LOC100217086 [Zea mays].               226508692       ALOG                                              LOC100274783          202   eukaryota>viridiplantae         Zea mays                             uncharacterized protein LOC100274783 [Zea mays].               212722430       ALOG                                              LOC100192495          277   eukaryota>viridiplantae         Zea mays                             uncharacterized protein LOC100192495 [Zea mays].               212723306       ALOG                                              LOC100192901          201   eukaryota>viridiplantae         Zea mays                             uncharacterized protein LOC100192901 [Zea mays].               195644002       ALOG                                              -                     276   eukaryota>viridiplantae         Zea mays                             hypothetical protein [Zea mays].               259490587       ALOG                                              LOC100304411          213   eukaryota>viridiplantae         Zea mays                             uncharacterized protein LOC100304411 [Zea mays].               308081234       ALOG                                              LOC100502216          247   eukaryota>viridiplantae         Zea mays                             uncharacterized protein LOC100502216 [Zea mays].               219887295       ALOG                                              -                     247   eukaryota>viridiplantae         Zea mays                             unknown [Zea mays].               219362749       ALOG                                              LOC100217012          270   eukaryota>viridiplantae         Zea mays                             uncharacterized protein LOC100217012 [Zea mays].               147776506       ALOG                                              VITISV_034817         177   eukaryota>viridiplantae         Vitis vinifera                       hypothetical protein VITISV_034817 [Vitis vinifera].               297740903       ALOG                                              VIT_00021754001       267   eukaryota>viridiplantae         Vitis vinifera                       unnamed protein product, partial [Vitis vinifera].               225453046       ALOG                                              LOC100260366          181   eukaryota>viridiplantae         Vitis vinifera                       PREDICTED: uncharacterized protein LOC100260366 [Vitis vinifera].               225461669       ALOG                                              LOC100257253          220   eukaryota>viridiplantae         Vitis vinifera                       PREDICTED: uncharacterized protein LOC100257253 [Vitis vinifera].               147795605       ALOG                                              VITISV_012478         175   eukaryota>viridiplantae         Vitis vinifera                       hypothetical protein VITISV_012478 [Vitis vinifera].               359483734       ALOG                                              LOC100254926          149   eukaryota>viridiplantae         Vitis vinifera                       PREDICTED: uncharacterized protein LOC100254926 [Vitis vinifera].               225434496       ALOG                                              LOC100265705          231   eukaryota>viridiplantae         Vitis vinifera                       PREDICTED: uncharacterized protein LOC100265705 [Vitis vinifera].               302143425       ALOG                                              VIT_00020708001       158   eukaryota>viridiplantae         Vitis vinifera                       unnamed protein product, partial [Vitis vinifera].               147768778       ALOG                                              VITISV_019556         231   eukaryota>viridiplantae         Vitis vinifera                       hypothetical protein VITISV_019556 [Vitis vinifera].               147784987       ALOG                                              VITISV_034969         158   eukaryota>viridiplantae         Vitis vinifera                       hypothetical protein VITISV_034969 [Vitis vinifera].               225438109       ALOG                                              LOC100244642          183   eukaryota>viridiplantae         Vitis vinifera                       PREDICTED: uncharacterized protein LOC100244642 [Vitis vinifera].               147781091       ALOG                                              VITISV_036348         177   eukaryota>viridiplantae         Vitis vinifera                       hypothetical protein VITISV_036348 [Vitis vinifera].               225446615       ALOG                                              LOC100266079          167   eukaryota>viridiplantae         Vitis vinifera                       PREDICTED: uncharacterized protein LOC100266079 [Vitis vinifera].               225444171       ALOG                                              LOC100249172          220   eukaryota>viridiplantae         Vitis vinifera                       PREDICTED: uncharacterized protein LOC100249172 [Vitis vinifera].               242063442       ALOG                                              SORBIDRAFT_04g036620  251   eukaryota>viridiplantae         Sorghum bicolor                      hypothetical protein SORBIDRAFT_04g036620 [Sorghum bicolor].               242093890       ALOG                                              SORBIDRAFT_10g027020  258   eukaryota>viridiplantae         Sorghum bicolor                      hypothetical protein SORBIDRAFT_10g027020 [Sorghum bicolor].               242060628       ALOG                                              SORBIDRAFT_04g004470  258   eukaryota>viridiplantae         Sorghum bicolor                      hypothetical protein SORBIDRAFT_04g004470 [Sorghum bicolor].               242073700       ALOG                                              SORBIDRAFT_06g022610  199   eukaryota>viridiplantae         Sorghum bicolor                      hypothetical protein SORBIDRAFT_06g022610 [Sorghum bicolor].               270309064       ALOG                                              SbG1                  271   eukaryota>viridiplantae         Sorghum bicolor                      G1 protein [Sorghum bicolor].               242059117       ALOG                                              SORBIDRAFT_03g038670  215   eukaryota>viridiplantae         Sorghum bicolor                      hypothetical protein SORBIDRAFT_03g038670 [Sorghum bicolor].               242047378       ALOG                                              SORBIDRAFT_02g002650  284   eukaryota>viridiplantae         Sorghum bicolor                      hypothetical protein SORBIDRAFT_02g002650 [Sorghum bicolor].               242090831       ALOG                                              SORBIDRAFT_09g023120  240   eukaryota>viridiplantae         Sorghum bicolor                      hypothetical protein SORBIDRAFT_09g023120 [Sorghum bicolor].               242065808       ALOG                                              SORBIDRAFT_04g026450  208   eukaryota>viridiplantae         Sorghum bicolor                      hypothetical protein SORBIDRAFT_04g026450 [Sorghum bicolor].               242055545       ALOG                                              SORBIDRAFT_03g045430  320   eukaryota>viridiplantae         Sorghum bicolor                      hypothetical protein SORBIDRAFT_03g045430 [Sorghum bicolor].               242090165       ALOG                                              SORBIDRAFT_09g016440  325   eukaryota>viridiplantae         Sorghum bicolor                      hypothetical protein SORBIDRAFT_09g016440 [Sorghum bicolor].               242034181       ALOG                                              SORBIDRAFT_01g019290  205   eukaryota>viridiplantae         Sorghum bicolor                      hypothetical protein SORBIDRAFT_01g019290 [Sorghum bicolor].               270309068       ALOG                                              SmG1L1                225   eukaryota>viridiplantae         Selaginella moellendorffii           G1-like protein [Selaginella moellendorffii].               302783941       ALOG                                              SELMODRAFT_36560      153   eukaryota>viridiplantae         Selaginella moellendorffii           hypothetical protein SELMODRAFT_36560, partial [Selaginella moellendorffii].               302756799       ALOG                                              SELMODRAFT_68566      173   eukaryota>viridiplantae         Selaginella moellendorffii           hypothetical protein SELMODRAFT_68566, partial [Selaginella moellendorffii].               255582505       ALOG                                              RCOM_0499620          169   eukaryota>viridiplantae         Ricinus communis                     conserved hypothetical protein [Ricinus communis].               255579066       ALOG                                              RCOM_0551570          197   eukaryota>viridiplantae         Ricinus communis                     conserved hypothetical protein [Ricinus communis].               255561148       ALOG                                              RCOM_0536430          174   eukaryota>viridiplantae         Ricinus communis                     conserved hypothetical protein [Ricinus communis].               255563158       ALOG                                              RCOM_1015400          180   eukaryota>viridiplantae         Ricinus communis                     conserved hypothetical protein [Ricinus communis].               255561933       ALOG                                              RCOM_0609070          198   eukaryota>viridiplantae         Ricinus communis                     conserved hypothetical protein [Ricinus communis].               255586269       ALOG                                              RCOM_0237740          246   eukaryota>viridiplantae         Ricinus communis                     conserved hypothetical protein [Ricinus communis].               224128744       ALOG                                              POPTRDRAFT_814378     170   eukaryota>viridiplantae         Sorghum trichocarpa                  predicted protein [Populus trichocarpa].               118484985       ALOG                                              -                     170   eukaryota>viridiplantae         Populus trichocarpa                  unknown [Populus trichocarpa].               224079089       ALOG                                              POPTRDRAFT_555675     177   eukaryota>viridiplantae         Populus trichocarpa                  predicted protein [Populus trichocarpa].               224116896       ALOG                                              POPTRDRAFT_660778     177   eukaryota>viridiplantae         Populus trichocarpa                  predicted protein [Populus trichocarpa].               224105897       ALOG                                              POPTRDRAFT_1087573    223   eukaryota>viridiplantae         Populus trichocarpa                  predicted protein [Populus trichocarpa].               224082830       ALOG                                              POPTRDRAFT_205887     162   eukaryota>viridiplantae         Populus trichocarpa                  predicted protein, partial [Populus trichocarpa].               224117036       ALOG                                              POPTRDRAFT_660852     176   eukaryota>viridiplantae         Populus trichocarpa                  predicted protein [Populus trichocarpa].               224064784       ALOG                                              POPTRDRAFT_409144     152   eukaryota>viridiplantae         Populus trichocarpa                  predicted protein, partial [Populus trichocarpa].               118486790       ALOG                                              -                     183   eukaryota>viridiplantae         Populus trichocarpa                  unknown [Populus trichocarpa].               224117042       ALOG                                              POPTRDRAFT_585775     176   eukaryota>viridiplantae         Populus trichocarpa                  predicted protein [Populus trichocarpa].               224055375       ALOG                                              POPTRDRAFT_1068687    228   eukaryota>viridiplantae         Populus trichocarpa                  predicted protein [Populus trichocarpa].               224056771       ALOG                                              POPTRDRAFT_177269     133   eukaryota>viridiplantae         Populus trichocarpa                  predicted protein, partial [Populus trichocarpa].               118482719       ALOG                                              -                     150   eukaryota>viridiplantae         Populus trichocarpa                  unknown [Populus trichocarpa].               224066239       ALOG                                              POPTRDRAFT_412071     149   eukaryota>viridiplantae         Populus trichocarpa                  predicted protein, partial [Populus trichocarpa].               224099917       ALOG                                              POPTRDRAFT_420721     148   eukaryota>viridiplantae         Populus trichocarpa                  predicted protein, partial [Populus trichocarpa].               118486110       ALOG                                              -                     199   eukaryota>viridiplantae         Populus trichocarpa                  unknown [Populus trichocarpa].               224144569       ALOG                                              POPTRDRAFT_248133     139   eukaryota>viridiplantae         Populus trichocarpa                  predicted protein, partial [Populus trichocarpa].               224104927       ALOG                                              POPTRDRAFT_200002     138   eukaryota>viridiplantae         Populus trichocarpa                  predicted protein, partial [Populus trichocarpa].               224112577       ALOG                                              POPTRDRAFT_281212     138   eukaryota>viridiplantae         Populus trichocarpa                  predicted protein, partial [Populus trichocarpa].               118488802       ALOG                                              -                     203   eukaryota>viridiplantae         Populus trichocarpa                  unknown [Populus trichocarpa].               224123548       ALOG                                              POPTRDRAFT_241421     137   eukaryota>viridiplantae         Populus trichocarpa                  predicted protein, partial [Populus trichocarpa].               224115610       ALOG                                              POPTRDRAFT_823134     236   eukaryota>viridiplantae         Populus trichocarpa                  predicted protein [Populus trichocarpa].               383165943       ALOG                                              2_8751_01             134   eukaryota>viridiplantae         Pinus taeda                          hypothetical protein 2_8751_01, partial [Pinus taeda].               361068467       ALOG                                              CL376Contig1_05       106   eukaryota>viridiplantae         Pinus radiata                        hypothetical protein CL376Contig1_05, partial [Pinus radiata].               361068465       ALOG                                              CL376Contig1_05       106   eukaryota>viridiplantae         Pinus lambertiana                    hypothetical protein CL376Contig1_05, partial [Pinus lambertiana].               116780542       ALOG                                              -                     226   eukaryota>viridiplantae         Picea sitchensis                     unknown [Picea sitchensis].               148910397       ALOG                                              -                     235   eukaryota>viridiplantae         Picea sitchensis                     unknown [Picea sitchensis].               224286692       ALOG                                              -                     236   eukaryota>viridiplantae         Picea sitchensis                     unknown [Picea sitchensis].               168016540       ALOG                                              PHYPADRAFT_48231      140   eukaryota>viridiplantae         Physcomitrella patens subsp. patens  predicted protein, partial [Physcomitrella patens subsp. patens].               168001026       ALOG                                              PHYPADRAFT_52777      144   eukaryota>viridiplantae         Physcomitrella patens subsp. patens  predicted protein, partial [Physcomitrella patens subsp. patens].               168002068       ALOG                                              PHYPADRAFT_53176      140   eukaryota>viridiplantae         Physcomitrella patens subsp. patens  predicted protein, partial [Physcomitrella patens subsp. patens].               168025129       ALOG                                              PHYPADRAFT_48282      142   eukaryota>viridiplantae         Physcomitrella patens subsp. patens  predicted protein, partial [Physcomitrella patens subsp. patens].               270309066       ALOG                                              PpG1L1                204   eukaryota>viridiplantae         Physcomitrella patens                G1-like protein [Physcomitrella patens].               47847657        ALOG                                              OSJNOa148N02.6        253   eukaryota>viridiplantae         Oryza sativa Japonica Group          hypothetical protein [Oryza sativa Japonica Group].               51038155        ALOG                                              OSJNBa0052E20.15      238   eukaryota>viridiplantae         Oryza sativa Japonica Group          hypothetical protein [Oryza sativa Japonica Group].               222631925       ALOG                                              OsJ_18887             251   eukaryota>viridiplantae         Oryza sativa Japonica Group          hypothetical protein OsJ_18887 [Oryza sativa Japonica Group].               222631227       ALOG                                              OsJ_18171             265   eukaryota>viridiplantae         Oryza sativa Japonica Group          hypothetical protein OsJ_18171 [Oryza sativa Japonica Group].               125590992       ALOG                                              OsJ_15463             202   eukaryota>viridiplantae         Oryza sativa Japonica Group          hypothetical protein OsJ_15463 [Oryza sativa Japonica Group].               222623268       ALOG                                              OsJ_07578             225   eukaryota>viridiplantae         Oryza sativa Japonica Group          hypothetical protein OsJ_07578 [Oryza sativa Japonica Group].               125580940       ALOG                                              OsJ_05520             275   eukaryota>viridiplantae         Oryza sativa Japonica Group          hypothetical protein OsJ_05520 [Oryza sativa Japonica Group].               222619484       ALOG                                              OsJ_03942             189   eukaryota>viridiplantae         Oryza sativa Japonica Group          hypothetical protein OsJ_03942 [Oryza sativa Japonica Group].               218199066       ALOG                                              OsI_24839             301   eukaryota>viridiplantae         Oryza sativa Indica Group            hypothetical protein OsI_24839 [Oryza sativa Indica Group].               125552681       ALOG                                              OsI_20303             239   eukaryota>viridiplantae         Oryza sativa Indica Group            hypothetical protein OsI_20303 [Oryza sativa Indica Group].               125543059       ALOG                                              OsI_10694             212   eukaryota>viridiplantae         Oryza sativa Indica Group            hypothetical protein OsI_10694 [Oryza sativa Indica Group].               297727691       ALOG                                              Os10g0478000          204   eukaryota>viridiplantae         Oryza sativa Japonica Group          Os10g0478000 [Oryza sativa Japonica Group].               115469472       ALOG                                              Os06g0672400          277   eukaryota>viridiplantae         Oryza sativa Japonica Group          Os06g0672400 [Oryza sativa Japonica Group].               115463333       ALOG                                              Os05g0347400          284   eukaryota>viridiplantae         Oryza sativa Japonica Group          Os05g0347400 [Oryza sativa Japonica Group].               115459426       ALOG                                              Os04g0516200          202   eukaryota>viridiplantae         Oryza sativa Japonica Group          Os04g0516200 [Oryza sativa Japonica Group].               115449465       ALOG                                              Os02g0811000          248   eukaryota>viridiplantae         Oryza sativa Japonica Group          Os02g0811000 [Oryza sativa Japonica Group].               115447383       ALOG                                              Os02g0623400          232   eukaryota>viridiplantae         Oryza sativa Japonica Group          Os02g0623400 [Oryza sativa Japonica Group].               115444445       ALOG                                              Os02g0166800          191   eukaryota>viridiplantae         Oryza sativa Japonica Group          Os02g0166800 [Oryza sativa Japonica Group].               27260935        ALOG                                              OJ1417_E01.118        276   eukaryota>viridiplantae         Oryza sativa Japonica Group          hypothetical protein [Oryza sativa Japonica Group].               15624056        ALOG                                              B1088C09.26           212   eukaryota>viridiplantae         Oryza sativa Japonica Group          hypothetical protein [Oryza sativa Japonica Group].               270309076       ALOG                                              OgG1-2                161   eukaryota>viridiplantae         Oryza grandiglumis                   G1 protein [Oryza grandiglumis].               270309070       ALOG                                              OgG1-1                272   eukaryota>viridiplantae         Oryza grandiglumis                   G1 protein [Oryza grandiglumis].               357452175       ALOG                                              MTR_2g076400          178   eukaryota>viridiplantae         Medicago truncatula                  177 protein [Medicago truncatula].               357441377       ALOG                                              MTR_1g080210          209   eukaryota>viridiplantae         Medicago truncatula                  hypothetical protein MTR_1g080210 [Medicago truncatula].               357474603       ALOG                                              MTR_4g079930          176   eukaryota>viridiplantae         Medicago truncatula                  177 protein [Medicago truncatula].               388517641       ALOG                                              -                     170   eukaryota>viridiplantae         Medicago truncatula                  unknown [Medicago truncatula].               357441159       ALOG                                              MTR_1g075990          183   eukaryota>viridiplantae         Medicago truncatula                  hypothetical protein MTR_1g075990, partial [Medicago truncatula].               388519977       ALOG                                              -                     197   eukaryota>viridiplantae         Medicago truncatula                  unknown [Medicago truncatula].               217074244       ALOG                                              -                     167   eukaryota>viridiplantae         Medicago truncatula                  unknown [Medicago truncatula].               357491035       ALOG                                              MTR_5g072510          257   eukaryota>viridiplantae         Medicago truncatula                  hypothetical protein MTR_5g072510 [Medicago truncatula].               357458123       ALOG                                              MTR_3g031830          223   eukaryota>viridiplantae         Medicago truncatula                  hypothetical protein MTR_3g031830 [Medicago truncatula].               357453693       ALOG                                              MTR_2g092950          186   eukaryota>viridiplantae         Medicago truncatula                  177 protein [Medicago truncatula].               357518963       ALOG                                              MTR_8g086350          185   eukaryota>viridiplantae         Medicago truncatula                  177 protein [Medicago truncatula].               388522259       ALOG                                              -                     184   eukaryota>viridiplantae         Medicago truncatula                  unknown [Medicago truncatula].               388506144       ALOG                                              -                     173   eukaryota>viridiplantae         Lotus japonicus                      unknown [Lotus japonicus].               388506178       ALOG                                              -                     185   eukaryota>viridiplantae         Lotus japonicus                      unknown [Lotus japonicus].               388519073       ALOG                                              -                     179   eukaryota>viridiplantae         Lotus japonicus                      unknown [Lotus japonicus].               388493306       ALOG                                              -                     179   eukaryota>viridiplantae         Lotus japonicus                      unknown [Lotus japonicus].               326523777       ALOG                                              -                     260   eukaryota>viridiplantae         Hordeum vulgare subsp. vulgare       predicted protein [Hordeum vulgare subsp. vulgare].               326492371       ALOG                                              -                     232   eukaryota>viridiplantae         Hordeum vulgare subsp. vulgare       predicted protein [Hordeum vulgare subsp. vulgare].               326534398       ALOG                                              -                     199   eukaryota>viridiplantae         Hordeum vulgare subsp. vulgare       predicted protein [Hordeum vulgare subsp. vulgare].               356525290       ALOG                                              LOC100810655          167   eukaryota>viridiplantae         Glycine max                          PREDICTED: uncharacterized protein LOC100810655 [Glycine max].               356551892       ALOG                                              LOC100780494          218   eukaryota>viridiplantae         Glycine max                          PREDICTED: uncharacterized protein LOC100780494 [Glycine max].               351721177       ALOG                                              LOC100527409          211   eukaryota>viridiplantae         Glycine max                          uncharacterized protein LOC100527409 [Glycine max].               356571190       ALOG                                              LOC100786135          183   eukaryota>viridiplantae         Glycine max                          PREDICTED: uncharacterized protein LOC100786135 [Glycine max].               356535364       ALOG                                              LOC100789834          199   eukaryota>viridiplantae         Glycine max                          PREDICTED: uncharacterized protein LOC100789834 isoform 1 [Glycine max].               356549228       ALOG                                              LOC100778886          181   eukaryota>viridiplantae         Glycine max                          PREDICTED: uncharacterized protein LOC100778886 [Glycine max].               356505027       ALOG                                              LOC100778283          203   eukaryota>viridiplantae         Glycine max                          PREDICTED: uncharacterized protein LOC100778283 [Glycine max].               356576525       ALOG                                              LOC100806497          189   eukaryota>viridiplantae         Glycine max                          PREDICTED: uncharacterized protein LOC100806497 [Glycine max].               356504125       ALOG                                              LOC100803965          184   eukaryota>viridiplantae         Glycine max                          PREDICTED: uncharacterized protein LOC100803965 [Glycine max].               356524906       ALOG                                              LOC100798406          247   eukaryota>viridiplantae         Glycine max                          PREDICTED: uncharacterized protein LOC100798406 [Glycine max].               356500896       ALOG                                              LOC100802715          219   eukaryota>viridiplantae         Glycine max                          PREDICTED: uncharacterized protein LOC100802715 [Glycine max].               351725703       ALOG                                              LOC100527164          203   eukaryota>viridiplantae         Glycine max                          uncharacterized protein LOC100527164 [Glycine max].               356569498       ALOG                                              LOC100810350          229   eukaryota>viridiplantae         Glycine max                          PREDICTED: uncharacterized protein LOC100810350 [Glycine max].               356544090       ALOG                                              LOC100806493          184   eukaryota>viridiplantae         Glycine max                          PREDICTED: uncharacterized protein LOC100806493 [Glycine max].               351722645       ALOG                                              LOC100527554          194   eukaryota>viridiplantae         Glycine max                          uncharacterized protein LOC100527554 [Glycine max].               356537589       ALOG                                              LOC100775798          235   eukaryota>viridiplantae         Glycine max                          PREDICTED: uncharacterized protein LOC100775798 [Glycine max].               356543912       ALOG                                              LOC100802937          175   eukaryota>viridiplantae         Glycine max                          PREDICTED: uncharacterized protein LOC100802937 [Glycine max].               356543082       ALOG                                              LOC100806134          177   eukaryota>viridiplantae         Glycine max                          PREDICTED: uncharacterized protein LOC100806134 [Glycine max].               356537028       ALOG                                              LOC100803605          207   eukaryota>viridiplantae         Glycine max                          PREDICTED: uncharacterized protein LOC100803605 [Glycine max].               356510740       ALOG                                              LOC100775838          236   eukaryota>viridiplantae         Glycine max                          PREDICTED: uncharacterized protein LOC100775838 [Glycine max].               356535206       ALOG                                              LOC100791424          176   eukaryota>viridiplantae         Glycine max                          PREDICTED: uncharacterized protein LOC100791424 [Glycine max].               356515072       ALOG                                              LOC100815968          179   eukaryota>viridiplantae         Glycine max                          PREDICTED: uncharacterized protein LOC100815968 [Glycine max].               351722585       ALOG                                              LOC100306082          181   eukaryota>viridiplantae         Glycine max                          uncharacterized protein LOC100306082 [Glycine max].               351721022       ALOG                                              LOC100306225          175   eukaryota>viridiplantae         Glycine max                          uncharacterized protein LOC100306225 [Glycine max].               357140656       ALOG                                              LOC100826147          207   eukaryota>viridiplantae         Brachypodium distachyon              PREDICTED: uncharacterized protein LOC100826147 [Brachypodium distachyon].               357129415       ALOG                                              LOC100845070          292   eukaryota>viridiplantae         Brachypodium distachyon              PREDICTED: uncharacterized protein LOC100845070 [Brachypodium distachyon].               357131230       ALOG                                              LOC100842541          217   eukaryota>viridiplantae         Brachypodium distachyon              PREDICTED: uncharacterized protein LOC100842541 [Brachypodium distachyon].               357117372       ALOG                                              LOC100846559          279   eukaryota>viridiplantae         Brachypodium distachyon              PREDICTED: uncharacterized protein LOC100846559 [Brachypodium distachyon].               357150235       ALOG                                              LOC100837100          197   eukaryota>viridiplantae         Brachypodium distachyon              PREDICTED: uncharacterized protein LOC100837100 [Brachypodium distachyon].               357143445       ALOG                                              LOC100836663          244   eukaryota>viridiplantae         Brachypodium distachyon              PREDICTED: uncharacterized protein LOC100836663 [Brachypodium distachyon].               357136695       ALOG                                              LOC100822544          272   eukaryota>viridiplantae         Brachypodium distachyon              PREDICTED: uncharacterized protein LOC100822544 [Brachypodium distachyon].               357119445       ALOG                                              LOC100828158          259   eukaryota>viridiplantae         Brachypodium distachyon              PREDICTED: uncharacterized protein LOC100828158 [Brachypodium distachyon].               357167991       ALOG                                              LOC100821276          209   eukaryota>viridiplantae         Brachypodium distachyon              PREDICTED: uncharacterized protein LOC100821276 [Brachypodium distachyon].               15229310        ALOG                                              AT3G04510             201   eukaryota>viridiplantae         Arabidopsis thaliana                 uncharacterized protein [Arabidopsis thaliana].               18406012        ALOG                                              AT2G42610             177   eukaryota>viridiplantae         Arabidopsis thaliana                 uncharacterized protein [Arabidopsis thaliana].               15219952        ALOG                                              AT1G16910             164   eukaryota>viridiplantae         Arabidopsis thaliana                 uncharacterized protein [Arabidopsis thaliana].               15233942        ALOG                                              AT4G18610             191   eukaryota>viridiplantae         Arabidopsis thaliana                 uncharacterized protein [Arabidopsis thaliana].               18390725        ALOG                                              AT1G07090             196   eukaryota>viridiplantae         Arabidopsis thaliana                 uncharacterized protein [Arabidopsis thaliana].               18412120        ALOG                                              AT1G78815             195   eukaryota>viridiplantae         Arabidopsis thaliana                 uncharacterized protein [Arabidopsis thaliana].               18402578        ALOG                                              AT2G31160             219   eukaryota>viridiplantae         Arabidopsis thaliana                 uncharacterized protein [Arabidopsis thaliana].               186510343       ALOG                                              AT3G23290             195   eukaryota>viridiplantae         Arabidopsis thaliana                 uncharacterized protein [Arabidopsis thaliana].               15237680        ALOG                                              AT5G58500             182   eukaryota>viridiplantae         Arabidopsis thaliana                 uncharacterized protein [Arabidopsis thaliana].               15241821        ALOG                                              AT5G28490             190   eukaryota>viridiplantae         Arabidopsis thaliana                 uncharacterized protein [Arabidopsis thaliana].               21592379        ALOG                                              -                     195   eukaryota>viridiplantae         Arabidopsis thaliana                 unknown [Arabidopsis thaliana].               107738137       ALOG                                              -                     195   eukaryota>viridiplantae         Arabidopsis thaliana                 At1g78815 [Arabidopsis thaliana].               297789570       ALOG                                              ARALYDRAFT_920315     193   eukaryota>viridiplantae         Arabidopsis lyrata subsp. lyrata     hypothetical protein ARALYDRAFT_920315 [Arabidopsis lyrata subsp. lyrata].               297822735       ALOG                                              ARALYDRAFT_901988     193   eukaryota>viridiplantae         Arabidopsis lyrata subsp. lyrata     hypothetical protein ARALYDRAFT_901988 [Arabidopsis lyrata subsp. lyrata].               297813085       ALOG                                              ARALYDRAFT_489650     192   eukaryota>viridiplantae         Arabidopsis lyrata subsp. lyrata     light-dependent short hypocotyls 1 [Arabidopsis lyrata subsp. lyrata].               297791123       ALOG                                              ARALYDRAFT_916864     182   eukaryota>viridiplantae         Arabidopsis lyrata subsp. lyrata     hypothetical protein ARALYDRAFT_916864 [Arabidopsis lyrata subsp. lyrata].               297833134       ALOG                                              ARALYDRAFT_477707     197   eukaryota>viridiplantae         Arabidopsis lyrata subsp. lyrata     hypothetical protein ARALYDRAFT_477707 [Arabidopsis lyrata subsp. lyrata].               297789696       ALOG                                              ARALYDRAFT_497295     219   eukaryota>viridiplantae         Arabidopsis lyrata subsp. lyrata     hypothetical protein ARALYDRAFT_497295 [Arabidopsis lyrata subsp. lyrata].               297848996       ALOG                                              ARALYDRAFT_887918     198   eukaryota>viridiplantae         Arabidopsis lyrata subsp. lyrata     hypothetical protein ARALYDRAFT_887918 [Arabidopsis lyrata subsp. lyrata].               297796773       ALOG                                              ARALYDRAFT_919048     181   eukaryota>viridiplantae         Arabidopsis lyrata subsp. lyrata     hypothetical protein ARALYDRAFT_919048 [Arabidopsis lyrata subsp. lyrata].               297804298       ALOG                                              ARALYDRAFT_914818     191   eukaryota>viridiplantae         Arabidopsis lyrata subsp. lyrata     hypothetical protein ARALYDRAFT_914818 [Arabidopsis lyrata subsp. lyrata].               297822853       ALOG                                              ARALYDRAFT_344884     238   eukaryota>viridiplantae         Arabidopsis lyrata subsp. lyrata     hypothetical protein ARALYDRAFT_344884 [Arabidopsis lyrata subsp. lyrata].               297842663       ALOG                                              ARALYDRAFT_895782     191   eukaryota>viridiplantae         Arabidopsis lyrata subsp. lyrata     At1g78815 [Arabidopsis lyrata subsp. lyrata].               297831074       ALOG                                              ARALYDRAFT_898842     195   eukaryota>viridiplantae         Arabidopsis lyrata subsp. lyrata     hypothetical protein ARALYDRAFT_898842 [Arabidopsis lyrata subsp. lyrata].               297824227       ALOG                                              ARALYDRAFT_903634     177   eukaryota>viridiplantae         Arabidopsis lyrata subsp. lyrata     hypothetical protein ARALYDRAFT_903634 [Arabidopsis lyrata subsp. lyrata].               38637158        ALOG                                              P0035F08.32           221   eukaryota>viridiplantae         Oryza sativa Japonica Group          hypothetical protein [Oryza sativa Japonica Group].               40253552        ALOG                                              OSJNBa0049G15.28      213   eukaryota>viridiplantae         Oryza sativa Japonica Group          hypothetical protein [Oryza sativa Japonica Group].               54291813        ALOG                                              OSJNBa0053E01.4       211   eukaryota>viridiplantae         Oryza sativa Japonica Group          hypothetical protein [Oryza sativa Japonica Group].               57899777        ALOG                                              OSJNBa0014K08.18      166   eukaryota>viridiplantae         Oryza sativa Japonica Group          hypothetical protein [Oryza sativa Japonica Group].               13786460        ALOG                                              OSJNBa0076F20.24      126   eukaryota>viridiplantae         Oryza sativa Japonica Group          hypothetical protein [Oryza sativa Japonica Group].               270309072       ALOG                                              OgG1-2                104   eukaryota>viridiplantae         Oryza grandiglumis                   G1 protein [Oryza grandiglumis].               296083665       ALOG                                              VIT_00027827001       103   eukaryota>viridiplantae         Vitis vinifera                       unnamed protein product, partial [Vitis vinifera].               302143632       ALOG                                              VIT_00023521001       92    eukaryota>viridiplantae         Vitis vinifera                       unnamed protein product, partial [Vitis vinifera].               38566542        ALOG                                              -                     89    eukaryota>viridiplantae         Arabidopsis thaliana                 At3g23290 [Arabidopsis thaliana].               302142898       ALOG                                              VIT_00014203001       106   eukaryota>viridiplantae         Vitis vinifera                       unnamed protein product, partial [Vitis vinifera].               357444833       ALOG                                              MTR_1g114020          177   eukaryota>viridiplantae         Medicago truncatula                  hypothetical protein MTR_1g114020 [Medicago truncatula].               297745849       ALOG                                              VIT_00024677001       226   eukaryota>viridiplantae         Vitis vinifera                       unnamed protein product, partial [Vitis vinifera].               358348199       ALOG                                              MTR_119s0026          420   eukaryota>viridiplantae         Medicago truncatula                  hypothetical protein MTR_119s0026 [Medicago truncatula].               53791991        ALOG                                              OJ1212_H09.17         236   eukaryota>viridiplantae         Oryza sativa Japonica Group          hypothetical protein [Oryza sativa Japonica Group].               297744158       ALOG                                              VIT_00028348001       246   eukaryota>viridiplantae         Vitis vinifera                       unnamed protein product, partial [Vitis vinifera].               222636071       ALOG                                              OsJ_22333             244   eukaryota>viridiplantae         Oryza sativa Japonica Group          hypothetical protein OsJ_22333 [Oryza sativa Japonica Group].               # 4;               240256009       TIR+AP-ATPase+ALOG+TIR+AP-ATPase+LRR_repeats      AT4G19500             1309  eukaryota>viridiplantae         Arabidopsis thaliana                 P-loop NTPase and Toll/interleukin-1 receptor (TIR) homology domain protein [Arabidopsis thaliana].               297804202       X+ALOG+TIR+AP-ATPase+LRR_repeats                  ARALYDRAFT_658157     1122  eukaryota>viridiplantae         Arabidopsis lyrata subsp. lyrata     predicted protein [Arabidopsis lyrata subsp. lyrata].               242068111       AP-ATPase+AP-ATPase+ALOG+STYKINase                SORBIDRAFT_05g008160  1345  eukaryota>viridiplantae         Sorghum bicolor                      hypothetical protein SORBIDRAFT_05g008160 [Sorghum bicolor].               227438197       B#+TIR+AP-ATPase+ALOG+LRR_repeats                 -                     1108  eukaryota>viridiplantae         Brassica rapa subsp. pekinensis      disease resistance protein [Brassica rapa subsp. pekinensis].               # 12;               156348362       ALOG                                              NEMVEDRAFT_v1g143493  122   eukaryota>metazoa>cnidaria      Nematostella vectensis               hypothetical protein NEMVEDRAFT_v1g143493, partial [Nematostella vectensis].               156406991       ALOG                                              NEMVEDRAFT_v1g81144   96    eukaryota>metazoa>cnidaria      Nematostella vectensis               predicted protein, partial [Nematostella vectensis].               156384162       ALOG                                              NEMVEDRAFT_v1g105130  83    eukaryota>metazoa>cnidaria      Nematostella vectensis               predicted protein, partial [Nematostella vectensis].               156358300       ALOG+recomb_frag                                  NEMVEDRAFT_v1g218021  558   eukaryota>metazoa>cnidaria      Nematostella vectensis               predicted protein [Nematostella vectensis].               156352960       N6AMT+ALOG+recomb                                 NEMVEDRAFT_v1g220156  672   eukaryota>metazoa>cnidaria      Nematostella vectensis               hypothetical protein NEMVEDRAFT_v1g220156 [Nematostella vectensis].               156351440       ALOG                                              NEMVEDRAFT_v1g140854  97    eukaryota>metazoa>cnidaria      Nematostella vectensis               predicted protein, partial [Nematostella vectensis].               156370246       ALOG                                              NEMVEDRAFT_v1g213033  417   eukaryota>metazoa>cnidaria      Nematostella vectensis               predicted protein [Nematostella vectensis].               260795011       N6AMT+ZnR+ALOG+recombinase_fragment               BRAFLDRAFT_68991     Note 1 eukaryota>metazoa>chordata      Branchiostoma floridae               hypothetical protein BRAFLDRAFT_68991 [Branchiostoma floridae].               Adig1000023598  ALOG+recombinase_fragment                         Adig1000023598        612   eukaryota>cnidaria              Acropora digitifera                  adi_v1.18287               Lgig1000000655  ALOG                                              Lgig1000000655        126   eukaryota>metazoa>mollusca      Lottia gigantea                      gw1.188.17.1               Lgig1000014628  ALOG                                              Lgig1000014628        188   eukaryota>metazoa>mollusca      Lottia gigantea                      fgenesh2_pg.C_sca_114000014               Lgig1000016045  ALOG                                              Lgig1000016045        333   eukaryota>metazoa>mollusca      Lottia gigantea                      fgenesh2_pg.C_sca_274000004               # 3; cDNA/genomic sequences               93299263        ALOG+recombinase                                 -                      676   eukaryota>metazoa>echinodermata Patiria pectinifera                  DB416162 APG Asterina pectinifera 45-hour embryo cDNA library Patiria pectinifera cDNA clone apg11a06 5', mRNA sequence.               242186836       Full element extracted                           -                      Note2 eukaryota>stramenopiles         Ectocarpus siliculosus               FP265341 immatures sporophytes normalised Ectocarpus siliculosus cDNA clone LQ0AAB36YG05 5', mRNA sequence.               260745145       Recombinase                                      -                      888   eukaryota>metazoa>mollusca      Crassostrea gigas                    CU991723 Pascal Favrel cDNA library all stages (wy0aaa) Crassostrea gigas cDNA clone wy0aaa26f07, mRNA sequence.                #Note 1: The sequence in NR is incorrectly reconstructed, see Branchiostoma element to derive a complete sequence               #Note 2: The Ectocarpus siliculosus sequence was first obtained from a partial cDNA, which was used to extract the complete element from the genome. See below for more details               ```                Back to Contents                 ---                **- Structure of the ALOG domain-containing DIRS1-like transposons in Ectocarpus siliculosus**                 ```               element 1                                                <----5' repeat------------------------------------------------------------------------------------------------------------------------------------------------>                                                                                                               <-------------------------------------------------------------------------------------------------------------------------------------------------------------------------------------------------------------------------------------------------------------------------------------------------------------------------------------------------------------------------------------------------------------------------------------------------------------------------------------------------------------------------------------------------------------------------------------------------------------------------------------------------------------------------------------------------------------------------------------------------------------------------------------------------------------------------------------------------------------------------------------------------------------------------------------------------------------------------------------------------------------------------------<---------------------------------------------------------------------------------------------------------------------------------------------------------------------------------<-----frame 3 begins   TM protein with  7 TM helices---------------------------------------------------------------------------------------------------------------------------------------------------------------------------------------------------------------------------------------------------------------------------------------------------------------------------------------------------------------------------------------------------------------------------------------------------------------------------------------------------------------------------------------------------------------------------------------------------------------------------------------------------------------------------------------------------------------------------------------------------------------------------------------------------------------------------------------------------------------------------------------------------------------------------------------------------------------------------------------------------------------------------------------------------------------------------------------------------------------------------------------------------------------------------------------------------------------------------<------------------------------------------------------------------------------------------------------------------------------------------------------------------------------------------------------------frame 3 ends---------->------------------------------------------------------------------------------------------------------------------------------------frame-1-gag-ZnK------------------------------------------------------------------------------------------------------>                                                                                                                                                                                                                                                                                                                                                                                                                                                                                                                                                                                                                                                      <--- frame 2 RT begins-----------------------------------------------------------------------------------------------------------------------------------------------------------------------------------------------------------------------------------------------------------------------------------------------------------------------------------------------------------------------------------------------------------------------------------------------------------------------------------------------------------------------------------------------------------------------------------------------------------------------------------------------------------------------------------------------------------------------------------------------------------------------------------------------------------------------------------------------------------------------------------------------------------------------------------------------------------------------------------------------------------------------------------------------------------------------------------------------------------------------------------------------------------------------------------------------------------------------------------------------------------------------------------------------------------------------------------------------------------------------------------------------------------------------------------------------------------------------------------------------<---RNaseH--------------------------------------------------------------------------------------------------------------------------------------------------------------------------------------------------------------------------------------------------------------------------------------------------------------------------------------------------------------------------------------------------------------------------------------------------------------------------><-- MT continues in frame 2 ---------------------------------------------------------------------------------------------------------------------------------------------------------------------------------------------------------------------------------------------------------------------------------------------------------------------------------------------------------------------------------------------------------------------fs---<-- ZnR+ALOG+integrase in frame 3----------------------------------------------------------------------------------------------------------------------------------------------------------------------------------------------------------------------------------------------------------------------------------------------------------------------------------------------------------------------------------------------------------------------------------------------------------------------------------------------------------------------------------------------------------------------------------------------------------------------------------------------------------------------------------------------------------------------------------------------------------------------------------------------------------------------------------------------------------------------------------------------------------------------------------------------------------------------------------------------------------------------------------------------------------------------------------------------------------------------------------------------------------------------------------------------------------------------------------------------------------------------------------------------------------------------------------------------------------------------------------------------------------------------------------------------------------------------------------------------------------------------------------------------------------------------------------------------------------------->                                                                                                                                                          |                                                                          <--- 3' repeat------------------------------------------------------------------------------------------------------------------------------------------------>               ENA|CABU01011092|CABU01011092.1 GGATTCTCAAAACAGGAAATGAAGAAAGAGGCACGATTTAAGAATTCTCCAGACGTAAAGATCCAGAACAAAGGAGGAGGAAGAACACACTCTCACTTCCTTTTGTCAAACCTGTGCAAGACAGGAAAACAGAGGAAAGGAGAGTCTCCTTTTTGAGACGCTCATCATCCGTCCGCGCCCTCCGCTCTCCTCGAGCGGCGACCCCGACCGTCCGTCCCTCTGGGATTAGTTCCTTTCCCCTGTTTGTGTTTCTCTTCTTCCCGGTTCATCATGGCAACGGTGCCGAGTTTCCAGCTAGATGCCCTCCACGCGCCAGGACCCGAGGCAGTCATCGAGCAGTTATACGACCTCTGCGCGCCGAAGAGTGGCAACGGCGGTGAGCGGGTCATGGACCTGACCCGCCTGGGCGTGCTACCGCACGGACGGCAGCTGGAAACAGCCTCCCTAGACGACCTGTGCCGGATCTTCGGCGCTTTGGTCGACGCCGGCTCAGCCAGCGACAAGCCTGGCATGATGGCGGTCGGCCTTCCCGCGCGTGGGAATGCCGACTCCGTGGCCGTGGGCGACGCCCGCATGAAGGCGGCCCTAGCCCAGGCCCTCCTCCAGTACCTGTGGCGCTGCGACCACCACGGCGGTCTCCGGCTATCGGCGACCGCGGTTCTTCTCCCGGCTCCTCCCACCTTGGCGACCGCACCTTCAGCAGCAGAAGGAGCACCCTCTTCCGCCCCTTCAGCAAGGGCCGGCAGCACACCCTCTTCCCCTTCCTCTGCATCCACGTCGGCTGCGGCGGCAGCGGCTGCCTCGCCGTCTTCCCCCGCCACTACCGCGGGCGAGGCTACCGCGGGCAAGCGCCTCCGGTCGGAGATCTCTGGAGCCGTGGCGGACCTGGAGGAGGAGAGTGTGGCCGGGGATCGCGAGCCTCGAGTGAAGAAGAAGTCGCTCGTGTACACCACAATTTCCAGCAAGGTGAGTCTTGTAGACATTATATGGTTCTTGCTCGTTTTCGGCCGGCCCACGTCCCACAGCAGTCGTCGTCTCTCCCCGTCCCTCATGGATTTCTGCACTGCAGCATTCGTTCGTCGTGCTCTCTCTCAGCATTCCCTGCCGTCATTTTCTTCGACGCAGGCCATAGCTGGCTGCCCGTGGGTTTCATCACGTGGTGCTTACAATATGAGTGGATTTTCCTTTCGGGAACGCCCGCTCGACATATATTTGTTCGTTTTCAACATCCTCTCTTTTTCTTCTCCTCACGGCGCCCGCCTCTTGCCGGTGTGCTCTTGCTTAGGAGATACTGCTGTGTAGCCGTACGTAGGGACGTGGGTCGCCATCTAGTTTCTGTCTCTATGGTAGGTGGTAGGTGTCTATGGTAGGTGTCTTTCTTTTGGGCTATCCGTGAGCGGCGTCACACGCTCGGATTTGTAAAATTGCTTGTTCGTCGCGCACTTGCACCCGTCACCTCTACAACCTACTTGAAACTTACACCCACAACATCTATTGGGTGAGGGCATGCCGCCTCCGCGCGCTAGCCCCTTTTCGTTTCTCTTCTCCCCTGCTATACATCACGTTTCATTTTCGTTTGTGAGGGACTCTTTCGGTTGCTCTCTCCGTGGCATCTCTTTCTGTCAACATGTCGACTTTGGCAGAACAATATGTCAATATATAATATATTATTATATATGTATATTTATATATTTAATATTTCTATGCATATTATTGTATATTGATATATATACATATTGTTCTTCAAAGCCTATATATATCTTGTTCTTCAAGTAGCCGACATCTCTCTCTCTCTATAAATATATTGGCTACCCCCTTTTCGTTTCTCTTCTCCCCTGCTATACATCACGTTTCATTTTCGTTTGTGAGGGACTCTTTCGGTTGCTCTCTCCGTGGCATCTCTTTCTGTCAACATGTCGACTTTGGCAGAACAATATGTAACTATATAATATATTATTATGCATGTATATTTATATATTTAATATTTCTATGCATATTATTATATATTGATATATATACATATTGTTCTTCAAAGCCTATATATATCTTGTTCTTCAAGAAGCCGACATCTCTCTCTCTCTATAAATATATTTCTCCTTGTGCGATCCGTTCGGAGGCAGTTTCAGGTACCGCCTCTCGCCACCTGCTGGGCCCGCAGGGCGAAGAAAGCGGGATGTACCACTTTGTTTTAGAAAGCGCTACGGGCGCCGCATGGCAGCCATCCTGTTTGGAACATGTTTCCACTGTCGTAGGTCCGGCGTTTTCTTTCCCTCACCGTTTTCACCTCGATTTGGTTGTCTCGGCCCGTCTTTTCCTACGCGTTTCTCCGCTATCTCAGCTACCCATCTCTCATGGTTTTTTCTCACTCTCACCGTTTTTCGGCTCGGTTTTCTCAAGCGTTTCTCCGCTTCAGTTCGGTTTTTTCTCAAGCGATTCTCCGCTATCTCAGCTACCCATCTCTCGGGTTTTTCTCTCTCTCACCGTTTTTCAGCTCGGTTTTCTCAAGCGTTTCTCCGCTATCTCAGCTACCCATGTCTCAGGGTGTTTTCTTTCCTTCACCGTTTCTCAACTCGGTTTCTCAAGCGATTCTCCGCTATCTCAGCTACCCATCTCTCGGGTTTTTTCTCCCTCTCACCGTTTTTCAGCTCGGTTTTCTCAAGCGTTTCTCCACTATCTCAGCTACCCGTGTCTCAGGGTTTTTTCTTTCCTTCGCCGTTTTTCAGCTCGGTTTCTCAAGCGATTCTCCGCTATCTCCGCTGCCCATCTCTCGGGTTTTTTCTCCCTCTCACCGTTTTCTCCGCTATCTCAGCTACCCATTTCGCGGGTTTTGTCTTTCTTTCACCGTTTTTCAGCTCGGTTTTCTCACGCGTTTCTCTGCTATCTCAGCTACCCTCCTCTCGGGTGTTTTTTCTTTCTTTCACCGTTTCTCAACTCGGTTTCTCTCAAACGTTTCTCCGCTATCTCCGCTACCCTCTCTCAATTTTTCTTTCTTTCGCCGTATTTCAACTCGGTTTCTCTCCAGTCTCCACTGTTTCACATTTTCCACTCGGTTTCTCGAGCGTTTTCTCCGCTATTTTCTGACGTTTCTCCGCTATCTCCGGTTTTCAGCTCGGTTTCTTTTCTCGAGCGTTTTCTCCGCTATCTCAAATGTTTCAAGTGGATTTTTTCTCAACCGTTTCTCCGTTGTGTCAGCTATCCCTCTCTCAGGGTTGTTTCTTTCACCGTTTTCAGCTCGGTTTTATCCGGGCGTTTTTCTTCGCGTGTCTGGTTCCGATGTGCTTTACCCGTCTCTCCGGGTATCTCTTCTCGCCGTCTCTTTCCACTCGGTTTTTCTCAAGCGTTTCTCTGCTATCTCGCCCTTTCCGCGGCTTTCTTTTTTCGCAATTCTTCCCTTTCAGTTCGCAGCCCTGTGGACGGGTTTGCTTACCAGTGTAAGCGGGCTCACGACTTTCGACGCCTCCGTCAAGCACCAGCGGGTGCTAGAGATCAAAACCAAGGTCCAGGAGCTAGCTAGGCTAGCGGCTATGGACCCCGAGCTGACGGACGAACGCACGTTTGTCGACACGCTCGCCGTGGGTATTGATGCGGCGATGCAATGCGGCCGCCCGGATGCGGCCGCGGTATGGGTACATGACAAACTCATGTCCCAAACCGAGGAGGGTCGTAAAACGAGATCGTGGCCAGGTGGTAAAAGAGCTGTCGGCCATCACCAAGTTTCTGGGGGGGCCCAAGCATTCCTCAGCTCCAGCCGGACCTTGGAGCCCACCCCCCATGCAGCCCATGGCCCTTACCCCGCATTTTGGGGGATATAGCCCCTATGGTATGCAGCCACAGGGGGGGCACTATACCCCGAGCCGGGGACCCGGGGGGCGACACAGGGCCTCTGGCGGCCGCAGTAGCCGAGGGGGTGGCAGCGGGTTTAGCAGAAAGACCGCTTTGTGCAACACTTGCCGCCGTGCCGGCAAGACCGGGGCAGATATCGAACACTCGTTCCGTGTCTGCCCCTTTGTCCAATGCCATAAGTGCCATGGTCGGGGACACGTAATTCAGAATTGCCCCAACTAAATAGTTAGTGATTCGCTGAGCGGTACGCGTTTCTCGTCTGTTGGTTTCCCGGTTCTCGTCGTTCGGGGGGCATCTGGATGTTCAGCGACACGGGACATGTTTCTCTCGTTTCTGCCAAGTTTTCGCGTTCAGTGGGTTTTCGGCCGTTTTTCGGTTTTTCGCCTCGGAGTTTCCTTCATTGGTGAAAGTTCGTTATCTATTGGTTCAATCCACCTTTCTTTCTTTCAGCTCGAGGTTCATAAGGGTTTTCTCAGCCCGATATGATAGCCTTTCCTTTTCGAATGCACGCTCCCACCCACGCATCATGCGCGTCGGAGGACGCGTCGCCCGACCACGTATTTCCGGTCCGATGGGCACCATGTGGACTCACCAATTTCACCGTCCTGACTGATATACTCTGTAACGATTCTCACATTTCGTCACGTGATCCCACATTACCTCCTGAAATTACTGGTCATATACATCTTTCTCGAGCACTCTCCCACGCCTCTGCACCTTTCCCTTTCTTGGTTTGGACTTTGGTCGGCGGTTGACGCGCGGATCGATTCATGTAGATTTACACTGAAATCATCCTTAAACCTAGGTGAACGAAATTTCCACTTTGCGTGCTACTCTCCCCTAGCATATGCGACATAAGTCACACACGTGCAGCCAACAACATACGTTGGTTATGGCAAAACAAGCCCAGTTTTCCGACATCAGAGATTTTCTTCTACACATTTCATGCGTGTCACAGGTCGGATCGGGAGCAGCCTTGTTCCTCGCCGAGAGAGCCGCACGTACCGCACACATTCGCACACAGTTAGTGGGCGGGGACCTGATCAGTGCATCAGCGAAACAGTCAACCGTCGACCTCTCTAATATTCGGCGCCTTCAACAATCTCACTGCCTACCAGACCAAGCTAGGATGTGGGTGACGGACGATTTCGTTCCGGCTCACTTGCAAACAGCGACTGAGTTCTGGCGGGACGAAATTTTACAGGATACTCCCGTTGGAGATCGCAATACCCTCTTGGGGTGGGTCAAAGGGGTCAACGTTTACGACTTCGTAGACACAAAGGCAACAGGCATTTTTCACGGTGCTTCGTACAACGGCGCTGACCTTACGTCAATACATCTGCCGAACCACGTTCCCGACGAACATGTATCATGGGTCACGAGCGAAGTCGCTAAGCTCGCAGCGACAGGTTGCGTTACGAGATGGAAGGACGTCGCCGACGTTAGCGTATACGCCAAACCGCAAATGGTGCTCCCGTTGGGTGTGGAGCCCACGAAACCCAGGTTGATATGGGACGCACGGTGGCTTAATCTCATGTGCCGCCACCACCCTTTCAGCATGGATGGCGTAGGCAAGGTAGCTCAGTGCGCATGGCCCGGAGCTCATCAAGTCACTATTGATCATAAGGCTGGATACCACCACGTTGCCCTAGATAAGGGTTCTTGGCAATATTTTGGCTTCGAGTGGGAAGGCGAGCTGTACGTGTTTACCGTGCTTGCTTTCGGGTGGTGTTCAGCCCCGTTCATATACGCTTCTCTTTCAGAGGCAGTCGCTCGATACCTGCGAGCAAGAGACATTCCCGTGCTAACATGGATAGACGATTTCTATTTGACCAATTTTCGATCCACCCGCACGCTCAGCTATGACGAGCAGCTCAAAGCAGCTCAGACGACCGCGTATGTCGCACTAGAAGTTCTCTATAGCGCGGGATACTTCATTTCGCTGAAGAAGTGTGAGCTCATCCCTACGACGAGCTTGGTTTTCCTGGGGATTATCTGTGACTCCGATAACAGGCGTTTCGAGGTCCCAGAGGACAAACTCGCCAAGCTCGAGGCGATTCTAGTCGACGCTATTGCGTCGGAGTCCATCACCTTTCAGATGTTAGAGAAGCTCGCAGGCAAGTGCACTAGTCTATCGGTAGCGGTTCCGGTGGCGGCTCTTTACACGCACCATATGTACAAGAGCATCGCAGTTTTCCAGCGACGCGGCGGTCGCAAGCCTAGCATGACGATACCAGTCCCAAAGAACAGCGGTCTTATGTTCGAGTTGAAACGCTGGTTGGAAGTTCGTGAGCATTTCAACGGGGCGTCGTGGTACCGTGCCGAGCATAAGCAATTAGCTCTTACCGGGGCATCGGATGCCTCCTCGGGAGGATGGGGGGGACTCATTAGGAGTCCAGGCCAACCCATTTTCAAAGCAGGCGGAGATTTCCCGCTACACGTCGCGCAGCAACATATCAACGTGCAGGAAGGGTACGCTCTCCAGCAGACTCTGCGTCTGTTTTCGGATAGCCAACCTTCTCAGTTGGCCGGGTCCACCCTCATTTCCAAGGTGGATAGCAAAGTCTTACACGACGCCTTTAAGAAAGGGCGGTCTTCCAACACCCTCATGCATGAGATTATTACTGATCTGTTCTGGTTGCAAGTGCGACGCGATTTCACGCTGAAATTGAAGTGGGTGAGCTCCGAGGAAAACGCTGAGGCTGATGGTATTTCTAGACCAGGATCGGACGATTTTGTGAGACTAGATGAGCGGATGTTCGGCGACCTATGCGCGTGGGCGGGTGAGCAGGTGACCATGGATTTAATGGCCACACCCGCCTCGGTTCACAAGCGCTGGGTAGATGGGCGCTGCACTAGTGAGGATCTGCCATTTTACTCCAGATACCATACACAAGGGTGTGCCGGGGTTGACGTGCTTACGCAAAATGTTCGGTTCATGCCTGGTTCAACGACGGAGGAGTGTTTCGGTTTCTGCTTCCCTCCGACGAGCATGGTAGGAGTGTTTCTTCAGCACTTGGAGGAGTGTCGCGCAAAGGCGCTTGTGATCGTCCCAGATCGGAAGCAGTACTGGTTTCCACGGATAGCGGACGCGGCTACGCGATCACGGACGTTGTCGATCGGTAGCGGGGGTGAGTCACCCTTTTTCCGGGTACACCACCAGAAAGGGTCCGAGCGTTTTCATTTCAAACGGTCAGGAATGCTAGCTGTGGTAGTGGATTTCGCCCGGAATGCTAAGTGATGAAGAGTTCGTTCGGCTCGTTCTCAACGTGTCTCCTGTTCATGTTCGGTGGTTTGTGGGACAACGCATTCTTTACCAGGCTCTCCAGGCTGTGTTCACAGCCCTTGGCCTCATCGTGAGCATCTTAGAGGCGATAATTCCCAAGCGAGATCGTCTCGGCGAGGCGAAGCTTCCTCCTCCGCCGCTCGTGAAGGTGTCAGCATCGCGGGCACGGGTCTTATGCCCGGAGTGCCACAGTGAAAACGATGATTCGTTTCGGTTCTGTCAATGGTGCGGGTACTCAATAGCCCAGCACCAACCTCGGACAACACCTCCTCTTCAAGTGGACGAAGAGGCTATTTCAAGGCGATACCAACAATTTCTAAATGCATGGGCGGAGAAAGCTTCCGCGCGCAGTCGGTCGGCCACATGGGCTTTGTTCAGCAACTTCCTAGCATCTCGAAGGAACGGGTCTGTGTCTATTGAAAATGCACAACCAAAGGATGTGGTAGAGTTCCTGTGCTGGTTAGACTCCTGCGGTTCAAGACGGCGTACAATCGTGCATGCTAAACACTGCGAAGCCGTGGGCACCAAAGATCTTACAGCTTGTTCAACAGACAAGGGAGAATGTAGCCTTAGATACGCCTTTGACTCTCTCCGAACAAATCATGTCTCCAAGTTATCCATGGTGTTCGAGAAGGAAATGGGAGTGGTGACACCGTGGAGCAAGACCATGCGGGTTGGTAACCCGGTGAAAAGCGAGCTAGTTGCGCAATATATGGCGTTCACCACAAGTGAGCAAAAGCAGGCGGGAGTATTGGTGAAACAAGCACCGGTCATTCTTCGAAGCCATCTGGAGAAGATTATTTTTCCAATGCAGATCAGGCTTCAATACGCCTCATCCGACGTCGAACGGGTTACGTTGGCTAGGGACATTGCGTTCTTTTCGGTGGCTTTTAGCACAACCAAGAGAGGAGTGGAGCTCACAAATATCCTCATCCAACGTATTTTGCGACTCCCAAATCGAAGCGGCCTCATGTGCAATTTTCAGTGGGGAAAAACCCAACGGGATGGAGCGGATCATATCCTGACCGTACCGTATGATGAGGAATACGTGGCAATCTGCCCAGTTCGAGCGGTAGAGAGGTTTATAGCCGTGGGGAAGCAGGTCGGATGGGACACTACCTCAGGTTACCTTTTCCCAGACATTTCGGAGTCTATGCAAGGTGAAGCGCAGAGAGGCAAGTTGCCGGTGGCTACTAGCAGGATGTCTGAAGCACTAAAGAGATACGCCGCGGCAGTAGGAGAGACCCAAGGGTTCTCTCTACACTCCTTTAGGTCAGGAGGGGCGGTTTCCAGAGCCCTCGCAGGAGATTCTCTATCGACAATCATGCAGAAGGCATATTGGAAAAGTCCAAAGACTGCTTGGCGATACATGCGACTCATGGAAGTAGTGGCTCCAGGATCCGAGGGTACCGCCATGGTCGAGGGAGTTTCAGAGGAACAATACAGGCAGTTGAATGAGTTCGGTTTGAGTGAACAGAGCCGATCGTTGTCAGCGTTTAGCAATAAGCCTTTGCTTTAGGTGGTCTCGTTTGTTTATTTAAATGGTCTTATTTCAGCCATGGTCTCTCCTCAATGCCTCCACATTCATCTGTTTTCTATCAGTAAAGTATGGGATGAGAAAACTCATCTTGTTCGAGTGAGTTTCCTAAGTAAGGGAGAGAAGTGGATACACCGACCGCGGGAGGTGATGCCACTGTTTACATCCCATACTATCTGAGGAGAAGAACTGACTCGAAAATACTGATGGATTCTCAAAACAGGAAATGAAGAAAGAGGCACGATTTAAGAATTCTCCAGACGTAAAGATCCAGAACAAAGGAGGAGGAAGAACACACTCTCACTTCCTTTTGTCAAACCTGTGCAAGACAGGAAAACAGAGGAAAGGAGAGTCTCCTTTTTGAGAC               Frame 1                         G--F--S--K--Q--E--M--K--K--E--A--R--F--K--N--S--P--D--V--K--I--Q--N--K--G--G--G--R--T--H--S--H--F--L--L--S--N--L--C--K--T--G--K--Q--R--K--G--E--S--P--F--*--D--A--H--H--P--S--A--P--S--A--L--L--E--R--R--P--R--P--S--V--P--L--G--L--V--P--F--P--C--L--C--F--S--S--S--R--F--I--M--A--T--V--P--S--F--Q--L--D--A--L--H--A--P--G--P--E--A--V--I--E--Q--L--Y--D--L--C--A--P--K--S--G--N--G--G--E--R--V--M--D--L--T--R--L--G--V--L--P--H--G--R--Q--L--E--T--A--S--L--D--D--L--C--R--I--F--G--A--L--V--D--A--G--S--A--S--D--K--P--G--M--M--A--V--G--L--P--A--R--G--N--A--D--S--V--A--V--G--D--A--R--M--K--A--A--L--A--Q--A--L--L--Q--Y--L--W--R--C--D--H--H--G--G--L--R--L--S--A--T--A--V--L--L--P--A--P--P--T--L--A--T--A--P--S--A--A--E--G--A--P--S--S--A--P--S--A--R--A--G--S--T--P--S--S--P--S--S--A--S--T--S--A--A--A--A--A--A--A--S--P--S--S--P--A--T--T--A--G--E--A--T--A--G--K--R--L--R--S--E--I--S--G--A--V--A--D--L--E--E--E--S--V--A--G--D--R--E--P--R--V--K--K--K--S--L--V--Y--T--T--I--S--S--K--V--S--L--V--D--I--I--W--F--L--L--V--F--G--R--P--T--S--H--S--S--R--R--L--S--P--S--L--M--D--F--C--T--A--A--F--V--R--R--A--L--S--Q--H--S--L--P--S--F--S--S--T--Q--A--I--A--G--C--P--W--V--S--S--R--G--A--Y--N--M--S--G--F--S--F--R--E--R--P--L--D--I--Y--L--F--V--F--N--I--L--S--F--S--S--P--H--G--A--R--L--L--P--V--C--S--C--L--G--D--T--A--V--*--P--Y--V--G--T--W--V--A--I--*--F--L--S--L--W--*--V--V--G--V--Y--G--R--C--L--S--F--G--L--S--V--S--G--V--T--R--S--D--L--*--N--C--L--F--V--A--H--L--H--P--S--P--L--Q--P--T--*--N--L--H--P--Q--H--L--L--G--E--G--M--P--P--P--R--A--S--P--F--S--F--L--F--S--P--A--I--H--H--V--S--F--S--F--V--R--D--S--F--G--C--S--L--R--G--I--S--F--C--Q--H--V--D--F--G--R--T--I--C--Q--Y--I--I--Y--Y--Y--I--C--I--F--I--Y--L--I--F--L--C--I--L--L--Y--I--D--I--Y--T--Y--C--S--S--K--P--I--Y--I--L--F--F--K--*--P--T--S--L--S--L--Y--K--Y--I--G--Y--P--L--F--V--S--L--L--P--C--Y--T--S--R--F--I--F--V--C--E--G--L--F--R--L--L--S--P--W--H--L--F--L--S--T--C--R--L--W--Q--N--N--M--*--L--Y--N--I--L--L--C--M--Y--I--Y--I--F--N--I--S--M--H--I--I--I--Y--*--Y--I--Y--I--L--F--F--K--A--Y--I--Y--L--V--L--Q--E--A--D--I--S--L--S--L--*--I--Y--F--S--L--C--D--P--F--G--G--S--F--R--Y--R--L--S--P--P--A--G--P--A--G--R--R--K--R--D--V--P--L--C--F--R--K--R--Y--G--R--R--M--A--A--I--L--F--G--T--C--F--H--C--R--R--S--G--V--F--F--P--S--P--F--S--P--R--F--G--C--L--G--P--S--F--P--T--R--F--S--A--I--S--A--T--H--L--S--W--F--F--L--T--L--T--V--F--R--L--G--F--L--K--R--F--S--A--S--V--R--F--F--L--K--R--F--S--A--I--S--A--T--H--L--S--G--F--S--L--S--H--R--F--S--A--R--F--S--Q--A--F--L--R--Y--L--S--Y--P--C--L--R--V--F--S--F--L--H--R--F--S--T--R--F--L--K--R--F--S--A--I--S--A--T--H--L--S--G--F--F--S--L--S--P--F--F--S--S--V--F--S--S--V--S--P--L--S--Q--L--P--V--S--Q--G--F--F--F--P--S--P--F--F--S--S--V--S--Q--A--I--L--R--Y--L--R--C--P--S--L--G--F--F--L--P--L--T--V--F--S--A--I--S--A--T--H--F--A--G--F--V--F--L--S--P--F--F--S--S--V--F--S--R--V--S--L--L--S--Q--L--P--S--S--R--V--F--F--L--S--F--T--V--S--Q--L--G--F--S--Q--T--F--L--R--Y--L--R--Y--P--L--S--I--F--L--S--F--A--V--F--Q--L--G--F--S--P--V--S--T--V--S--H--F--P--L--G--F--S--S--V--F--S--A--I--F--*--R--F--S--A--I--S--G--F--Q--L--G--F--F--S--R--A--F--S--P--L--S--Q--M--F--Q--V--D--F--F--S--T--V--S--P--L--C--Q--L--S--L--S--Q--G--C--F--F--H--R--F--Q--L--G--F--I--R--A--F--F--F--A--C--L--V--P--M--C--F--T--R--L--S--G--Y--L--F--S--P--S--L--S--T--R--F--F--S--S--V--S--L--L--S--R--P--F--R--G--F--L--F--S--Q--F--F--P--F--S--S--Q--P--C--G--R--V--C--L--P--V--*--A--G--S--R--L--S--T--P--P--S--S--T--S--G--C--*--R--S--K--P--R--S--R--S--*--L--G--*--R--L--W--T--P--S--*--R--T--N--A--R--L--S--T--R--S--P--W--V--L--M--R--R--C--N--A--A--A--R--M--R--P--R--Y--G--Y--M--T--N--S--C--P--K--P--R--R--V--V--K--R--D--R--G--Q--V--V--K--E--L--S--A--I--T--K--F--L--G--G--P--K--H--S--S--A--P--A--G--P--W--S--P--P--P--M--Q--P--M--A--L--T--P--H--F--G--G--Y--S--P--Y--G--M--Q--P--Q--G--G--H--Y--T--P--S--R--G--P--G--G--R--H--R--A--S--G--G--R--S--S--R--G--G--G--S--G--F--S--R--K--T--A--L--C--N--T--C--R--R--A--G--K--T--G--A--D--I--E--H--S--F--R--V--C--P--F--V--Q--C--H--K--C--H--G--R--G--H--V--I--Q--N--C--P--N--*--I--V--S--D--S--L--S--G--T--R--F--S--S--V--G--F--P--V--L--V--V--R--G--A--S--G--C--S--A--T--R--D--M--F--L--S--F--L--P--S--F--R--V--Q--W--V--F--G--R--F--S--V--F--R--L--G--V--S--F--I--G--E--S--S--L--S--I--G--S--I--H--L--S--F--F--Q--L--E--V--H--K--G--F--L--S--P--I--*--*--P--F--L--F--E--C--T--L--P--P--T--H--H--A--R--R--R--T--R--R--P--T--T--Y--F--R--S--D--G--H--H--V--D--S--P--I--S--P--S--*--L--I--Y--S--V--T--I--L--T--F--R--H--V--I--P--H--Y--L--L--K--L--L--V--I--Y--I--F--L--E--H--S--P--T--P--L--H--L--S--L--S--W--F--G--L--W--S--A--V--D--A--R--I--D--S--C--R--F--T--L--K--S--S--L--N--L--G--E--R--N--F--H--F--A--C--Y--S--P--L--A--Y--A--T--*--V--T--H--V--Q--P--T--T--Y--V--G--Y--G--K--T--S--P--V--F--R--H--Q--R--F--S--S--T--H--F--M--R--V--T--G--R--I--G--S--S--L--V--P--R--R--E--S--R--T--Y--R--T--H--S--H--T--V--S--G--R--G--P--D--Q--C--I--S--E--T--V--N--R--R--P--L--*--Y--S--A--P--S--T--I--S--L--P--T--R--P--S--*--D--V--G--D--G--R--F--R--S--G--S--L--A--N--S--D--*--V--L--A--G--R--N--F--T--G--Y--S--R--W--R--S--Q--Y--P--L--G--V--G--Q--R--G--Q--R--L--R--L--R--R--H--K--G--N--R--H--F--S--R--C--F--V--Q--R--R--*--P--Y--V--N--T--S--A--E--P--R--S--R--R--T--C--I--M--G--H--E--R--S--R--*--A--R--S--D--R--L--R--Y--E--M--E--G--R--R--R--R--*--R--I--R--Q--T--A--N--G--A--P--V--G--C--G--A--H--E--T--Q--V--D--M--G--R--T--V--A--*--S--H--V--P--P--P--P--F--Q--H--G--W--R--R--Q--G--S--S--V--R--M--A--R--S--S--S--S--H--Y--*--S--*--G--W--I--P--P--R--C--P--R--*--G--F--L--A--I--F--W--L--R--V--G--R--R--A--V--R--V--Y--R--A--C--F--R--V--V--F--S--P--V--H--I--R--F--S--F--R--G--S--R--S--I--P--A--S--K--R--H--S--R--A--N--M--D--R--R--F--L--F--D--Q--F--S--I--H--P--H--A--Q--L--*--R--A--A--Q--S--S--S--D--D--R--V--C--R--T--R--S--S--L--*--R--G--I--L--H--F--A--E--E--V--*--A--H--P--Y--D--E--L--G--F--P--G--D--Y--L--*--L--R--*--Q--A--F--R--G--P--R--G--Q--T--R--Q--A--R--G--D--S--S--R--R--Y--C--V--G--V--H--H--L--S--D--V--R--E--A--R--R--Q--V--H--*--S--I--G--S--G--S--G--G--G--S--L--H--A--P--Y--V--Q--E--H--R--S--F--P--A--T--R--R--S--Q--A--*--H--D--D--T--S--P--K--E--Q--R--S--Y--V--R--V--E--T--L--V--G--S--S--*--A--F--Q--R--G--V--V--V--P--C--R--A--*--A--I--S--S--Y--R--G--I--G--C--L--L--G--R--M--G--G--T--H--*--E--S--R--P--T--H--F--Q--S--R--R--R--F--P--A--T--R--R--A--A--T--Y--Q--R--A--G--R--V--R--S--P--A--D--S--A--S--V--F--G--*--P--T--F--S--V--G--R--V--H--P--H--F--Q--G--G--*--Q--S--L--T--R--R--L--*--E--R--A--V--F--Q--H--P--H--A--*--D--Y--Y--*--S--V--L--V--A--S--A--T--R--F--H--A--E--I--E--V--G--E--L--R--G--K--R--*--G--*--W--Y--F--*--T--R--I--G--R--F--C--E--T--R--*--A--D--V--R--R--P--M--R--V--G--G--*--A--G--D--H--G--F--N--G--H--T--R--L--G--S--Q--A--L--G--R--W--A--L--H--*--*--G--S--A--I--L--L--Q--I--P--Y--T--R--V--C--R--G--*--R--A--Y--A--K--C--S--V--H--A--W--F--N--D--G--G--V--F--R--F--L--L--P--S--D--E--H--G--R--S--V--S--S--A--L--G--G--V--S--R--K--G--A--C--D--R--P--R--S--E--A--V--L--V--S--T--D--S--G--R--G--Y--A--I--T--D--V--V--D--R--*--R--G--*--V--T--L--F--P--G--T--P--P--E--R--V--R--A--F--S--F--Q--T--V--R--N--A--S--C--G--S--G--F--R--P--E--C--*--V--M--K--S--S--F--G--S--F--S--T--C--L--L--F--M--F--G--G--L--W--D--N--A--F--F--T--R--L--S--R--L--C--S--Q--P--L--A--S--S--*--A--S--*--R--R--*--F--P--S--E--I--V--S--A--R--R--S--F--L--L--R--R--S--*--R--C--Q--H--R--G--H--G--S--Y--A--R--S--A--T--V--K--T--M--I--R--F--G--S--V--N--G--A--G--T--Q--*--P--S--T--N--L--G--Q--H--L--L--F--K--W--T--K--R--L--F--Q--G--D--T--N--N--F--*--M--H--G--R--R--K--L--P--R--A--V--G--R--P--H--G--L--C--S--A--T--S--*--H--L--E--G--T--G--L--C--L--L--K--M--H--N--Q--R--M--W--*--S--S--C--A--G--*--T--P--A--V--Q--D--G--V--Q--S--C--M--L--N--T--A--K--P--W--A--P--K--I--L--Q--L--V--Q--Q--T--R--E--N--V--A--L--D--T--P--L--T--L--S--E--Q--I--M--S--P--S--Y--P--W--C--S--R--R--K--W--E--W--*--H--R--G--A--R--P--C--G--L--V--T--R--*--K--A--S--*--L--R--N--I--W--R--S--P--Q--V--S--K--S--R--R--E--Y--W--*--N--K--H--R--S--F--F--E--A--I--W--R--R--L--F--F--Q--C--R--S--G--F--N--T--P--H--P--T--S--N--G--L--R--W--L--G--T--L--R--S--F--R--W--L--L--A--Q--P--R--E--E--W--S--S--Q--I--S--S--S--N--V--F--C--D--S--Q--I--E--A--A--S--C--A--I--F--S--G--E--K--P--N--G--M--E--R--I--I--S--*--P--Y--R--M--M--R--N--T--W--Q--S--A--Q--F--E--R--*--R--G--L--*--P--W--G--S--R--S--D--G--T--L--P--Q--V--T--F--S--Q--T--F--R--S--L--C--K--V--K--R--R--E--A--S--C--R--W--L--L--A--G--C--L--K--H--*--R--D--T--P--R--Q--*--E--R--P--K--G--S--L--Y--T--P--L--G--Q--E--G--R--F--P--E--P--S--Q--E--I--L--Y--R--Q--S--C--R--R--H--I--G--K--V--Q--R--L--L--G--D--T--C--D--S--W--K--*--W--L--Q--D--P--R--V--P--P--W--S--R--E--F--Q--R--N--N--T--G--S--*--M--S--S--V--*--V--N--R--A--D--R--C--Q--R--L--A--I--S--L--C--F--R--W--S--R--L--F--I--*--M--V--L--F--Q--P--W--S--L--L--N--A--S--T--F--I--C--F--L--S--V--K--Y--G--M--R--K--L--I--L--F--E--*--V--S--*--V--R--E--R--S--G--Y--T--D--R--G--R--*--C--H--C--L--H--P--I--L--S--E--E--K--N--*--L--E--N--T--D--G--F--S--K--Q--E--M--K--K--E--A--R--F--K--N--S--P--D--V--K--I--Q--N--K--G--G--G--R--T--H--S--H--F--L--L--S--N--L--C--K--T--G--K--Q--R--K--G--E--S--P--F--*--D--               Frame 2                         -D--S--Q--N--R--K--*--R--K--R--H--D--L--R--I--L--Q--T--*--R--S--R--T--K--E--E--E--E--H--T--L--T--S--F--C--Q--T--C--A--R--Q--E--N--R--G--K--E--S--L--L--F--E--T--L--I--I--R--P--R--P--P--L--S--S--S--G--D--P--D--R--P--S--L--W--D--*--F--L--S--P--V--C--V--S--L--L--P--G--S--S--W--Q--R--C--R--V--S--S--*--M--P--S--T--R--Q--D--P--R--Q--S--S--S--S--Y--T--T--S--A--R--R--R--V--A--T--A--V--S--G--S--W--T--*--P--A--W--A--C--Y--R--T--D--G--S--W--K--Q--P--P--*--T--T--C--A--G--S--S--A--L--W--S--T--P--A--Q--P--A--T--S--L--A--*--W--R--S--A--F--P--R--V--G--M--P--T--P--W--P--W--A--T--P--A--*--R--R--P--*--P--R--P--S--S--S--T--C--G--A--A--T--T--T--A--V--S--G--Y--R--R--P--R--F--F--S--R--L--L--P--P--W--R--P--H--L--Q--Q--Q--K--E--H--P--L--P--P--L--Q--Q--G--P--A--A--H--P--L--P--L--P--L--H--P--R--R--L--R--R--Q--R--L--P--R--R--L--P--P--P--L--P--R--A--R--L--P--R--A--S--A--S--G--R--R--S--L--E--P--W--R--T--W--R--R--R--V--W--P--G--I--A--S--L--E--*--R--R--S--R--S--C--T--P--Q--F--P--A--R--*--V--L--*--T--L--Y--G--S--C--S--F--S--A--G--P--R--P--T--A--V--V--V--S--P--R--P--S--W--I--S--A--L--Q--H--S--F--V--V--L--S--L--S--I--P--C--R--H--F--L--R--R--R--P--*--L--A--A--R--G--F--H--H--V--V--L--T--I--*--V--D--F--P--F--G--N--A--R--S--T--Y--I--C--S--F--S--T--S--S--L--F--L--L--L--T--A--P--A--S--C--R--C--A--L--A--*--E--I--L--L--C--S--R--T--*--G--R--G--S--P--S--S--F--C--L--Y--G--R--W--*--V--S--M--V--G--V--F--L--L--G--Y--P--*--A--A--S--H--A--R--I--C--K--I--A--C--S--S--R--T--C--T--R--H--L--Y--N--L--L--E--T--Y--T--H--N--I--Y--W--V--R--A--C--R--L--R--A--L--A--P--F--R--F--S--S--P--L--L--Y--I--T--F--H--F--R--L--*--G--T--L--S--V--A--L--S--V--A--S--L--S--V--N--M--S--T--L--A--E--Q--Y--V--N--I--*--Y--I--I--I--Y--V--Y--L--Y--I--*--Y--F--Y--A--Y--Y--C--I--L--I--Y--I--H--I--V--L--Q--S--L--Y--I--S--C--S--S--S--S--R--H--L--S--L--S--I--N--I--L--A--T--P--F--S--F--L--F--S--P--A--I--H--H--V--S--F--S--F--V--R--D--S--F--G--C--S--L--R--G--I--S--F--C--Q--H--V--D--F--G--R--T--I--C--N--Y--I--I--Y--Y--Y--A--C--I--F--I--Y--L--I--F--L--C--I--L--L--Y--I--D--I--Y--T--Y--C--S--S--K--P--I--Y--I--L--F--F--K--K--P--T--S--L--S--L--Y--K--Y--I--S--P--C--A--I--R--S--E--A--V--S--G--T--A--S--R--H--L--L--G--P--Q--G--E--E--S--G--M--Y--H--F--V--L--E--S--A--T--G--A--A--W--Q--P--S--C--L--E--H--V--S--T--V--V--G--P--A--F--S--F--P--H--R--F--H--L--D--L--V--V--S--A--R--L--F--L--R--V--S--P--L--S--Q--L--P--I--S--H--G--F--F--S--L--S--P--F--F--G--S--V--F--S--S--V--S--P--L--Q--F--G--F--F--S--S--D--S--P--L--S--Q--L--P--I--S--R--V--F--L--S--L--T--V--F--Q--L--G--F--L--K--R--F--S--A--I--S--A--T--H--V--S--G--C--F--L--S--F--T--V--S--Q--L--G--F--S--S--D--S--P--L--S--Q--L--P--I--S--R--V--F--S--P--S--H--R--F--S--A--R--F--S--Q--A--F--L--H--Y--L--S--Y--P--C--L--R--V--F--S--F--L--R--R--F--S--A--R--F--L--K--R--F--S--A--I--S--A--A--H--L--S--G--F--F--S--L--S--P--F--S--P--L--S--Q--L--P--I--S--R--V--L--S--F--F--H--R--F--S--A--R--F--S--H--A--F--L--C--Y--L--S--Y--P--P--L--G--C--F--F--F--L--S--P--F--L--N--S--V--S--L--K--R--F--S--A--I--S--A--T--L--S--Q--F--F--F--L--S--P--Y--F--N--S--V--S--L--Q--S--P--L--F--H--I--F--H--S--V--S--R--A--F--S--P--L--F--S--D--V--S--P--L--S--P--V--F--S--S--V--S--F--L--E--R--F--L--R--Y--L--K--C--F--K--W--I--F--S--Q--P--F--L--R--C--V--S--Y--P--S--L--R--V--V--S--F--T--V--F--S--S--V--L--S--G--R--F--S--S--R--V--W--F--R--C--A--L--P--V--S--P--G--I--S--S--R--R--L--F--P--L--G--F--S--Q--A--F--L--C--Y--L--A--L--S--A--A--F--F--F--R--N--S--S--L--S--V--R--S--P--V--D--G--F--A--Y--Q--C--K--R--A--H--D--F--R--R--L--R--Q--A--P--A--G--A--R--D--Q--N--Q--G--P--G--A--S--*--A--S--G--Y--G--P--R--A--D--G--R--T--H--V--C--R--H--A--R--R--G--Y--*--C--G--D--A--M--R--P--P--G--C--G--R--G--M--G--T--*--Q--T--H--V--P--N--R--G--G--S--*--N--E--I--V--A--R--W--*--K--S--C--R--P--S--P--S--F--W--G--G--P--S--I--P--Q--L--Q--P--D--L--G--A--H--P--P--C--S--P--W--P--L--P--R--I--L--G--D--I--A--P--M--V--C--S--H--R--G--G--T--I--P--R--A--G--D--P--G--G--D--T--G--P--L--A--A--A--V--A--E--G--V--A--A--G--L--A--E--R--P--L--C--A--T--L--A--A--V--P--A--R--P--G--Q--I--S--N--T--R--S--V--S--A--P--L--S--N--A--I--S--A--M--V--G--D--T--*--F--R--I--A--P--T--K--*--L--V--I--R--*--A--V--R--V--S--R--L--L--V--S--R--F--S--S--F--G--G--H--L--D--V--Q--R--H--G--T--C--F--S--R--F--C--Q--V--F--A--F--S--G--F--S--A--V--F--R--F--F--A--S--E--F--P--S--L--V--K--V--R--Y--L--L--V--Q--S--T--F--L--S--F--S--S--R--F--I--R--V--F--S--A--R--Y--D--S--L--S--F--S--N--A--R--S--H--P--R--I--M--R--V--G--G--R--V--A--R--P--R--I--S--G--P--M--G--T--M--W--T--H--Q--F--H--R--P--D--*--Y--T--L--*--R--F--S--H--F--V--T--*--S--H--I--T--S--*--N--Y--W--S--Y--T--S--F--S--S--T--L--P--R--L--C--T--F--P--F--L--G--L--D--F--G--R--R--L--T--R--G--S--I--H--V--D--L--H--*--N--H--P--*--T--*--V--N--E--I--S--T--L--R--A--T--L--P--*--H--M--R--H--K--S--H--T--C--S--Q--Q--H--T--L--V--M--A--K--Q--A--Q--F--S--D--I--R--D--F--L--L--H--I--S--C--V--S--Q--V--G--S--G--A--A--L--F--L--A--E--R--A--A--R--T--A--H--I--R--T--Q--L--V--G--G--D--L--I--S--A--S--A--K--Q--S--T--V--D--L--S--N--I--R--R--L--Q--Q--S--H--C--L--P--D--Q--A--R--M--W--V--T--D--D--F--V--P--A--H--L--Q--T--A--T--E--F--W--R--D--E--I--L--Q--D--T--P--V--G--D--R--N--T--L--L--G--W--V--K--G--V--N--V--Y--D--F--V--D--T--K--A--T--G--I--F--H--G--A--S--Y--N--G--A--D--L--T--S--I--H--L--P--N--H--V--P--D--E--H--V--S--W--V--T--S--E--V--A--K--L--A--A--T--G--C--V--T--R--W--K--D--V--A--D--V--S--V--Y--A--K--P--Q--M--V--L--P--L--G--V--E--P--T--K--P--R--L--I--W--D--A--R--W--L--N--L--M--C--R--H--H--P--F--S--M--D--G--V--G--K--V--A--Q--C--A--W--P--G--A--H--Q--V--T--I--D--H--K--A--G--Y--H--H--V--A--L--D--K--G--S--W--Q--Y--F--G--F--E--W--E--G--E--L--Y--V--F--T--V--L--A--F--G--W--C--S--A--P--F--I--Y--A--S--L--S--E--A--V--A--R--Y--L--R--A--R--D--I--P--V--L--T--W--I--D--D--F--Y--L--T--N--F--R--S--T--R--T--L--S--Y--D--E--Q--L--K--A--A--Q--T--T--A--Y--V--A--L--E--V--L--Y--S--A--G--Y--F--I--S--L--K--K--C--E--L--I--P--T--T--S--L--V--F--L--G--I--I--C--D--S--D--N--R--R--F--E--V--P--E--D--K--L--A--K--L--E--A--I--L--V--D--A--I--A--S--E--S--I--T--F--Q--M--L--E--K--L--A--G--K--C--T--S--L--S--V--A--V--P--V--A--A--L--Y--T--H--H--M--Y--K--S--I--A--V--F--Q--R--R--G--G--R--K--P--S--M--T--I--P--V--P--K--N--S--G--L--M--F--E--L--K--R--W--L--E--V--R--E--H--F--N--G--A--S--W--Y--R--A--E--H--K--Q--L--A--L--T--G--A--S--D--A--S--S--G--G--W--G--G--L--I--R--S--P--G--Q--P--I--F--K--A--G--G--D--F--P--L--H--V--A--Q--Q--H--I--N--V--Q--E--G--Y--A--L--Q--Q--T--L--R--L--F--S--D--S--Q--P--S--Q--L--A--G--S--T--L--I--S--K--V--D--S--K--V--L--H--D--A--F--K--K--G--R--S--S--N--T--L--M--H--E--I--I--T--D--L--F--W--L--Q--V--R--R--D--F--T--L--K--L--K--W--V--S--S--E--E--N--A--E--A--D--G--I--S--R--P--G--S--D--D--F--V--R--L--D--E--R--M--F--G--D--L--C--A--W--A--G--E--Q--V--T--M--D--L--M--A--T--P--A--S--V--H--K--R--W--V--D--G--R--C--T--S--E--D--L--P--F--Y--S--R--Y--H--T--Q--G--C--A--G--V--D--V--L--T--Q--N--V--R--F--M--P--G--S--T--T--E--E--C--F--G--F--C--F--P--P--T--S--M--V--G--V--F--L--Q--H--L--E--E--C--R--A--K--A--L--V--I--V--P--D--R--K--Q--Y--W--F--P--R--I--A--D--A--A--T--R--S--R--T--L--S--I--G--S--G--G--E--S--P--F--F--R--V--H--H--Q--K--G--S--E--R--F--H--F--K--R--S--G--M--L--A--V--V--V--D--F--A--R--N--A--K--*--*--R--V--R--S--A--R--S--Q--R--V--S--C--S--C--S--V--V--C--G--T--T--H--S--L--P--G--S--P--G--C--V--H--S--P--W--P--H--R--E--H--L--R--G--D--N--S--Q--A--R--S--S--R--R--G--E--A--S--S--S--A--A--R--E--G--V--S--I--A--G--T--G--L--M--P--G--V--P--Q--*--K--R--*--F--V--S--V--L--S--M--V--R--V--L--N--S--P--A--P--T--S--D--N--T--S--S--S--S--G--R--R--G--Y--F--K--A--I--P--T--I--S--K--C--M--G--G--E--S--F--R--A--Q--S--V--G--H--M--G--F--V--Q--Q--L--P--S--I--S--K--E--R--V--C--V--Y--*--K--C--T--T--K--G--C--G--R--V--P--V--L--V--R--L--L--R--F--K--T--A--Y--N--R--A--C--*--T--L--R--S--R--G--H--Q--R--S--Y--S--L--F--N--R--Q--G--R--M--*--P--*--I--R--L--*--L--S--P--N--K--S--C--L--Q--V--I--H--G--V--R--E--G--N--G--S--G--D--T--V--E--Q--D--H--A--G--W--*--P--G--E--K--R--A--S--C--A--I--Y--G--V--H--H--K--*--A--K--A--G--G--S--I--G--E--T--S--T--G--H--S--S--K--P--S--G--E--D--Y--F--S--N--A--D--Q--A--S--I--R--L--I--R--R--R--T--G--Y--V--G--*--G--H--C--V--L--F--G--G--F--*--H--N--Q--E--R--S--G--A--H--K--Y--P--H--P--T--Y--F--A--T--P--K--S--K--R--P--H--V--Q--F--S--V--G--K--N--P--T--G--W--S--G--S--Y--P--D--R--T--V--*--*--G--I--R--G--N--L--P--S--S--S--G--R--E--V--Y--S--R--G--E--A--G--R--M--G--H--Y--L--R--L--P--F--P--R--H--F--G--V--Y--A--R--*--S--A--E--R--Q--V--A--G--G--Y--*--Q--D--V--*--S--T--K--E--I--R--R--G--S--R--R--D--P--R--V--L--S--T--L--L--*--V--R--R--G--G--F--Q--S--P--R--R--R--F--S--I--D--N--H--A--E--G--I--L--E--K--S--K--D--C--L--A--I--H--A--T--H--G--S--S--G--S--R--I--R--G--Y--R--H--G--R--G--S--F--R--G--T--I--Q--A--V--E--*--V--R--F--E--*--T--E--P--I--V--V--S--V--*--Q--*--A--F--A--L--G--G--L--V--C--L--F--K--W--S--Y--F--S--H--G--L--S--S--M--P--P--H--S--S--V--F--Y--Q--*--S--M--G--*--E--N--S--S--C--S--S--E--F--P--K--*--G--R--E--V--D--T--P--T--A--G--G--D--A--T--V--Y--I--P--Y--Y--L--R--R--R--T--D--S--K--I--L--M--D--S--Q--N--R--K--*--R--K--R--H--D--L--R--I--L--Q--T--*--R--S--R--T--K--E--E--E--E--H--T--L--T--S--F--C--Q--T--C--A--R--Q--E--N--R--G--K--E--S--L--L--F--E--T-               Frame 3                         --I--L--K--T--G--N--E--E--R--G--T--I--*--E--F--S--R--R--K--D--P--E--Q--R--R--R--K--N--T--L--S--L--P--F--V--K--P--V--Q--D--R--K--T--E--E--R--R--V--S--F--L--R--R--S--S--S--V--R--A--L--R--S--P--R--A--A--T--P--T--V--R--P--S--G--I--S--S--F--P--L--F--V--F--L--F--F--P--V--H--H--G--N--G--A--E--F--P--A--R--C--P--P--R--A--R--T--R--G--S--H--R--A--V--I--R--P--L--R--A--E--E--W--Q--R--R--*--A--G--H--G--P--D--P--P--G--R--A--T--A--R--T--A--A--G--N--S--L--P--R--R--P--V--P--D--L--R--R--F--G--R--R--R--L--S--Q--R--Q--A--W--H--D--G--G--R--P--S--R--A--W--E--C--R--L--R--G--R--G--R--R--P--H--E--G--G--P--S--P--G--P--P--P--V--P--V--A--L--R--P--P--R--R--S--P--A--I--G--D--R--G--S--S--P--G--S--S--H--L--G--D--R--T--F--S--S--R--R--S--T--L--F--R--P--F--S--K--G--R--Q--H--T--L--F--P--F--L--C--I--H--V--G--C--G--G--S--G--C--L--A--V--F--P--R--H--Y--R--G--R--G--Y--R--G--Q--A--P--P--V--G--D--L--W--S--R--G--G--P--G--G--G--E--C--G--R--G--S--R--A--S--S--E--E--E--V--A--R--V--H--H--N--F--Q--Q--G--E--S--C--R--H--Y--M--V--L--A--R--F--R--P--A--H--V--P--Q--Q--S--S--S--L--P--V--P--H--G--F--L--H--C--S--I--R--S--S--C--S--L--S--A--F--P--A--V--I--F--F--D--A--G--H--S--W--L--P--V--G--F--I--T--W--C--L--Q--Y--E--W--I--F--L--S--G--T--P--A--R--H--I--F--V--R--F--Q--H--P--L--F--F--F--S--S--R--R--P--P--L--A--G--V--L--L--L--R--R--Y--C--C--V--A--V--R--R--D--V--G--R--H--L--V--S--V--S--M--V--G--G--R--C--L--W--*--V--S--F--F--W--A--I--R--E--R--R--H--T--L--G--F--V--K--L--L--V--R--R--A--L--A--P--V--T--S--T--T--Y--L--K--L--T--P--T--T--S--I--G--*--G--H--A--A--S--A--R--*--P--L--F--V--S--L--L--P--C--Y--T--S--R--F--I--F--V--C--E--G--L--F--R--L--L--S--P--W--H--L--F--L--S--T--C--R--L--W--Q--N--N--M--S--I--Y--N--I--L--L--Y--M--Y--I--Y--I--F--N--I--S--M--H--I--I--V--Y--*--Y--I--Y--I--L--F--F--K--A--Y--I--Y--L--V--L--Q--V--A--D--I--S--L--S--L--*--I--Y--W--L--P--P--F--R--F--S--S--P--L--L--Y--I--T--F--H--F--R--L--*--G--T--L--S--V--A--L--S--V--A--S--L--S--V--N--M--S--T--L--A--E--Q--Y--V--T--I--*--Y--I--I--M--H--V--Y--L--Y--I--*--Y--F--Y--A--Y--Y--Y--I--L--I--Y--I--H--I--V--L--Q--S--L--Y--I--S--C--S--S--R--S--R--H--L--S--L--S--I--N--I--F--L--L--V--R--S--V--R--R--Q--F--Q--V--P--P--L--A--T--C--W--A--R--R--A--K--K--A--G--C--T--T--L--F--*--K--A--L--R--A--P--H--G--S--H--P--V--W--N--M--F--P--L--S--*--V--R--R--F--L--S--L--T--V--F--T--S--I--W--L--S--R--P--V--F--S--Y--A--F--L--R--Y--L--S--Y--P--S--L--M--V--F--S--H--S--H--R--F--S--A--R--F--S--Q--A--F--L--R--F--S--S--V--F--S--Q--A--I--L--R--Y--L--S--Y--P--S--L--G--F--F--S--L--S--P--F--F--S--S--V--F--S--S--V--S--P--L--S--Q--L--P--M--S--Q--G--V--F--F--P--S--P--F--L--N--S--V--S--Q--A--I--L--R--Y--L--S--Y--P--S--L--G--F--F--L--P--L--T--V--F--Q--L--G--F--L--K--R--F--S--T--I--S--A--T--R--V--S--G--F--F--L--S--F--A--V--F--Q--L--G--F--S--S--D--S--P--L--S--P--L--P--I--S--R--V--F--S--P--S--H--R--F--L--R--Y--L--S--Y--P--F--R--G--F--C--L--S--F--T--V--F--Q--L--G--F--L--T--R--F--S--A--I--S--A--T--L--L--S--G--V--F--S--F--F--H--R--F--S--T--R--F--L--S--N--V--S--P--L--S--P--L--P--S--L--N--F--S--F--F--R--R--I--S--T--R--F--L--S--S--L--H--C--F--T--F--S--T--R--F--L--E--R--F--L--R--Y--F--L--T--F--L--R--Y--L--R--F--S--A--R--F--L--F--S--S--V--F--S--A--I--S--N--V--S--S--G--F--F--L--N--R--F--S--V--V--S--A--I--P--L--S--G--L--F--L--S--P--F--S--A--R--F--Y--P--G--V--F--L--R--V--S--G--S--D--V--L--Y--P--S--L--R--V--S--L--L--A--V--S--F--H--S--V--F--L--K--R--F--S--A--I--S--P--F--P--R--L--S--F--F--A--I--L--P--F--Q--F--A--A--L--W--T--G--L--L--T--S--V--S--G--L--T--T--F--D--A--S--V--K--H--Q--R--V--L--E--I--K--T--K--V--Q--E--L--A--R--L--A--A--M--D--P--E--L--T--D--E--R--T--F--V--D--T--L--A--V--G--I--D--A--A--M--Q--C--G--R--P--D--A--A--A--V--W--V--H--D--K--L--M--S--Q--T--E--E--G--R--K--T--R--S--W--P--G--G--K--R--A--V--G--H--H--Q--V--S--G--G--A--Q--A--F--L--S--S--S--R--T--L--E--P--T--P--H--A--A--H--G--P--Y--P--A--F--W--G--I--*--P--L--W--Y--A--A--T--G--G--A--L--Y--P--E--P--G--T--R--G--A--T--Q--G--L--W--R--P--Q--*--P--R--G--W--Q--R--V--*--Q--K--D--R--F--V--Q--H--L--P--P--C--R--Q--D--R--G--R--Y--R--T--L--V--P--C--L--P--L--C--P--M--P--*--V--P--W--S--G--T--R--N--S--E--L--P--Q--L--N--S--*--*--F--A--E--R--Y--A--F--L--V--C--W--F--P--G--S--R--R--S--G--G--I--W--M--F--S--D--T--G--H--V--S--L--V--S--A--K--F--S--R--S--V--G--F--R--P--F--F--G--F--S--P--R--S--F--L--H--W--*--K--F--V--I--Y--W--F--N--P--P--F--F--L--S--A--R--G--S--*--G--F--S--Q--P--D--M--I--A--F--P--F--R--M--H--A--P--T--H--A--S--C--A--S--E--D--A--S--P--D--H--V--F--P--V--R--W--A--P--C--G--L--T--N--F--T--V--L--T--D--I--L--C--N--D--S--H--I--S--S--R--D--P--T--L--P--P--E--I--T--G--H--I--H--L--S--R--A--L--S--H--A--S--A--P--F--P--F--L--V--W--T--L--V--G--G--*--R--A--D--R--F--M--*--I--Y--T--E--I--I--L--K--P--R--*--T--K--F--P--L--C--V--L--L--S--P--S--I--C--D--I--S--H--T--R--A--A--N--N--I--R--W--L--W--Q--N--K--P--S--F--P--T--S--E--I--F--F--Y--T--F--H--A--C--H--R--S--D--R--E--Q--P--C--S--S--P--R--E--P--H--V--P--H--T--F--A--H--S--*--W--A--G--T--*--S--V--H--Q--R--N--S--Q--P--S--T--S--L--I--F--G--A--F--N--N--L--T--A--Y--Q--T--K--L--G--C--G--*--R--T--I--S--F--R--L--T--C--K--Q--R--L--S--S--G--G--T--K--F--Y--R--I--L--P--L--E--I--A--I--P--S--W--G--G--S--K--G--S--T--F--T--T--S--*--T--Q--R--Q--Q--A--F--F--T--V--L--R--T--T--A--L--T--L--R--Q--Y--I--C--R--T--T--F--P--T--N--M--Y--H--G--S--R--A--K--S--L--S--S--Q--R--Q--V--A--L--R--D--G--R--T--S--P--T--L--A--Y--T--P--N--R--K--W--C--S--R--W--V--W--S--P--R--N--P--G--*--Y--G--T--H--G--G--L--I--S--C--A--A--T--T--L--S--A--W--M--A--*--A--R--*--L--S--A--H--G--P--E--L--I--K--S--L--L--I--I--R--L--D--T--T--T--L--P--*--I--R--V--L--G--N--I--L--A--S--S--G--K--A--S--C--T--C--L--P--C--L--L--S--G--G--V--Q--P--R--S--Y--T--L--L--F--Q--R--Q--S--L--D--T--C--E--Q--E--T--F--P--C--*--H--G--*--T--I--S--I--*--P--I--F--D--P--P--A--R--S--A--M--T--S--S--S--K--Q--L--R--R--P--R--M--S--H--*--K--F--S--I--A--R--D--T--S--F--R--*--R--S--V--S--S--S--L--R--R--A--W--F--S--W--G--L--S--V--T--P--I--T--G--V--S--R--S--Q--R--T--N--S--P--S--S--R--R--F--*--S--T--L--L--R--R--S--P--S--P--F--R--C--*--R--S--S--Q--A--S--A--L--V--Y--R--*--R--F--R--W--R--L--F--T--R--T--I--C--T--R--A--S--Q--F--S--S--D--A--A--V--A--S--L--A--*--R--Y--Q--S--Q--R--T--A--V--L--C--S--S--*--N--A--G--W--K--F--V--S--I--S--T--G--R--R--G--T--V--P--S--I--S--N--*--L--L--P--G--H--R--M--P--P--R--E--D--G--G--D--S--L--G--V--Q--A--N--P--F--S--K--Q--A--E--I--S--R--Y--T--S--R--S--N--I--S--T--C--R--K--G--T--L--S--S--R--L--C--V--C--F--R--I--A--N--L--L--S--W--P--G--P--P--S--F--P--R--W--I--A--K--S--Y--T--T--P--L--R--K--G--G--L--P--T--P--S--C--M--R--L--L--L--I--C--S--G--C--K--C--D--A--I--S--R--*--N--*--S--G--*--A--P--R--K--T--L--R--L--M--V--F--L--D--Q--D--R--T--I--L--*--D--*--M--S--G--C--S--A--T--Y--A--R--G--R--V--S--R--*--P--W--I--*--W--P--H--P--P--R--F--T--S--A--G--*--M--G--A--A--L--V--R--I--C--H--F--T--P--D--T--I--H--K--G--V--P--G--L--T--C--L--R--K--M--F--G--S--C--L--V--Q--R--R--R--S--V--S--V--S--A--S--L--R--R--A--W--*--E--C--F--F--S--T--W--R--S--V--A--Q--R--R--L--*--S--S--Q--I--G--S--S--T--G--F--H--G--*--R--T--R--L--R--D--H--G--R--C--R--S--V--A--G--V--S--H--P--F--S--G--Y--T--T--R--K--G--P--S--V--F--I--S--N--G--Q--E--C--*--L--W--*--W--I--S--P--G--M--L--S--D--E--E--F--V--R--L--V--L--N--V--S--P--V--H--V--R--W--F--V--G--Q--R--I--L--Y--Q--A--L--Q--A--V--F--T--A--L--G--L--I--V--S--I--L--E--A--I--I--P--K--R--D--R--L--G--E--A--K--L--P--P--P--P--L--V--K--V--S--A--S--R--A--R--V--L--C--P--E--C--H--S--E--N--D--D--S--F--R--F--C--Q--W--C--G--Y--S--I--A--Q--H--Q--P--R--T--T--P--P--L--Q--V--D--E--E--A--I--S--R--R--Y--Q--Q--F--L--N--A--W--A--E--K--A--S--A--R--S--R--S--A--T--W--A--L--F--S--N--F--L--A--S--R--R--N--G--S--V--S--I--E--N--A--Q--P--K--D--V--V--E--F--L--C--W--L--D--S--C--G--S--R--R--R--T--I--V--H--A--K--H--C--E--A--V--G--T--K--D--L--T--A--C--S--T--D--K--G--E--C--S--L--R--Y--A--F--D--S--L--R--T--N--H--V--S--K--L--S--M--V--F--E--K--E--M--G--V--V--T--P--W--S--K--T--M--R--V--G--N--P--V--K--S--E--L--V--A--Q--Y--M--A--F--T--T--S--E--Q--K--Q--A--G--V--L--V--K--Q--A--P--V--I--L--R--S--H--L--E--K--I--I--F--P--M--Q--I--R--L--Q--Y--A--S--S--D--V--E--R--V--T--L--A--R--D--I--A--F--F--S--V--A--F--S--T--T--K--R--G--V--E--L--T--N--I--L--I--Q--R--I--L--R--L--P--N--R--S--G--L--M--C--N--F--Q--W--G--K--T--Q--R--D--G--A--D--H--I--L--T--V--P--Y--D--E--E--Y--V--A--I--C--P--V--R--A--V--E--R--F--I--A--V--G--K--Q--V--G--W--D--T--T--S--G--Y--L--F--P--D--I--S--E--S--M--Q--G--E--A--Q--R--G--K--L--P--V--A--T--S--R--M--S--E--A--L--K--R--Y--A--A--A--V--G--E--T--Q--G--F--S--L--H--S--F--R--S--G--G--A--V--S--R--A--L--A--G--D--S--L--S--T--I--M--Q--K--A--Y--W--K--S--P--K--T--A--W--R--Y--M--R--L--M--E--V--V--A--P--G--S--E--G--T--A--M--V--E--G--V--S--E--E--Q--Y--R--Q--L--N--E--F--G--L--S--E--Q--S--R--S--L--S--A--F--S--N--K--P--L--L--*--V--V--S--F--V--Y--L--N--G--L--I--S--A--M--V--S--P--Q--C--L--H--I--H--L--F--S--I--S--K--V--W--D--E--K--T--H--L--V--R--V--S--F--L--S--K--G--E--K--W--I--H--R--P--R--E--V--M--P--L--F--T--S--H--T--I--*--G--E--E--L--T--R--K--Y--*--W--I--L--K--T--G--N--E--E--R--G--T--I--*--E--F--S--R--R--K--D--P--E--Q--R--R--R--K--N--T--L--S--L--P--F--V--K--P--V--Q--D--R--K--T--E--E--R--R--V--S--F--L--R--H               |                element 2                                                <------------------------------------------------------------------------------------------------------------------------------------------------------------->                                                                                                               <----------------------------------------------------------------------------------------------------------------------------------------------------------------------------------------------------------------------------------------------------------------------------------------------------------------------------------------------------------------------------------------------------------------------------------------------------------------------------------------------------------------------------------------------------------------------------------------------------------------------------------------------------------------------------------------------------------------------------------------------------------------------------------------------------------------------------------------------------------------------------------------------------------------------------------------------------------------------------------------------------------------------------------<---------------------------------------------------------------------------------------------------------------------------------------------------------------------------------<-------------------------------------------------------------------------------------------------------------------------------------------------------------------------------------------------------------------------------------------------------------------------------------------------------------------------------------------------------------------------------------------------------------------------------------------------------------------------------------------------------------------------------------------------------------------------------------------------------------------------------------------------------------------------------------------------------------------------------------------------------------------------------------------------------------------------------------------------------------------------------------------------------------------------------------------------------------------------------------------------------------------------------------------------------------------------------->------------------------------------->--------------------------------------------------------------------------------------------<-------------------------------------------------------------------------------------------------------------------------------------------------------------------------------------------------------------------------------------------------------------------------------------------------------------------------------------------------------------------------------------------------------------------------------------------------------------------------------------------------------------------------------------------------------->                                                                                                                                                                                                                                                                                                                                                                                                                                                                                                                                                                                                                                                   <------------------------------------------------------------------------------------------------------------------------------------------------------------------------------------------------------------------------------------------------------------------------------------------------------------------------------------------------------------------------------------------------------------------------------------------------------------------------------------------------------------------------------------------------------------------------------------------------------------------------------------------------------------------------------------------------------------------------------------------------------------------------------------------------------------------------------------------------------------------------------------------------------------------------------------------------------------------------------------------------------------------------------------------------------------------------------------------------------------------------------------------------------------------------------------------------------------------------------------------------------------------------------------------------------------------------------------------------------------------------------------------------------------------------------------------------------------------------------------------------------------------------------------------------------------------------------------------------------------------------------------------------------------------------------------------------------------------------------------------------------------------------------------------------------------------------------------------------------------------------------------------------------------------------------------------------------------------------------------------------------------------------------------------------------------------------------------------------------------------------------------------------------------------------------------------------------------------------------------------------------------------------------------------------------------------------------------------------------------------------------------------------------------------------------------------------------------------>                                                                                                                                                                                                                                                                                                                                                                                                                                                                                                                                                     <-----------------------------------------------------------------3'repeat------------------------------------------------------------------------------------>               ENA|CABU01010653|CABU01010653.1 GGATTCTCAAAACAGGAAATGAAGAAAGAGGCACGATTTAAGAATTCTCCAGACGTAAAGATCCAGAACAAAGGAGGAGGAAGAACACACTCTCACTTCCTTTTGTCAAACCTGTGCAAGACAGGAAAACAGAGGAAAGGAGAGTCTCCTTTTTGAGACGCTCATCATCCGTCCGCGCCCTCCGCTCTCCTCGAGCGGCGACCGCGACCGTCCGTCCCTCTGGGATTAGTTCCTTTCCCCTGTTTGTGTTTCTCTTCTTCCCGGTTCATCATGGCAACGGTGCCGAGTTTCCAGCTAGATGCCCTCCACGCGCCAGGACCCGAGGCAGTCATCGAGCAGTTATACGACCTCTGCGCGCCGAAGAGTGGCAACGGCGGTGAGCGGGTCATGGACCTGACCCGCCTGGGCGTGCTACCGCACGGACGGCAGCTGGAAACAGCCTCCCTAGACGACCTGTGCCGGATCTTCGGCGCTTTGGTCGACGCCGGCTCAGCCAGCGACAAGCCTGGCATGATGGCGGTCGGCCTTCCCGCGCGTGGGAATGCCGACTCCGTGGCCGTGGGCGACGCCCGCATGAAGGCGGCCCTAGCCCAGGCCCTCCTCCAGTACCTGTGGCGCTGCGACCACCACGGCGGTCTCCGGCTATCGGCGACCGCGGTTCTTCTCCCGGCTCCTCCCACCTTGGCGACCGCACCTTCAGCAGCAGAAGGAGCACCCTCTTCCGCCCCTTCAGCAAGGGCCGGCAGCACACCCTCTTCCCCTTCCTCTGCATCCACGTCGGCTGCGGCGGCAGCGGCTGCCTCGCCGTCTTCCCCCGCCACTACCGCGGGCGAGGCTACCGCGGGCAAGCGCCTCCGGTCGGAGATCTCTGGAGCCGTGGCGGACCTGGAGGAGGAGAGTGTGGCCGGGGATCGCGAGCCTCGAGTGAAGAAGAAGTCGCTCGTGTACACCACAATTTCCAGCAAGGTGAGTCTTGTAGACATTATATGGTTCTTGCTCGTTTTCGGCCGGCCCACGTCCCACAGCAGTCGTCGTCTCTCCCCGTCCCTCATGGATTTCTGCACTGCAGCATTCGTTCGTCGTGCTCTCTCTCAGCATTCCCTGCCGTCATTTTCTTCGACGCAGGCCATAGCTGGCTGCCCGTGGGTTTCATCACGTGGTGCTTACAATATGAGTGGATTTTCCTTTCGGGAACGCCCGCTCGACATATATTTGTTCGTTTCAACATCCTCTCTTTTTCTTCTCCTCACGGCGCCCGCCTCTTGCCGGTGTGCTCTTGCTTAGGAGATACTGCTGTGTAGCCGTACGTAGGGACGTGGGTCGCCATCTAGTTTCTGTCTCTATGGTAGGTGGTAGGTGTCTATGGTAGGTGTCTTTCTTTTGGGCTATCCGTGAGCGGCGTCACACGCTCGGATTTGTAAAATTGCTTGTTCGTCGCGCACTTGCACCCGTCATTTCTACAACCTACTTGAAACTTACACCCACAACATCTATTGGGTGAGGGCATGCCGCCTCCGCGCGCTAGCCCCTTTTCGTTTCTCTTCTCCCCTGCTATACATCACGTTTCATTTTCGTTTGTGAGGGACTCTTTCGGTTGCTCTCTCCGTGGCATCTCTTTCTGTCAACATGTCGACTTTGGCAGAACAATATGTAAATATATGATATATTATTATATATGTATATTTATATATTTAATATTTCTATGCATATTATTGTATATTGATATATATACATATTGTTCTTCAAAGCCTATATATATCTTGTTCTTCAAGTAGCCGACATCTCTCTCTCTCTATAAATATATTGGCTACCCCCTTTTCGTTTCTCTTCTCCCCTGCTATACATCACGTTTCATTTTCGTTTGTGAGGGACTCTTTCGGTTGCTCTCTCCGTGGCATCTCTTTCTGTCAACATGTCGACTTTGGCAGAACAATATGTAAATATATAATATATTATTATGCATGTATATTTATATATTTTATATTTCTATGCATATTATTATATATTGATATATATACATATTGTTCTTCAAAGCCTATATATATCTTGTTCTTCAAGAAGCCGACATCTCTCTCTCTCTATAAATATATTTCTCCTTGTGCGATCCGTTCGGAGGCAGTTTCAGGTACCGCCTCTCGCCACCTGCTGGGCCCGCAGGGCGAAGAAAGCGGGATGTACCACTTTGTTTTAGGAAGCGCTACGGGCGCCGCATGGCAGCCATCCTGTTTGGAACATGTTTCCACTGTCGTAGGTCCGGCGTTTTCTTTCCCTCACCGTTTTCACCTCGATTTGGTTGTCTCGGCCCGTCTTTTCCTACGCGTTTCTCCGCTATCTCAGCTACCCATCTCTCATGGTTTTTTCTCACTCTCACCGTTTTTCGGCTCGGTTTTCTCAAGCGTTTCTCCGCTTCAGTTCGGTTTTTTCTCAAGCGATTCTCCGCTATCTCAGCTACCCATCTCTCGGGTTTTTTCTCTCTCTCACCGTTTTTCAGCTCGGTTTTCTCAAGCGTTTCTCCGCTATCTCAGCTACCCATGTCTCAGGGTGTTTTCTTTCCTTCACCGTTTCTCAACTCGGTTTCTCAAGCGATTCTCCGCTATCTCAGCTACCCATCTCTCGGGTTTTTTCTCCCTCTCACCGTTTTTCAGCTCGGTTTTCTCAAGCGTTTCTCCACTATCTCAGCTACCCGTGTCTCAGGGTTTTTTCTTTCCTTCGCCGTTTTTCAGCTCGGTTTCTCAAGCGATTCTCCGCTATCTCCGCTGCCCATCTCTCGGGTTTTTTCTCCCTCTCACCGTTTTCTCCGCTATCTCAGCTACCCATTTCGCGGGTTTTGTCTTTCTTTCACCGTTTTTCAGCTCGGTTTTCTCACGCGTTTCTCTGCTATCTCAGCTACCCTCCTCTCGGGTGTTTTTCTTTCTTTCACCGTTTCTCAACTCGGTTTCTCTCAAACGTTTCTCCGCTATCTCCGCTACCCTCTCTCAATTTTTCTTTCTTTCGCCGTATTTCAACTCGGTTTCTCTCCAGTCTCCACTGTTTCACATTTTCCACTCGGTTTCTCGAGCGTTTTCTCCGCTATTTTCTGACGTTTCTCCGCTATCTCCGGTTTTCAGCTCGGTTTCTTTTCTCGAGCGTTTTCTCCGCTATCTCAAATGTTTCAAGTGGATTTTTTCTCAACCGTTTCTCCGTTGTGTCAGCTATCCCTCTCTCAGGGTTGTTTCTTTCACCGTTTTCAGCTCGGTTTTATCCGGCGTTTTTCTTCGCGTGTCTGGTTCCGATGTGCTTTACCCGTCTCTCCGGGTATCTCTTCTCGCCGTCTCTTTCCACTCGGTTTTTCTCAAGCGTTTCTCTGCTATCTCGCCCTTTCCGCGGCTTTCTTTTTTCGCAATTCTTCCCTTTTAGTTCGCAGCCCTGTGGACGGGTTTGCTTACCAGTGTAAGCGGGCTCACGACTTTCGACGCCTCCGTCAAGCACCAGCGGGTGCTAGAGATCAAAACCAAGGTCCAGGAGCTAGCTAGGCTAGCGGCTATGGACCCCGAGCTGACGGACGAACGCACGTTTGTCGACACGCTCGCCGTGGGTATTGATGCGGCGATGCAATGCGGCCGCCCGGATGCGGCCGCGGTATGGGTACATGACAAACTCATGTCCCAAACCGAGGAGGGTCGTAAAACAGATCGTGGCCAGGTGGTAAAAGAGCTGTCGGCCATCACCAAGTTTCTGGGGGGGCCCAAGCATTCCTCAGCTCCAGCCGGACCTTGGAGCCCACCCCCCATGCAGCCCATGGCCCTTACCCCGCATTTTGGGGGATATAGCCCCTATGGTATGCAGCCACAGGGGGGGCACTATACCCCGAGCCGGGGACCCGGGGGGCGACACAGGGCCTCTGGCGGCCGCAGTAGCCGAGGGGGTGGCAGCGGGTTTAGCAGACAGACCGCTTTGTGCAACACTTGCCGCCGTGCCGGCAAGACCGGGGCAGATATCGAACACTCGTTCCGTGTCTGCCCCTTTGTCCAATGCCATAAGTGCCATGGTCGGGGACACGTAATTCAGAATTGCCCCAACTAAATAGTTAGTGATTCGCTGAGCGGTACGCGTTTCTCGTCTGTTGGTTTCCCGGTTCTCGTCGTTCGGGGGGCATCTGGATGTTCAGCGACACGGGACATGTTTCTCTCGTTTCTGCCAAGTTTTCGCGTTCAGTGGGTTTTCGGCCGTTTTTCGGTTTTTCGCCTCGGAGTTTCCTTCATTGGTGAAAGTTCGTTATCTATTGGTTCAATCCACCTTTCTTTCTTTCAGCTCGAGGTTCATAAGGGTTTTCTCAGCCCGATATGATAGCCTTTCCTTTTCGAATGCACGCTCCCACCCACGCATCATGCGCGTCGGAGGACGCGTCGCCCGACCACGTATTTCCGGTCCGATGGGCACCATGTGGACTCACCAATTTCACCGTCCTGACTGATATACTCTGTAACGATTCTCACATTTCGTCACGTGATCCCACATTACCTCCTGAAATTACTGGTCATATACATCTTTCTCGAGCACTCTCCCACGCCTCTGCACCTTTCCCTTTCTTGGTTTGGACTTTGGTCGGCGGTTGACGCGCGGATCGATTCATGTAGATTTACACTGAAATCATCCTTAAACCTAGGTGAACGAAATTTCCACTTTGCGTGCTACTCTCCCCTAGCATATGCGACATAAGTCACACACGTGCAGCCAACAACATACGTTGGTTATGGCAAAACAAGCCCAGTTTTCCGACATCAGAGATTTTCTTCTACACATTTCATGCGTGTCACCGGTCGGATCGGGAGCAGCCTTGTTCCTCGCCGAGAGAGCCGCACGTACCGCACACATTCGCACACAGTTAGTGGGCGGGGACCTGATCAGTGCATCAGCGAAACAGTCAACCGTCGACCTCTCTAATATTCGGCGCCTTCAACAATCTCACTGCCTACCAGACCAAGCTAGGATGTGGGTGACGGACGATTTCGTTCCGGCTCACTTGCAAACAGCGACTGAGTTCTGGCGGGACGAAATTTTACAGGATACTCCCGTTGGAGATCGCAATACCCTCTTGGGGTGGGTCAAAGGGGTCAACGTTTACGACTTCGTAGACACAAAGGCAACAGGCATTTTTCACGGTGCTTCGTACAACGGCGCTGACCTTACGTCAATACATCTGCCGAACCACGTTCCCGACGAACATGTATCATGGGTCACGAGCGAAGTCGCTAAGCTCGCAGCGACAGGTTGCGTTGCGAGATGGAAGGACGTCGCCGACGTTAGCAAACCGCAAATGGTGCTCCCGTTGGGTGTGGAGCCCACGAAACCCAGGTTGATATGGGACGCACGGTGGCTTAATCTCATGTGCCGCCACCACCCTTTCAGCATGGATGGCGTAGGCAAGGTAGCTCAGTGCGCATGGCCCGGAGCTCATCAAGTCACTATTGATCATAAGGCTGGATACCACCACGTTGCCCTAGATAAGGGTTCTTGGCAATATTTTGGCTTCGAGTGGGAAGGCGAGCTGTACGTGTTTACCGTGCTTGCTTTCGGGTGGTGTTCAGCCCCGTTCATATACGCTTCTCTTTCAGAGGCAGTCGCTCGATACCTGCGAGCAAGAGACATTCCCGTGCTAACATGGATAGACGATTTCTATTTGACCAATTTTCGATCCACCCGCACGCTCCGCTATGACGAGCAGCTCAAAGCAGCTCAGACGACCGCGTATGTCGCACTAGAAGTTCTCTATCGCGCGGGATACTTCATTTCGCTGAAGAAGTGTGAGCTCATCCCTACGACGAGCTTGGTTTTCCTGGGGATTATCTGTGACTCCGATAACAGGCGTTTCGAGGTCCCAGAGGACAAACTCGCCAAGCTCGAGGCGATTCTAGTCGACGCTATTGCGTCGGAGTCCATCACCTTTCAGATGTTAGAGAAGCTCGCAGGCAAGTGCACTAGTCTATCGGTAGCGGTTCCGGTGGCGGCTCTTTACACGCACCATATGTACAAGAGCATCGCAGTTTTCCAGCGACGCGGCGGTCGCAAGCCTAGCATGACGATACCAGTCCCAAAGAACAGCGGTCTTATGTTCGAGTTGAAACGCTGGTTGGAAGTTCGTGAGCATTTCAACGGGGCGTCGTGGTACCGTGCCGAGCATAAGCAATTAGCTCTTACCGGGGCATCGGATGCCTCCTCGGGAGGATGGGGGGGACTCATTAGGAGTCCAGGCCAACCCATTTTCAAAGCAGGCGGAGATTTCCCGCTACACGTCGCGCAGCAACATATCAACGTGCAGGAAGGGTACGCTCTCCAGCAGACTCTGCGTCTGTTTTCGGATAGCCAACCTTCTCAGTTGGCCGGGTCCACCCTCATTTCCAAGGTGGATAGCAAAGTCTTACACGACGCCTTTAAGAAAGGGCGGTCTTCCAACACCCTCATGCATGAGATTATTACTGATCTGTTCTGGTTGCAAGTGCGACGCGATTTCACGCTGAAATTGAAGTGGGTGAGCTCCGAGGAAAACGCTGAGGCTGATGGTATTTCTAGACCAGGATCGGACGATTTTGTGAGACTAGATGAGCGGATGTTCGGCGACCTATGCGCGTGGGCGGGTGAGCAGGTGACCATGGATTTAATGGCCACACCCGCCTCGGTTCACAAGCGCTGGGTAGATGGGCGCTGCACTAGTGAGGATCTGCCATTTTACTCCAGATACCATACACAAGGGTGTGCCGGGGTTGACGTGCTTACGCAAAATGTTCGGTTCATGCCTGGTTCAACGACGGAGGAGTGTTTCGGTTTCTGCTTCCCTCCGACGAGCATGGTAGGAGTGTTTCTTCAGCACTTGGAGGAGTGTCGCGCAAAGGCGCTTGTGATCGTCCCAGATCGGAAGCAGTACTGGTTTCCACGGATAGCGGACGCGGCTACGCGATCACGGACGTTGTCGATCGGTAGCGGGGGTGAGTCACCCTTTTTCCGGGTACACCACCAGAAAGGGTCCGAGCGTTTTCATTTCAAACGGTCAGGAATGCTAGCTGTGGTAGTGGATTTCGCCCGGAATGCTAAGTGATGAAGAGTTCGTTCGGCTCGTTCTCAACGTGTCTCCTGTTCATGTTCGGTGGTTTGTGGGACAACGCATTCTTTACCAGGCTCTCCAGGCTGTGTTCACAGCCCTTGGCCTCATCGTGAGCATCTTAGAGGCGATAATTCCCAAGCGAGATCGTCTCGGCGAGGCGAAGCTTCCTCCTCCGCCGCTCGTGAAGGTGTCAGCATCGCGGGCACGGGTCTTATGCCCGGAGTGCCACAGTGAAAACGATGATTCGTTTCGGTTCTGTCAATGGTGCGGGTACTCAATAGCCCAGCACCAACCTCGGACAACACCTCCTCTTCAAGTGGACGAAGAGGCTATTTCAAGGCGATACCAACAATTTCTAAATGCATGGGCGGAGAAAGCTTCCGCGCGCAGTCGGTCGGCCACATGGGCTTTGTTCAGCAACTTCCTAGCATCTCGAAGGAACGGGTCTGTGTCTATTGAAAATGCACAACCAAAGGATGTGGTAGAGTTCCTGTGCTGGTTAGACTCCTGCGGTTCAAGACGGCGTACAATCGTGCATGCTAAACACTGCGAAGCCGTGGGCACCAAAGATCTTACAGCTTGTTCAACAGACAAGGGAGAATGTAGCCTTAGATACGCCTTTGACTCTCTCCGAACAAATCATGTCTCCAAGTTATCCATGGTGTTCGAGAAGGAAATGGGAGTGGTGACACCGTGGAGCAAGACCATGCGGGTTGGTAACCCGGTGAAAAGCGAGCTAGTTGCGCAATATATGGCGTTCACCACAAGTGAGCAAAAAAAGCAGGCGGGAGTATTGGTGAAACAAGCACCGGTCATTCTTCGAAGCCATCTGGAGAAGATTATTTTTCCAATGCAGATCAGGCTTCAATACGCCTCATCCGACGTCGAACGGGTTACGTTGGCTAGGGACATTGCGTTCTTTTCGGTGGCTTTTAGCACAACCAAGAGAGGAGTGGAGCTCACAAATATCCTCATCCAACGTATTTTGCGACTCCCAAATCGAAGCGGCCTCATGTGCAATTTTCAGTGGGGAAAAACCCAACGGGATGGAGCGGATCATATCCTGACCGTACCGTATGATGAGGAATACGTGGCAATCTGCCCAGTTCGAGCGGTAGAGAGGTTTATAGCCGTGGGGAAGCAGGTCGGATGGGACACTACCTCAGGTTACCTTTTCCCAGACATTTCGGAGTCTATGCAAGGTGAAGCGCAGAGAGGCAAGTTGCCGGTGGCTACTAGCAGGATGTCATGTCTGAAGCACTAAAGAGATACGCCGCGGCAGTAGGAGAGACCCAAGGGTTCTCTCTACACTCCTTTAGGTCAGGAGGGGCGGTTTCCAGAGCCCTCGCAGGAGATTCTCTATCGACAATCATGCAGAAGGCATATTGGAAAAGTCCAAAGACTGCTTGGCGATACATGCGACTCATGGAAGTAGTGGCTCCAGGATCCGAGGGTACCGCCATGGTCGAGGGAGTTTCAGAGGAACAATACAGGCAGTTGAATGAGTTCGGTTTGAGTGAACAGAGCCGATCGTTGTCAGCGTTTAGCAATAAGCCTTTGCTTTAGGTGGTCTCGTTTGTTTATTTAAATGGTCTTATTTCAGCCATGGTCTCTCCTCAATGCCTCCACATTCATCTGTTTTCTATCAGTAAAGTATGGGATGAGAAAACTCATCTTGTTCGAGTGAGTTTCCTAAGTAAGGGAGAGAAGTGGATACACCGACCGCGGGAGGTGATGCCACTGTTTACATCCCATACTATCTGAGGAGAAGAACTGACTCGAAAATACTGATGGATTCTCAAAACAGGAAATGAAGAAAGAGGCACGATTTAAGAATTCTCCAGACGTAAAGATCCAGAACAAAGGAGGAGGAAGAACACACTCTCACTTCCTTTTGTCAAACCTGTGCAAGACAGGAAAACAGAGGAAAGGAGAGTCTCCTTTTTGAGAC               Frame 1                         G--F--S--K--Q--E--M--K--K--E--A--R--F--K--N--S--P--D--V--K--I--Q--N--K--G--G--G--R--T--H--S--H--F--L--L--S--N--L--C--K--T--G--K--Q--R--K--G--E--S--P--F--*--D--A--H--H--P--S--A--P--S--A--L--L--E--R--R--P--R--P--S--V--P--L--G--L--V--P--F--P--C--L--C--F--S--S--S--R--F--I--M--A--T--V--P--S--F--Q--L--D--A--L--H--A--P--G--P--E--A--V--I--E--Q--L--Y--D--L--C--A--P--K--S--G--N--G--G--E--R--V--M--D--L--T--R--L--G--V--L--P--H--G--R--Q--L--E--T--A--S--L--D--D--L--C--R--I--F--G--A--L--V--D--A--G--S--A--S--D--K--P--G--M--M--A--V--G--L--P--A--R--G--N--A--D--S--V--A--V--G--D--A--R--M--K--A--A--L--A--Q--A--L--L--Q--Y--L--W--R--C--D--H--H--G--G--L--R--L--S--A--T--A--V--L--L--P--A--P--P--T--L--A--T--A--P--S--A--A--E--G--A--P--S--S--A--P--S--A--R--A--G--S--T--P--S--S--P--S--S--A--S--T--S--A--A--A--A--A--A--A--S--P--S--S--P--A--T--T--A--G--E--A--T--A--G--K--R--L--R--S--E--I--S--G--A--V--A--D--L--E--E--E--S--V--A--G--D--R--E--P--R--V--K--K--K--S--L--V--Y--T--T--I--S--S--K--V--S--L--V--D--I--I--W--F--L--L--V--F--G--R--P--T--S--H--S--S--R--R--L--S--P--S--L--M--D--F--C--T--A--A--F--V--R--R--A--L--S--Q--H--S--L--P--S--F--S--S--T--Q--A--I--A--G--C--P--W--V--S--S--R--G--A--Y--N--M--S--G--F--S--F--R--E--R--P--L--D--I--Y--L--F--V--S--T--S--S--L--F--L--L--L--T--A--P--A--S--C--R--C--A--L--A--*--E--I--L--L--C--S--R--T--*--G--R--G--S--P--S--S--F--C--L--Y--G--R--W--*--V--S--M--V--G--V--F--L--L--G--Y--P--*--A--A--S--H--A--R--I--C--K--I--A--C--S--S--R--T--C--T--R--H--F--Y--N--L--L--E--T--Y--T--H--N--I--Y--W--V--R--A--C--R--L--R--A--L--A--P--F--R--F--S--S--P--L--L--Y--I--T--F--H--F--R--L--*--G--T--L--S--V--A--L--S--V--A--S--L--S--V--N--M--S--T--L--A--E--Q--Y--V--N--I--*--Y--I--I--I--Y--V--Y--L--Y--I--*--Y--F--Y--A--Y--Y--C--I--L--I--Y--I--H--I--V--L--Q--S--L--Y--I--S--C--S--S--S--S--R--H--L--S--L--S--I--N--I--L--A--T--P--F--S--F--L--F--S--P--A--I--H--H--V--S--F--S--F--V--R--D--S--F--G--C--S--L--R--G--I--S--F--C--Q--H--V--D--F--G--R--T--I--C--K--Y--I--I--Y--Y--Y--A--C--I--F--I--Y--F--I--F--L--C--I--L--L--Y--I--D--I--Y--T--Y--C--S--S--K--P--I--Y--I--L--F--F--K--K--P--T--S--L--S--L--Y--K--Y--I--S--P--C--A--I--R--S--E--A--V--S--G--T--A--S--R--H--L--L--G--P--Q--G--E--E--S--G--M--Y--H--F--V--L--G--S--A--T--G--A--A--W--Q--P--S--C--L--E--H--V--S--T--V--V--G--P--A--F--S--F--P--H--R--F--H--L--D--L--V--V--S--A--R--L--F--L--R--V--S--P--L--S--Q--L--P--I--S--H--G--F--F--S--L--S--P--F--F--G--S--V--F--S--S--V--S--P--L--Q--F--G--F--F--S--S--D--S--P--L--S--Q--L--P--I--S--R--V--F--S--L--S--H--R--F--S--A--R--F--S--Q--A--F--L--R--Y--L--S--Y--P--C--L--R--V--F--S--F--L--H--R--F--S--T--R--F--L--K--R--F--S--A--I--S--A--T--H--L--S--G--F--F--S--L--S--P--F--F--S--S--V--F--S--S--V--S--P--L--S--Q--L--P--V--S--Q--G--F--F--F--P--S--P--F--F--S--S--V--S--Q--A--I--L--R--Y--L--R--C--P--S--L--G--F--F--L--P--L--T--V--F--S--A--I--S--A--T--H--F--A--G--F--V--F--L--S--P--F--F--S--S--V--F--S--R--V--S--L--L--S--Q--L--P--S--S--R--V--F--F--F--L--S--P--F--L--N--S--V--S--L--K--R--F--S--A--I--S--A--T--L--S--Q--F--F--F--L--S--P--Y--F--N--S--V--S--L--Q--S--P--L--F--H--I--F--H--S--V--S--R--A--F--S--P--L--F--S--D--V--S--P--L--S--P--V--F--S--S--V--S--F--L--E--R--F--L--R--Y--L--K--C--F--K--W--I--F--S--Q--P--F--L--R--C--V--S--Y--P--S--L--R--V--V--S--F--T--V--F--S--S--V--L--S--G--V--F--L--R--V--S--G--S--D--V--L--Y--P--S--L--R--V--S--L--L--A--V--S--F--H--S--V--F--L--K--R--F--S--A--I--S--P--F--P--R--L--S--F--F--A--I--L--P--F--*--F--A--A--L--W--T--G--L--L--T--S--V--S--G--L--T--T--F--D--A--S--V--K--H--Q--R--V--L--E--I--K--T--K--V--Q--E--L--A--R--L--A--A--M--D--P--E--L--T--D--E--R--T--F--V--D--T--L--A--V--G--I--D--A--A--M--Q--C--G--R--P--D--A--A--A--V--W--V--H--D--K--L--M--S--Q--T--E--E--G--R--K--T--D--R--G--Q--V--V--K--E--L--S--A--I--T--K--F--L--G--G--P--K--H--S--S--A--P--A--G--P--W--S--P--P--P--M--Q--P--M--A--L--T--P--H--F--G--G--Y--S--P--Y--G--M--Q--P--Q--G--G--H--Y--T--P--S--R--G--P--G--G--R--H--R--A--S--G--G--R--S--S--R--G--G--G--S--G--F--S--R--Q--T--A--L--C--N--T--C--R--R--A--G--K--T--G--A--D--I--E--H--S--F--R--V--C--P--F--V--Q--C--H--K--C--H--G--R--G--H--V--I--Q--N--C--P--N--*--I--V--S--D--S--L--S--G--T--R--F--S--S--V--G--F--P--V--L--V--V--R--G--A--S--G--C--S--A--T--R--D--M--F--L--S--F--L--P--S--F--R--V--Q--W--V--F--G--R--F--S--V--F--R--L--G--V--S--F--I--G--E--S--S--L--S--I--G--S--I--H--L--S--F--F--Q--L--E--V--H--K--G--F--L--S--P--I--*--*--P--F--L--F--E--C--T--L--P--P--T--H--H--A--R--R--R--T--R--R--P--T--T--Y--F--R--S--D--G--H--H--V--D--S--P--I--S--P--S--*--L--I--Y--S--V--T--I--L--T--F--R--H--V--I--P--H--Y--L--L--K--L--L--V--I--Y--I--F--L--E--H--S--P--T--P--L--H--L--S--L--S--W--F--G--L--W--S--A--V--D--A--R--I--D--S--C--R--F--T--L--K--S--S--L--N--L--G--E--R--N--F--H--F--A--C--Y--S--P--L--A--Y--A--T--*--V--T--H--V--Q--P--T--T--Y--V--G--Y--G--K--T--S--P--V--F--R--H--Q--R--F--S--S--T--H--F--M--R--V--T--G--R--I--G--S--S--L--V--P--R--R--E--S--R--T--Y--R--T--H--S--H--T--V--S--G--R--G--P--D--Q--C--I--S--E--T--V--N--R--R--P--L--*--Y--S--A--P--S--T--I--S--L--P--T--R--P--S--*--D--V--G--D--G--R--F--R--S--G--S--L--A--N--S--D--*--V--L--A--G--R--N--F--T--G--Y--S--R--W--R--S--Q--Y--P--L--G--V--G--Q--R--G--Q--R--L--R--L--R--R--H--K--G--N--R--H--F--S--R--C--F--V--Q--R--R--*--P--Y--V--N--T--S--A--E--P--R--S--R--R--T--C--I--M--G--H--E--R--S--R--*--A--R--S--D--R--L--R--C--E--M--E--G--R--R--R--R--*--Q--T--A--N--G--A--P--V--G--C--G--A--H--E--T--Q--V--D--M--G--R--T--V--A--*--S--H--V--P--P--P--P--F--Q--H--G--W--R--R--Q--G--S--S--V--R--M--A--R--S--S--S--S--H--Y--*--S--*--G--W--I--P--P--R--C--P--R--*--G--F--L--A--I--F--W--L--R--V--G--R--R--A--V--R--V--Y--R--A--C--F--R--V--V--F--S--P--V--H--I--R--F--S--F--R--G--S--R--S--I--P--A--S--K--R--H--S--R--A--N--M--D--R--R--F--L--F--D--Q--F--S--I--H--P--H--A--P--L--*--R--A--A--Q--S--S--S--D--D--R--V--C--R--T--R--S--S--L--S--R--G--I--L--H--F--A--E--E--V--*--A--H--P--Y--D--E--L--G--F--P--G--D--Y--L--*--L--R--*--Q--A--F--R--G--P--R--G--Q--T--R--Q--A--R--G--D--S--S--R--R--Y--C--V--G--V--H--H--L--S--D--V--R--E--A--R--R--Q--V--H--*--S--I--G--S--G--S--G--G--G--S--L--H--A--P--Y--V--Q--E--H--R--S--F--P--A--T--R--R--S--Q--A--*--H--D--D--T--S--P--K--E--Q--R--S--Y--V--R--V--E--T--L--V--G--S--S--*--A--F--Q--R--G--V--V--V--P--C--R--A--*--A--I--S--S--Y--R--G--I--G--C--L--L--G--R--M--G--G--T--H--*--E--S--R--P--T--H--F--Q--S--R--R--R--F--P--A--T--R--R--A--A--T--Y--Q--R--A--G--R--V--R--S--P--A--D--S--A--S--V--F--G--*--P--T--F--S--V--G--R--V--H--P--H--F--Q--G--G--*--Q--S--L--T--R--R--L--*--E--R--A--V--F--Q--H--P--H--A--*--D--Y--Y--*--S--V--L--V--A--S--A--T--R--F--H--A--E--I--E--V--G--E--L--R--G--K--R--*--G--*--W--Y--F--*--T--R--I--G--R--F--C--E--T--R--*--A--D--V--R--R--P--M--R--V--G--G--*--A--G--D--H--G--F--N--G--H--T--R--L--G--S--Q--A--L--G--R--W--A--L--H--*--*--G--S--A--I--L--L--Q--I--P--Y--T--R--V--C--R--G--*--R--A--Y--A--K--C--S--V--H--A--W--F--N--D--G--G--V--F--R--F--L--L--P--S--D--E--H--G--R--S--V--S--S--A--L--G--G--V--S--R--K--G--A--C--D--R--P--R--S--E--A--V--L--V--S--T--D--S--G--R--G--Y--A--I--T--D--V--V--D--R--*--R--G--*--V--T--L--F--P--G--T--P--P--E--R--V--R--A--F--S--F--Q--T--V--R--N--A--S--C--G--S--G--F--R--P--E--C--*--V--M--K--S--S--F--G--S--F--S--T--C--L--L--F--M--F--G--G--L--W--D--N--A--F--F--T--R--L--S--R--L--C--S--Q--P--L--A--S--S--*--A--S--*--R--R--*--F--P--S--E--I--V--S--A--R--R--S--F--L--L--R--R--S--*--R--C--Q--H--R--G--H--G--S--Y--A--R--S--A--T--V--K--T--M--I--R--F--G--S--V--N--G--A--G--T--Q--*--P--S--T--N--L--G--Q--H--L--L--F--K--W--T--K--R--L--F--Q--G--D--T--N--N--F--*--M--H--G--R--R--K--L--P--R--A--V--G--R--P--H--G--L--C--S--A--T--S--*--H--L--E--G--T--G--L--C--L--L--K--M--H--N--Q--R--M--W--*--S--S--C--A--G--*--T--P--A--V--Q--D--G--V--Q--S--C--M--L--N--T--A--K--P--W--A--P--K--I--L--Q--L--V--Q--Q--T--R--E--N--V--A--L--D--T--P--L--T--L--S--E--Q--I--M--S--P--S--Y--P--W--C--S--R--R--K--W--E--W--*--H--R--G--A--R--P--C--G--L--V--T--R--*--K--A--S--*--L--R--N--I--W--R--S--P--Q--V--S--K--K--S--R--R--E--Y--W--*--N--K--H--R--S--F--F--E--A--I--W--R--R--L--F--F--Q--C--R--S--G--F--N--T--P--H--P--T--S--N--G--L--R--W--L--G--T--L--R--S--F--R--W--L--L--A--Q--P--R--E--E--W--S--S--Q--I--S--S--S--N--V--F--C--D--S--Q--I--E--A--A--S--C--A--I--F--S--G--E--K--P--N--G--M--E--R--I--I--S--*--P--Y--R--M--M--R--N--T--W--Q--S--A--Q--F--E--R--*--R--G--L--*--P--W--G--S--R--S--D--G--T--L--P--Q--V--T--F--S--Q--T--F--R--S--L--C--K--V--K--R--R--E--A--S--C--R--W--L--L--A--G--C--H--V--*--S--T--K--E--I--R--R--G--S--R--R--D--P--R--V--L--S--T--L--L--*--V--R--R--G--G--F--Q--S--P--R--R--R--F--S--I--D--N--H--A--E--G--I--L--E--K--S--K--D--C--L--A--I--H--A--T--H--G--S--S--G--S--R--I--R--G--Y--R--H--G--R--G--S--F--R--G--T--I--Q--A--V--E--*--V--R--F--E--*--T--E--P--I--V--V--S--V--*--Q--*--A--F--A--L--G--G--L--V--C--L--F--K--W--S--Y--F--S--H--G--L--S--S--M--P--P--H--S--S--V--F--Y--Q--*--S--M--G--*--E--N--S--S--C--S--S--E--F--P--K--*--G--R--E--V--D--T--P--T--A--G--G--D--A--T--V--Y--I--P--Y--Y--L--R--R--R--T--D--S--K--I--L--M--D--S--Q--N--R--K--*--R--K--R--H--D--L--R--I--L--Q--T--*--R--S--R--T--K--E--E--E--E--H--T--L--T--S--F--C--Q--T--C--A--R--Q--E--N--R--G--K--E--S--L--L--F--E--T               Frame 2                          D--S--Q--N--R--K--*--R--K--R--H--D--L--R--I--L--Q--T--*--R--S--R--T--K--E--E--E--E--H--T--L--T--S--F--C--Q--T--C--A--R--Q--E--N--R--G--K--E--S--L--L--F--E--T--L--I--I--R--P--R--P--P--L--S--S--S--G--D--R--D--R--P--S--L--W--D--*--F--L--S--P--V--C--V--S--L--L--P--G--S--S--W--Q--R--C--R--V--S--S--*--M--P--S--T--R--Q--D--P--R--Q--S--S--S--S--Y--T--T--S--A--R--R--R--V--A--T--A--V--S--G--S--W--T--*--P--A--W--A--C--Y--R--T--D--G--S--W--K--Q--P--P--*--T--T--C--A--G--S--S--A--L--W--S--T--P--A--Q--P--A--T--S--L--A--*--W--R--S--A--F--P--R--V--G--M--P--T--P--W--P--W--A--T--P--A--*--R--R--P--*--P--R--P--S--S--S--T--C--G--A--A--T--T--T--A--V--S--G--Y--R--R--P--R--F--F--S--R--L--L--P--P--W--R--P--H--L--Q--Q--Q--K--E--H--P--L--P--P--L--Q--Q--G--P--A--A--H--P--L--P--L--P--L--H--P--R--R--L--R--R--Q--R--L--P--R--R--L--P--P--P--L--P--R--A--R--L--P--R--A--S--A--S--G--R--R--S--L--E--P--W--R--T--W--R--R--R--V--W--P--G--I--A--S--L--E--*--R--R--S--R--S--C--T--P--Q--F--P--A--R--*--V--L--*--T--L--Y--G--S--C--S--F--S--A--G--P--R--P--T--A--V--V--V--S--P--R--P--S--W--I--S--A--L--Q--H--S--F--V--V--L--S--L--S--I--P--C--R--H--F--L--R--R--R--P--*--L--A--A--R--G--F--H--H--V--V--L--T--I--*--V--D--F--P--F--G--N--A--R--S--T--Y--I--C--S--F--Q--H--P--L--F--F--F--S--S--R--R--P--P--L--A--G--V--L--L--L--R--R--Y--C--C--V--A--V--R--R--D--V--G--R--H--L--V--S--V--S--M--V--G--G--R--C--L--W--*--V--S--F--F--W--A--I--R--E--R--R--H--T--L--G--F--V--K--L--L--V--R--R--A--L--A--P--V--I--S--T--T--Y--L--K--L--T--P--T--T--S--I--G--*--G--H--A--A--S--A--R--*--P--L--F--V--S--L--L--P--C--Y--T--S--R--F--I--F--V--C--E--G--L--F--R--L--L--S--P--W--H--L--F--L--S--T--C--R--L--W--Q--N--N--M--*--I--Y--D--I--L--L--Y--M--Y--I--Y--I--F--N--I--S--M--H--I--I--V--Y--*--Y--I--Y--I--L--F--F--K--A--Y--I--Y--L--V--L--Q--V--A--D--I--S--L--S--L--*--I--Y--W--L--P--P--F--R--F--S--S--P--L--L--Y--I--T--F--H--F--R--L--*--G--T--L--S--V--A--L--S--V--A--S--L--S--V--N--M--S--T--L--A--E--Q--Y--V--N--I--*--Y--I--I--M--H--V--Y--L--Y--I--L--Y--F--Y--A--Y--Y--Y--I--L--I--Y--I--H--I--V--L--Q--S--L--Y--I--S--C--S--S--R--S--R--H--L--S--L--S--I--N--I--F--L--L--V--R--S--V--R--R--Q--F--Q--V--P--P--L--A--T--C--W--A--R--R--A--K--K--A--G--C--T--T--L--F--*--E--A--L--R--A--P--H--G--S--H--P--V--W--N--M--F--P--L--S--*--V--R--R--F--L--S--L--T--V--F--T--S--I--W--L--S--R--P--V--F--S--Y--A--F--L--R--Y--L--S--Y--P--S--L--M--V--F--S--H--S--H--R--F--S--A--R--F--S--Q--A--F--L--R--F--S--S--V--F--S--Q--A--I--L--R--Y--L--S--Y--P--S--L--G--F--F--L--S--L--T--V--F--Q--L--G--F--L--K--R--F--S--A--I--S--A--T--H--V--S--G--C--F--L--S--F--T--V--S--Q--L--G--F--S--S--D--S--P--L--S--Q--L--P--I--S--R--V--F--S--P--S--H--R--F--S--A--R--F--S--Q--A--F--L--H--Y--L--S--Y--P--C--L--R--V--F--S--F--L--R--R--F--S--A--R--F--L--K--R--F--S--A--I--S--A--A--H--L--S--G--F--F--S--L--S--P--F--S--P--L--S--Q--L--P--I--S--R--V--L--S--F--F--H--R--F--S--A--R--F--S--H--A--F--L--C--Y--L--S--Y--P--P--L--G--C--F--S--F--F--H--R--F--S--T--R--F--L--S--N--V--S--P--L--S--P--L--P--S--L--N--F--S--F--F--R--R--I--S--T--R--F--L--S--S--L--H--C--F--T--F--S--T--R--F--L--E--R--F--L--R--Y--F--L--T--F--L--R--Y--L--R--F--S--A--R--F--L--F--S--S--V--F--S--A--I--S--N--V--S--S--G--F--F--L--N--R--F--S--V--V--S--A--I--P--L--S--G--L--F--L--S--P--F--S--A--R--F--Y--P--A--F--F--F--A--C--L--V--P--M--C--F--T--R--L--S--G--Y--L--F--S--P--S--L--S--T--R--F--F--S--S--V--S--L--L--S--R--P--F--R--G--F--L--F--S--Q--F--F--P--F--S--S--Q--P--C--G--R--V--C--L--P--V--*--A--G--S--R--L--S--T--P--P--S--S--T--S--G--C--*--R--S--K--P--R--S--R--S--*--L--G--*--R--L--W--T--P--S--*--R--T--N--A--R--L--S--T--R--S--P--W--V--L--M--R--R--C--N--A--A--A--R--M--R--P--R--Y--G--Y--M--T--N--S--C--P--K--P--R--R--V--V--K--Q--I--V--A--R--W--*--K--S--C--R--P--S--P--S--F--W--G--G--P--S--I--P--Q--L--Q--P--D--L--G--A--H--P--P--C--S--P--W--P--L--P--R--I--L--G--D--I--A--P--M--V--C--S--H--R--G--G--T--I--P--R--A--G--D--P--G--G--D--T--G--P--L--A--A--A--V--A--E--G--V--A--A--G--L--A--D--R--P--L--C--A--T--L--A--A--V--P--A--R--P--G--Q--I--S--N--T--R--S--V--S--A--P--L--S--N--A--I--S--A--M--V--G--D--T--*--F--R--I--A--P--T--K--*--L--V--I--R--*--A--V--R--V--S--R--L--L--V--S--R--F--S--S--F--G--G--H--L--D--V--Q--R--H--G--T--C--F--S--R--F--C--Q--V--F--A--F--S--G--F--S--A--V--F--R--F--F--A--S--E--F--P--S--L--V--K--V--R--Y--L--L--V--Q--S--T--F--L--S--F--S--S--R--F--I--R--V--F--S--A--R--Y--D--S--L--S--F--S--N--A--R--S--H--P--R--I--M--R--V--G--G--R--V--A--R--P--R--I--S--G--P--M--G--T--M--W--T--H--Q--F--H--R--P--D--*--Y--T--L--*--R--F--S--H--F--V--T--*--S--H--I--T--S--*--N--Y--W--S--Y--T--S--F--S--S--T--L--P--R--L--C--T--F--P--F--L--G--L--D--F--G--R--R--L--T--R--G--S--I--H--V--D--L--H--*--N--H--P--*--T--*--V--N--E--I--S--T--L--R--A--T--L--P--*--H--M--R--H--K--S--H--T--C--S--Q--Q--H--T--L--V--M--A--K--Q--A--Q--F--S--D--I--R--D--F--L--L--H--I--S--C--V--S--P--V--G--S--G--A--A--L--F--L--A--E--R--A--A--R--T--A--H--I--R--T--Q--L--V--G--G--D--L--I--S--A--S--A--K--Q--S--T--V--D--L--S--N--I--R--R--L--Q--Q--S--H--C--L--P--D--Q--A--R--M--W--V--T--D--D--F--V--P--A--H--L--Q--T--A--T--E--F--W--R--D--E--I--L--Q--D--T--P--V--G--D--R--N--T--L--L--G--W--V--K--G--V--N--V--Y--D--F--V--D--T--K--A--T--G--I--F--H--G--A--S--Y--N--G--A--D--L--T--S--I--H--L--P--N--H--V--P--D--E--H--V--S--W--V--T--S--E--V--A--K--L--A--A--T--G--C--V--A--R--W--K--D--V--A--D--V--S--K--P--Q--M--V--L--P--L--G--V--E--P--T--K--P--R--L--I--W--D--A--R--W--L--N--L--M--C--R--H--H--P--F--S--M--D--G--V--G--K--V--A--Q--C--A--W--P--G--A--H--Q--V--T--I--D--H--K--A--G--Y--H--H--V--A--L--D--K--G--S--W--Q--Y--F--G--F--E--W--E--G--E--L--Y--V--F--T--V--L--A--F--G--W--C--S--A--P--F--I--Y--A--S--L--S--E--A--V--A--R--Y--L--R--A--R--D--I--P--V--L--T--W--I--D--D--F--Y--L--T--N--F--R--S--T--R--T--L--R--Y--D--E--Q--L--K--A--A--Q--T--T--A--Y--V--A--L--E--V--L--Y--R--A--G--Y--F--I--S--L--K--K--C--E--L--I--P--T--T--S--L--V--F--L--G--I--I--C--D--S--D--N--R--R--F--E--V--P--E--D--K--L--A--K--L--E--A--I--L--V--D--A--I--A--S--E--S--I--T--F--Q--M--L--E--K--L--A--G--K--C--T--S--L--S--V--A--V--P--V--A--A--L--Y--T--H--H--M--Y--K--S--I--A--V--F--Q--R--R--G--G--R--K--P--S--M--T--I--P--V--P--K--N--S--G--L--M--F--E--L--K--R--W--L--E--V--R--E--H--F--N--G--A--S--W--Y--R--A--E--H--K--Q--L--A--L--T--G--A--S--D--A--S--S--G--G--W--G--G--L--I--R--S--P--G--Q--P--I--F--K--A--G--G--D--F--P--L--H--V--A--Q--Q--H--I--N--V--Q--E--G--Y--A--L--Q--Q--T--L--R--L--F--S--D--S--Q--P--S--Q--L--A--G--S--T--L--I--S--K--V--D--S--K--V--L--H--D--A--F--K--K--G--R--S--S--N--T--L--M--H--E--I--I--T--D--L--F--W--L--Q--V--R--R--D--F--T--L--K--L--K--W--V--S--S--E--E--N--A--E--A--D--G--I--S--R--P--G--S--D--D--F--V--R--L--D--E--R--M--F--G--D--L--C--A--W--A--G--E--Q--V--T--M--D--L--M--A--T--P--A--S--V--H--K--R--W--V--D--G--R--C--T--S--E--D--L--P--F--Y--S--R--Y--H--T--Q--G--C--A--G--V--D--V--L--T--Q--N--V--R--F--M--P--G--S--T--T--E--E--C--F--G--F--C--F--P--P--T--S--M--V--G--V--F--L--Q--H--L--E--E--C--R--A--K--A--L--V--I--V--P--D--R--K--Q--Y--W--F--P--R--I--A--D--A--A--T--R--S--R--T--L--S--I--G--S--G--G--E--S--P--F--F--R--V--H--H--Q--K--G--S--E--R--F--H--F--K--R--S--G--M--L--A--V--V--V--D--F--A--R--N--A--K--*--*--R--V--R--S--A--R--S--Q--R--V--S--C--S--C--S--V--V--C--G--T--T--H--S--L--P--G--S--P--G--C--V--H--S--P--W--P--H--R--E--H--L--R--G--D--N--S--Q--A--R--S--S--R--R--G--E--A--S--S--S--A--A--R--E--G--V--S--I--A--G--T--G--L--M--P--G--V--P--Q--*--K--R--*--F--V--S--V--L--S--M--V--R--V--L--N--S--P--A--P--T--S--D--N--T--S--S--S--S--G--R--R--G--Y--F--K--A--I--P--T--I--S--K--C--M--G--G--E--S--F--R--A--Q--S--V--G--H--M--G--F--V--Q--Q--L--P--S--I--S--K--E--R--V--C--V--Y--*--K--C--T--T--K--G--C--G--R--V--P--V--L--V--R--L--L--R--F--K--T--A--Y--N--R--A--C--*--T--L--R--S--R--G--H--Q--R--S--Y--S--L--F--N--R--Q--G--R--M--*--P--*--I--R--L--*--L--S--P--N--K--S--C--L--Q--V--I--H--G--V--R--E--G--N--G--S--G--D--T--V--E--Q--D--H--A--G--W--*--P--G--E--K--R--A--S--C--A--I--Y--G--V--H--H--K--*--A--K--K--A--G--G--S--I--G--E--T--S--T--G--H--S--S--K--P--S--G--E--D--Y--F--S--N--A--D--Q--A--S--I--R--L--I--R--R--R--T--G--Y--V--G--*--G--H--C--V--L--F--G--G--F--*--H--N--Q--E--R--S--G--A--H--K--Y--P--H--P--T--Y--F--A--T--P--K--S--K--R--P--H--V--Q--F--S--V--G--K--N--P--T--G--W--S--G--S--Y--P--D--R--T--V--*--*--G--I--R--G--N--L--P--S--S--S--G--R--E--V--Y--S--R--G--E--A--G--R--M--G--H--Y--L--R--L--P--F--P--R--H--F--G--V--Y--A--R--*--S--A--E--R--Q--V--A--G--G--Y--*--Q--D--V--M--S--E--A--L--K--R--Y--A--A--A--V--G--E--T--Q--G--F--S--L--H--S--F--R--S--G--G--A--V--S--R--A--L--A--G--D--S--L--S--T--I--M--Q--K--A--Y--W--K--S--P--K--T--A--W--R--Y--M--R--L--M--E--V--V--A--P--G--S--E--G--T--A--M--V--E--G--V--S--E--E--Q--Y--R--Q--L--N--E--F--G--L--S--E--Q--S--R--S--L--S--A--F--S--N--K--P--L--L--*--V--V--S--F--V--Y--L--N--G--L--I--S--A--M--V--S--P--Q--C--L--H--I--H--L--F--S--I--S--K--V--W--D--E--K--T--H--L--V--R--V--S--F--L--S--K--G--E--K--W--I--H--R--P--R--E--V--M--P--L--F--T--S--H--T--I--*--G--E--E--L--T--R--K--Y--*--W--I--L--K--T--G--N--E--E--R--G--T--I--*--E--F--S--R--R--K--D--P--E--Q--R--R--R--K--N--T--L--S--L--P--F--V--K--P--V--Q--D--R--K--T--E--E--R--R--V--S--F--L--R               Frame 3                         --I--L--K--T--G--N--E--E--R--G--T--I--*--E--F--S--R--R--K--D--P--E--Q--R--R--R--K--N--T--L--S--L--P--F--V--K--P--V--Q--D--R--K--T--E--E--R--R--V--S--F--L--R--R--S--S--S--V--R--A--L--R--S--P--R--A--A--T--A--T--V--R--P--S--G--I--S--S--F--P--L--F--V--F--L--F--F--P--V--H--H--G--N--G--A--E--F--P--A--R--C--P--P--R--A--R--T--R--G--S--H--R--A--V--I--R--P--L--R--A--E--E--W--Q--R--R--*--A--G--H--G--P--D--P--P--G--R--A--T--A--R--T--A--A--G--N--S--L--P--R--R--P--V--P--D--L--R--R--F--G--R--R--R--L--S--Q--R--Q--A--W--H--D--G--G--R--P--S--R--A--W--E--C--R--L--R--G--R--G--R--R--P--H--E--G--G--P--S--P--G--P--P--P--V--P--V--A--L--R--P--P--R--R--S--P--A--I--G--D--R--G--S--S--P--G--S--S--H--L--G--D--R--T--F--S--S--R--R--S--T--L--F--R--P--F--S--K--G--R--Q--H--T--L--F--P--F--L--C--I--H--V--G--C--G--G--S--G--C--L--A--V--F--P--R--H--Y--R--G--R--G--Y--R--G--Q--A--P--P--V--G--D--L--W--S--R--G--G--P--G--G--G--E--C--G--R--G--S--R--A--S--S--E--E--E--V--A--R--V--H--H--N--F--Q--Q--G--E--S--C--R--H--Y--M--V--L--A--R--F--R--P--A--H--V--P--Q--Q--S--S--S--L--P--V--P--H--G--F--L--H--C--S--I--R--S--S--C--S--L--S--A--F--P--A--V--I--F--F--D--A--G--H--S--W--L--P--V--G--F--I--T--W--C--L--Q--Y--E--W--I--F--L--S--G--T--P--A--R--H--I--F--V--R--F--N--I--L--S--F--S--S--P--H--G--A--R--L--L--P--V--C--S--C--L--G--D--T--A--V--*--P--Y--V--G--T--W--V--A--I--*--F--L--S--L--W--*--V--V--G--V--Y--G--R--C--L--S--F--G--L--S--V--S--G--V--T--R--S--D--L--*--N--C--L--F--V--A--H--L--H--P--S--F--L--Q--P--T--*--N--L--H--P--Q--H--L--L--G--E--G--M--P--P--P--R--A--S--P--F--S--F--L--F--S--P--A--I--H--H--V--S--F--S--F--V--R--D--S--F--G--C--S--L--R--G--I--S--F--C--Q--H--V--D--F--G--R--T--I--C--K--Y--M--I--Y--Y--Y--I--C--I--F--I--Y--L--I--F--L--C--I--L--L--Y--I--D--I--Y--T--Y--C--S--S--K--P--I--Y--I--L--F--F--K--*--P--T--S--L--S--L--Y--K--Y--I--G--Y--P--L--F--V--S--L--L--P--C--Y--T--S--R--F--I--F--V--C--E--G--L--F--R--L--L--S--P--W--H--L--F--L--S--T--C--R--L--W--Q--N--N--M--*--I--Y--N--I--L--L--C--M--Y--I--Y--I--F--Y--I--S--M--H--I--I--I--Y--*--Y--I--Y--I--L--F--F--K--A--Y--I--Y--L--V--L--Q--E--A--D--I--S--L--S--L--*--I--Y--F--S--L--C--D--P--F--G--G--S--F--R--Y--R--L--S--P--P--A--G--P--A--G--R--R--K--R--D--V--P--L--C--F--R--K--R--Y--G--R--R--M--A--A--I--L--F--G--T--C--F--H--C--R--R--S--G--V--F--F--P--S--P--F--S--P--R--F--G--C--L--G--P--S--F--P--T--R--F--S--A--I--S--A--T--H--L--S--W--F--F--L--T--L--T--V--F--R--L--G--F--L--K--R--F--S--A--S--V--R--F--F--L--K--R--F--S--A--I--S--A--T--H--L--S--G--F--F--S--L--S--P--F--F--S--S--V--F--S--S--V--S--P--L--S--Q--L--P--M--S--Q--G--V--F--F--P--S--P--F--L--N--S--V--S--Q--A--I--L--R--Y--L--S--Y--P--S--L--G--F--F--L--P--L--T--V--F--Q--L--G--F--L--K--R--F--S--T--I--S--A--T--R--V--S--G--F--F--L--S--F--A--V--F--Q--L--G--F--S--S--D--S--P--L--S--P--L--P--I--S--R--V--F--S--P--S--H--R--F--L--R--Y--L--S--Y--P--F--R--G--F--C--L--S--F--T--V--F--Q--L--G--F--L--T--R--F--S--A--I--S--A--T--L--L--S--G--V--F--L--S--F--T--V--S--Q--L--G--F--S--Q--T--F--L--R--Y--L--R--Y--P--L--S--I--F--L--S--F--A--V--F--Q--L--G--F--S--P--V--S--T--V--S--H--F--P--L--G--F--S--S--V--F--S--A--I--F--*--R--F--S--A--I--S--G--F--Q--L--G--F--F--S--R--A--F--S--P--L--S--Q--M--F--Q--V--D--F--F--S--T--V--S--P--L--C--Q--L--S--L--S--Q--G--C--F--F--H--R--F--Q--L--G--F--I--R--R--F--S--S--R--V--W--F--R--C--A--L--P--V--S--P--G--I--S--S--R--R--L--F--P--L--G--F--S--Q--A--F--L--C--Y--L--A--L--S--A--A--F--F--F--R--N--S--S--L--L--V--R--S--P--V--D--G--F--A--Y--Q--C--K--R--A--H--D--F--R--R--L--R--Q--A--P--A--G--A--R--D--Q--N--Q--G--P--G--A--S--*--A--S--G--Y--G--P--R--A--D--G--R--T--H--V--C--R--H--A--R--R--G--Y--*--C--G--D--A--M--R--P--P--G--C--G--R--G--M--G--T--*--Q--T--H--V--P--N--R--G--G--S--*--N--R--S--W--P--G--G--K--R--A--V--G--H--H--Q--V--S--G--G--A--Q--A--F--L--S--S--S--R--T--L--E--P--T--P--H--A--A--H--G--P--Y--P--A--F--W--G--I--*--P--L--W--Y--A--A--T--G--G--A--L--Y--P--E--P--G--T--R--G--A--T--Q--G--L--W--R--P--Q--*--P--R--G--W--Q--R--V--*--Q--T--D--R--F--V--Q--H--L--P--P--C--R--Q--D--R--G--R--Y--R--T--L--V--P--C--L--P--L--C--P--M--P--*--V--P--W--S--G--T--R--N--S--E--L--P--Q--L--N--S--*--*--F--A--E--R--Y--A--F--L--V--C--W--F--P--G--S--R--R--S--G--G--I--W--M--F--S--D--T--G--H--V--S--L--V--S--A--K--F--S--R--S--V--G--F--R--P--F--F--G--F--S--P--R--S--F--L--H--W--*--K--F--V--I--Y--W--F--N--P--P--F--F--L--S--A--R--G--S--*--G--F--S--Q--P--D--M--I--A--F--P--F--R--M--H--A--P--T--H--A--S--C--A--S--E--D--A--S--P--D--H--V--F--P--V--R--W--A--P--C--G--L--T--N--F--T--V--L--T--D--I--L--C--N--D--S--H--I--S--S--R--D--P--T--L--P--P--E--I--T--G--H--I--H--L--S--R--A--L--S--H--A--S--A--P--F--P--F--L--V--W--T--L--V--G--G--*--R--A--D--R--F--M--*--I--Y--T--E--I--I--L--K--P--R--*--T--K--F--P--L--C--V--L--L--S--P--S--I--C--D--I--S--H--T--R--A--A--N--N--I--R--W--L--W--Q--N--K--P--S--F--P--T--S--E--I--F--F--Y--T--F--H--A--C--H--R--S--D--R--E--Q--P--C--S--S--P--R--E--P--H--V--P--H--T--F--A--H--S--*--W--A--G--T--*--S--V--H--Q--R--N--S--Q--P--S--T--S--L--I--F--G--A--F--N--N--L--T--A--Y--Q--T--K--L--G--C--G--*--R--T--I--S--F--R--L--T--C--K--Q--R--L--S--S--G--G--T--K--F--Y--R--I--L--P--L--E--I--A--I--P--S--W--G--G--S--K--G--S--T--F--T--T--S--*--T--Q--R--Q--Q--A--F--F--T--V--L--R--T--T--A--L--T--L--R--Q--Y--I--C--R--T--T--F--P--T--N--M--Y--H--G--S--R--A--K--S--L--S--S--Q--R--Q--V--A--L--R--D--G--R--T--S--P--T--L--A--N--R--K--W--C--S--R--W--V--W--S--P--R--N--P--G--*--Y--G--T--H--G--G--L--I--S--C--A--A--T--T--L--S--A--W--M--A--*--A--R--*--L--S--A--H--G--P--E--L--I--K--S--L--L--I--I--R--L--D--T--T--T--L--P--*--I--R--V--L--G--N--I--L--A--S--S--G--K--A--S--C--T--C--L--P--C--L--L--S--G--G--V--Q--P--R--S--Y--T--L--L--F--Q--R--Q--S--L--D--T--C--E--Q--E--T--F--P--C--*--H--G--*--T--I--S--I--*--P--I--F--D--P--P--A--R--S--A--M--T--S--S--S--K--Q--L--R--R--P--R--M--S--H--*--K--F--S--I--A--R--D--T--S--F--R--*--R--S--V--S--S--S--L--R--R--A--W--F--S--W--G--L--S--V--T--P--I--T--G--V--S--R--S--Q--R--T--N--S--P--S--S--R--R--F--*--S--T--L--L--R--R--S--P--S--P--F--R--C--*--R--S--S--Q--A--S--A--L--V--Y--R--*--R--F--R--W--R--L--F--T--R--T--I--C--T--R--A--S--Q--F--S--S--D--A--A--V--A--S--L--A--*--R--Y--Q--S--Q--R--T--A--V--L--C--S--S--*--N--A--G--W--K--F--V--S--I--S--T--G--R--R--G--T--V--P--S--I--S--N--*--L--L--P--G--H--R--M--P--P--R--E--D--G--G--D--S--L--G--V--Q--A--N--P--F--S--K--Q--A--E--I--S--R--Y--T--S--R--S--N--I--S--T--C--R--K--G--T--L--S--S--R--L--C--V--C--F--R--I--A--N--L--L--S--W--P--G--P--P--S--F--P--R--W--I--A--K--S--Y--T--T--P--L--R--K--G--G--L--P--T--P--S--C--M--R--L--L--L--I--C--S--G--C--K--C--D--A--I--S--R--*--N--*--S--G--*--A--P--R--K--T--L--R--L--M--V--F--L--D--Q--D--R--T--I--L--*--D--*--M--S--G--C--S--A--T--Y--A--R--G--R--V--S--R--*--P--W--I--*--W--P--H--P--P--R--F--T--S--A--G--*--M--G--A--A--L--V--R--I--C--H--F--T--P--D--T--I--H--K--G--V--P--G--L--T--C--L--R--K--M--F--G--S--C--L--V--Q--R--R--R--S--V--S--V--S--A--S--L--R--R--A--W--*--E--C--F--F--S--T--W--R--S--V--A--Q--R--R--L--*--S--S--Q--I--G--S--S--T--G--F--H--G--*--R--T--R--L--R--D--H--G--R--C--R--S--V--A--G--V--S--H--P--F--S--G--Y--T--T--R--K--G--P--S--V--F--I--S--N--G--Q--E--C--*--L--W--*--W--I--S--P--G--M--L--S--D--E--E--F--V--R--L--V--L--N--V--S--P--V--H--V--R--W--F--V--G--Q--R--I--L--Y--Q--A--L--Q--A--V--F--T--A--L--G--L--I--V--S--I--L--E--A--I--I--P--K--R--D--R--L--G--E--A--K--L--P--P--P--P--L--V--K--V--S--A--S--R--A--R--V--L--C--P--E--C--H--S--E--N--D--D--S--F--R--F--C--Q--W--C--G--Y--S--I--A--Q--H--Q--P--R--T--T--P--P--L--Q--V--D--E--E--A--I--S--R--R--Y--Q--Q--F--L--N--A--W--A--E--K--A--S--A--R--S--R--S--A--T--W--A--L--F--S--N--F--L--A--S--R--R--N--G--S--V--S--I--E--N--A--Q--P--K--D--V--V--E--F--L--C--W--L--D--S--C--G--S--R--R--R--T--I--V--H--A--K--H--C--E--A--V--G--T--K--D--L--T--A--C--S--T--D--K--G--E--C--S--L--R--Y--A--F--D--S--L--R--T--N--H--V--S--K--L--S--M--V--F--E--K--E--M--G--V--V--T--P--W--S--K--T--M--R--V--G--N--P--V--K--S--E--L--V--A--Q--Y--M--A--F--T--T--S--E--Q--K--K--Q--A--G--V--L--V--K--Q--A--P--V--I--L--R--S--H--L--E--K--I--I--F--P--M--Q--I--R--L--Q--Y--A--S--S--D--V--E--R--V--T--L--A--R--D--I--A--F--F--S--V--A--F--S--T--T--K--R--G--V--E--L--T--N--I--L--I--Q--R--I--L--R--L--P--N--R--S--G--L--M--C--N--F--Q--W--G--K--T--Q--R--D--G--A--D--H--I--L--T--V--P--Y--D--E--E--Y--V--A--I--C--P--V--R--A--V--E--R--F--I--A--V--G--K--Q--V--G--W--D--T--T--S--G--Y--L--F--P--D--I--S--E--S--M--Q--G--E--A--Q--R--G--K--L--P--V--A--T--S--R--M--S--C--L--K--H--*--R--D--T--P--R--Q--*--E--R--P--K--G--S--L--Y--T--P--L--G--Q--E--G--R--F--P--E--P--S--Q--E--I--L--Y--R--Q--S--C--R--R--H--I--G--K--V--Q--R--L--L--G--D--T--C--D--S--W--K--*--W--L--Q--D--P--R--V--P--P--W--S--R--E--F--Q--R--N--N--T--G--S--*--M--S--S--V--*--V--N--R--A--D--R--C--Q--R--L--A--I--S--L--C--F--R--W--S--R--L--F--I--*--M--V--L--F--Q--P--W--S--L--L--N--A--S--T--F--I--C--F--L--S--V--K--Y--G--M--R--K--L--I--L--F--E--*--V--S--*--V--R--E--R--S--G--Y--T--D--R--G--R--*--C--H--C--L--H--P--I--L--S--E--E--K--N--*--L--E--N--T--D--G--F--S--K--Q--E--M--K--K--E--A--R--F--K--N--S--P--D--V--K--I--Q--N--K--G--G--G--R--T--H--S--H--F--L--L--S--N--L--C--K--T--G--K--Q--R--K--G--E--S--P--F--*--D                Note  genome sequences for Ectocarpus siliculosus can be searched at  EBI               ```                Back to Contents                 ---                **- Structure of the ALOG domain-containing DIRS1-like transposons in Branchiostoma floridae and coordinates of other fragmentary elements**                 ```               The Branchiostoma element was reconstructed from this genome sequence               260806091 and corresponds               to the protein 260795011                 Boundaries                                                                                                                                                                                                                                                                                                                                                                                                                                                                                                                                                                                                                                                                                                                                                                                                                                                                                                                                                                                                                                                                                                                                                                   <---------Pre-RT- frame 1 ----------------------------------------------------------------------------------------------------------------------------------------------------------------------------------------------------------------------------------------------------------------------------------------------------------------------------------------------------------------------------------------------------------------------------------------------------------------------------------------------------------------------<-- RT begins here in all in frame 1-----------------------------------------------------------------------------------------------------------------------------------------------------------------------------------------------------------------------------------------------------------------------------------------------------------------------------------------------------------------------------------------------------------------------------------------------------------------------------------------------------------------------------------------------------------------------------------------------------------------------------------------------------------------------------------------------------------------------------------------------------------------------------------------------------------------------------------------------------><-RNaseH in frame 1----------------------------------------------------------------------------------------------------------------------------------------------------------------------------------------------------------------------------------------------------------------------------------------------------------------------------------------------------------------------------------------------------------------------------><---N6A methyltransferase in frame 1--------------------------------------------------------------------------------------------------------------------------------------------------------------------------------------------------------------------------------------------------------------------------------------------------------------------------------------------------------------------------------------------------------------------------------------------------------------------------------------------------------------------------------><-- Zinc Ribbon in frame 3 ------------------------------------------------------------------------------------------------------------------------------<----ALOG in frame 3----------------------------------------------------------------------------------------------------------------------------------------------------------------------------------------------------------------------------------------<  Integrase in frame 1--------------------------------------------------------------------ALOG ends here ------->-----------------------------------------------------------------------------------------------< Beyond this point an integrase domain from a distinct location was spliced to reconstruct the ancestral sequence               260806091  CTGACTTTCTTTCATGATCGTTAGAATTTCCTTTGTTTATGTTATGAATAAAGATGTTCAGTAACATAGGTTGATTTGCTGTTTCCCTTTTTGTTAATTCTTTAATTGCTTCTATTTGCATGAGAAAGGTGGGCGTCTCCAACTTAGTCAATTTATTCGTTGGTTGGTACACCCCTACCCCTAGGGAGACCATTCAGTTGAATGGTGGGCGTCTCCAACTTAGTCAATTTATTCGTTACTTAGTACACCCCTACCCCTAGGGACACCATACTATGTGGATTTAGTCACAAGAAATACTCGCTCCTGATTGGCTGAGGAAAGATGGCCATGTTCTAAGTATCGGTGGCATCTGCAACTGATTGGAGCAAGATGCCGCCGTGCAAATTAATGACGGGATCTTTTCTTTGTATCTAACTGAGAAACTCGGTATGTGGCTAGTCTCTACCAGACTCCGGATCGCTGAAAAAATCGTAGAAGTGGAACAAATAGAGGAGTAAGCCGGCCAGAGGAATATAGCCGGCTAGGCAACAGCACGCGAGCTGTTCCCTGGCCGGTTATATTCCTCTCTATTTGTTCCATTTCTACGATTTTCCAGCGATCCGGAGTCTGGTAGAGACTAGTATGTGGCCCAGACGTGCCAGGCGCTGTCTGTATGCGTCGAGAACCCGTGTCCCCAGACGGTCAAACCACCCGCGCATTGTGGGAAGAAATACGCGGGGTCACACCCTGAAATCCCCCCCCCCCCCCCCTCACCCACGCATCACTCTCACATATAACAAGACTCGAGTCGTGCGTAGGAGATTGTCCTAGATCCATTCCGTGTAGCAGATGATTTCATGTTGTGCAAGCAAGCATTTGTTGTGTCCGTTTTGGCGTAGAGATTATCATTTTTCTATGTATTGATTTTCACCCCACAACCGCTGTCTAGCCCTCGGGAGGTGCTCAGAAAAAGTTGCGGCGGTCAATTGATTATTGTGGCCGGATTGATTGTCTAATCATTCAGGGAGCGAGGTACAGTCTGTGACGTCGGGACCAATCCCCCGATAAGCTTCTCAAGTTTGACTCGGAGTCCCAAGTATCTCGATAGATCCTCAATGTTTCTACGCATGTTTCCTTCCTTAGAACCCAACCATGAAGAAAATTTCTCGCCCCGGGAGGTCACCCCGCCAGGCTTCGTTTGACCCTTACAACATAGCCACGCGTGCCGATGAGGACACCACGTGGGTGACGTATGACGGGCGACCGTGGCCGGAACCCGTCCCGACAACTCCGATACCAGCGGAGGAAGCCATAGCCACCCAGGAGACCATGGATCCGACCACCTTGCCGTTTAGAAACCCAGATTGCTTTATAGCTGGGTCTCTGCACAACAATAAAGAAGTATGGGCACAACTAACACCATTCTCCGACAAGGGAGAAGACGTGTTAAGATGGATCACGAACAAAGTAAACGTCAGTGACTTTTTCACACCTTTTGATGGGTCTTTCAAAGGTAACACATACAAAGCTGCCACACCCCCATCAATCATCCTCCCCAACGCCAGAAACTGTGTAGAGCACCATGACTTTCTAGCCTCTCAGATAAAAGATAGGTTAGCAGACGGCTCCATTAAAGTGTGGGGAAGGGTAGGACATTGTGACCCCCCTCATTTAGTATTGCCCCTTACGGTAGAGCCTTCAAAACCGAGAATGTGCTGGGATGGGCGTTTTCTTAACCTCTGGACGAAACGCTGCCCATTCAATCTAGACGCAATCACAGAGGCCCCAAGGATGCTATACAAAGGAGGTTACATGACCCACACAGATGAAAAAAGTGGTTACTCGCACATCGGCCTGACGGAAGAGAGTTGGACCTACTTTGGCTTTCAATTCGATGACGTTCTGTACGTCTACTCATCCCTGCCATTCGGCTGGTCCGCGTCAGCCTTTATTTTCCACACAACAGGAAGTGTAATCTCGTCGTATGCTAGACATCTAGGGGTACCAACAATGCTGTATATTGATGACCGTTTGAACGGCGAAGCCACAGCCCCTCCGCCCATGACCATAGCACTACCCCTCCACCCATACACGCAAGCACAGAAAGCAAGTTACGTCATGTGTGAGTTGCTCACGAGAGTGGGTTACTACCTCAGGCTGAATAAATGTATCATCATCCCGACACAGATTTTGGTACACTTAGGTGCTGGGTTGAACTCCAAAGATGGCGTCTTCTTCTTCCCGGATGACAAACGTCTAAAATTCATAACACTCAGGGAGGAGATTCTTAGCGCCCCCATGGTATCTCTTCACACCCTGCAGAAATTCGTCGGCAAATGCGTCTCAATGGCGCTTATGGTCCCGGGAGCGCGCATTTACACGAGACAATGTAATAAACTAGTTGGAGAAATGACAAAGAAAGGGAAAATCCGCACCAGGCTTCCAGCCGGCGTTCGCCCTGAAATAGAACATTGGCGCTTTGTAGATAATGACATGAAGCCGGTCCCATGGAGGGACGAGCGACACTCGTCCATCACGAGAGCATCCGATGCGTCAGGTTACGGATGGGGTGCGGTACTGCACGGGGTACCCAGAGTCGGACGTTAAACTGGGGGATTACTGGTCAGAGCAGGATAGAGCTAAGGACATCAATTGTAAAGAAGCGAAAGCAGTAGCCCTCACCCTTAAAGCAGCCAAACAATGGATCGCTATTTCAAGAGTACACATGAAAGTAGACAACAGGGCGGTAGTAGACTCGTGGCAAGGCAGCGGGGCCCGGGCTCTGCCGCTGCGCTGAATGAGGAAATCAAGAACATCTTCCAGACCACATTGGCCCTGAATATATCGCTATCTATGTCACACGTCAAAACAAAAGATAACGAGGCAGATCAACCATCCAGACAGCTGTCCAAAACAGACTGCATGTTGGCACCTCATCTTTGGCAACGTGTGCAATCAGCATTTGGGGGTCAGCGGGGACATTCAGTAGACTTGATGGCGTTGGATTCTAACGTACAGAGAGACAATTCGGGTAACCCGCTAAAGCATTATACCCCTTATCCAACGCCGGAGTCAGCAGGTGTAGACGTATTCAGGCACAATCCCACAACTTCTCCGGGGGGAGAGAGAGAGAATGCGTATGTTTTCCCACCCATCAACATGACAGGGGCAGTCATCAATTTCCTGATACAATGGAAAGCAGACGTGACGGTCCCCAAGTTGTGCCCGAGACCCCTGTGGTGGCCGACACTGTTACGGGTGGCGAAAACAAGCATCAAATTAACAGAAGCAGGAAATAGTTCAGCTCTATGGTTTCCATCCAAGCAAGGTCTGAGACCTGGAAAGGCATTCCAGTACGAACTGTGGGCGTTCAGGATAGAACAAGAATAAGGTTACACCCCGGTACCTTACCATAGAAGAACAGTAGAACATACGTTATCACGATGTCAAAGATTAAACACCACCCTCTCGCCCGCATACAGCCACTACCCCTGATTCCACGACAATGGACGTCATCAGTGGCATGCCCAGAATGCCATCGCCACAACGATCACGACTTCAGGCTGTGTCAAATGTGCGGATACGAAAGGCAACCCTTCCCACCACCAAGGGAAAAGTTGGATGTGGACGACGACAAGATTCAGGCACGGCTAGAAGAAATAAACAAACTAGCCGTAAGCTCAGACTATGGAAAAAAGAAAGCTGCACTTGAAAACGAGTTAAGAAACTTCTTAGGTAATAGGTCTCCCCCCAAGGACCTCACCACAGCCTCGCCAAAAGACGTTTGCAGTTTCTTAGTCTGGAAAGACGAAGGGGGGGGGGGGACTATAGTACATAAGACGGGTTGTAAGAACTTTGGGGAAAAACGAAAGTCGGTGTGTGGCTGCCCGAAGAGACTGGCAGCCGGGACGGTAGACTCCGTTATCGGACAACTACGATCGATTTTTACGAAATCGGGGAGGGGCGGCGAATGGAACGACGCGATATTTGCGGGAAACCCAGCAGCCGCACCTGAGGGTAGGAGAATACCTTAGGATAACAAAGGCAGAGCAGGCTAACGCCTTGATTCAACCTACACAAGCTCAACCGGTCTTCCATGACAAGTTGGAGGCTTTGTGTACGCATATCGCCATGAAATTGAGAGACCCAAAGACTAAGGAAAGTAAGCTTTTCACTTTGGCTCGGGATCAAGCGTTTTTCAAGGCCATGTTCTTCGGAGCGGACAGAGCAGCAGACCTAAGCAGGTGTAAATCAGAAGAACTGGCGTGGCTGCCGGAGGGTGAGGGCATCCTATTCAACCATACTTTTGGCAAGACATTGCGAGACGGAACAGCGAACACGTTCCCAATCCTAGCTAACGAAAACAGCGCCATCTGCCCAGTTCGAGGTTTACAGGCATACTTCACAATGGCCACGGCTCTCGGTATTAACCTCTCGAAAGGTTATCTTTTCCGAGCAATGAATAAATCGAAAGAGGTGATCAACGAGCCCTTTTCTTACGACGCAGCCCAGAGCCGTTTCAAGGAGTATTTGATCGAAATAAACAGGTACGAAGGAGACACCCTTCACGGTCTGCGTACGGCCAGTGCCATCACCATAGCAATGGGGGGCGCGAGCCAGTCAGCCCTCATGGCACACGTGGGATGGCGGGAGAAAGCGACAGCTCAGCGATATATGCAACTCAGGAAGGTGTGCCACGAGGAGTCCCCTGCGGCCATCCTACGCGAACAAGTGACACCGGGAGCCCGAAAAGACAACCCTAATAAGAAACGGATCACAGATGCGGCAACAGTGTATCAGTGTAAGAACAGTACAGATTACAAGCCAGTGCTTTAGTTTAATTTCCCCAAGACGGTATTACAATGTTTAGCAAATAACATGCCCTTTTCTCTAGTCAGCTTACTGGGGTAAGTGTGGTATCTAGCAGAATAGTAATAGGAACATTTGTGAGGTGGCGAATACCCATTGACAATATCTGTATTTGACCAGATGTATACGGCGTGAGAAGAGATCGGACTGTTA               1          M--T--F--F--H--D--R--$--N--F--L--C--L--C--Y--E--$--R--C--S--V--T--$--V--D--L--L--F--P--F--L--L--I--L--$--L--L--L--F--A--$--E--R--W--A--S--P--T--$--S--I--Y--S--L--V--G--T--P--L--P--L--G--R--P--F--S--$--M--V--G--V--S--N--L--V--N--L--F--V--T--$--Y--T--P--T--P--R--D--T--I--L--C--G--F--S--H--K--K--Y--S--L--L--I--G--$--G--K--M--A--M--F--$--V--S--V--A--S--A--T--D--W--S--K--M--P--P--C--K--L--M--T--G--S--F--L--C--I--$--L--R--N--S--V--C--G--$--S--L--P--D--S--G--S--L--K--K--S--$--K--W--N--K--$--R--S--K--P--A--R--G--I--$--P--A--R--Q--Q--H--A--S--C--S--L--A--G--Y--I--P--L--Y--L--F--H--F--Y--D--F--P--A--I--R--S--L--V--E--T--S--M--W--P--R--R--A--R--R--C--L--Y--A--S--R--T--R--V--P--R--R--S--N--H--P--R--I--V--G--R--N--T--R--G--H--T--L--K--S--P--P--P--P--P--H--P--R--I--T--L--T--Y--N--K--T--R--V--V--R--R--R--L--S--$--I--H--S--V--$--Q--M--I--S--C--C--A--S--K--H--L--L--C--P--F--W--R--R--D--Y--H--F--S--M--Y--$--F--S--P--H--N--R--C--L--A--L--G--R--C--S--E--K--V--A--A--V--N--$--L--L--W--P--D--$--L--S--N--H--S--G--S--E--V--Q--S--V--T--S--G--P--I--P--R--$--A--S--Q--V--$--L--G--V--P--S--I--S--I--D--P--Q--C--F--Y--A--C--F--L--P--$--N--P--T--M--K--K--I--S--R--P--G--R--S--P--R--Q--A--S--F--D--P--Y--N--I--A--T--R--A--D--E--D--T--T--W--V--T--Y--D--G--R--P--W--P--E--P--V--P--T--T--P--I--P--A--E--E--A--I--A--T--Q--E--T--M--D--P--T--T--L--P--F--R--N--P--D--C--F--I--A--G--S--L--H--N--N--K--E--V--W--A--Q--L--T--P--F--S--D--K--G--E--D--V--L--R--W--I--T--N--K--V--N--V--S--D--F--F--T--P--F--D--G--S--F--K--G--N--T--Y--K--A--A--T--P--P--S--I--I--L--P--N--A--R--N--C--V--E--H--H--D--F--L--A--S--Q--I--K--D--R--L--A--D--G--S--I--K--V--W--G--R--V--G--H--C--D--P--P--H--L--V--L--P--L--T--V--E--P--S--K--P--R--M--C--W--D--G--R--F--L--N--L--W--T--K--R--C--P--F--N--L--D--A--I--T--E--A--P--R--M--L--Y--K--G--G--Y--M--T--H--T--D--E--K--S--G--Y--S--H--I--G--L--T--E--E--S--W--T--Y--F--G--F--Q--F--D--D--V--L--Y--V--Y--S--S--L--P--F--G--W--S--A--S--A--F--I--F--H--T--T--G--S--V--I--S--S--Y--A--R--H--L--G--V--P--T--M--L--Y--I--D--D--R--L--N--G--E--A--T--A--P--P--P--M--T--I--A--L--P--L--H--P--Y--T--Q--A--Q--K--A--S--Y--V--M--C--E--L--L--T--R--V--G--Y--Y--L--R--L--N--K--C--I--I--I--P--T--Q--I--L--V--H--L--G--A--G--L--N--S--K--D--G--V--F--F--F--P--D--D--K--R--L--K--F--I--T--L--R--E--E--I--L--S--A--P--M--V--S--L--H--T--L--Q--K--F--V--G--K--C--V--S--M--A--L--M--V--P--G--A--R--I--Y--T--R--Q--C--N--K--L--V--G--E--M--T--K--K--G--K--I--R--T--R--L--P--A--G--V--R--P--E--I--E--H--W--R--F--V--D--N--D--M--K--P--V--P--W--R--D--E--R--H--S--S--I--T--R--A--S--D--A--S--G--Y--G--W--G--A--V--L--H--G--V--P--R--V--G--R--$--T--G--G--L--L--V--R--A--G--$--S--$--G--H--Q--L--$--R--S--E--S--S--S--P--H--P--$--S--S--Q--T--M--D--R--Y--F--K--S--T--H--E--S--R--Q--Q--G--G--S--R--L--V--A--R--Q--R--G--P--G--S--A--A--A--L--N--E--E--I--K--N--I--F--Q--T--T--L--A--L--N--I--S--L--S--M--S--H--V--K--T--K--D--N--E--A--D--Q--P--S--R--Q--L--S--K--T--D--C--M--L--A--P--H--L--W--Q--R--V--Q--S--A--F--G--G--Q--R--G--H--S--V--D--L--M--A--L--D--S--N--V--Q--R--D--N--S--G--N--P--L--K--H--Y--T--P--Y--P--T--P--E--S--A--G--V--D--V--F--R--H--N--P--T--T--S--P--G--G--E--R--E--N--A--Y--V--F--P--P--I--N--M--T--G--A--V--I--N--F--L--I--Q--W--K--A--D--V--T--V--P--K--L--C--P--R--P--L--W--W--P--T--L--L--R--V--A--K--T--S--I--K--L--T--E--A--G--N--S--S--A--L--W--F--P--S--K--Q--G--L--R--P--G--K--A--F--Q--Y--E--L--W--A--F--R--I--E--Q--E--$--G--Y--T--P--V--P--Y--H--R--R--T--V--E--H--T--L--S--R--C--Q--R--L--N--T--T--L--S--P--A--Y--S--H--Y--P--$--F--H--D--N--G--R--H--Q--W--H--A--Q--N--A--I--A--T--T--I--T--T--S--G--C--V--K--C--A--D--T--K--G--N--P--S--H--H--Q--G--K--S--W--M--W--T--T--T--R--F--R--H--G--$--K--K--$--T--N--$--P--$--A--Q--T--M--E--K--R--K--L--H--L--K--T--S--$--E--T--S--$--V--I--G--L--P--P--R--T--S--P--Q--P--R--Q--K--T--F--A--V--S--$--S--G--K--T--K--G--G--G--G--L--$--Y--I--R--R--V--V--R--T--L--G--K--N--E--S--R--C--V--A--A--R--R--D--W--Q--P--G--R--$--T--P--L--S--D--N--Y--D--R--F--L--R--N--R--G--G--A--A--N--G--T--T--R--Y--L--R--E--T--Q--Q--P--H--L--R--V--G--E--Y--L--R--I--T--K--A--E--Q--A--N--A--L--I--Q--P--T--Q--A--Q--P--V--F--H--D--K--L--E--A--L----               2          $--L--S--F--M--I--V--R--I--S--F--V--Y--V--M--N--K--D--V--Q--$--H--R--L--I--C--C--F--P--F--C--$--F--F--N--C--F--Y--L--H--E--K--G--G--R--L--Q--L--S--Q--F--I--R--W--L--V--H--P--Y--P--$--G--D--H--S--V--E--W--W--A--S--P--T--$--S--I--Y--S--L--L--S--T--P--L--P--L--G--T--P--Y--Y--V--D--L--V--T--R--N--T--R--S--$--L--A--E--E--R--W--P--C--S--K--Y--R--W--H--L--Q--L--I--G--A--R--C--R--R--A--N--$--$--R--D--L--F--F--V--S--N--$--E--T--R--Y--V--A--S--L--Y--Q--T--P--D--R--$--K--N--R--R--S--G--T--N--R--G--V--S--R--P--E--E--Y--S--R--L--G--N--S--T--R--A--V--P--W--P--V--I--F--L--S--I--C--S--I--S--T--I--F--Q--R--S--G--V--W--$--R--L--V--C--G--P--D--V--P--G--A--V--C--M--R--R--E--P--V--S--P--D--G--Q--T--T--R--A--L--W--E--E--I--R--G--V--T--P--$--N--P--P--P--P--P--L--T--H--A--S--L--S--H--I--T--R--L--E--S--C--V--G--D--C--P--R--S--I--P--C--S--R--$--F--H--V--V--Q--A--S--I--C--C--V--R--F--G--V--E--I--I--I--F--L--C--I--D--F--H--P--T--T--A--V--$--P--S--G--G--A--Q--K--K--L--R--R--S--I--D--Y--C--G--R--I--D--C--L--I--I--Q--G--A--R--Y--S--L--$--R--R--D--Q--S--P--D--K--L--L--K--F--D--S--E--S--Q--V--S--R--$--I--L--N--V--S--T--H--V--S--F--L--R--T--Q--P--$--R--K--F--L--A--P--G--G--H--P--A--R--L--R--L--T--L--T--T--$--P--R--V--P--M--R--T--P--R--G--$--R--M--T--G--D--R--G--R--N--P--S--R--Q--L--R--Y--Q--R--R--K--P--$--P--P--R--R--P--W--I--R--P--P--C--R--L--E--T--Q--I--A--L--$--L--G--L--C--T--T--I--K--K--Y--G--H--N--$--H--H--S--P--T--R--E--K--T--C--$--D--G--S--R--T--K--$--T--S--V--T--F--S--H--L--L--M--G--L--S--K--V--T--H--T--K--L--P--H--P--H--Q--S--S--S--P--T--P--E--T--V--$--S--T--M--T--F--$--P--L--R--$--K--I--G--$--Q--T--A--P--L--K--C--G--E--G--$--D--I--V--T--P--L--I--$--Y--C--P--L--R--$--S--L--Q--N--R--E--C--A--G--M--G--V--F--L--T--S--G--R--N--A--A--H--S--I--$--T--Q--S--Q--R--P--Q--G--C--Y--T--K--E--V--T--$--P--T--Q--M--K--K--V--V--T--R--T--S--A--$--R--K--R--V--G--P--T--L--A--F--N--S--M--T--F--C--T--S--T--H--P--C--H--S--A--G--P--R--Q--P--L--F--S--T--Q--Q--E--V--$--S--R--R--M--L--D--I--$--G--Y--Q--Q--C--C--I--L--M--T--V--$--T--A--K--P--Q--P--L--R--P--$--P--$--H--Y--P--S--T--H--T--R--K--H--R--K--Q--V--T--S--C--V--S--C--S--R--E--W--V--T--T--S--G--$--I--N--V--S--S--S--R--H--R--F--W--Y--T--$--V--L--G--$--T--P--K--M--A--S--S--S--S--R--M--T--N--V--$--N--S--$--H--S--G--R--R--F--L--A--P--P--W--Y--L--F--T--P--C--R--N--S--S--A--N--A--S--Q--W--R--L--W--S--R--E--R--A--F--T--R--D--N--V--I--N--$--L--E--K--$--Q--R--K--G--K--S--A--P--G--F--Q--P--A--F--A--L--K--$--N--I--G--A--L--$--I--M--T--$--S--R--S--H--G--G--T--S--D--T--R--P--S--R--E--H--P--M--R--Q--V--T--D--G--V--R--Y--C--T--G--Y--P--E--S--D--V--K--L--G--D--Y--W--S--E--Q--D--R--A--K--D--I--N--C--K--E--A--K--A--V--A--L--T--L--K--A--A--K--Q--W--I--A--I--S--R--V--H--M--K--V--D--N--R--A--V--V--D--S--W--Q--G--S--G--A--R--A--L--P--L--R--$--M--R--K--S--R--T--S--S--R--P--H--W--P--$--I--Y--R--Y--L--C--H--T--S--K--Q--K--I--T--R--Q--I--N--H--P--D--S--C--P--K--Q--T--A--C--W--H--L--I--F--G--N--V--C--N--Q--H--L--G--V--S--G--D--I--Q--$--T--$--W--R--W--I--L--T--Y--R--E--T--I--R--V--T--R--$--S--I--I--P--L--I--Q--R--R--S--Q--Q--V--$--T--Y--S--G--T--I--P--Q--L--L--R--G--E--R--E--R--M--R--M--F--S--H--P--S--T--$--Q--G--Q--S--S--I--S--$--Y--N--G--K--Q--T--$--R--S--P--S--C--A--R--D--P--C--G--G--R--H--C--Y--G--W--R--K--Q--A--S--N--$--Q--K--Q--E--I--V--Q--L--Y--G--F--H--P--S--K--V--$--D--L--E--R--H--S--S--T--N--C--G--R--S--G--$--N--K--N--K--V--T--P--R--Y--L--T--I--E--E--Q--$--N--I--R--Y--H--D--V--K--D--$--T--P--P--S--R--P--H--T--A--T--T--P--D--S--T--T--M--D--V--I--S--G--M--P--R--M--P--S--P--Q--R--S--R--L--Q--A--V--S--N--V--R--I--R--K--A--T--L--P--T--T--K--G--K--V--G--C--G--R--R--Q--D--S--G--T--A--R--R--N--K--Q--T--S--R--K--L--R--L--W--K--K--E--S--C--T--$--K--R--V--K--K--L--L--R--$--$--V--S--P--Q--G--P--H--H--S--L--A--K--R--R--L--Q--F--L--S--L--E--R--R--R--G--G--G--D--Y--S--T--$--D--G--L--$--E--L--W--G--K--T--K--V--G--V--W--L--P--E--E--T--G--S--R--D--G--R--L--R--Y--R--T--T--T--I--D--F--Y--E--I--G--E--G--R--R--M--E--R--R--D--I--C--G--K--P--S--S--R--T--$--G--$--E--N--T--L--G--$--Q--R--Q--S--R--L--T--P--$--F--N--L--H--K--L--N--R--S--S--M--T--S--W--R--L--X--               3          D--F--L--S--$--S--L--E--F--P--L--F--M--L--$--I--K--M--F--S--N--I--G--$--F--A--V--S--L--F--V--N--S--L--I--A--S--I--C--M--R--K--V--G--V--S--N--L--V--N--L--F--V--G--W--Y--T--P--T--P--R--E--T--I--Q--L--N--G--G--R--L--Q--L--S--Q--F--I--R--Y--L--V--H--P--Y--P--$--G--H--H--T--M--W--I--$--S--Q--E--I--L--A--P--D--W--L--R--K--D--G--H--V--L--S--I--G--G--I--C--N--$--L--E--Q--D--A--A--V--Q--I--N--D--G--I--F--S--L--Y--L--T--E--K--L--G--M--W--L--V--S--T--R--L--R--I--A--E--K--I--V--E--V--E--Q--I--E--E--$--A--G--Q--R--N--I--A--G--$--A--T--A--R--E--L--F--P--G--R--L--Y--S--S--L--F--V--P--F--L--R--F--S--S--D--P--E--S--G--R--D--$--Y--V--A--Q--T--C--Q--A--L--S--V--C--V--E--N--P--C--P--Q--T--V--K--P--P--A--H--C--G--K--K--Y--A--G--S--H--P--E--I--P--P--P--P--P--S--P--T--H--H--S--H--I--$--Q--D--S--S--R--A--$--E--I--V--L--D--P--F--R--V--A--D--D--F--M--L--C--K--Q--A--F--V--V--S--V--L--A--$--R--L--S--F--F--Y--V--L--I--F--T--P--Q--P--L--S--S--P--R--E--V--L--R--K--S--C--G--G--Q--L--I--I--V--A--G--L--I--V--$--S--F--R--E--R--G--T--V--C--D--V--G--T--N--P--P--I--S--F--S--S--L--T--R--S--P--K--Y--L--D--R--S--S--M--F--L--R--M--F--P--S--L--E--P--N--H--E--E--N--F--S--P--R--E--V--T--P--P--G--F--V--$--P--L--Q--H--S--H--A--C--R--$--G--H--H--V--G--D--V--$--R--A--T--V--A--G--T--R--P--D--N--S--D--T--S--G--G--S--H--S--H--P--G--D--H--G--S--D--H--L--A--V--$--K--P--R--L--L--Y--S--W--V--S--A--Q--Q--$--R--S--M--G--T--T--N--T--I--L--R--Q--G--R--R--R--V--K--M--D--H--E--Q--S--K--R--Q--$--L--F--H--T--F--$--W--V--F--Q--R--$--H--I--Q--S--C--H--T--P--I--N--H--P--P--Q--R--Q--K--L--C--R--A--P--$--L--S--S--L--S--D--K--R--$--V--S--R--R--L--H--$--S--V--G--K--G--R--T--L--$--P--P--S--F--S--I--A--P--Y--G--R--A--F--K--T--E--N--V--L--G--W--A--F--S--$--P--L--D--E--T--L--P--I--Q--S--R--R--N--H--R--G--P--K--D--A--I--Q--R--R--L--H--D--P--H--R--$--K--K--W--L--L--A--H--R--P--D--G--R--E--L--D--L--L--W--L--S--I--R--$--R--S--V--R--L--L--I--P--A--I--R--L--V--R--V--S--L--Y--F--P--H--N--R--K--C--N--L--V--V--C--$--T--S--R--G--T--N--N--A--V--Y--$--$--P--F--E--R--R--S--H--S--P--S--A--H--D--H--S--T--T--P--P--P--I--H--A--S--T--E--S--K--L--R--H--V--$--V--A--H--E--S--G--L--L--P--Q--A--E--$--M--Y--H--H--P--D--T--D--F--G--T--L--R--C--W--V--E--L--Q--R--W--R--L--L--L--P--G--$--Q--T--S--K--I--H--N--T--Q--G--G--D--S--$--R--P--H--G--I--S--S--H--P--A--E--I--R--R--Q--M--R--L--N--G--A--Y--G--P--G--S--A--H--L--H--E--T--M--$--$--T--S--W--R--N--D--K--E--R--E--N--P--H--Q--A--S--S--R--R--S--P--$--N--R--T--L--A--L--C--R--$--$--H--E--A--G--P--M--E--G--R--A--T--L--V--H--H--E--S--I--R--C--V--R--L--R--M--G--C--G--T--A--R--G--T--Q--S--R--T--L--N--W--G--I--T--G--Q--S--R--I--E--L--R--T--S--I--V--K--K--R--K--Q--$--P--S--P--L--K--Q--P--N--N--G--S--L--F--Q--E--Y--T--$--K--$--T--T--G--R--$--$--T--R--G--K--A--A--G--P--G--L--C--R--C--A--E--$--G--N--Q--E--H--L--P--D--H--I--G--P--E--Y--I--A--I--Y--V--T--R--Q--N--K--R--$--R--G--R--S--T--I--Q--T--A--V--Q--N--R--L--H--V--G--T--S--S--L--A--T--C--A--I--S--I--W--G--S--A--G--T--F--S--R--L--D--G--V--G--F--$--R--T--E--R--Q--F--G--$--P--A--K--A--L--Y--P--L--S--N--A--G--V--S--R--C--R--R--I--Q--A--Q--S--H--N--F--S--G--G--R--E--R--E--C--V--C--F--P--T--H--Q--H--D--R--G--S--H--Q--F--P--D--T--M--E--S--R--R--D--G--P--Q--V--V--P--E--T--P--V--V--A--D--T--V--T--G--G--E--N--K--H--Q--I--N--R--S--R--K--$--F--S--S--M--V--S--I--Q--A--R--S--E--T--W--K--G--I--P--V--R--T--V--G--V--Q--D--R--T--R--I--R--L--H--P--G--T--L--P--$--K--N--S--R--T--Y--V--I--T--M--S--K--I--K--H--H--P--L--A--R--I--Q--P--L--P--L--I--P--R--Q--W--T--S--S--V--A--C--P--E--C--H--R--H--N--D--H--D--F--R--L--C--Q--M--C--G--Y--E--R--Q--P--F--P--P--P--R--E--K--L--D--V--D--D--D--K--I--Q--A--R--L--E--E--I--N--K--L--A--V--S--S--D--Y--G--K--K--K--A--A--L--E--N--E--L--R--N--F--L--G--N--R--S--P--P--K--D--L--T--T--A--S--P--K--D--V--C--S--F--L--V--W--K--D--E--G--G--G--G--T--I--V--H--K--T--G--C--K--N--F--G--E--K--R--K--S--V--C--G--C--P--K--R--L--A--A--G--T--V--D--S--V--I--G--Q--L--R--S--I--F--T--K--S--G--R--G--G--E--W--N--D--A--I--F--A--G--N--P--A--A--A--P--E--G--R--R--I--P--$--D--N--K--G--R--A--G--$--R--L--D--S--T--Y--T--S--S--T--G--L--P--$--Q--V--G--G--F--X--               >reconstructed Bflo               NPTMKKISRPGRSPRQASFDPYNIATRADEDTTWVTYDGRPWPEPVPTTPIPAEEAIATQETMDPTTLPFRNPDCFIAGSLHNNKEVWAQLTPFSDKGEDVLRWITNKVNVSDFFTPFDGSFKGNTYKAATPPSIILPNARNCVEHHDFLASQIKDRLADGSIKVWGRVGHCDPPHLVLPLTVEPSKPRMCWDGRFLNLWTKRCPFNLDAITEAPRMLYKGGYMTHTDEKSGYSHIGLTEESWTYFGFQFDDVLYVYSSLPFGWSASAFIFHTTGSVISSYARHLGVPTMLYIDDRLNGEATAPPPMTIALPLHPYTQAQKASYVMCELLTRVGYYLRLNKCIIIPTQILVHLGAGLNSKDGVFFFPDDKRLKFITLREEILSAPMVSLHTLQKFVGKCVSMALMVPGARIYTRQCNKLVGEMTKKGKIRTRLPAGVRPEIEHWRFVDNDMKPVPWRDERHSSITRASDASGYGWGAVLHGVPRVGRXTGGLLVRAGXSXGHQLXRsesssphpXSSQTMDRYFKSTHESRQQGGSRLVARQRGPGSAAALNEEIKNIFQTTLALNISLSMSHVKTKDNEADQPSRQLSKTDCMLAPHLWQRVQSAFGGQRGHSVDLMALDSNVQRDNSGNPLKHYTPYPTPESAGVDVFRHNPTTSPGGERENAYVFPPINMTGAVINFLIQWKADVTVPKLCPRPLWWPTLLRVAKTSIKLTEAGNSSALWFPSKQGLRPGKAFQYELWAFRIEQEXKNSRTYVITMSKIKHHPLARIQPLPLIPRQWTSSVACPECHRHNDHDFRLCQMCGYERQPFPPPREKLDVDDDKIQARLEEINKLAVSSDYGKKKAALENELRNFLGNRSPPKDLTTASPKDVCSFLVWKDEGGGGTIVHKTGCKNFGEKRKSVCGCPKRLAAGTVDSVIGQLRSIFTKSGRGGEWNDAIFAGNPAAAPEGRRIPXTPLSDNYDRFLRNRGGAANGTTRYLRETQQPHLRVGEYLRITKAEQANALIQPTQAQPVFHDKLEALCTHIAMKLRDPKTKESKLFTLARDQAFFKAMFFGADRAADLSRCKSEELAWLPEGEGILFNHTFGKTLRDGTANTFPILANENSAICPVRGLQAYFTMATALGINLSKGYLFRAMNKSKEVINEPFSYDAAQSRFKEYLIEINRYEGDTLHGLRTASAITIAMGGASQSALMAHVGWREKATAQRYMQLRKVCHEESPAAILREQVTPGARKDNPNKKRITDAATVYQCKNSTDYKPVL               <--------------------------------------------------------------------------preRT-----------------------------------------------------------------------------------------------><-------------------------------------------------------------------------------------------------------------------------------------------------------------------------RT-----------------------------------------------------------------------------------------------------------------><---------------------------------------------------------------------RNaseH-------------------------------------------------------------------><-------------------------------------------------------------Methylase-------------------------------------------------------------------------------------------------------><-----------------ZnR-----------------------------><-----------------ALOG----------------------------------------------------------------------------------------------------><----------------------------------------------------------------------------------------------------------------------------------------------------------------------------------------------------------Integrase-catalytic------------------------------------------------------------------------------------>                - Coordinates of other ALOG containing DIRS-1-like elements most of which are predicted to be inactive                                 ```                GI          Range         Strand orientation               260806091 : 253621..257310               260781169 : 58082..60771   (+ve strand)               260784594 : 283402..289134 (fragmented element)               ```               ```                Back to Contents                 ---                **- Structure of the ALOG domain-containing DIRS1-like transposons in Nematostella vectensis and coordinates of other fragmentary elements**                 ```               The below element is derived from the genome sequence of NEMVEDRAFT_v1g220156 of Nematostella (gi: 156352960)               Boundaries                                                                                                                                                                                                                                                                                                                                                                                             <------------------------Adenine methylase frame 3---------------------------------------------------------------------------------------------------------------------------------------------------------------------------------------------------------------------------------------------------------------------------------------------------------------------------------------------------------------------------------------------------------------------------------------------------------------------------------->                                                                                                                                                        <--Zinc ribbon--------------------------------------------->-----------------------------------------------------------------------------------------------------------<---frame 3 ALOG----------------------------------------------------------------------------------------------------------------------------------------------------------------------------------------------------------------------------------------------------------------------------------------------------------------------------------------------------------------------><--Frame 3 integrase------------------------------------------------------------------------------------------------------------------------------------------------------------------------------------------------------------------------------------------------------------------------------><--frame2---integrase-------------------------------------------------------------------------------------------------------------------------------------------------------------------------------------------------------------------------------------------------------------------------------------------------------------------------------------------------------------------------------------------------------------------------------------------------------------------------->               >fhj_rc   GGTGGGGCGGGTGTTTGTCCTAGCCATCGCAGGCTGATATCCAGACAAGGGGGTACTGGAACGAGGCCGAGTGGCAGCAACCTATTGCCGAGAAGGAAGCTCTGGCTTTGTTGTTCACGCTGGAAAATCTGCTTTGTCACTACACAAATGTTAGAGTCGTCTATACGGATAACAAGGTGCTACTAGGCGCGTGGCAGAGGCAAGTCTCGAAGTCGGCGGAAATATCGAGTATTATAAAGCGCTTATTCGCGTTTACGTTCGCCAAGAACCTGGCACTGGTCCTGTACTTTGTTCCTTCTCGCAACAACCCTGCCGATTCCCCATCGCGCGTATTATCCGATCTCGACTGCACTTTAAGTGCGCAGACATGGAGAAGCATTGATATCGCATTCGGCCCGCACTCTATTGACCTGATGGCGTTACCGTCTAACGTGATGCACGATCACGCCGGCCGCCCGTTGCGGTTTTTCTCCCAGCTCCCCTGCGTTCAAGCGGAGAGCACGAACGTTTTTGCGCATTCTCTGTTGCCCGAGGAAAACGCGTATGTCTTCCCACCCTTCATTCTCATGGGCCCGTTGCTTGGGCATCTCTCTAAGCGAGCTTGTCCGTTCTCTATTGTGGTGCCGGACATTACCCCGAGGAAGTATTGGTGGTCTGTTTTAAAGCGGAGAGCGGCGGCGTCTTTCAAGCTGGGCAGTCGGGGCTCGTTATCTTCGCTACTGTTCCCCGCTAAATCAGGAGCGGCTCCGTGGCTAAATCAGGAGCGGCTCCGTGGCAGCCGCGCGCCCTTCAATGGGACCTTTGGGTTTTCAGGGTTATCGCTCAAGAGAAGTGACTTTGTTGGTACGACGAGCTGTATTAGACTGATGTATATGGACAATCTGGGTTATGTTATGTAGCTACAGGGGGCAATACTTTTGCATTAAATACTTTCAGTAGCTTGAAACGAATTATGTTGTTTATTTTAATTAATTCACCCCTTCTTTTCCCGCCTTTTATTTTCTGTTTAGGACCTTCCCGAAGTGGTAAGGGCATGGGTTCCCGCGGTAGGATGTCCGGAGTGTGGGTATGCAAATGATCACACATTCTCGTTTTGCCAGAGATGTGGATTTCAGCGCCGGGAGAAAGATAAGGCCGACACCCAAAAGAAGATAAATATCGACCTTCCTTTTTTGGACCGGCGGTTAGAATCCCTTACATCCTCTAGGAATAATAAGCCATATCAGAAGCGAACGTCAAGCTTACTTAAGGAGCTGGAAAGGTTTTTAGACTCTTTGACGCCACCTAAAAACCTTATGTCGGCGTCCCCCCGAGATATTAATCGGTTTCTGGTCTGGAAGGATGAAGGGGGACGCACGAAGATTCATAAGCCAACATGTACAAAGTATGGTTCCGCGGGTTCGGCACGGTGTAGGTGTCCGTCTAGGCTAGCTGCAGGGACGGTCGACAGTATTATTGGCAAGTTGCGGGCAATTTTTGCGGAAGCGGGGAGGAAAGGCGAGTGGAATGAAATGCTAAACATTGGCAATCCCTCGTCACACCGGTCAGCAAAGGGTTATCTTACATCTATCCGAGAAGAGCAGGCGATGGCTCACGTAAGTCCGAAGCAGGCCACGCCAATTTTCTTTGATAAGCTAGCCAAGCTTTGCCGCTTCCTTCGGAACTTGGTTTTTGTCGAAAAAGCCACCTCTATCCAACGACATATTCACGCTAGGGATTTGGCGTTTTTCTGTTTGGATTTCTTTGCCGGCGATCGAGCTTCGGGCCTAGGGAGGGTTTTAACCAAGGAAGCGCTTGCTTCCAAAGATGGGGAAACCATCTGGTTCCGGCATACATACGGTAAGACGTTACGGGGCGGAGGGGATACTAACGTTTTTCCGATTAAGAAATATGCCTAGACCCCGTAGCCTGCCCGGTAGCGAACCTTCGTCTATATATCAAGCTATGTGATATAATGAAAATTAACCTGAGGGAAGGTTACATATTTAGGGCAACCGATGGTGCAACCAAGGTTTCAGACAACCCCTTTGTAGGTTCTACTATCGCGAACAGGCTTAAACTACACCTGGGTAAAGCGGACATCCTAGAAGGGGAGACGATGCACGGTTTTAGAAGCGGGTGCTCCATCACTCTCTCACTTCTTAGGGTGTCTACGGAAGATGTGGCAAGACACGTCGGCTGGAAGTCTACTTCTACAGCGGATTATTATTCGCAGACGGGGAAGGTTATGAACGCCGAGCGGGTGGCAGATGCACTAGCTGAAAGTTCTGTCCCAAACGCTACAGGGGAGACTCCGGCTCTTTCTTTGGCAGCCGATTTTACCACAAACAACAGGTTGGGTAATTTATCTCTCGCTTTTCCTTAGCTGAATGTATGCCGTTTTTTCTGAGTTTTGGGAGGAAGATAATAAAGCCGTCGAATATTCCCGGAGTCATTTATTGTGATAGTTTAGTCTCGTTACGCATGGGGTAAGAGGACAGGGTTAATGCCGGAGCCAAATTCGATTCCCATAACTTAACTCACGCGGAGTCAGTAAGCGGCATATTCTTGAGTGAAAATGATTCACTATTTTCATTAGAGGATATGTCGCGAAACACAACCGTGCGTGAGTGAGTTTACACCAAACTCCCTAGAGACATTCCGGGGTATGGTATGTAGATATTTGGCGAGTATGGGCCTATTTAAGAAATTTATCTAAAAGTCTCGAGGCTTTTAACGTA               fhj_rc_1  --G--G--A--G--V--C--P--S--H--R--R--L--I--S--R--Q--G--G--T--G--T--R--P--S--G--S--N--L--L--P--R--R--K--L--W--L--C--C--S--R--W--K--I--C--F--V--T--T--Q--M--L--E--S--S--I--R--I--T--R--C--Y--*--A--R--G--R--G--K--S--R--S--R--R--K--Y--R--V--L--*--S--A--Y--S--R--L--R--S--P--R--T--W--H--W--S--C--T--L--F--L--L--A--T--T--L--P--I--P--H--R--A--Y--Y--P--I--S--T--A--L--*--V--R--R--H--G--E--A--L--I--S--H--S--A--R--T--L--L--T--*--W--R--Y--R--L--T--*--C--T--I--T--P--A--A--R--C--G--F--S--P--S--S--P--A--F--K--R--R--A--R--T--F--L--R--I--L--C--C--P--R--K--T--R--M--S--S--H--P--S--F--S--W--A--R--C--L--G--I--S--L--S--E--L--V--R--S--L--L--W--C--R--T--L--P--R--G--S--I--G--G--L--F--*--S--G--E--R--R--R--L--S--S--W--A--V--G--A--R--Y--L--R--Y--C--S--P--L--N--Q--E--R--L--R--G--*--I--R--S--G--S--V--A--A--A--R--P--S--M--G--P--L--G--F--Q--G--Y--R--S--R--E--V--T--L--L--V--R--R--A--V--L--D--*--C--I--W--T--I--W--V--M--L--C--S--Y--R--G--Q--Y--F--C--I--K--Y--F--Q--*--L--E--T--N--Y--V--V--Y--F--N--*--F--T--P--S--F--P--A--F--Y--F--L--F--R--T--F--P--K--W--*--G--H--G--F--P--R--*--D--V--R--S--V--G--M--Q--M--I--T--H--S--R--F--A--R--D--V--D--F--S--A--G--R--K--I--R--P--T--P--K--R--R--*--I--S--T--F--L--F--W--T--G--G--*--N--P--L--H--P--L--G--I--I--S--H--I--R--S--E--R--Q--A--Y--L--R--S--W--K--G--F--*--T--L--*--R--H--L--K--T--L--C--R--R--P--P--E--I--L--I--G--F--W--S--G--R--M--K--G--D--A--R--R--F--I--S--Q--H--V--Q--S--M--V--P--R--V--R--H--G--V--G--V--R--L--G--*--L--Q--G--R--S--T--V--L--L--A--S--C--G--Q--F--L--R--K--R--G--G--K--A--S--G--M--K--C--*--T--L--A--I--P--R--H--T--G--Q--Q--R--V--I--L--H--L--S--E--K--S--R--R--W--L--T--*--V--R--S--R--P--R--Q--F--S--L--I--S--*--P--S--F--A--A--S--F--G--T--W--F--L--S--K--K--P--P--L--S--N--D--I--F--T--L--G--I--W--R--F--S--V--W--I--S--L--P--A--I--E--L--R--A--*--G--G--F--*--P--R--K--R--L--L--P--K--M--G--K--P--S--G--S--G--I--H--T--V--R--R--Y--G--A--E--G--I--L--T--F--F--R--L--R--N--M--P--R--P--R--S--L--P--G--S--E--P--S--S--I--Y--Q--A--M--*--Y--N--E--N--*--P--E--G--R--L--H--I--*--G--N--R--W--C--N--Q--G--F--R--Q--P--L--C--R--F--Y--Y--R--E--Q--A--*--T--T--P--G--*--S--G--H--P--R--R--G--D--D--A--R--F--*--K--R--V--L--H--H--S--L--T--S--*--G--V--Y--G--R--C--G--K--T--R--R--L--E--V--Y--F--Y--S--G--L--L--F--A--D--G--E--G--Y--E--R--R--A--G--G--R--C--T--S--*--K--F--C--P--K--R--Y--R--G--D--S--G--S--F--F--G--S--R--F--Y--H--K--Q--Q--V--G--*--F--I--S--R--F--S--L--A--E--C--M--P--F--F--L--S--F--G--R--K--I--I--K--P--S--N--I--P--G--V--I--Y--C--D--S--L--V--S--L--R--M--G--*--E--D--R--V--N--A--G--A--K--F--D--S--H--N--L--T--H--A--E--S--V--S--G--I--F--L--S--E--N--D--S--L--F--S--L--E--D--M--S--R--N--T--T--V--R--E--*--V--Y--T--K--L--P--R--D--I--P--G--Y--G--M--*--I--F--G--E--Y--G--P--I--*--E--I--Y--L--K--V--S--R--L--L--T--*--Y--               fhj_rc_2  --V--G--R--V--F--V--L--A--I--A--G--*--Y--P--D--K--G--V--L--E--R--G--R--V--A--A--T--Y--C--R--E--G--S--S--G--F--V--V--H--A--G--K--S--A--L--S--L--H--K--C--*--S--R--L--Y--G--*--Q--G--A--T--R--R--V--A--E--A--S--L--E--V--G--G--N--I--E--Y--Y--K--A--L--I--R--V--Y--V--R--Q--E--P--G--T--G--P--V--L--C--S--F--S--Q--Q--P--C--R--F--P--I--A--R--I--I--R--S--R--L--H--F--K--C--A--D--M--E--K--H--*--Y--R--I--R--P--A--L--Y--*--P--D--G--V--T--V--*--R--D--A--R--S--R--R--P--P--V--A--V--F--L--P--A--P--L--R--S--S--G--E--H--E--R--F--C--A--F--S--V--A--R--G--K--R--V--C--L--P--T--L--H--S--H--G--P--V--A--W--A--S--L--*--A--S--L--S--V--L--Y--C--G--A--G--H--Y--P--E--E--V--L--V--V--C--F--K--A--E--S--G--G--V--F--Q--A--G--Q--S--G--L--V--I--F--A--T--V--P--R--*--I--R--S--G--S--V--A--K--S--G--A--A--P--W--Q--P--R--A--L--Q--W--D--L--W--V--F--R--V--I--A--Q--E--K--*--L--C--W--Y--D--E--L--Y--*--T--D--V--Y--G--Q--S--G--L--C--Y--V--A--T--G--G--N--T--F--A--L--N--T--F--S--S--L--K--R--I--M--L--F--I--L--I--N--S--P--L--L--F--P--P--F--I--F--C--L--G--P--S--R--S--G--K--G--M--G--S--R--G--R--M--S--G--V--W--V--C--K--*--S--H--I--L--V--L--P--E--M--W--I--S--A--P--G--E--R--*--G--R--H--P--K--E--D--K--Y--R--P--S--F--F--G--P--A--V--R--I--P--Y--I--L--*--E--*--*--A--I--S--E--A--N--V--K--L--T--*--G--A--G--K--V--F--R--L--F--D--A--T--*--K--P--Y--V--G--V--P--P--R--Y--*--S--V--S--G--L--E--G--*--R--G--T--H--E--D--S--*--A--N--M--Y--K--V--W--F--R--G--F--G--T--V--*--V--S--V--*--A--S--C--R--D--G--R--Q--Y--Y--W--Q--V--A--G--N--F--C--G--S--G--E--E--R--R--V--E--*--N--A--K--H--W--Q--S--L--V--T--P--V--S--K--G--L--S--Y--I--Y--P--R--R--A--G--D--G--S--R--K--S--E--A--G--H--A--N--F--L--*--*--A--S--Q--A--L--P--L--P--S--E--L--G--F--C--R--K--S--H--L--Y--P--T--T--Y--S--R--*--G--F--G--V--F--L--F--G--F--L--C--R--R--S--S--F--G--P--R--E--G--F--N--Q--G--S--A--C--F--Q--R--W--G--N--H--L--V--P--A--Y--I--R--*--D--V--T--G--R--R--G--Y--*--R--F--S--D--*--E--I--C--L--D--P--V--A--C--P--V--A--N--L--R--L--Y--I--K--L--C--D--I--M--K--I--N--L--R--E--G--Y--I--F--R--A--T--D--G--A--T--K--V--S--D--N--P--F--V--G--S--T--I--A--N--R--L--K--L--H--L--G--K--A--D--I--L--E--G--E--T--M--H--G--F--R--S--G--C--S--I--T--L--S--L--L--R--V--S--T--E--D--V--A--R--H--V--G--W--K--S--T--S--T--A--D--Y--Y--S--Q--T--G--K--V--M--N--A--E--R--V--A--D--A--L--A--E--S--S--V--P--N--A--T--G--E--T--P--A--L--S--L--A--A--D--F--T--T--N--N--R--L--G--N--L--S--L--A--F--P--*--L--N--V--C--R--F--F--*--V--L--G--G--R--*--*--S--R--R--I--F--P--E--S--F--I--V--I--V--*--S--R--Y--A--W--G--K--R--T--G--L--M--P--E--P--N--S--I--P--I--T--*--L--T--R--S--Q--*--A--A--Y--S--*--V--K--M--I--H--Y--F--H--*--R--I--C--R--E--T--Q--P--C--V--S--E--F--T--P--N--S--L--E--T--F--R--G--M--V--C--R--Y--L--A--S--M--G--L--F--K--K--F--I--*--K--S--R--G--F--*--R--I--K--M--N--R--S--T--               fhj_rc_3  --W--G--G--C--L--S--*--P--S--Q--A--D--I--Q--T--R--G--Y--W--N--E--A--E--W--Q--Q--P--I--A--E--K--E--A--L--A--L--L--F--T--L--E--N--L--L--C--H--Y--T--N--V--R--V--V--Y--T--D--N--K--V--L--L--G--A--W--Q--R--Q--V--S--K--S--A--E--I--S--S--I--I--K--R--L--F--A--F--T--F--A--K--N--L--A--L--V--L--Y--F--V--P--S--R--N--N--P--A--D--S--P--S--R--V--L--S--D--L--D--C--T--L--S--A--Q--T--W--R--S--I--D--I--A--F--G--P--H--S--I--D--L--M--A--L--P--S--N--V--M--H--D--H--A--G--R--P--L--R--F--F--S--Q--L--P--C--V--Q--A--E--S--T--N--V--F--A--H--S--L--L--P--E--E--N--A--Y--V--F--P--P--F--I--L--M--G--P--L--L--G--H--L--S--K--R--A--C--P--F--S--I--V--V--P--D--I--T--P--R--K--Y--W--W--S--V--L--K--R--R--A--A--A--S--F--K--L--G--S--R--G--S--L--S--S--L--L--F--P--A--K--S--G--A--A--P--W--L--N--Q--E--R--L--R--G--S--R--A--P--F--N--G--T--F--G--F--S--G--L--S--L--K--R--S--D--F--V--G--T--T--S--C--I--R--L--M--Y--M--D--N--L--G--Y--V--M--*--L--Q--G--A--I--L--L--H--*--I--L--S--V--A--*--N--E--L--C--C--L--F--*--L--I--H--P--F--F--S--R--L--L--F--S--V--*--D--L--P--E--V--V--R--A--W--V--P--A--V--G--C--P--E--C--G--Y--A--N--D--H--T--F--S--F--C--Q--R--C--G--F--Q--R--R--E--K--D--K--A--D--T--Q--K--K--I--N--I--D--L--P--F--L--D--R--R--L--E--S--L--T--S--S--R--N--N--K--P--Y--Q--K--R--T--S--S--L--L--K--E--L--E--R--F--L--D--S--L--T--P--P--K--N--L--M--S--A--S--P--R--D--I--N--R--F--L--V--W--K--D--E--G--G--R--T--K--I--H--K--P--T--C--T--K--Y--G--S--A--G--S--A--R--C--R--C--P--S--R--L--A--A--G--T--V--D--S--I--I--G--K--L--R--A--I--F--A--E--A--G--R--K--G--E--W--N--E--M--L--N--I--G--N--P--S--S--H--R--S--A--K--G--Y--L--T--S--I--R--E--E--Q--A--M--A--H--V--S--P--K--Q--A--T--P--I--F--F--D--K--L--A--K--L--C--R--F--L--R--N--L--V--F--V--E--K--A--T--S--I--Q--R--H--I--H--A--R--D--L--A--F--F--C--L--D--F--F--A--G--D--R--A--S--G--L--G--R--V--L--T--K--E--A--L--A--S--K--D--G--E--T--I--W--F--R--H--T--Y--G--K--T--L--R--G--G--G--D--T--N--V--F--P--I--K--K--Y--A--*--T--P--*--P--A--R--*--R--T--F--V--Y--I--S--S--Y--V--I--*--*--K--L--T--*--G--K--V--T--Y--L--G--Q--P--M--V--Q--P--R--F--Q--T--T--P--L--*--V--L--L--S--R--T--G--L--N--Y--T--W--V--K--R--T--S--*--K--G--R--R--C--T--V--L--E--A--G--A--P--S--L--S--H--F--L--G--C--L--R--K--M--W--Q--D--T--S--A--G--S--L--L--L--Q--R--I--I--I--R--R--R--G--R--L--*--T--P--S--G--W--Q--M--H--*--L--K--V--L--S--Q--T--L--Q--G--R--L--R--L--F--L--W--Q--P--I--L--P--Q--T--T--G--W--V--I--Y--L--S--L--F--L--S--*--M--Y--A--V--F--S--E--F--W--E--E--D--N--K--A--V--E--Y--S--R--S--H--L--L--*--*--F--S--L--V--T--H--G--V--R--G--Q--G--*--C--R--S--Q--I--R--F--P--*--L--N--S--R--G--V--S--K--R--H--I--L--E--*--K--*--F--T--I--F--I--R--G--Y--V--A--K--H--N--R--A--*--V--S--L--H--Q--T--P--*--R--H--S--G--V--W--Y--V--D--I--W--R--V--W--A--Y--L--R--N--L--S--K--S--L--E--A--F--N--V--                 - Coordinates of other ALOG containing DIRS-1-like elements most of which are predicted to be inactive                GI         Range          Strand orientation/notes               156352963 :84557..88045 (-ve strand)               156381872 : 648524..651943 (-ve strand)               156372905 : 503045..500114 (-ve strand)               156407154 : 855264..858406 (-ve strand)               156384295 : 237253..240280 (-ve strand)               156367185 : 36005..38522 (-ve strand)               156405776 : 289156..292020 (-ve strand)               156348365 : 6811..9108 (-ve strand)               156354991 : 46867..49527  (-ve strand)               156369722 : 30154..31573  (very fragmented)               156351445 : 77397.. 81734(+ve strand)               156368559 : 449489..455510 (+ve strand)               156395194 : 873010..876708 (+ve strand)               156358315 : 180879..184279 (+ve strand)               156363194 : 321555..324323 (+ve strand)               156375098 : 540878..543883(+ve strand               156375098 : 540878..543904(+ve strand)               156358418 : 65454..70426 (+ve strand)-- missing integrase               156340835 : 8148..13740 (+ve strand)-- ALOG inactivated? or is it classical DIRS1?               156338653 : 2..2604 (Missing RT)               156394357 : 1005492..1184431 (Missing integrase)               156354012 : 140646..145923 (Missing integrase)               156360556 : 77489..79835 (Missing RT N-terminus)               156370919 : 274333..276687 (Missing integrase)               156378492 : 638587..640239  (Missing C-terminal integrase)               ```                Back to Contents                 ---                **- Structure of the ALOG domain-containing DIRS1-like transposons in Crassostrea gigas**                 ```               Boundaries                                                                                                                                                                                                                                                                                                                                                                                                                                                                                                                                                                                                                                                                                                                                                                                                                                                                                                                                                                                                                                                                                                                                                                                                                                                                                                                                                                                                                                                                                                                                                                                                                                                                                                                                                                                                                                                                                                                                                                                                                                                                                                                                                                                                                                                                                                                                                                                                                                                                                                                                                                                                                                                                                                                                                                                                                                                                                                                                                                                                                                                                                                                                                                                                                                                                                                                                                                                                                                                                     <--frame 3 fragmented RT-----------------------------------------------------------------------------------------------------------------------------------------------------------------------------------------------------------------------------------------------------------<--frame 2 RT------->------------------------------------RT ends---->< RNaseH continues frame 2-----------------------------------------------------------------------------------------------------------------------------------------------------------------------------------------------------------------------------------------------------------------------------------------------------------------------------------------------------------------------<--DNA methylase continues in same frame-2with stop codons------------------------------------------------------------------------------------------------------------------------------------------------------------------------------------------------------------------------------------------------------------------------------------------>                                                                                                                                                                                                                                                              <--ALOG in frame 1---------------------------------------------------------------<-continues in frame 3--------------------------------------------------------------------------------------------------------------->               element1_Cgig_270063446      .       1        :         :         :         :         50        :         :         :         :         100       :         :         :         :         150       :         :         :         :         200       :         :         :         :         250       :         :         :         :         300       :         :         :         :         350       :         :         :         :         400       :         :         :         :         450       :         :         :         :         500       :         :         :         :         550       :         :         :         :         600       :         :         :         :         650       :         :         :         :         700       :         :         :         :         750       :         :         :         :         800       :         :         :         :         850       :         :         :         :         900       :         :         :         :         950       :         :         :         :         1000      :         :         :         :         1050      :         :         :         :         1100      :         :         :         :         1150      :         :         :         :         1200      :         :         :         :         1250      :         :         :         :         1300      :         :         :         :         1350      :         :         :         :         1400      :         :         :         :         1450      :         :         :         :         1500      :         :         :         :         1550      :         :         :         :         1600      :         :         :         :         1650      :         :         :         :         1700      :         :         :         :         1750      :         :         :         :         1800      :         :         :         :         1850      :         :         :         :         1900      :         :         :         :         1950      :         :         :         :         2000      :         :         :         :         2050      :         :         :         :         2100      :         :         :         :         2150      :         :         :         :         2200      :         :         :         :         2250      :         :         :         :         2300      :         :         :         :         2350      :         :         :         :         2400      :         :         :         :         2450      :         :         :         :         2500      :         :         :         :         2550      :         :         :         :         2600      :         :         :         :         2650      :         :         :         :         2700      :         :         :         :         2750      :         :         :         :         2800      :         :         :         :         2850      :         :         :         :         2900      :         :         :         :         2950      :         :         :         :         3000      :         :         :         :         3050      :         :         :         :         3100      :         :         :         :         3150      :         :         :         :         3200      :         :         :         :         3250      :         :         :         :         3300      :         :         :         :         3350      :         :         :         :         3400      :         :         :         :         3450      :         :         :         :         3500      :         :         :         :         3550      :         :         :         :         3600      :         :         :         :         3650      :         :         :         :         3700      :         :         :         :         3750      :         :         :         :         3800      :         :         :         :         3850      :         :         :         :         3900      :         :         :         :         3950      :         :         :         :         4000      :         :         :         :         4050      :         :         :         :         4100      :         :         :         :         4150      :         :         :         :         4200      :         :         :         :         4250      :         :         :         :         4300      :         :         :         :         4350      :         :         :         :         4400      :         :         :         :         4450      :         :         :         :         4500      :         :         :         :         4550      :         :         :         :         4600      :         :         :         :         4650      :         :         :         :         4700      :         :         :         :         4750      :         :         :         :         4800      :         :         :         :         4850      :         :         :         :         4900      :         :         :         :         4950      :         :         :         :         5000      :         :         :         :         5050      :         :         :         :         5100      :         :         :         :         5150      :         :         :         :         5200      :         :         :         :         5250      :         :         :         :         5300      :         :         :         :         5350      :         :         :         :         5400      :         :         :         :         5450      :         :         :         :         5500      :         :         :         :         5550      :         :         :         :         5600      :         :         :         :         5650      :         :         :         :         5700      :         :         :         :         5750      :         :         :         :         5800      :         :         :         :         5850      :         :         :         :         5900      :         :         :         :         5950      :         :         :         :         6000      :         :         :         :         6050      :         :         :         :         6100      :         :         :         :         6150      :         :         :         :         6200      :         :         :         :         6250      :         :         :         :         6300      :         :         :         :         6350      :         :         :         :         6400      :         :         :         :         6450      :         :         :         :         6500      :         :         :         :         6550      :         :         :         :         6600      :         :         :         :         6650      :         :         :         :         6700      :         :         :         :         6750      :         :         :         :         6800      :         :         :         :         6850      :         :         :         :         6900      :         :         :         :         6950      :         :         :         :         |    .               element1_Cgig_270063446      1       AGACAAGGATGGTACACATATTCAGCAGGTGAAGGACAAGATTTAAGTTCTCTGTACCCAGTCCCAGTCCAGTGAAGCACTGATTTATTAAGTTTAGATGAATTATCTAGGTTAGCTAGAAAAGTGAACCATGAGGACATGGATGTGTATGAAGAACTGTATAGAAAATGTACATGTACAGTACAGCTCACGAGTATCCCGACACCCACCGTGTAAAAAACACGGTGGAAAACGGTCTGTGTCGCACCGTGTACACGGTGTGACACCGTGTATGTGTATTGGAAAATTCAAGAAAAAGCACCGTGTCGCACGGTGGCCGCCCGTGTAACTCCGTGGCCACTGTGTTACTCCGTGGATATTGTTACCTGAGACGTTGATAGAAATAGCGTATGTTTAGAAATAAATAAATTATGGCTAAACAAGGGTTGAAACATATATATCCTTTTCATATTTAAAAAAGAAATGAATATCGACTTAAGTTGCACGGACCCAGAAAATTGTCGGGGCTGTCAACGTGAAGTTATTTTTTTGTGATCTGAAAACTCGACGTACATGGGTACCTTTTAATTCATTTGTTCTTAAATAAATTGAAATAAATTCACCAAATCTATTTAATAATCAATTGATATATAATCAAAATTCAAATCAATTTAATTCATCGTGTTGTTTGTTTCTAAACACGCTTGATGTTGTAACTGACGATCCGATTAAAATATCGGGAGCTTAAACGTCTTATTTTCTGTTATATCTGAAAATACTTTCTGATTTATAAATTAATTTACAGCCTTACTTTAAACCAATCAAAAATGAGAAATGAAAACATTTCAGATCATGTTTTTTTTTAAAACATGTTGATGTGCTGTAGTAAATAACAACCCCATATTACCGGTGTCTCGAATAATTTTGACTTCAGCTATTTATGTAGCAATAAATAAACAAAAAATCTCTTTTATATTTTAGATTATAGATAAATACATGTACATACTCTTGTTAAAGAAATATTGAAGCATTGTACATGTACTAAACATATACCCGCATTTCATAAAATAACTTTTAACTTTATTTCCGTACAGCTGGTAATGGCGCCGTGATTTCACATGAAAAAATCACTCGTGCCATACACCTGTACGGAAGCTGATCAGATACACAACATTGTCTTTCATCTTGGGTTCTATACACAACAGGTGTTATTGTCTGTCTTGTTAGGTGTCATTTTAATTTACAACTAACTTTGTGTATATTTGGACGTCTGAACAGGTGTACTCGAGATGCCGTTATTCACACCTTTCCACGGACACCTGTTTGGTAACTCATCACAACCCGTCGTGTGTATGGTCTGAATATATTTTACTTCTACTTTGTTAGTTCCATGATTTTTCCTAATCTCTAACAAACAAAACAAATTGTCTGTAGATGTGTACACAAACTACTACACGTAAGACTATCGGCGCTTGGATCCGGAGAACAATTCCGAATTCAGCAACTCTATAAATGCGGGAATTTCACAAAGTATTAACTTGCGATAAGAAATTATTTACCGGGTACACATACATGTATGTACGAAAAAACTGATTGATTAAAAATATGAATGAGATAATACCGGTAACTTTTTGCAAGCATTTATAACAAACACAACTTTATTTACTTTATAACCAATCTAATATGTGTGATGTGAAATAGCGTCGAAATTTTAACGAAAAATCTTATTACATCCTATACTTTACAAACTTCAAGTCATTGTGTTGAAATCGTTTATTAAAACCATGGATCTAAAATAAATGAATTAAATTCATACAAATAGAACTCTTATAATTCTAAAATGATTGCACAAAAGACAAAAGTACAGAGAGAGAGAGAGAGAGAGAGAGATTTTACAGCGCAATATTCCAACCCTCATACATGCAGTATAAGAATGAAAAAACTAAACATTTCAAACACTAAATACTTCATTGTTAAACATTTTTCTATTTTGCGGACTTAAAAAAAAACAATAGAACGACTTTTATTCCTTATCATGGAAGGCATGGAAGGAGCACGTGGTCTATCTCGCGGGCTGATTTGATTGACAGGGATAGCACGTGGACTCTGACTGCATAGCTTCTATCGCAATTTTAGGGAAAAGGTTTCAATATAAGGGGAACTATAAATCTAACTTATTACACTGAATAACAGGTGAAATGTACTTAAAATTAAACTCATACTGTACCAGTATTCTCTCTCTCTCTCTCTCTCTCTCTCTCTCTCTCTCTCTCTCTCTCTCTCTCTCTCTCTCTCTCTCTCTCTCTCTCTCTCTCTCTCTCTCTCTCTCTCTCTCATATGGCGATCATTAAACAATCAAACAGTAATTATTGCAATACTGAGTAGTTTCTTGGAAGACAAATAATTTCTAAACTCAAGTTGCCTATAAAGATGAAATAAAAACTTATCAAGTACAGATCCACGGATTCAAAGCGCTATGAATATGTACCGTGCGGTACTACATGGACAAGTACATCACACATATGTATAAAATAAAGAAAATTTAAAAATATGAATATAAAGCTGTATAATTATATTATTTAGAGAAAGTACAGTTGTTTTCAACAACTTGTTAGATTTGTTATCGTTGAATAGAGTATAAGTGAGAGAAGATCACATATGATTCTATGCACTATTGAAACTGCCAATCTACAGCAACTACGATTACTACTACGTTTAAAAACGGACTGAAACAAAAACAAAAACTTATGGAACAGTGTATTTGAATTTTAAAAAGGAAAATAAAGTGTTAGTGTTTTATTTGATCAAACCTTTATGTGGGCGATATCGATTTTTTTGTTCTTAATATCAACAAAAATCAACCGTAACAATCGCATACGTCTGAAAATTGCGTGCCTTAAAATTTTATTTATTTATTTCTATTTCTTTGTCTTTTTCTTTAATTCTCTTACGTACATCAATAAATATTTTATTCTTTATAAGTTTTATTCCTAAATCCTCCTTTTTATCACAGGTAAATACTGCGTGTTACACCGTGTTACTGTCCGTGTTGCATCGCGTTGCACCGTGTCGCACCGTGTTTTTTCTCGTTCCGTGGCCGTAACAGAGAACAATAGATCAAAGGTACCGACACTTCCACGCTCAGCGTGGACTTTCAAACACGGTGGTCGGTGTTCTTGTCGGGATACTCGTGAGCTGTACTGTAGTAGGATGCAGAGCAAGATTAACATTGGAAATGTTTTGTATAACGGTGCTTGGGGGGAAAGGTGCAGGTACAGTGACTAAGGCCATAAATAAATGTTTAAAGGAGAGTGGGGTTTCTGAAGTTTATCATGGTTTACACAGTTTTGCCTTTTGGATGGAAGGCCTCACCATATATTTATCAAACTGTTGGAATGACAATTACATATTTTCTTAGAAAATTGAATGTAATAACAACTCAGTATATTGATGACCGTTTGGTGATAGCTAACCCATCAGAAGGCACTACTGACTTGGAAGCAAAAGAACATTGTTTTTGGGTGATATATGCTCTACTTCAAATTTTAATCCGTTTAGGTTACACAGTATCACTTGAAAAATCAAATACTGGCGTTTCTTAGACTCGTGGAAGGGTTACGCAAAGTGGAGGTCAGAGAGTCATAAGCAAGAAAATACCAGTATTTCAACAGATGCTTCTGGCTACAAGTATGGGGCAGTAGTTTGTTTTCAGGGGGAAAAGGTTGAACTTGGAGATTTTTTGGAGACAAACGACAGTAGACCTATTCATTTGAAGGAAGCTGAGGCAGTCCTTCAGGTCTTGAAGTCAATAGAGGAAGTAATTAGAGATTCAAGGGTTGACTTACTGGTAGATAATGTTGCTGTTCTTAGTGTTTGGAACAATCAAGGAGGACGGGATAGATCTTTGAACAACATAACAAAACAGATATTTCAGTTGGTGACTTCTTGTAATTGTGATCTGCACATGCAATATGTACCCTCAGAGTGAATGGGGCAGATTTACCTGTTTGAATGCATGTTTGAATATTGGTTCATGGCAGCGTGTTGAAAGGCAATTTGGACCTCATTCAATGGACCTCATGGCTATTGATTCTAATGTGATGAAATCTTCAGGAGGTACAGCACTACCAAATTTAACCCATATCCTACCCCATATTCATCTTGTGTAAATTTGTTTGCTCAGGATTTGCCAGTGGTGGTTAATGCATATGTTTATCCTCCTTTTGCCCTTATACATCCTGTACTCTTATTTTTAAGGGAACAGAAAGTTGGTATGTGCACCTTTGTCCTACCATTGATCAAGCCTGTACCTGTTTGGTGGCCTCTTGTTCAAAAGCATGTAATCCAGAGTTTAGAGTTAGGGACCAGGGGAGATAAGGCAGTAATAAGGGTTCCTTAGAAAAGGTTTCATCACTGATAATAAGGGTCTGAGATGGCCACTAGTTGCATTCAGGCTATTGTTTATCTAAGCTAATTGTTTTTCATTTTGTAGGGAGAAAGCTTGGATCTGGTTAAGATTGACAAGAGGATTAAGGAATTGAATGACAAATCTTCATCATCATCTTTATCTAAGAGAAGAGATACTTTGAAAAACTCTCTAAATTTATTTCTTCCACTATTGAGGAAAAATTGCACAATATCTAATTGTACCCCTGATGATCTAAAGCATTTCTGGTATGGAAGGATGATTTTGGTAAAACACCAGTTCATAAAATAAATTGTACATTTTTGGGTACTAAAGTTGTGAATGCCCAAGAGGATTAGCAGCTGGGACAGTATCGGTCATGGTATAAAATTTGTCGGAGATCTTTTACGAAAGTGGTCGTGGTAAATTCTGGGATGAGAGAAGTAATACAGGTAACCCTGCAGCGGCACATTGTATCAAACAGTATGTGAAACTTATTCAAGAGGAACAAGCGGCAGCACATGTAGTTTCTAAGCAGGCAAATCCAACTTTTCTGGGTAAAATAAACAGAATTGTCTCGTATATAGATAATGAATTGGGTAGGGTTGATATTTCATCGAAGGAAAGATATGTGCACCTTAAAGATCAAGCTTGGAGGAAATTACAGTTCTTTGGGGGAAATCGGGCAAATGACTAGGGAATGCTTGTAGGACAGGAGGTAAAGAGATTAGATGACAATTCTGGTTTAGTTAAAAAACAAACATTTGGGAAAACTTTAAGAGGAAATAAACATAAATCTCATGTTTTTGTTATTAAGAAATGCAGAGACAAGACTGTTTGTCCAGTAACTGGTTTGCACACATATGTTCAAGATTGTAAAAGAATGGGCGTGTATCTTGCAAATGGTTTTCTTTTTCGAGTTGTTTTAGAGAATGGTAGAGTCATAGGTGATAGAGTGACATATTCAGTAATGTATGAAAGACTGATTCGGTATCTTACTTTGTTAGGAATATATGAGGGTGAGACTCCTCATAGTTTTAGAGTCGGGTGTGCAGTGACTCTGGCTTTATCTGGTTCGGTTGCCAATGTAGGACAAATTATGAACCATGTGGGATGGTAGGGAGAGGGAACAGCAGAATATTATAGTAGATTGCCAGCTTTAGTGGAATCTGATTATATTGCAGGTAGATTAGCAGACAGTGCAGGTCAATGTGATTTTGTGGAAGATCAGTTCCAACAGTATGGGGATTTTAATATTTTACAAAAAGCTTTTCATAAACGATAAGGCCTTCAAAATGTCAATACAATTAAGGGAATAAGACAGATCCTTTGTTTTCGAATTTGCAGTAGATTAATTATCCCAAGTCTAATACGTCACCGCGAAAGTGATGACGTCATCTTTCTCAAAGATGTACCAATGATACTGAATATTGAGGATGAGCACGGTAGATGGCATTGATGGTTCAGAGTATCCATGAGTTTACCCCATCCACCCCACCCTTGTATATGTTCTTGTTCTCGGTTCATGATAATGACTACATTTCCCAATACATGAGAAGAACTAGAGCACGAACGTATACACCTGGGATAGGTATAGGTGCAATGTCCATCTCTATTTCATCAATTTAAATTTTGCAATTTCTTACTCTTTGCAGACGTATATGTTGTACATTTGATGTTCAAATGCAACATTTTACATGTAATGATAAAACATACCTATGTCTGGGATCAGAAGAACCAGTAGGAAAAGAATTGCGAAAATCTCCATGAAACATGTCAAGAGTCCCCCAACTACTGCCTCCGACACGGGGAATGCCAACTCAGATCCCATCTCGTAAACCAACGGAAAAGAGCCGTTGATTAACATTCCTCCGATGATAGCTGATATGTAGATTTCAACTGCAAGAAATCATATTTTGTTGAATTTTTACCATCTTTTACATCAAATGTGACAAAGTTTCTATATTTTTATAAAATACTCTACTAAAATACGATAAGGTAAATTTTCAACAACCGCGTCTGTACACTAATTCTAAATCATGGTATCATCTAAATAACAAGTGGTGATCATATCATTGGGAAACGGAAATTCAATCTTTCCAAAAACGTATTGATTAATAAAGAGAATGATAACACTTACTCTTAAATTCATCAATTTGAAACAATGATACCTTTAGAGATAGGTATGATTCCAGAGCTCATGAGAGAAAACCACACATAGCCTAGGACTGCTCCGCCATTCATCAAGATTAAAAAGAATTTAGTGTGTCTCAAAAATATATCTGAAAATCTGCAACAAAATTTAATACAGATAACTATCACAGCTATTTATGAATTGTCATTCTACCCGATAAACATGATTACAAAAGATGTTCGTGTCCAAAATACCTCCCAATAAGAAGTCCAGACACGCCTCCAGCTGCTGTCATGTAGAATCCTAACCATCCGACTTCACTCTTAAGATATTATGGAGAAAACACAGCATTTCGCATATTTTAGTCTAAGTGGACAGATTAATTTTCTTTGCTTGAAGTTCAAGGTCCATAAACTGCTTATAAACTGTCTTTATGCTTTAAGATTTCAGTATGCATGTAATTTGCTAAACCCTGACTACAAT   7001               element1_Cgig_270063446      1       R--Q--G--W--Y--T--Y--S--A--G--E--G--Q--D--L--S--S--L--Y--P--V--P--V--Q--*--S--T--D--L--L--S--L--D--E--L--S--R--L--A--R--K--V--N--H--E--D--M--D--V--Y--E--E--L--Y--R--K--C--T--C--T--V--Q--L--T--S--I--P--T--P--T--V--*--K--T--R--W--K--T--V--C--V--A--P--C--T--R--C--D--T--V--Y--V--Y--W--K--I--Q--E--K--A--P--C--R--T--V--A--A--R--V--T--P--W--P--L--C--Y--S--V--D--I--V--T--*--D--V--D--R--N--S--V--C--L--E--I--N--K--L--W--L--N--K--G--*--N--I--Y--I--L--F--I--F--K--K--E--M--N--I--D--L--S--C--T--D--P--E--N--C--R--G--C--Q--R--E--V--I--F--L--*--S--E--N--S--T--Y--M--G--T--F--*--F--I--C--S--*--I--N--*--N--K--F--T--K--S--I--*--*--S--I--D--I--*--S--K--F--K--S--I--*--F--I--V--L--F--V--S--K--H--A--*--C--C--N--*--R--S--D--*--N--I--G--S--L--N--V--L--F--S--V--I--S--E--N--T--F--*--F--I--N--*--F--T--A--L--L--*--T--N--Q--K--*--E--M--K--T--F--Q--I--M--F--F--F--K--T--C--*--C--A--V--V--N--N--N--P--I--L--P--V--S--R--I--I--L--T--S--A--I--Y--V--A--I--N--K--Q--K--I--S--F--I--F--*--I--I--D--K--Y--M--Y--I--L--L--L--K--K--Y--*--S--I--V--H--V--L--N--I--Y--P--H--F--I--K--*--L--L--T--L--F--P--Y--S--W--*--W--R--R--D--F--T--*--K--N--H--S--C--H--T--P--V--R--K--L--I--R--Y--T--T--L--S--F--I--L--G--S--I--H--N--R--C--Y--C--L--S--C--*--V--S--F--*--F--T--T--N--F--V--Y--I--W--T--S--E--Q--V--Y--S--R--C--R--Y--S--H--L--S--T--D--T--C--L--V--T--H--H--N--P--S--C--V--W--S--E--Y--I--L--L--L--L--C--*--F--H--D--F--S--*--S--L--T--N--K--T--N--C--L--*--M--C--T--Q--T--T--T--R--K--T--I--G--A--W--I--R--R--T--I--P--N--S--A--T--L--*--M--R--E--F--H--K--V--L--T--C--D--K--K--L--F--T--G--Y--T--Y--M--Y--V--R--K--N--*--L--I--K--N--M--N--E--I--I--P--V--T--F--C--K--H--L--*--Q--T--Q--L--Y--L--L--Y--N--Q--S--N--M--C--D--V--K--*--R--R--N--F--N--E--K--S--Y--Y--I--L--Y--F--T--N--F--K--S--L--C--*--N--R--L--L--K--P--W--I--*--N--K--*--I--K--F--I--Q--I--E--L--L--*--F--*--N--D--C--T--K--D--K--S--T--E--R--E--R--E--R--E--R--F--Y--S--A--I--F--Q--P--S--Y--M--Q--Y--K--N--E--K--T--K--H--F--K--H--*--I--L--H--C--*--T--F--F--Y--F--A--D--L--K--K--N--N--R--T--T--F--I--P--Y--H--G--R--H--G--R--S--T--W--S--I--S--R--A--D--L--I--D--R--D--S--T--W--T--L--T--A--*--L--L--S--Q--F--*--G--K--G--F--N--I--R--G--T--I--N--L--T--Y--Y--T--E--*--Q--V--K--C--T--*--N--*--T--H--T--V--P--V--F--S--L--S--L--S--L--S--L--S--L--S--L--S--L--S--L--S--L--S--L--S--L--S--L--S--L--S--L--S--L--S--L--S--H--M--A--I--I--K--Q--S--N--S--N--Y--C--N--T--E--*--F--L--G--R--Q--I--I--S--K--L--K--L--P--I--K--M--K--*--K--L--I--K--Y--R--S--T--D--S--K--R--Y--E--Y--V--P--C--G--T--T--W--T--S--T--S--H--I--C--I--K--*--R--K--F--K--N--M--N--I--K--L--Y--N--Y--I--I--*--R--K--Y--S--C--F--Q--Q--L--V--R--F--V--I--V--E--*--S--I--S--E--R--R--S--H--M--I--L--C--T--I--E--T--A--N--L--Q--Q--L--R--L--L--L--R--L--K--T--D--*--N--K--N--K--N--L--W--N--S--V--F--E--F--*--K--G--K--*--S--V--S--V--L--F--D--Q--T--F--M--W--A--I--S--I--F--L--F--L--I--S--T--K--I--N--R--N--N--R--I--R--L--K--I--A--C--L--K--I--L--F--I--Y--F--Y--F--F--V--F--F--F--N--S--L--T--Y--I--N--K--Y--F--I--L--Y--K--F--Y--S--*--I--L--L--F--I--T--G--K--Y--C--V--L--H--R--V--T--V--R--V--A--S--R--C--T--V--S--H--R--V--F--S--R--S--V--A--V--T--E--N--N--R--S--K--V--P--T--L--P--R--S--A--W--T--F--K--H--G--G--R--C--S--C--R--D--T--R--E--L--Y--C--S--R--M--Q--S--K--I--N--I--G--N--V--L--Y--N--G--A--W--G--E--R--C--R--Y--S--D--*--G--H--K--*--M--F--K--G--E--W--G--F--*--S--L--S--W--F--T--Q--F--C--L--L--D--G--R--P--H--H--I--F--I--K--L--L--E--*--Q--L--H--I--F--L--E--N--*--M--*--*--Q--L--S--I--L--M--T--V--W--*--*--L--T--H--Q--K--A--L--L--T--W--K--Q--K--N--I--V--F--G--*--Y--M--L--Y--F--K--F--*--S--V--*--V--T--Q--Y--H--L--K--N--Q--I--L--A--F--L--R--L--V--E--G--L--R--K--V--E--V--R--E--S--*--A--R--K--Y--Q--Y--F--N--R--C--F--W--L--Q--V--W--G--S--S--L--F--S--G--G--K--G--*--T--W--R--F--F--G--D--K--R--Q--*--T--Y--S--F--E--G--S--*--G--S--P--S--G--L--E--V--N--R--G--S--N--*--R--F--K--G--*--L--T--G--R--*--C--C--C--S--*--C--L--E--Q--S--R--R--T--G--*--I--F--E--Q--H--N--K--T--D--I--S--V--G--D--F--L--*--L--*--S--A--H--A--I--C--T--L--R--V--N--G--A--D--L--P--V--*--M--H--V--*--I--L--V--H--G--S--V--L--K--G--N--L--D--L--I--Q--W--T--S--W--L--L--I--L--M--*--*--N--L--Q--E--V--Q--H--Y--Q--I--*--P--I--S--Y--P--I--F--I--L--C--K--F--V--C--S--G--F--A--S--G--G--*--C--I--C--L--S--S--F--C--P--Y--T--S--C--T--L--I--F--K--G--T--E--S--W--Y--V--H--L--C--P--T--I--D--Q--A--C--T--C--L--V--A--S--C--S--K--A--C--N--P--E--F--R--V--R--D--Q--G--R--*--G--S--N--K--G--S--L--E--K--V--S--S--L--I--I--R--V--*--D--G--H--*--L--H--S--G--Y--C--L--S--K--L--I--V--F--H--F--V--G--R--K--L--G--S--G--*--D--*--Q--E--D--*--G--I--E--*--Q--I--F--I--I--I--F--I--*--E--K--R--Y--F--E--K--L--S--K--F--I--S--S--T--I--E--E--K--L--H--N--I--*--L--Y--P--*--*--S--K--A--F--L--V--W--K--D--D--F--G--K--T--P--V--H--K--I--N--C--T--F--L--G--T--K--V--V--N--A--Q--E--D--*--Q--L--G--Q--Y--R--S--W--Y--K--I--C--R--R--S--F--T--K--V--V--V--V--N--S--G--M--R--E--V--I--Q--V--T--L--Q--R--H--I--V--S--N--S--M--*--N--L--F--K--R--N--K--R--Q--H--M--*--F--L--S--R--Q--I--Q--L--F--W--V--K--*--T--E--L--S--R--I--*--I--M--N--W--V--G--L--I--F--H--R--R--K--D--M--C--T--L--K--I--K--L--G--G--N--Y--S--S--L--G--E--I--G--Q--M--T--R--E--C--L--*--D--R--R--*--R--D--*--M--T--I--L--V--*--L--K--N--K--H--L--G--K--L--*--E--E--I--N--I--N--L--M--F--L--L--L--R--N--A--E--T--R--L--F--V--Q--*--L--V--C--T--H--M--F--K--I--V--K--E--W--A--C--I--L--Q--M--V--F--F--F--E--L--F--*--R--M--V--E--S--*--V--I--E--*--H--I--Q--*--C--M--K--D--*--F--G--I--L--L--C--*--E--Y--M--R--V--R--L--L--I--V--L--E--S--G--V--Q--*--L--W--L--Y--L--V--R--L--P--M--*--D--K--L--*--T--M--W--D--G--R--E--R--E--Q--Q--N--I--I--V--D--C--Q--L--*--W--N--L--I--I--L--Q--V--D--*--Q--T--V--Q--V--N--V--I--L--W--K--I--S--S--N--S--M--G--I--L--I--F--Y--K--K--L--F--I--N--D--K--A--F--K--M--S--I--Q--L--R--E--*--D--R--S--F--V--F--E--F--A--V--D--*--L--S--Q--V--*--Y--V--T--A--K--V--M--T--S--S--F--S--K--M--Y--Q--*--Y--*--I--L--R--M--S--T--V--D--G--I--D--G--S--E--Y--P--*--V--Y--P--I--H--P--T--L--V--Y--V--L--V--L--G--S--*--*--*--L--H--F--P--I--H--E--K--N--*--S--T--N--V--Y--T--W--D--R--Y--R--C--N--V--H--L--Y--F--I--N--L--N--F--A--I--S--Y--S--L--Q--T--Y--M--L--Y--I--*--C--S--N--A--T--F--Y--M--*--*--*--N--I--P--M--S--G--I--R--R--T--S--R--K--R--I--A--K--I--S--M--K--H--V--K--S--P--P--T--T--A--S--D--T--G--N--A--N--S--D--P--I--S--*--T--N--G--K--E--P--L--I--N--I--P--P--M--I--A--D--M--*--I--S--T--A--R--N--H--I--L--L--N--F--Y--H--L--L--H--Q--M--*--Q--S--F--Y--I--F--I--K--Y--S--T--K--I--R--*--G--K--F--S--T--T--A--S--V--H--*--F--*--I--M--V--S--S--K--*--Q--V--V--I--I--S--L--G--N--G--N--S--I--F--P--K--T--Y--*--L--I--K--R--M--I--T--L--T--L--K--F--I--N--L--K--Q--*--Y--L--*--R--*--V--*--F--Q--S--S--*--E--K--T--T--H--S--L--G--L--L--R--H--S--S--R--L--K--R--I--*--C--V--S--K--I--Y--L--K--I--C--N--K--I--*--Y--R--*--L--S--Q--L--F--M--N--C--H--S--T--R--*--T--*--L--Q--K--M--F--V--S--K--I--P--P--N--K--K--S--R--H--A--S--S--C--C--H--V--E--S--*--P--S--D--F--T--L--K--I--L--W--R--K--H--S--I--S--H--I--L--V--*--V--D--R--L--I--F--F--A--*--S--S--R--S--I--N--C--L--*--T--V--F--M--L--*--D--F--S--M--H--V--I--C--*--T--L--T--T----   1               element1_Cgig_270063446      2       -D--K--D--G--T--H--I--Q--Q--V--K--D--K--I--*--V--L--C--T--Q--S--Q--S--S--E--A--L--I--Y--*--V--*--M--N--Y--L--G--*--L--E--K--*--T--M--R--T--W--M--C--M--K--N--C--I--E--N--V--H--V--Q--Y--S--S--R--V--S--R--H--P--P--C--K--K--H--G--G--K--R--S--V--S--H--R--V--H--G--V--T--P--C--M--C--I--G--K--F--K--K--K--H--R--V--A--R--W--P--P--V--*--L--R--G--H--C--V--T--P--W--I--L--L--P--E--T--L--I--E--I--A--Y--V--*--K--*--I--N--Y--G--*--T--R--V--E--T--Y--I--S--F--S--Y--L--K--K--K--*--I--S--T--*--V--A--R--T--Q--K--I--V--G--A--V--N--V--K--L--F--F--C--D--L--K--T--R--R--T--W--V--P--F--N--S--F--V--L--K--*--I--E--I--N--S--P--N--L--F--N--N--Q--L--I--Y--N--Q--N--S--N--Q--F--N--S--S--C--C--L--F--L--N--T--L--D--V--V--T--D--D--P--I--K--I--S--G--A--*--T--S--Y--F--L--L--Y--L--K--I--L--S--D--L--*--I--N--L--Q--P--Y--F--K--P--I--K--N--E--K--*--K--H--F--R--S--C--F--F--L--K--H--V--D--V--L--*--*--I--T--T--P--Y--Y--R--C--L--E--*--F--*--L--Q--L--F--M--*--Q--*--I--N--K--K--S--L--L--Y--F--R--L--*--I--N--T--C--T--Y--S--C--*--R--N--I--E--A--L--Y--M--Y--*--T--Y--T--R--I--S--*--N--N--F--*--L--Y--F--R--T--A--G--N--G--A--V--I--S--H--E--K--I--T--R--A--I--H--L--Y--G--S--*--S--D--T--Q--H--C--L--S--S--W--V--L--Y--T--T--G--V--I--V--C--L--V--R--C--H--F--N--L--Q--L--T--L--C--I--F--G--R--L--N--R--C--T--R--D--A--V--I--H--T--F--P--R--T--P--V--W--*--L--I--T--T--R--R--V--Y--G--L--N--I--F--Y--F--Y--F--V--S--S--M--I--F--P--N--L--*--Q--T--K--Q--I--V--C--R--C--V--H--K--L--L--H--V--R--L--S--A--L--G--S--G--E--Q--F--R--I--Q--Q--L--Y--K--C--G--N--F--T--K--Y--*--L--A--I--R--N--Y--L--P--G--T--H--T--C--M--Y--E--K--T--D--*--L--K--I--*--M--R--*--Y--R--*--L--F--A--S--I--Y--N--K--H--N--F--I--Y--F--I--T--N--L--I--C--V--M--*--N--S--V--E--I--L--T--K--N--L--I--T--S--Y--T--L--Q--T--S--S--H--C--V--E--I--V--Y--*--N--H--G--S--K--I--N--E--L--N--S--Y--K--*--N--S--Y--N--S--K--M--I--A--Q--K--T--K--V--Q--R--E--R--E--R--E--R--D--F--T--A--Q--Y--S--N--P--H--T--C--S--I--R--M--K--K--L--N--I--S--N--T--K--Y--F--I--V--K--H--F--S--I--L--R--T--*--K--K--T--I--E--R--L--L--F--L--I--M--E--G--M--E--G--A--R--G--L--S--R--G--L--I--*--L--T--G--I--A--R--G--L--*--L--H--S--F--Y--R--N--F--R--E--K--V--S--I--*--G--E--L--*--I--*--L--I--T--L--N--N--R--*--N--V--L--K--I--K--L--I--L--Y--Q--Y--S--L--S--L--S--L--S--L--S--L--S--L--S--L--S--L--S--L--S--L--S--L--S--L--S--L--S--L--S--L--S--L--S--L--I--W--R--S--L--N--N--Q--T--V--I--I--A--I--L--S--S--F--L--E--D--K--*--F--L--N--S--S--C--L--*--R--*--N--K--N--L--S--S--T--D--P--R--I--Q--S--A--M--N--M--Y--R--A--V--L--H--G--Q--V--H--H--T--Y--V--*--N--K--E--N--L--K--I--*--I--*--S--C--I--I--I--L--F--R--E--S--T--V--V--F--N--N--L--L--D--L--L--S--L--N--R--V--*--V--R--E--D--H--I--*--F--Y--A--L--L--K--L--P--I--Y--S--N--Y--D--Y--Y--Y--V--*--K--R--T--E--T--K--T--K--T--Y--G--T--V--Y--L--N--F--K--K--E--N--K--V--L--V--F--Y--L--I--K--P--L--C--G--R--Y--R--F--F--C--S--*--Y--Q--Q--K--S--T--V--T--I--A--Y--V--*--K--L--R--A--L--K--F--Y--L--F--I--S--I--S--L--S--F--S--L--I--L--L--R--T--S--I--N--I--L--F--F--I--S--F--I--P--K--S--S--F--L--S--Q--V--N--T--A--C--Y--T--V--L--L--S--V--L--H--R--V--A--P--C--R--T--V--F--F--L--V--P--W--P--*--Q--R--T--I--D--Q--R--Y--R--H--F--H--A--Q--R--G--L--S--N--T--V--V--G--V--L--V--G--I--L--V--S--C--T--V--V--G--C--R--A--R--L--T--L--E--M--F--C--I--T--V--L--G--G--K--G--A--G--T--V--T--K--A--I--N--K--C--L--K--E--S--G--V--S--E--V--Y--H--G--L--H--S--F--A--F--W--M--E--G--L--T--I--Y--L--S--N--C--W--N--D--N--Y--I--F--S--*--K--I--E--C--N--N--N--S--V--Y--*--*--P--F--G--D--S--*--P--I--R--R--H--Y--*--L--G--S--K--R--T--L--F--L--G--D--I--C--S--T--S--N--F--N--P--F--R--L--H--S--I--T--*--K--I--K--Y--W--R--F--L--D--S--W--K--G--Y--A--K--W--R--S--E--S--H--K--Q--E--N--T--S--I--S--T--D--A--S--G--Y--K--Y--G--A--V--V--C--F--Q--G--E--K--V--E--L--G--D--F--L--E--T--N--D--S--R--P--I--H--L--K--E--A--E--A--V--L--Q--V--L--K--S--I--E--E--V--I--R--D--S--R--V--D--L--L--V--D--N--V--A--V--L--S--V--W--N--N--Q--G--G--R--D--R--S--L--N--N--I--T--K--Q--I--F--Q--L--V--T--S--C--N--C--D--L--H--M--Q--Y--V--P--S--E--*--M--G--Q--I--Y--L--F--E--C--M--F--E--Y--W--F--M--A--A--C--*--K--A--I--W--T--S--F--N--G--P--H--G--Y--*--F--*--C--D--E--I--F--R--R--Y--S--T--T--K--F--N--P--Y--P--T--P--Y--S--S--C--V--N--L--F--A--Q--D--L--P--V--V--V--N--A--Y--V--Y--P--P--F--A--L--I--H--P--V--L--L--F--L--R--E--Q--K--V--G--M--C--T--F--V--L--P--L--I--K--P--V--P--V--W--W--P--L--V--Q--K--H--V--I--Q--S--L--E--L--G--T--R--G--D--K--A--V--I--R--V--P--*--K--R--F--H--H--*--*--*--G--S--E--M--A--T--S--C--I--Q--A--I--V--Y--L--S--*--L--F--F--I--L--*--G--E--S--L--D--L--V--K--I--D--K--R--I--K--E--L--N--D--K--S--S--S--S--S--L--S--K--R--R--D--T--L--K--N--S--L--N--L--F--L--P--L--L--R--K--N--C--T--I--S--N--C--T--P--D--D--L--K--H--F--W--Y--G--R--M--I--L--V--K--H--Q--F--I--K--*--I--V--H--F--W--V--L--K--L--*--M--P--K--R--I--S--S--W--D--S--I--G--H--G--I--K--F--V--G--D--L--L--R--K--W--S--W--*--I--L--G--*--E--K--*--Y--R--*--P--C--S--G--T--L--Y--Q--T--V--C--E--T--Y--S--R--G--T--S--G--S--T--C--S--F--*--A--G--K--S--N--F--S--G--*--N--K--Q--N--C--L--V--Y--R--*--*--I--G--*--G--*--Y--F--I--E--G--K--I--C--A--P--*--R--S--S--L--E--E--I--T--V--L--W--G--K--S--G--K--*--L--G--N--A--C--R--T--G--G--K--E--I--R--*--Q--F--W--F--S--*--K--T--N--I--W--E--N--F--K--R--K--*--T--*--I--S--C--F--C--Y--*--E--M--Q--R--Q--D--C--L--S--S--N--W--F--A--H--I--C--S--R--L--*--K--N--G--R--V--S--C--K--W--F--S--F--S--S--C--F--R--E--W--*--S--H--R--*--*--S--D--I--F--S--N--V--*--K--T--D--S--V--S--Y--F--V--R--N--I--*--G--*--D--S--S--*--F--*--S--R--V--C--S--D--S--G--F--I--W--F--G--C--Q--C--R--T--N--Y--E--P--C--G--M--V--G--R--G--N--S--R--I--L--*--*--I--A--S--F--S--G--I--*--L--Y--C--R--*--I--S--R--Q--C--R--S--M--*--F--C--G--R--S--V--P--T--V--W--G--F--*--Y--F--T--K--S--F--S--*--T--I--R--P--S--K--C--Q--Y--N--*--G--N--K--T--D--P--L--F--S--N--L--Q--*--I--N--Y--P--K--S--N--T--S--P--R--K--*--*--R--H--L--S--Q--R--C--T--N--D--T--E--Y--*--G--*--A--R--*--M--A--L--M--V--Q--S--I--H--E--F--T--P--S--T--P--P--L--Y--M--F--L--F--S--V--H--D--N--D--Y--I--S--Q--Y--M--R--R--T--R--A--R--T--Y--T--P--G--I--G--I--G--A--M--S--I--S--I--S--S--I--*--I--L--Q--F--L--T--L--C--R--R--I--C--C--T--F--D--V--Q--M--Q--H--F--T--C--N--D--K--T--Y--L--C--L--G--S--E--E--P--V--G--K--E--L--R--K--S--P--*--N--M--S--R--V--P--Q--L--L--P--P--T--R--G--M--P--T--Q--I--P--S--R--K--P--T--E--K--S--R--*--L--T--F--L--R--*--*--L--I--C--R--F--Q--L--Q--E--I--I--F--C--*--I--F--T--I--F--Y--I--K--C--D--K--V--S--I--F--L--*--N--T--L--L--K--Y--D--K--V--N--F--Q--Q--P--R--L--Y--T--N--S--K--S--W--Y--H--L--N--N--K--W--*--S--Y--H--W--E--T--E--I--Q--S--F--Q--K--R--I--D--*--*--R--E--*--*--H--L--L--L--N--S--S--I--*--N--N--D--T--F--R--D--R--Y--D--S--R--A--H--E--R--K--P--H--I--A--*--D--C--S--A--I--H--Q--D--*--K--E--F--S--V--S--Q--K--Y--I--*--K--S--A--T--K--F--N--T--D--N--Y--H--S--Y--L--*--I--V--I--L--P--D--K--H--D--Y--K--R--C--S--C--P--K--Y--L--P--I--R--S--P--D--T--P--P--A--A--V--M--*--N--P--N--H--P--T--S--L--L--R--Y--Y--G--E--N--T--A--F--R--I--F--*--S--K--W--T--D--*--F--S--L--L--E--V--Q--G--P--*--T--A--Y--K--L--S--L--C--F--K--I--S--V--C--M--*--F--A--K--P--*--L--Q---   2               element1_Cgig_270063446      3       --T--R--M--V--H--I--F--S--R--*--R--T--R--F--K--F--S--V--P--S--P--S--P--V--K--H--*--F--I--K--F--R--*--I--I--*--V--S--*--K--S--E--P--*--G--H--G--C--V--*--R--T--V--*--K--M--Y--M--Y--S--T--A--H--E--Y--P--D--T--H--R--V--K--N--T--V--E--N--G--L--C--R--T--V--Y--T--V--*--H--R--V--C--V--L--E--N--S--R--K--S--T--V--S--H--G--G--R--P--C--N--S--V--A--T--V--L--L--R--G--Y--C--Y--L--R--R--*--*--K--*--R--M--F--R--N--K--*--I--M--A--K--Q--G--L--K--H--I--Y--P--F--H--I--*--K--R--N--E--Y--R--L--K--L--H--G--P--R--K--L--S--G--L--S--T--*--S--Y--F--F--V--I--*--K--L--D--V--H--G--Y--L--L--I--H--L--F--L--N--K--L--K--*--I--H--Q--I--Y--L--I--I--N--*--Y--I--I--K--I--Q--I--N--L--I--H--R--V--V--C--F--*--T--R--L--M--L--*--L--T--I--R--L--K--Y--R--E--L--K--R--L--I--F--C--Y--I--*--K--Y--F--L--I--Y--K--L--I--Y--S--L--T--L--N--Q--S--K--M--R--N--E--N--I--S--D--H--V--F--F--*--N--M--L--M--C--C--S--K--*--Q--P--H--I--T--G--V--S--N--N--F--D--F--S--Y--L--C--S--N--K--*--T--K--N--L--F--Y--I--L--D--Y--R--*--I--H--V--H--T--L--V--K--E--I--L--K--H--C--T--C--T--K--H--I--P--A--F--H--K--I--T--F--N--F--I--S--V--Q--L--V--M--A--P--*--F--H--M--K--K--S--L--V--P--Y--T--C--T--E--A--D--Q--I--H--N--I--V--F--H--L--G--F--Y--T--Q--Q--V--L--L--S--V--L--L--G--V--I--L--I--Y--N--*--L--C--V--Y--L--D--V--*--T--G--V--L--E--M--P--L--F--T--P--F--H--G--H--L--F--G--N--S--S--Q--P--V--V--C--M--V--*--I--Y--F--T--S--T--L--L--V--P--*--F--F--L--I--S--N--K--Q--N--K--L--S--V--D--V--Y--T--N--Y--Y--T--*--D--Y--R--R--L--D--P--E--N--N--S--E--F--S--N--S--I--N--A--G--I--S--Q--S--I--N--L--R--*--E--I--I--Y--R--V--H--I--H--V--C--T--K--K--L--I--D--*--K--Y--E--*--D--N--T--G--N--F--L--Q--A--F--I--T--N--T--T--L--F--T--L--*--P--I--*--Y--V--*--C--E--I--A--S--K--F--*--R--K--I--L--L--H--P--I--L--Y--K--L--Q--V--I--V--L--K--S--F--I--K--T--M--D--L--K--*--M--N--*--I--H--T--N--R--T--L--I--I--L--K--*--L--H--K--R--Q--K--Y--R--E--R--E--R--E--R--E--I--L--Q--R--N--I--P--T--L--I--H--A--V--*--E--*--K--N--*--T--F--Q--T--L--N--T--S--L--L--N--I--F--L--F--C--G--L--K--K--K--Q--*--N--D--F--Y--S--L--S--W--K--A--W--K--E--H--V--V--Y--L--A--G--*--F--D--*--Q--G--*--H--V--D--S--D--C--I--A--S--I--A--I--L--G--K--R--F--Q--Y--K--G--N--Y--K--S--N--L--L--H--*--I--T--G--E--M--Y--L--K--L--N--S--Y--C--T--S--I--L--S--L--S--L--S--L--S--L--S--L--S--L--S--L--S--L--S--L--S--L--S--L--S--L--S--L--S--L--S--L--S--L--S--Y--G--D--H--*--T--I--K--Q--*--L--L--Q--Y--*--V--V--S--W--K--T--N--N--F--*--T--Q--V--A--Y--K--D--E--I--K--T--Y--Q--V--Q--I--H--G--F--K--A--L--*--I--C--T--V--R--Y--Y--M--D--K--Y--I--T--H--M--Y--K--I--K--K--I--*--K--Y--E--Y--K--A--V--*--L--Y--Y--L--E--K--V--Q--L--F--S--T--T--C--*--I--C--Y--R--*--I--E--Y--K--*--E--K--I--T--Y--D--S--M--H--Y--*--N--C--Q--S--T--A--T--T--I--T--T--T--F--K--N--G--L--K--Q--K--Q--K--L--M--E--Q--C--I--*--I--L--K--R--K--I--K--C--*--C--F--I--*--S--N--L--Y--V--G--D--I--D--F--F--V--L--N--I--N--K--N--Q--P--*--Q--S--H--T--S--E--N--C--V--P--*--N--F--I--Y--L--F--L--F--L--C--L--F--L--*--F--S--Y--V--H--Q--*--I--F--Y--S--L--*--V--L--F--L--N--P--P--F--Y--H--R--*--I--L--R--V--T--P--C--Y--C--P--C--C--I--A--L--H--R--V--A--P--C--F--F--S--F--R--G--R--N--R--E--Q--*--I--K--G--T--D--T--S--T--L--S--V--D--F--Q--T--R--W--S--V--F--L--S--G--Y--S--*--A--V--L--*--*--D--A--E--Q--D--*--H--W--K--C--F--V--*--R--C--L--G--G--K--V--Q--V--Q--*--L--R--P--*--I--N--V--*--R--R--V--G--F--L--K--F--I--M--V--Y--T--V--L--P--F--G--W--K--A--S--P--Y--I--Y--Q--T--V--G--M--T--I--T--Y--F--L--R--K--L--N--V--I--T--T--Q--Y--I--D--D--R--L--V--I--A--N--P--S--E--G--T--T--D--L--E--A--K--E--H--C--F--W--V--I--Y--A--L--L--Q--I--L--I--R--L--G--Y--T--V--S--L--E--K--S--N--T--G--V--S--*--T--R--G--R--V--T--Q--S--G--G--Q--R--V--I--S--K--K--I--P--V--F--Q--Q--M--L--L--A--T--S--M--G--Q--*--F--V--F--R--G--K--R--L--N--L--E--I--F--W--R--Q--T--T--V--D--L--F--I--*--R--K--L--R--Q--S--F--R--S--*--S--Q--*--R--K--*--L--E--I--Q--G--L--T--Y--W--*--I--M--L--L--F--L--V--F--G--T--I--K--E--D--G--I--D--L--*--T--T--*--Q--N--R--Y--F--S--W--*--L--L--V--I--V--I--C--T--C--N--M--Y--P--Q--S--E--W--G--R--F--T--C--L--N--A--C--L--N--I--G--S--W--Q--R--V--E--R--Q--F--G--P--H--S--M--D--L--M--A--I--D--S--N--V--M--K--S--S--G--G--T--A--L--P--N--L--T--H--I--L--P--H--I--H--L--V--*--I--C--L--L--R--I--C--Q--W--W--L--M--H--M--F--I--L--L--L--P--L--Y--I--L--Y--S--Y--F--*--G--N--R--K--L--V--C--A--P--L--S--Y--H--*--S--S--L--Y--L--F--G--G--L--L--F--K--S--M--*--S--R--V--*--S--*--G--P--G--E--I--R--Q--*--*--G--F--L--R--K--G--F--I--T--D--N--K--G--L--R--W--P--L--V--A--F--R--L--L--F--I--*--A--N--C--F--S--F--C--R--E--K--A--W--I--W--L--R--L--T--R--G--L--R--N--*--M--T--N--L--H--H--H--L--Y--L--R--E--E--I--L--*--K--T--L--*--I--Y--F--F--H--Y--*--G--K--I--A--Q--Y--L--I--V--P--L--M--I--*--S--I--S--G--M--E--G--*--F--W--*--N--T--S--S--*--N--K--L--Y--I--F--G--Y--*--S--C--E--C--P--R--G--L--A--A--G--T--V--S--V--M--V--*--N--L--S--E--I--F--Y--E--S--G--R--G--K--F--W--D--E--R--S--N--T--G--N--P--A--A--A--H--C--I--K--Q--Y--V--K--L--I--Q--E--E--Q--A--A--A--H--V--V--S--K--Q--A--N--P--T--F--L--G--K--I--N--R--I--V--S--Y--I--D--N--E--L--G--R--V--D--I--S--S--K--E--R--Y--V--H--L--K--D--Q--A--W--R--K--L--Q--F--F--G--G--N--R--A--N--D--*--G--M--L--V--G--Q--E--V--K--R--L--D--D--N--S--G--L--V--K--K--Q--T--F--G--K--T--L--R--G--N--K--H--K--S--H--V--F--V--I--K--K--C--R--D--K--T--V--C--P--V--T--G--L--H--T--Y--V--Q--D--C--K--R--M--G--V--Y--L--A--N--G--F--L--F--R--V--V--L--E--N--G--R--V--I--G--D--R--V--T--Y--S--V--M--Y--E--R--L--I--R--Y--L--T--L--L--G--I--Y--E--G--E--T--P--H--S--F--R--V--G--C--A--V--T--L--A--L--S--G--S--V--A--N--V--G--Q--I--M--N--H--V--G--W--*--G--E--G--T--A--E--Y--Y--S--R--L--P--A--L--V--E--S--D--Y--I--A--G--R--L--A--D--S--A--G--Q--C--D--F--V--E--D--Q--F--Q--Q--Y--G--D--F--N--I--L--Q--K--A--F--H--K--R--*--G--L--Q--N--V--N--T--I--K--G--I--R--Q--I--L--C--F--R--I--C--S--R--L--I--I--P--S--L--I--R--H--R--E--S--D--D--V--I--F--L--K--D--V--P--M--I--L--N--I--E--D--E--H--G--R--W--H--*--W--F--R--V--S--M--S--L--P--H--P--P--H--P--C--I--C--S--C--S--R--F--M--I--M--T--T--F--P--N--T--*--E--E--L--E--H--E--R--I--H--L--G--*--V--*--V--Q--C--P--S--L--F--H--Q--F--K--F--C--N--F--L--L--F--A--D--V--Y--V--V--H--L--M--F--K--C--N--I--L--H--V--M--I--K--H--T--Y--V--W--D--Q--K--N--Q--*--E--K--N--C--E--N--L--H--E--T--C--Q--E--S--P--N--Y--C--L--R--H--G--E--C--Q--L--R--S--H--L--V--N--Q--R--K--R--A--V--D--*--H--S--S--D--D--S--*--Y--V--D--F--N--C--K--K--S--Y--F--V--E--F--L--P--S--F--T--S--N--V--T--K--F--L--Y--F--Y--K--I--L--Y--*--N--T--I--R--*--I--F--N--N--R--V--C--T--L--I--L--N--H--G--I--I--*--I--T--S--G--D--H--I--I--G--K--R--K--F--N--L--S--K--N--V--L--I--N--K--E--N--D--N--T--Y--S--*--I--H--Q--F--E--T--M--I--P--L--E--I--G--M--I--P--E--L--M--R--E--N--H--T--*--P--R--T--A--P--P--F--I--K--I--K--K--N--L--V--C--L--K--N--I--S--E--N--L--Q--Q--N--L--I--Q--I--T--I--T--A--I--Y--E--L--S--F--Y--P--I--N--M--I--T--K--D--V--R--V--Q--N--T--S--Q--*--E--V--Q--T--R--L--Q--L--L--S--C--R--I--L--T--I--R--L--H--S--*--D--I--M--E--K--T--Q--H--F--A--Y--F--S--L--S--G--Q--I--N--F--L--C--L--K--F--K--V--H--K--L--L--I--N--C--L--Y--A--L--R--F--Q--Y--A--C--N--L--L--N--P--D--Y--N--   3                                                                                                                                                                                                                                                                                                                                                                                                                                                                                                                                                                                                                                                                                                                                                                                                                                                                                                                                                                                                                                                                                                                                                                                                                                                                                                                                                                                                                                                                                                                                                                                                                                                                                                                                                                                                                                                                                                                                                                                                                                                                                                                         <--frame 3 fragmented RT-----------------------------------------------------------------------------------------------------------------------------------------------------------------------------------------------------------------------------------------------------------<--frame 2 RT------->------------------------------------RT ends---->< RNaseH continues frame 2-----------------------------------------------------------------------------------------------------------------------------------------------------------------------------------------------------------------------------------------------------------------------------------------------------------------------------------------------------------------------<--DNA methylase continues in same frame-2with stop codons------------------------------------------------------------------------------------------------------------------------------------------------------------------------------------------------------------------------------------------------------------------------------------------>                                                                                                                                                                                                                                                              <--ALOG in frame 1---------------------------------------------------------------<-continues in frame 3--------------------------------------------------------------------------------------------------------------->               gi|270063462|gb|GU207455.1|     .       ---------:---------:---------:------6200---------:---------:---------:---------:------6150---------:---------:---------:---------:------6100---------:---------:---------:---------:------6050---------:---------:---------:---------:------6000---------:---------:---------:---------:------5950---------:---------:---------:---------:------5900---------:---------:---------:---------:------5850---------:---------:---------:---------:------5800---------:---------:---------:---------:------5750---------:---------:---------:---------:------5700---------:---------:---------:---------:------5650---------:---------:---------:---------:------5600---------:---------:---------:---------:------5550---------:---------:---------:---------:------5500---------:---------:---------:---------:------5450---------:---------:---------:---------:------5400---------:---------:---------:---------:------5350---------:---------:---------:---------:------5300---------:---------:---------:---------:------5250---------:---------:---------:---------:------5200---------:---------:---------:---------:------5150---------:---------:---------:---------:------5100---------:---------:---------:---------:------5050---------:---------:---------:---------:------5000---------:---------:---------:---------:------4950---------:---------:---------:---------:------4900---------:---------:---------:---------:------4850---------:---------:---------:---------:------4800---------:---------:---------:---------:------4750---------:---------:---------:---------:------4700---------:---------:---------:---------:------4650---------:---------:---------:---------:------4600---------:---------:---------:---------:------4550---------:---------:---------:---------:------4500---------:---------:---------:---------:------4450---------:---------:---------:---------:------4400---------:---------:---------:---------:------4350---------:---------:---------:---------:------4300---------:---------:---------:---------:------4250---------:---------:---------:---------:------4200---------:---------:---------:---------:------4150---------:---------:---------:---------:------4100---------:---------:---------:---------:------4050---------:---------:---------:---------:------4000---------:---------:---------:---------:------3950---------:---------:---------:---------:------3900---------:---------:---------:---------:------3850---------:---------:---------:---------:------3800---------:---------:---------:---------:------3750---------:---------:---------:---------:------3700---------:---------:---------:---------:------3650---------:---------:---------:---------:------3600---------:---------:---------:---------:------3550---------:---------:---------:---------:------3500---------:---------:---------:---------:------3450---------:---------:---------:---------:------3400---------:---------:---------:---------:------3350---------:---------:---------:---------:------3300---------:---------:---------:---------:------3250---------:---------:---------:---------:------3200---------:---------:---------:---------:------3150---------:---------:---------:---------:------3100---------:---------:---------:---------:------3050---------:---------:---------:---------:------3000---------:---------:---------:---------:------2950---------:---------:---------:---------:------2900---------:---------:---------:---------:------2850---------:---------:---------:---------:------2800---------:---------:---------:---------:------2750---------:---------:---------:---------:------2700---------:---------:---------:---------:------2650---------:---------:---------:---------:------2600---------:---------:---------:---------:------2550---------:---------:---------:---------:------2500---------:---------:---------:---------:------2450---------:---------:---------:---------:------2400---------:---------:---------:---------:------2350---------:---------:---------:---------:------2300---------:---------:---------:---------:------2250---------:---------:---------:---------:------2200---------:---------:---------:---------:------2150---------:---------:---------:---------:------2100---------:---------:---------:---------:------2050---------:---------:---------:---------:------2000---------:---------:---------:---------:------1950---------:---------:---------:---------:------1900---------:---------:---------:---------:------1850---------:---------:---------:---------:------1800---------:---------:---------:---------:------1750---------:---------:---------:---------:------1700---------:---------:---------:---------:------1650---------:---------:---------:---------:------1600---------:---------:---------:---------:------1550---------:---------:---------:---------:------1500---------:---------:---------:---------:------1450---------:---------:---------:---------:------1400---------:---------:---------:---------:------1350---------:---------:---------:---------:------1300---------:---------:---------:---------:------1250---------:---------:---------:---------:------1200---------:---------:---------:---------:------1150---------:---------:---------:---------:------1100---------:---------:---------:---------:------1050---------:---------:---------:---------:------1000---------:---------:---------:---------:-------950---------:---------:---------:---------:-------900---------:---------:---------:---------:-------850---------:---------:---------:---------:-------800---------:---------:---------:---------:-------750---------:---------:---------:---------:-------700---------:---------:---------:---------:-------650---------:---------:---------:---------:-------600---------:---------:---------:---------:-------550---------:---------:---------:---------:-------500---------:---------:---------:---------:-------450---------:---------:---------:---------:-------400---------:---------:---------:---------:-------350---------:---------:---------:---------:-------300---------:---------:---------:---------:-------250---------:---------:---------:---------:-------200---------:---------:---------:---------:-------150---------:---------:---------:---------:-------100---------:---------:---------:---------:--------50---------:---------:---------:---------:--------1.               gi|270063462|gb|GU207455.1|     6239    TTAAACAACAAATATGAATTGCATTTTTGATATTATCTATCATAAAACAATATACTTCTATAGATTTGGTCATGATAAATTGTAGAATTGTACTTATTAACAGATTTAATTCTTAATTAGCTTAGAATGAGTCTTTATTTTGTCGCAACTACTATGTTTGGTAAAGTCATCATAAAGTATTTGCACTTTAGCACATAGAAAGCATATTATATACAAATAATATTTCTGAAGGATTTAGCATGTATCCCAAGTGTAATACGTCACTGCGAAAGTGATGACGTCATCTTTCTCAGAGCTACACCAAGGAGGAAGACCTATTGAGAAGCGGCAATGTAATATATTTTCTTATATTAGACAATAGAGCGGTCTGTTGCCGATGAGGGCATTGCCGATGAGTGTTTTTATATGGCCAGTGGATCTACAGTGATATATCAATCGCTGAGGATTGATTTTGGAGTGAAATTTTAAAAATGATGCGGCGTCGATGAGGACGACCACATGGTATGGCTGGTACTGGATGTACCATGAGGGCCAGATATACATAATCAGCGAGGTGGTAATAGCTTGTTTACCGCTGATGTTAGATTAATATTGGTGATGTGATTAAATGGTATGATTTTACTGAGACTGACGATGGATGATTGCACTGATTTAACATGGATTTTATGTAGGTCGAAAATCGCAGATAAAACGTATGCAACGTTTTAAAAAAAAATGTACCGTCAAGAGTAAATAATAACAAGCATACGTGTGATGTGAATCAATTAAACCTTTATGATTTTTCAATTTTGTGGCCGGAATTAGTAGATATGTACGGATCTATTTGTATATTAGCTTTTTTTACTTTCGGCTTGATGTTGTAGATGGTGTTACCTACTGTTTTAAAAAAAAACATTCCCTGAAAGGAAAACAATTATTACCAGTTATCAGAAAGTTGAGGGATATTCAAGATCCGCATGGCATTAAACAGTTAGCTAGGACTTCTTCTGCTAGGCGTACCGAAAATATGTCTGATGAGGATGTATTGAAGGCTGTTGAGGATTTAACTGGAGAAAACGGGATTGAGTTACTGGAACTGGTAGGTATTATTTACAATAAAACAAAGTTCTGTCATGAATGAAGAATTGATATTTATGATATTACAGTGTAATAGCTCTTAAATCTCTAATTAATAACTATAGAAGTTTAAGAACAAGTGATGCCATTTTATTGAATTAAATTCATTTAAAATAATAGGGAAGGACCCAAATAATGAGGATTTTGCAAAGTTACTACTCCACTTCTGCATATTACATTCTAATAAAAAAGGACAACCAGAAGGACAGGATTTGCATGATCCAGAATTAGGTCATTCGTCAGCAACGACTGCAGATCAAGAGAAAGGGCCTACTTCAAATTCAAGGAAGGCAACAGACAAAGATCAGGTACTCTTAGTTAACGGATTAGGTTTTTCATCAAAAGAGTGATTAATTATCTGTGATATTTAATTATTAGTAATTAATTATGTATTGTTAATAAACTGATTTCTAACCTATTAGTTTGCATTGATAGTGATTAATCATTTTGAATAAATGGTTAAACAATATTCACTTCAACCAGGTACAGTATTACTACTTGTGATGATAGTAATCATTATCAAATGATTTATGCATTTTAGGATTCAAATGGTGGAAAACGTAGTAAGCTACAAGATTCAACAACTGAAGTAAAAGACAAGGATGGTACACATATTCAGCAGGTGAAGGACAAGATTTAAGTTCTCTGTACCCAGTCCCAGCCCAGTGAAGCACTGATTTATTAAGTTTAGATGAATTATCTAGGTTAGCTAGAAAAGTGAACCATGAGGACATGGATGTGTATGAAGAACTGTATAGAAAATGTACATGTAGTAGGATGCAGAGCAAGATTAACATTGGAAATTTTTTGTATAACGGTGCTTGGGGGGAAAGGTGCAGGTACAGTGACTAAGGCCATAAATAAATGTTTAAAGGAGAGTGGGATTTCTGAAGTTTATCATGGTTTACACAGTTTTGCCTTTTGGATGGAAGGCCTCACCATATATTTATCAAACTGTTGGAATGACAATTACATATTTTCTTAGAAAATTGAATGTAATAACAACTCAGTATATTGATGACCGTTTGGTGATAGCTAACCCATCAGAAGGCACTACTGACTTGGAAGCAAAAGAACATTGTTTTTGGGTGATATATGCTCTACTTCAAATTTTAATCCGTTTAGGTTACACAGTATCACTTGAAAAATCAAATACTGGCGTTTCTTAGACTCGTGGAAGGGTTACGCAAAGTGGAGGTCAGAGAGTCATAAGCAAGAAAATACCAGTATTTCAACAGATGCTTCTGGCTACAAGTATGGGGCAGTAGTTTGTTTTCAGGGGGAAAAGGTTGAACTTGGAGATTTTTTGGAGACAAACGACAGTAGACCTATTCATTTGAAGGAAGCTGAGGCAGTCCTTCAGGTCTTGAAGTCAATAGAGGAAGTAATTAGAGATTCAAGGGTTGACTTACTGGTAGATAATGTTGCTGTTCTTAGTGTTTGGAACACTCAAGGAGGACGGGATAGATCTTTGAACAACATAACAAAACAGATATTTCAGTTGGTGACTTCTTGTAATTGTGATCTGCACATGCAATATGTACCCTCAGAGTGAATGGGGCAGATTTACCTGTTTGAATGCATGTTTGAATATTGGTTCATGGCAGCGTGTTGAAAGGCAATTTGGACCTCATTCAATGGACCTCATGGCTATTGATTCTAATGTGATGAAATATTCAGGAGGTACAGCACTACCAAATTTAACCCATATCCTACCCCATATTCATCTTGTGTAAATTTGTTTGCTCAGGATTTGCCAGTGGTGGTTAATGCATATGTTTATCCTCCTTTTGCCCTTATACATCCTGTACTCTTATTTTTAAGGGAACAGAAAGTTGGTATGTGCACCTTTGTCCTACCATTGATCAAGCCTGTACCTGTTTGGTGGCCTCTTGTTCAAAAGCATGTAATCCAGAGTTTAGAGTTAGGGACCAGGGGAGATAAGGCAGTAATAAGGGTTCCTTAGAAAAGGTTTCATCACTGATAATAAGGGTCTGAGATGGCCACTAGTTGCATTCAGGCTATTGTTTATCTAAGCTAATTGTTTTTCATTTTGTAGGGAGAAAGCTTGGATCTGGTTAAGATTGACAAGAGGATTAAGGAATTGAATGACAAATCTTCATCATCATCTTTATCTAAGAGAAGAGATACTTTGAAAAACTCTCTAAATTTATTTCTTCCACTATTGAGGAAAAATTGCACAATATCTAATTGTACCCCTGATGATCTAAAGCATTTCTGGTATGGAAGGATGATTTTGGTAAAACACCAGTTCATAAAATAAATTGTACATTTTTGGGTACTAAAGTTGTGAATGCCCAAGAGGATTAGCAGCTGGGACAGTATCGGTCATGGTATAAAATTTGTCGGAGATCTTTTACGAAAGTGGTCGTGGTAAATTCTGGGATGAGAGAAGTAATACAGGTAACCCTGCAGCGGCACATTGTATCAAACAGTATGTGAAACTTATTCAAGAGGAACAAGCGGCAGCACATGTAGTTTCTAAGCAGGCAAATCCAACTTTTCTGGGTAAAATAAACAGAATTGTCTCGTATATAGATAATGAATTGGGTAGGGTTGATATTTCATCGAAGGAAAGATATGTGCACCTTAAAGATCAAGCTTGGAGGAAATTACAGTTCTTTGGGGGAAATCGGGCAAATGACTAGGGAATGCTTGTAGGACAGGAGGTAAAGAGATTAGATGAATTCTGGTTTAGTTAAAAAACAAACATTTGGGAAAACTTTAAGAGGAAATAAACATAAATCTCATGTTTTTGTTATTAAGAAATGCAGAGACAAGACTGTTTGTCCAGTAACTGGTTTGCACACATATGTTCAAGATTGTAAAAGAATGGGCGTGTATCTTGCAAATGGTTTTCTTTTTCGAGTTGTTTTAGAGAATGGTAGAGTCATAGGTGATAGAGTGACATATTCAGTAATGTATGAAAGACTGATTCGGTATCTTACTTTGTTAGGAATATATGAGGGTGAGACTCCTCATAGTTTTAGAGTCGGGTGTGCAGTGACTCTGGCTTTATCTGGTTCGGTTGCCAATGTAGGACAAATTATGAACCATGTGGGATGGTAGGGAGAGGGAACAGCAGAATATTATAGTAGATTGCCAGCTTTAGTGGAATCTGATTATATTGCAGGTAGATTAGCAGACAGTGCAGGTCAATGTGATTTTGTGGAAGATCAGTTCCAACAGTATGGGGATTTTAATATTTTACAAAAAGCTTTTCATAAACGATAAGGCCTTCAAAATGTCAATACAATTAAGGGAATAAGACAGATCCTTTGTTTTCGAATTTGCAGTAGATTAATTATCCCAAGTCTAATACGTCACCGCGAAAGTGATGACGTCATCTTTCTCAAAGATGTACCAATGATACTGAATATTGAGGATGAGCACGGTAGATGGCATTGATGGTTCAGAGTATCCATGAGTTTACCCCATCCACCCCACCCTTGTATATGTTCTTGTTCTCGGTTCATGATAATGACTACATTTCCCAATACATGAGAAGAACTAGAGCACGAACGTATACACCTGGGATAGGTATAGGTGCAATGTCCATCTCTATTTCATCAATTTAAATTTTGCAATTTCTTACTCTTTGCAGACGTATATGTTGTACATTTGATGTTCAAATGCAACATTTTACATGTAATGATAAAACATACCTATGTCTGGGATCAGAAGAACCAGTAGGAAAAGAATTGCGAAAATCTCCATGAAACATGTCAAGAGTCCCCCAACTACTGCCTCCGACACGGGGAATGCCAACTCAGATCCCATCTCGTAAACCAACGGAAAAGAGCCGTTGATTAACATTCCTCCGATGATAGCTGATATGTAGATTTCAACTGCAAGAAATCATATTTTGTTGAATTTTTACCATCTTTTACATCAAATGTGACAAAGTTTCTATATTTTTATAAAATACTCTACTAAAATACGATAAGGTAAATTTTCAACAACCGCGTCTGTACACTAATTCTAAATCATGGTATCATCTAAATAACAAGTGGTGATCATATCATTGGGAAACGGAAATTCAATCTTTCCAAAAACGTATTGATTAATAAAGAGAATGATAACACTTACTCTTAAATTCATCAATTTGAAACAATGATACCTTTAGAGATAGGTATGATTCCAGAGCTCATGAGAGAAAACCACACATAGCCTAGGACTGCTCCGCCATTCATCAAGATTAAAAAGAATTTAGTGTGTCTCAAAAATATATCTGAAAATCTGCAACAAAATTTAATACAGATAACTATCACAGCTATTTATGAATTGTCATTCTACCCGATAAACATGATTACAAAAGATGTTCGTGTCCAAAATACCTCCCAATAAGAAGTCCAGACACGCCTCCAGCTGCTGTCATGTAGAATCCTAACCATCCGACTTCACTCTTAAGATATTATGGAGAAAACACAGCATTTCGCATATTTTAGTCTAAGTGGACAGATTAATTTTCTTTGCTTGAAGTTCAAGGTCCATAAACTGCTTATAAACTGTCTTTATGCTTTAAGATTTCAGTATGCATGTAATTTGCTAAACCCTGACTACAATTGTAATATTATTGTATTACAGAGAAAATGAATCCTTATAGACATTCGAGATCAATTTGAAAAACAAAACCCAAAAAAATAATCCTTTAACCTTTATTATATTGGTTTTTGCGAGCTAGAAGGCAATCTGGAGACATTTGTACTTACTTGATTCATACCGACTGGGTCTAATAACACATCTAATACAGCCTGCCAGCTTCCAAATACTCCATTAGGTATTGCATTGGCCAAACCCAACAATATGAGAGATTTGTTCCTTTAAATATTTTGAGAATATACTCAATTATTGAATAACACGCACAAAGATGTGTTTCCGCGAATGTTGCATTTTAAACTGCATTAGCTCAATCGTTGCTGTTTCTAAATGAATCTTTTCAACGAAACCCTGGGAATATCGAGAAATCCACATACAGTTCAAGTGCTTGGTTTGATAACAATGATACTCATACCTAGCCACACTTCCAAGAGATTCCTTGTAGTTGGTTCTCTGCACAGAAGCAGACGTACTTGGTGGTGTTGGCGGTTTGGCGGGAAGAT1               gi|270063462|gb|GU207455.1|     -1      L--N--N--K--Y--E--L--H--F--*--Y--Y--L--S--*--N--N--I--L--L--*--I--W--S--*--*--I--V--E--L--Y--L--L--T--D--L--I--L--N--*--L--R--M--S--L--Y--F--V--A--T--T--M--F--G--K--V--I--I--K--Y--L--H--F--S--T--*--K--A--Y--Y--I--Q--I--I--F--L--K--D--L--A--C--I--P--S--V--I--R--H--C--E--S--D--D--V--I--F--L--R--A--T--P--R--R--K--T--Y--*--E--A--A--M--*--Y--I--F--L--Y--*--T--I--E--R--S--V--A--D--E--G--I--A--D--E--C--F--Y--M--A--S--G--S--T--V--I--Y--Q--S--L--R--I--D--F--G--V--K--F--*--K--*--C--G--V--D--E--D--D--H--M--V--W--L--V--L--D--V--P--*--G--P--D--I--H--N--Q--R--G--G--N--S--L--F--T--A--D--V--R--L--I--L--V--M--*--L--N--G--M--I--L--L--R--L--T--M--D--D--C--T--D--L--T--W--I--L--C--R--S--K--I--A--D--K--T--Y--A--T--F--*--K--K--M--Y--R--Q--E--*--I--I--T--S--I--R--V--M--*--I--N--*--T--F--M--I--F--Q--F--C--G--R--N--*--*--I--C--T--D--L--F--V--Y--*--L--F--L--L--S--A--*--C--C--R--W--C--Y--L--L--F--*--K--K--T--F--P--E--R--K--T--I--I--T--S--Y--Q--K--V--E--G--Y--S--R--S--A--W--H--*--T--V--S--*--D--F--F--C--*--A--Y--R--K--Y--V--*--*--G--C--I--E--G--C--*--G--F--N--W--R--K--R--D--*--V--T--G--T--G--R--Y--Y--L--Q--*--N--K--V--L--S--*--M--K--N--*--Y--L--*--Y--Y--S--V--I--A--L--K--S--L--I--N--N--Y--R--S--L--R--T--S--D--A--I--L--L--N--*--I--H--L--K--*--*--G--R--T--Q--I--M--R--I--L--Q--S--Y--Y--S--T--S--A--Y--Y--I--L--I--K--K--D--N--Q--K--D--R--I--C--M--I--Q--N--*--V--I--R--Q--Q--R--L--Q--I--K--R--K--G--L--L--Q--I--Q--G--R--Q--Q--T--K--I--R--Y--S--*--L--T--D--*--V--F--H--Q--K--S--D--*--L--S--V--I--F--N--Y--*--*--L--I--M--Y--C--*--*--T--D--F--*--P--I--S--L--H--*--*--*--L--I--I--L--N--K--W--L--N--N--I--H--F--N--Q--V--Q--Y--Y--Y--L--*--*--*--*--S--L--S--N--D--L--C--I--L--G--F--K--W--W--K--T--*--*--A--T--R--F--N--N--*--S--K--R--Q--G--W--Y--T--Y--S--A--G--E--G--Q--D--L--S--S--L--Y--P--V--P--A--Q--*--S--T--D--L--L--S--L--D--E--L--S--R--L--A--R--K--V--N--H--E--D--M--D--V--Y--E--E--L--Y--R--K--C--T--C--S--R--M--Q--S--K--I--N--I--G--N--F--L--Y--N--G--A--W--G--E--R--C--R--Y--S--D--*--G--H--K--*--M--F--K--G--E--W--D--F--*--S--L--S--W--F--T--Q--F--C--L--L--D--G--R--P--H--H--I--F--I--K--L--L--E--*--Q--L--H--I--F--L--E--N--*--M--*--*--Q--L--S--I--L--M--T--V--W--*--*--L--T--H--Q--K--A--L--L--T--W--K--Q--K--N--I--V--F--G--*--Y--M--L--Y--F--K--F--*--S--V--*--V--T--Q--Y--H--L--K--N--Q--I--L--A--F--L--R--L--V--E--G--L--R--K--V--E--V--R--E--S--*--A--R--K--Y--Q--Y--F--N--R--C--F--W--L--Q--V--W--G--S--S--L--F--S--G--G--K--G--*--T--W--R--F--F--G--D--K--R--Q--*--T--Y--S--F--E--G--S--*--G--S--P--S--G--L--E--V--N--R--G--S--N--*--R--F--K--G--*--L--T--G--R--*--C--C--C--S--*--C--L--E--H--S--R--R--T--G--*--I--F--E--Q--H--N--K--T--D--I--S--V--G--D--F--L--*--L--*--S--A--H--A--I--C--T--L--R--V--N--G--A--D--L--P--V--*--M--H--V--*--I--L--V--H--G--S--V--L--K--G--N--L--D--L--I--Q--W--T--S--W--L--L--I--L--M--*--*--N--I--Q--E--V--Q--H--Y--Q--I--*--P--I--S--Y--P--I--F--I--L--C--K--F--V--C--S--G--F--A--S--G--G--*--C--I--C--L--S--S--F--C--P--Y--T--S--C--T--L--I--F--K--G--T--E--S--W--Y--V--H--L--C--P--T--I--D--Q--A--C--T--C--L--V--A--S--C--S--K--A--C--N--P--E--F--R--V--R--D--Q--G--R--*--G--S--N--K--G--S--L--E--K--V--S--S--L--I--I--R--V--*--D--G--H--*--L--H--S--G--Y--C--L--S--K--L--I--V--F--H--F--V--G--R--K--L--G--S--G--*--D--*--Q--E--D--*--G--I--E--*--Q--I--F--I--I--I--F--I--*--E--K--R--Y--F--E--K--L--S--K--F--I--S--S--T--I--E--E--K--L--H--N--I--*--L--Y--P--*--*--S--K--A--F--L--V--W--K--D--D--F--G--K--T--P--V--H--K--I--N--C--T--F--L--G--T--K--V--V--N--A--Q--E--D--*--Q--L--G--Q--Y--R--S--W--Y--K--I--C--R--R--S--F--T--K--V--V--V--V--N--S--G--M--R--E--V--I--Q--V--T--L--Q--R--H--I--V--S--N--S--M--*--N--L--F--K--R--N--K--R--Q--H--M--*--F--L--S--R--Q--I--Q--L--F--W--V--K--*--T--E--L--S--R--I--*--I--M--N--W--V--G--L--I--F--H--R--R--K--D--M--C--T--L--K--I--K--L--G--G--N--Y--S--S--L--G--E--I--G--Q--M--T--R--E--C--L--*--D--R--R--*--R--D--*--M--N--S--G--L--V--K--K--Q--T--F--G--K--T--L--R--G--N--K--H--K--S--H--V--F--V--I--K--K--C--R--D--K--T--V--C--P--V--T--G--L--H--T--Y--V--Q--D--C--K--R--M--G--V--Y--L--A--N--G--F--L--F--R--V--V--L--E--N--G--R--V--I--G--D--R--V--T--Y--S--V--M--Y--E--R--L--I--R--Y--L--T--L--L--G--I--Y--E--G--E--T--P--H--S--F--R--V--G--C--A--V--T--L--A--L--S--G--S--V--A--N--V--G--Q--I--M--N--H--V--G--W--*--G--E--G--T--A--E--Y--Y--S--R--L--P--A--L--V--E--S--D--Y--I--A--G--R--L--A--D--S--A--G--Q--C--D--F--V--E--D--Q--F--Q--Q--Y--G--D--F--N--I--L--Q--K--A--F--H--K--R--*--G--L--Q--N--V--N--T--I--K--G--I--R--Q--I--L--C--F--R--I--C--S--R--L--I--I--P--S--L--I--R--H--R--E--S--D--D--V--I--F--L--K--D--V--P--M--I--L--N--I--E--D--E--H--G--R--W--H--*--W--F--R--V--S--M--S--L--P--H--P--P--H--P--C--I--C--S--C--S--R--F--M--I--M--T--T--F--P--N--T--*--E--E--L--E--H--E--R--I--H--L--G--*--V--*--V--Q--C--P--S--L--F--H--Q--F--K--F--C--N--F--L--L--F--A--D--V--Y--V--V--H--L--M--F--K--C--N--I--L--H--V--M--I--K--H--T--Y--V--W--D--Q--K--N--Q--*--E--K--N--C--E--N--L--H--E--T--C--Q--E--S--P--N--Y--C--L--R--H--G--E--C--Q--L--R--S--H--L--V--N--Q--R--K--R--A--V--D--*--H--S--S--D--D--S--*--Y--V--D--F--N--C--K--K--S--Y--F--V--E--F--L--P--S--F--T--S--N--V--T--K--F--L--Y--F--Y--K--I--L--Y--*--N--T--I--R--*--I--F--N--N--R--V--C--T--L--I--L--N--H--G--I--I--*--I--T--S--G--D--H--I--I--G--K--R--K--F--N--L--S--K--N--V--L--I--N--K--E--N--D--N--T--Y--S--*--I--H--Q--F--E--T--M--I--P--L--E--I--G--M--I--P--E--L--M--R--E--N--H--T--*--P--R--T--A--P--P--F--I--K--I--K--K--N--L--V--C--L--K--N--I--S--E--N--L--Q--Q--N--L--I--Q--I--T--I--T--A--I--Y--E--L--S--F--Y--P--I--N--M--I--T--K--D--V--R--V--Q--N--T--S--Q--*--E--V--Q--T--R--L--Q--L--L--S--C--R--I--L--T--I--R--L--H--S--*--D--I--M--E--K--T--Q--H--F--A--Y--F--S--L--S--G--Q--I--N--F--L--C--L--K--F--K--V--H--K--L--L--I--N--C--L--Y--A--L--R--F--Q--Y--A--C--N--L--L--N--P--D--Y--N--C--N--I--I--V--L--Q--R--K--*--I--L--I--D--I--R--D--Q--F--E--K--Q--N--P--K--K--*--S--F--N--L--Y--Y--I--G--F--C--E--L--E--G--N--L--E--T--F--V--L--T--*--F--I--P--T--G--S--N--N--T--S--N--T--A--C--Q--L--P--N--T--P--L--G--I--A--L--A--K--P--N--N--M--R--D--L--F--L--*--I--F--*--E--Y--T--Q--L--L--N--N--T--H--K--D--V--F--P--R--M--L--H--F--K--L--H--*--L--N--R--C--C--F--*--M--N--L--F--N--E--T--L--G--I--S--R--N--P--H--T--V--Q--V--L--G--L--I--T--M--I--L--I--P--S--H--T--S--K--R--F--L--V--V--G--S--L--H--R--S--R--R--T--W--W--C--W--R--F--G--G--K    -1               gi|270063462|gb|GU207455.1|     -2      -*--T--T--N--M--N--C--I--F--D--I--I--Y--H--K--T--I--Y--F--Y--R--F--G--H--D--K--L--*--N--C--T--Y--*--Q--I--*--F--L--I--S--L--E--*--V--F--I--L--S--Q--L--L--C--L--V--K--S--S--*--S--I--C--T--L--A--H--R--K--H--I--I--Y--K--*--Y--F--*--R--I--*--H--V--S--Q--V--*--Y--V--T--A--K--V--M--T--S--S--F--S--E--L--H--Q--G--G--R--P--I--E--K--R--Q--C--N--I--F--S--Y--I--R--Q--*--S--G--L--L--P--M--R--A--L--P--M--S--V--F--I--W--P--V--D--L--Q--*--Y--I--N--R--*--G--L--I--L--E--*--N--F--K--N--D--A--A--S--M--R--T--T--T--W--Y--G--W--Y--W--M--Y--H--E--G--Q--I--Y--I--I--S--E--V--V--I--A--C--L--P--L--M--L--D--*--Y--W--*--C--D--*--M--V--*--F--Y--*--D--*--R--W--M--I--A--L--I--*--H--G--F--Y--V--G--R--K--S--Q--I--K--R--M--Q--R--F--K--K--K--C--T--V--K--S--K--*--*--Q--A--Y--V--*--C--E--S--I--K--P--L--*--F--F--N--F--V--A--G--I--S--R--Y--V--R--I--Y--L--Y--I--S--F--F--Y--F--R--L--D--V--V--D--G--V--T--Y--C--F--K--K--K--H--S--L--K--G--K--Q--L--L--P--V--I--R--K--L--R--D--I--Q--D--P--H--G--I--K--Q--L--A--R--T--S--S--A--R--R--T--E--N--M--S--D--E--D--V--L--K--A--V--E--D--L--T--G--E--N--G--I--E--L--L--E--L--V--G--I--I--Y--N--K--T--K--F--C--H--E--*--R--I--D--I--Y--D--I--T--V--*--*--L--L--N--L--*--L--I--T--I--E--V--*--E--Q--V--M--P--F--Y--*--I--K--F--I--*--N--N--R--E--G--P--K--*--*--G--F--C--K--V--T--T--P--L--L--H--I--T--F--*--*--K--R--T--T--R--R--T--G--F--A--*--S--R--I--R--S--F--V--S--N--D--C--R--S--R--E--R--A--Y--F--K--F--K--E--G--N--R--Q--R--S--G--T--L--S--*--R--I--R--F--F--I--K--R--V--I--N--Y--L--*--Y--L--I--I--S--N--*--L--C--I--V--N--K--L--I--S--N--L--L--V--C--I--D--S--D--*--S--F--*--I--N--G--*--T--I--F--T--S--T--R--Y--S--I--T--T--C--D--D--S--N--H--Y--Q--M--I--Y--A--F--*--D--S--N--G--G--K--R--S--K--L--Q--D--S--T--T--E--V--K--D--K--D--G--T--H--I--Q--Q--V--K--D--K--I--*--V--L--C--T--Q--S--Q--P--S--E--A--L--I--Y--*--V--*--M--N--Y--L--G--*--L--E--K--*--T--M--R--T--W--M--C--M--K--N--C--I--E--N--V--H--V--V--G--C--R--A--R--L--T--L--E--I--F--C--I--T--V--L--G--G--K--G--A--G--T--V--T--K--A--I--N--K--C--L--K--E--S--G--I--S--E--V--Y--H--G--L--H--S--F--A--F--W--M--E--G--L--T--I--Y--L--S--N--C--W--N--D--N--Y--I--F--S--*--K--I--E--C--N--N--N--S--V--Y--*--*--P--F--G--D--S--*--P--I--R--R--H--Y--*--L--G--S--K--R--T--L--F--L--G--D--I--C--S--T--S--N--F--N--P--F--R--L--H--S--I--T--*--K--I--K--Y--W--R--F--L--D--S--W--K--G--Y--A--K--W--R--S--E--S--H--K--Q--E--N--T--S--I--S--T--D--A--S--G--Y--K--Y--G--A--V--V--C--F--Q--G--E--K--V--E--L--G--D--F--L--E--T--N--D--S--R--P--I--H--L--K--E--A--E--A--V--L--Q--V--L--K--S--I--E--E--V--I--R--D--S--R--V--D--L--L--V--D--N--V--A--V--L--S--V--W--N--T--Q--G--G--R--D--R--S--L--N--N--I--T--K--Q--I--F--Q--L--V--T--S--C--N--C--D--L--H--M--Q--Y--V--P--S--E--*--M--G--Q--I--Y--L--F--E--C--M--F--E--Y--W--F--M--A--A--C--*--K--A--I--W--T--S--F--N--G--P--H--G--Y--*--F--*--C--D--E--I--F--R--R--Y--S--T--T--K--F--N--P--Y--P--T--P--Y--S--S--C--V--N--L--F--A--Q--D--L--P--V--V--V--N--A--Y--V--Y--P--P--F--A--L--I--H--P--V--L--L--F--L--R--E--Q--K--V--G--M--C--T--F--V--L--P--L--I--K--P--V--P--V--W--W--P--L--V--Q--K--H--V--I--Q--S--L--E--L--G--T--R--G--D--K--A--V--I--R--V--P--*--K--R--F--H--H--*--*--*--G--S--E--M--A--T--S--C--I--Q--A--I--V--Y--L--S--*--L--F--F--I--L--*--G--E--S--L--D--L--V--K--I--D--K--R--I--K--E--L--N--D--K--S--S--S--S--S--L--S--K--R--R--D--T--L--K--N--S--L--N--L--F--L--P--L--L--R--K--N--C--T--I--S--N--C--T--P--D--D--L--K--H--F--W--Y--G--R--M--I--L--V--K--H--Q--F--I--K--*--I--V--H--F--W--V--L--K--L--*--M--P--K--R--I--S--S--W--D--S--I--G--H--G--I--K--F--V--G--D--L--L--R--K--W--S--W--*--I--L--G--*--E--K--*--Y--R--*--P--C--S--G--T--L--Y--Q--T--V--C--E--T--Y--S--R--G--T--S--G--S--T--C--S--F--*--A--G--K--S--N--F--S--G--*--N--K--Q--N--C--L--V--Y--R--*--*--I--G--*--G--*--Y--F--I--E--G--K--I--C--A--P--*--R--S--S--L--E--E--I--T--V--L--W--G--K--S--G--K--*--L--G--N--A--C--R--T--G--G--K--E--I--R--*--I--L--V--*--L--K--N--K--H--L--G--K--L--*--E--E--I--N--I--N--L--M--F--L--L--L--R--N--A--E--T--R--L--F--V--Q--*--L--V--C--T--H--M--F--K--I--V--K--E--W--A--C--I--L--Q--M--V--F--F--F--E--L--F--*--R--M--V--E--S--*--V--I--E--*--H--I--Q--*--C--M--K--D--*--F--G--I--L--L--C--*--E--Y--M--R--V--R--L--L--I--V--L--E--S--G--V--Q--*--L--W--L--Y--L--V--R--L--P--M--*--D--K--L--*--T--M--W--D--G--R--E--R--E--Q--Q--N--I--I--V--D--C--Q--L--*--W--N--L--I--I--L--Q--V--D--*--Q--T--V--Q--V--N--V--I--L--W--K--I--S--S--N--S--M--G--I--L--I--F--Y--K--K--L--F--I--N--D--K--A--F--K--M--S--I--Q--L--R--E--*--D--R--S--F--V--F--E--F--A--V--D--*--L--S--Q--V--*--Y--V--T--A--K--V--M--T--S--S--F--S--K--M--Y--Q--*--Y--*--I--L--R--M--S--T--V--D--G--I--D--G--S--E--Y--P--*--V--Y--P--I--H--P--T--L--V--Y--V--L--V--L--G--S--*--*--*--L--H--F--P--I--H--E--K--N--*--S--T--N--V--Y--T--W--D--R--Y--R--C--N--V--H--L--Y--F--I--N--L--N--F--A--I--S--Y--S--L--Q--T--Y--M--L--Y--I--*--C--S--N--A--T--F--Y--M--*--*--*--N--I--P--M--S--G--I--R--R--T--S--R--K--R--I--A--K--I--S--M--K--H--V--K--S--P--P--T--T--A--S--D--T--G--N--A--N--S--D--P--I--S--*--T--N--G--K--E--P--L--I--N--I--P--P--M--I--A--D--M--*--I--S--T--A--R--N--H--I--L--L--N--F--Y--H--L--L--H--Q--M--*--Q--S--F--Y--I--F--I--K--Y--S--T--K--I--R--*--G--K--F--S--T--T--A--S--V--H--*--F--*--I--M--V--S--S--K--*--Q--V--V--I--I--S--L--G--N--G--N--S--I--F--P--K--T--Y--*--L--I--K--R--M--I--T--L--T--L--K--F--I--N--L--K--Q--*--Y--L--*--R--*--V--*--F--Q--S--S--*--E--K--T--T--H--S--L--G--L--L--R--H--S--S--R--L--K--R--I--*--C--V--S--K--I--Y--L--K--I--C--N--K--I--*--Y--R--*--L--S--Q--L--F--M--N--C--H--S--T--R--*--T--*--L--Q--K--M--F--V--S--K--I--P--P--N--K--K--S--R--H--A--S--S--C--C--H--V--E--S--*--P--S--D--F--T--L--K--I--L--W--R--K--H--S--I--S--H--I--L--V--*--V--D--R--L--I--F--F--A--*--S--S--R--S--I--N--C--L--*--T--V--F--M--L--*--D--F--S--M--H--V--I--C--*--T--L--T--T--I--V--I--L--L--Y--Y--R--E--N--E--S--L--*--T--F--E--I--N--L--K--N--K--T--Q--K--N--N--P--L--T--F--I--I--L--V--F--A--S--*--K--A--I--W--R--H--L--Y--L--L--D--S--Y--R--L--G--L--I--T--H--L--I--Q--P--A--S--F--Q--I--L--H--*--V--L--H--W--P--N--P--T--I--*--E--I--C--S--F--K--Y--F--E--N--I--L--N--Y--*--I--T--R--T--K--M--C--F--R--E--C--C--I--L--N--C--I--S--S--I--V--A--V--S--K--*--I--F--S--T--K--P--W--E--Y--R--E--I--H--I--Q--F--K--C--L--V--*--*--Q--*--Y--S--Y--L--A--T--L--P--R--D--S--L--*--L--V--L--C--T--E--A--D--V--L--G--G--V--G--G--L--A--G--R   -2               gi|270063462|gb|GU207455.1|     -3      --K--Q--Q--I--*--I--A--F--L--I--L--S--I--I--K--Q--Y--T--S--I--D--L--V--M--I--N--C--R--I--V--L--I--N--R--F--N--S--*--L--A--*--N--E--S--L--F--C--R--N--Y--Y--V--W--*--S--H--H--K--V--F--A--L--*--H--I--E--S--I--L--Y--T--N--N--I--S--E--G--F--S--M--Y--P--K--C--N--T--S--L--R--K--*--*--R--H--L--S--Q--S--Y--T--K--E--E--D--L--L--R--S--G--N--V--I--Y--F--L--I--L--D--N--R--A--V--C--C--R--*--G--H--C--R--*--V--F--L--Y--G--Q--W--I--Y--S--D--I--S--I--A--E--D--*--F--W--S--E--I--L--K--M--M--R--R--R--*--G--R--P--H--G--M--A--G--T--G--C--T--M--R--A--R--Y--T--*--S--A--R--W--*--*--L--V--Y--R--*--C--*--I--N--I--G--D--V--I--K--W--Y--D--F--T--E--T--D--D--G--*--L--H--*--F--N--M--D--F--M--*--V--E--N--R--R--*--N--V--C--N--V--L--K--K--N--V--P--S--R--V--N--N--N--K--H--T--C--D--V--N--Q--L--N--L--Y--D--F--S--I--L--W--P--E--L--V--D--M--Y--G--S--I--C--I--L--A--F--F--T--F--G--L--M--L--*--M--V--L--P--T--V--L--K--K--N--I--P--*--K--E--N--N--Y--Y--Q--L--S--E--S--*--G--I--F--K--I--R--M--A--L--N--S--*--L--G--L--L--L--L--G--V--P--K--I--C--L--M--R--M--Y--*--R--L--L--R--I--*--L--E--K--T--G--L--S--Y--W--N--W--*--V--L--F--T--I--K--Q--S--S--V--M--N--E--E--L--I--F--M--I--L--Q--C--N--S--S--*--I--S--N--*--*--L--*--K--F--K--N--K--*--C--H--F--I--E--L--N--S--F--K--I--I--G--K--D--P--N--N--E--D--F--A--K--L--L--L--H--F--C--I--L--H--S--N--K--K--G--Q--P--E--G--Q--D--L--H--D--P--E--L--G--H--S--S--A--T--T--A--D--Q--E--K--G--P--T--S--N--S--R--K--A--T--D--K--D--Q--V--L--L--V--N--G--L--G--F--S--S--K--E--*--L--I--I--C--D--I--*--L--L--V--I--N--Y--V--L--L--I--N--*--F--L--T--Y--*--F--A--L--I--V--I--N--H--F--E--*--M--V--K--Q--Y--S--L--Q--P--G--T--V--L--L--L--V--M--I--V--I--I--I--K--*--F--M--H--F--R--I--Q--M--V--E--N--V--V--S--Y--K--I--Q--Q--L--K--*--K--T--R--M--V--H--I--F--S--R--*--R--T--R--F--K--F--S--V--P--S--P--S--P--V--K--H--*--F--I--K--F--R--*--I--I--*--V--S--*--K--S--E--P--*--G--H--G--C--V--*--R--T--V--*--K--M--Y--M--*--*--D--A--E--Q--D--*--H--W--K--F--F--V--*--R--C--L--G--G--K--V--Q--V--Q--*--L--R--P--*--I--N--V--*--R--R--V--G--F--L--K--F--I--M--V--Y--T--V--L--P--F--G--W--K--A--S--P--Y--I--Y--Q--T--V--G--M--T--I--T--Y--F--L--R--K--L--N--V--I--T--T--Q--Y--I--D--D--R--L--V--I--A--N--P--S--E--G--T--T--D--L--E--A--K--E--H--C--F--W--V--I--Y--A--L--L--Q--I--L--I--R--L--G--Y--T--V--S--L--E--K--S--N--T--G--V--S--*--T--R--G--R--V--T--Q--S--G--G--Q--R--V--I--S--K--K--I--P--V--F--Q--Q--M--L--L--A--T--S--M--G--Q--*--F--V--F--R--G--K--R--L--N--L--E--I--F--W--R--Q--T--T--V--D--L--F--I--*--R--K--L--R--Q--S--F--R--S--*--S--Q--*--R--K--*--L--E--I--Q--G--L--T--Y--W--*--I--M--L--L--F--L--V--F--G--T--L--K--E--D--G--I--D--L--*--T--T--*--Q--N--R--Y--F--S--W--*--L--L--V--I--V--I--C--T--C--N--M--Y--P--Q--S--E--W--G--R--F--T--C--L--N--A--C--L--N--I--G--S--W--Q--R--V--E--R--Q--F--G--P--H--S--M--D--L--M--A--I--D--S--N--V--M--K--Y--S--G--G--T--A--L--P--N--L--T--H--I--L--P--H--I--H--L--V--*--I--C--L--L--R--I--C--Q--W--W--L--M--H--M--F--I--L--L--L--P--L--Y--I--L--Y--S--Y--F--*--G--N--R--K--L--V--C--A--P--L--S--Y--H--*--S--S--L--Y--L--F--G--G--L--L--F--K--S--M--*--S--R--V--*--S--*--G--P--G--E--I--R--Q--*--*--G--F--L--R--K--G--F--I--T--D--N--K--G--L--R--W--P--L--V--A--F--R--L--L--F--I--*--A--N--C--F--S--F--C--R--E--K--A--W--I--W--L--R--L--T--R--G--L--R--N--*--M--T--N--L--H--H--H--L--Y--L--R--E--E--I--L--*--K--T--L--*--I--Y--F--F--H--Y--*--G--K--I--A--Q--Y--L--I--V--P--L--M--I--*--S--I--S--G--M--E--G--*--F--W--*--N--T--S--S--*--N--K--L--Y--I--F--G--Y--*--S--C--E--C--P--R--G--L--A--A--G--T--V--S--V--M--V--*--N--L--S--E--I--F--Y--E--S--G--R--G--K--F--W--D--E--R--S--N--T--G--N--P--A--A--A--H--C--I--K--Q--Y--V--K--L--I--Q--E--E--Q--A--A--A--H--V--V--S--K--Q--A--N--P--T--F--L--G--K--I--N--R--I--V--S--Y--I--D--N--E--L--G--R--V--D--I--S--S--K--E--R--Y--V--H--L--K--D--Q--A--W--R--K--L--Q--F--F--G--G--N--R--A--N--D--*--G--M--L--V--G--Q--E--V--K--R--L--D--E--F--W--F--S--*--K--T--N--I--W--E--N--F--K--R--K--*--T--*--I--S--C--F--C--Y--*--E--M--Q--R--Q--D--C--L--S--S--N--W--F--A--H--I--C--S--R--L--*--K--N--G--R--V--S--C--K--W--F--S--F--S--S--C--F--R--E--W--*--S--H--R--*--*--S--D--I--F--S--N--V--*--K--T--D--S--V--S--Y--F--V--R--N--I--*--G--*--D--S--S--*--F--*--S--R--V--C--S--D--S--G--F--I--W--F--G--C--Q--C--R--T--N--Y--E--P--C--G--M--V--G--R--G--N--S--R--I--L--*--*--I--A--S--F--S--G--I--*--L--Y--C--R--*--I--S--R--Q--C--R--S--M--*--F--C--G--R--S--V--P--T--V--W--G--F--*--Y--F--T--K--S--F--S--*--T--I--R--P--S--K--C--Q--Y--N--*--G--N--K--T--D--P--L--F--S--N--L--Q--*--I--N--Y--P--K--S--N--T--S--P--R--K--*--*--R--H--L--S--Q--R--C--T--N--D--T--E--Y--*--G--*--A--R--*--M--A--L--M--V--Q--S--I--H--E--F--T--P--S--T--P--P--L--Y--M--F--L--F--S--V--H--D--N--D--Y--I--S--Q--Y--M--R--R--T--R--A--R--T--Y--T--P--G--I--G--I--G--A--M--S--I--S--I--S--S--I--*--I--L--Q--F--L--T--L--C--R--R--I--C--C--T--F--D--V--Q--M--Q--H--F--T--C--N--D--K--T--Y--L--C--L--G--S--E--E--P--V--G--K--E--L--R--K--S--P--*--N--M--S--R--V--P--Q--L--L--P--P--T--R--G--M--P--T--Q--I--P--S--R--K--P--T--E--K--S--R--*--L--T--F--L--R--*--*--L--I--C--R--F--Q--L--Q--E--I--I--F--C--*--I--F--T--I--F--Y--I--K--C--D--K--V--S--I--F--L--*--N--T--L--L--K--Y--D--K--V--N--F--Q--Q--P--R--L--Y--T--N--S--K--S--W--Y--H--L--N--N--K--W--*--S--Y--H--W--E--T--E--I--Q--S--F--Q--K--R--I--D--*--*--R--E--*--*--H--L--L--L--N--S--S--I--*--N--N--D--T--F--R--D--R--Y--D--S--R--A--H--E--R--K--P--H--I--A--*--D--C--S--A--I--H--Q--D--*--K--E--F--S--V--S--Q--K--Y--I--*--K--S--A--T--K--F--N--T--D--N--Y--H--S--Y--L--*--I--V--I--L--P--D--K--H--D--Y--K--R--C--S--C--P--K--Y--L--P--I--R--S--P--D--T--P--P--A--A--V--M--*--N--P--N--H--P--T--S--L--L--R--Y--Y--G--E--N--T--A--F--R--I--F--*--S--K--W--T--D--*--F--S--L--L--E--V--Q--G--P--*--T--A--Y--K--L--S--L--C--F--K--I--S--V--C--M--*--F--A--K--P--*--L--Q--L--*--Y--Y--C--I--T--E--K--M--N--P--Y--R--H--S--R--S--I--*--K--T--K--P--K--K--I--I--L--*--P--L--L--Y--W--F--L--R--A--R--R--Q--S--G--D--I--C--T--Y--L--I--H--T--D--W--V--*--*--H--I--*--Y--S--L--P--A--S--K--Y--S--I--R--Y--C--I--G--Q--T--Q--Q--Y--E--R--F--V--P--L--N--I--L--R--I--Y--S--I--I--E--*--H--A--Q--R--C--V--S--A--N--V--A--F--*--T--A--L--A--Q--S--L--L--F--L--N--E--S--F--Q--R--N--P--G--N--I--E--K--S--T--Y--S--S--S--A--W--F--D--N--N--D--T--H--T--*--P--H--F--Q--E--I--P--C--S--W--F--S--A--Q--K--Q--T--Y--L--V--V--L--A--V--W--R--E--D  -3                                                                                                                                                                                                                                                                                                                                                                                                                                                                                                                                                                                                                                                                                                                                                                                                                                                                                                                                                                                                                                                                                                                                                                                                                                                                                                                                                                                                                                                                                                                                                                                                                                                                                                                                                                                                                                                                                                                                                                                                                                                                                                                                                                                                                                                                                                                                                                                                                                                                                                                                                                                                                                                                                                                                                                                                                                  <--frame 3 fragmented RT-----------------------------------------------------------------------------------------------------------------------------------------------------------------------------------------------------------------------------------------------------------<--frame 2 RT------->------------------------------------RT ends---->< RNaseH continues frame 2-----------------------------------------------------------------------------------------------------------------------------------------------------------------------------------------------------------------------------------------------------------------------------------------------------------------------------------------------------------------------<--DNA methylase continues in same frame-2with stop codons------------------------------------------------------------------------------------------------------------------------------------------------------------------------------------------------------------------------------------------------------------------------------------------>                                                                                                                                                                                                                                                              <--ALOG in frame 1---------------------------------------------------------------<-continues in frame 3--------------------------------------------------------------------------------------------------------------->               gi|270063413|gb|GU207406.1|     .       1        :         :         :         :         50        :         :         :         :         100       :         :         :         :         150       :         :         :         :         200       :         :         :         :         250       :         :         :         :         300       :         :         :         :         350       :         :         :         :         400       :         :         :         :         450       :         :         :         :         500       :         :         :         :         550       :         :         :         :         600       :         :         :         :         650       :         :         :         :         700       :         :         :         :         750       :         :         :         :         800       :         :         :         :         850       :         :         :         :         900       :         :         :         :         950       :         :         :         :         1000      :         :         :         :         1050      :         :         :         :         1100      :         :         :         :         1150      :         :         :         :         1200      :         :         :         :         1250      :         :         :         :         1300      :         :         :         :         1350      :         :         :         :         1400      :         :         :         :         1450      :         :         :         :         1500      :         :         :         :         1550      :         :         :         :         1600      :         :         :         :         1650      :         :         :         :         1700      :         :         :         :         1750      :         :         :         :         1800      :         :         :         :         1850      :         :         :         :         1900      :         :         :         :         1950      :         :         :         :         2000      :         :         :         :         2050      :         :         :         :         2100      :         :         :         :         2150      :         :         :         :         2200      :         :         :         :         2250      :         :         :         :         2300      :         :         :         :         2350      :         :         :         :         2400      :         :         :         :         2450      :         :         :         :         2500      :         :         :         :         2550      :         :         :         :         2600      :         :         :         :         2650      :         :         :         :         2700      :         :         :         :         2750      :         :         :         :         2800      :         :         :         :         2850      :         :         :         :         2900      :         :         :         :         2950      :         :         :         :         3000      :         :         :         :         3050      :         :         :         :         3100      :         :         :         :         3150      :         :         :         :         3200      :         :         :         :         3250      :         :         :         :         3300      :         :         :         :         3350      :         :         :         :         3400      :         :         :         :         3450      :         :         :         :         3500      :         :         :         :         3550      :         :         :         :         3600      :         :         :         :         3650      :         :         :         :         3700      :         :         :         :         3750      :         :         :         :         3800      :         :         :         :         3850      :         :         :         :         3900      :         :         :         :         3950      :         :         :         :         4000      :         :         :         :         4050      :         :         :         :         4100      :         :         :         :         4150      :         :         :         :         4200      :         :         :         :         4250      :         :         :         :         4300      :         :         :         :         4350      :         :         :         :         4400      :         :         :         :         4450      :         :         :         :         4500      :         :         :         :         4550      :         :         :         :         4600      :         :         :         :         4650      :         :         :         :         4700      :         :         :         :         4750      :         :         :         :         4800      :         :         :         :         4850      :         :         :         :         4900      :         :         :         :         4950      :         :         :         :         5000      :         :         :         :         5050      :         :         :         :         5100      :         :         :         :         5150      :         :         :         :         5200      :         :         :         :         5250      :         :         :         :         5300      :         :         :         :         5350      :         :         :         :         5400      :         :         :         :         5450      :         :         :         :         5500      :         :         :         :         5550      :         :         :         :         5600      :         :         :         :         5650      :         :         :         :         5700      :         :         :         :         5750      :         :         :         :         5800      :         :         :         :         5850      :         :         :         :         5900      :         :         :         :         5950      :         :         :         :         6000      :         :         :         :         6050      :         :         :         :         6100      :         :         :         :         6150      :         :         :         :         6200      :         :         :         :         6250      :         :         :         :         6300      :         :         :         :         6350      :         :         :         :         6400      :         :         :         :         6450      :         :         :         :         6500      :         :         :         :         6550      :         :         :         :         6600      :         :         :         :         6650      :         :         :         :         6700      :         :         :         :         6750      :         :         :         :         6800      :         :         :         :         6850      :         :         :         :         6900      :         :        .               gi|270063413|gb|GU207406.1|     1       AGAGGGATATTGGAATATCCTTATACCTAAATCAAACAAAAGGTCGTTCCGAGAACAAAGTTGAATTTTTATCAACTACAAAATGAGACTTACCTGCAATATATTTTCTTTACTACATATCAATGTTATAATAAAAAAATTAAAATGCAATCAAAAGACTGAAATAAAACGTATGGAAATGGCTTGAATTGAAATCTTTCTGTATTTTATTGGGGCTCAATTGTTTCAAACTAAAAACTGATTAATGTCCATAATCGTTATGAACTAGAATACTACATGTACATAATGTATCGATGTTTGTAAAACACGCACTCTTTTCAAATTACGACCACGTGAAATATTCAACTACAGCATTCTTTATTATTTTTCAACTTAGGTTGAAGGTTTATAACCAATAAATTTCTAAATACATACAAATTTGCAGATTAAATCTATTTATATCAACCGATCGACCTTATTAAGACACATTTTAGACAATATTCATTCATCATAGCTTGCACGGTGTCCCGGCCGTTATTGTGTTTCTGTTAACAGAGTCGTCATATTTGTCTTCGGCATTAACATGGACGACGACATTATTTATGTCAGTTCGTTTATAGCGTGCTGGAAACAAGAAGAGTAGAACAAATCCTAGAAGTAGACTTCCTGATAAGGACCAGTTCATCCAGCTAGTTCCTATAATTAAACAACAAATATGAATTGCATTTTTGATATTATCTATCATAAAACAATATACTTCTATAGATTTGGTCATGATAAATTGTAGAATTGTACTTATTAACAGATTTAATTCTTAATTAGCTTAGAATGAGTCTTTATTTTGTCGCAACTACTATGTTTGGTAAAGTCATCATAAAGTATTTGCACTTTAGCACATAGAAAGCATATTATATACAAATAATATTTCTGAAGGATTTAGCATGTATCCCAAGTGTAATACGTCACTGCGAAAGTGATGACGTCATCTTTCTCAGAGCTACACCAAGGAGGAAGACCTATTGAGAAGCGGCAATGTAATATATTTTCTTATATTAGACAATAGAGCGGTCTGTTGCCGATGAGGGCATTGCCGATGAGTGTTTTTATATGGCCAGTGGATCTACAGTGATATATCAATCGCTGAGGATTGATTTTGGAGTGAAATTTTAAAAATGATGCGGCGTCGATGAGGACGACCACATGGTATGGCTGGTACTGGATGTACCATGAGGGCCAGATATACATAATCAGCGAGGTGGTAATAGCTTGTTTACCGCTGATGTTAGATTAATATTGGTGATGTGATTAAATGGTATGATTTTACTGAGACTGACGATGGATGATTGCACTGATTTAACATGGATTTTATGTAGGTCGAAAATCGCAGATAAAACGTATGCAACGTTTTAAAAAAAAATGTACCGTCAAGAGTAAATAATAACAAGCATACGTGTGATGTGAATCAATTAAACCTTTATGATTTTTCAATTTTGTGGCCGGAATTAGTAGATATGTACGGATCTATTTGTATATTAGCTTTTTTTACTTTCGGCTTGATGTTGTAGATGGTGTTACCTACTGTTTTAAAAAAAAACATTCCCTGAAAGGAAAACAATTATTACCAGTTATCAGAAAGTTGAGGGATATTCAAGATCCGCATGGCATTAAACAGTTAGCTAGGACTTCTTCTGCTAGGCGTACCGAAAATATGTCTGATGAGGATGTATTGAAGGCTGTTGAGGATTTAACTGGAGAAAACGGGATTGAGTTACTGGAACTGGTAGGTATTATTTACAATAAAACAAAGTTCTGTCATGAATGAAGAATTGATATTTATGATATTACAGTGTAATAGCTCTTAAATCTCTAATTAATAACTATAGAAGTTTAAGAACAAGTGATGCCATTTTATTGAATTAAATTCATTTAAAATAATAGGGAAGGACCCAAATAATGAGGATTTTGCAAAGTTACTACTCCACTTCTGCATATTACATTCTAATAAAAAAGGACAACCAGAAGGACAGGATTTGCATGATCCAGAATTAGGTCATTCGTCAGCAACGACTGCAGATCAAGAGAAAGGGCCTACTTCAAATTCAAGGAAGGCAACAGACAAAGATCAGGTACTCTTAGTTAACGGATTAGGTTTTTCATCAAAAGAGTGATTAATTATCTGTGATATTTAATTATTAGTAATTAATTATGTATTGTTAATAAACTGATTTCTAACCTATTAGTTTGCATTGATAGTGATTAATCATTTTGAATAAATGGTTAAACAATATTCACTTCAACCAGGTACAGTATTACTACTTGTGATGATAGTAATCATTATCAAATGATTTATGCATTTTAGGATTCAAATGGTGGAAAACGTAGTAAGCTACAAGATTCAACAACTGAAGTAAAAGACAAGGATGGTACACATATTCAGCAGGTGAAGGACAAGATTTAAGTTCTCTGTACCCAGTCCCAGCCCAGTGAAGCACTGATTTATTAAGTTTAGATGAATTATCTAGGTTAGCTAGAAAAGTGAACCATGAGGACATGGATGTGTATGAAGAACTGTATAGAAAATGTACATGTAGTAGGATGCAGAGCAAGATTAACATTGGAAATTTTTTGTATAACGGTGCTTGGGGGGAAAGGTGCAGGTACAGTGACTAAGGCCATAAATAAATGTTTAAAGGAGAGTGGGATTTCTGAAGTTTATCATGGTTTACACAGTTTTGCCTTTTGGATGGAAGGCCTCACCATATATTTATCAAACTGTTGGAATGACAATTACATATTTTCTTAGAAAATTGAATGTAATAACAACTCAGTATATTGATGACCGTTTGGTGATAGCTAACCCATCAGAAGGCACTACTGACTTGGAAGCAAAAGAACATTGTTTTTGGGTGATATATGCTCTACTTCAAATTTTAATCCGTTTAGGTTACACAGTATCACTTGAAAAATCAAATACTGGCGTTTCTTAGACTCGTGGAAGGGTTACGCAAAGTGGAGGTCAGAGAGTCATAAGCAAGAAAATACCAGTATTTCAACAGATGCTTCTGGCTACAAGTATGGGGCAGTAGTTTGTTTTCAGGGGGAAAAGGTTGAACTTGGAGATTTTTTGGAGACAAACGACAGTAGACCTATTCATTTGAAGGAAGCTGAGGCAGTCCTTCAGGTCTTGAAGTCAATAGAGGAAGTAATTAGAGATTCAAGGGTTGACTTACTGGTAGATAATGTTGCTGTTCTTAGTGTTTGGAACACTCAAGGAGGACGGGATAGATCTTTGAACAACATAACAAAACAGATATTTCAGTTGGTGACTTCTTGTAATTGTGATCTGCACATGCAATATGTACCCTCAGAGTGAATGGGGCAGATTTACCTGTTTGAATGCATGTTTGAATATTGGTTCATGGCAGCGTGTTGAAAGGCAATTTGGACCTCATTCAATGGACCTCATGGCTATTGATTCTAATGTGATGAAATATTCAGGAGGTACAGCACTACCAAATTTAACCCATATCCTACCCCATATTCATCTTGTGTAAATTTGTTTGCTCAGGATTTGCCAGTGGTGGTTAATGCATATGTTTATCCTCCTTTTGCCCTTATACATCCTGTACTCTTATTTTTAAGGGAACAGAAAGTTGGTATGTGCACCTTTGTCCTACCATTGATCAAGCCTGTACCTGTTTGGTGGCCTCTTGTTCAAAAGCATGTAATCCAGAGTTTAGAGTTAGGGACCAGGGGAGATAAGGCAGTAATAAGGGTTCCTTAGAAAAGGTTTCATCACTGATAATAAGGGTCTGAGATGGCCACTAGTTGCATTCAGGCTATTGTTTATCTAAGCTAATTGTTTTTCATTTTGTAGGGAGAAAGCTTGGATCTGGTTAAGATTGACAAGAGGATTAAGGAATTGAATGACAAATCTTCATCATCATCTTTATCTAAGAGAAGAGATACTTTGAAAAACTCTCTAAATTTATTTCTTCCACTATTGAGGAAAAATTGCACAATATCTAATTGTACCCCTGATGATCTAAAGCATTTCTGGTATGGAAGGATGATTTTGGTAAAACACCAGTTCATAAAATAAATTGTACATTTTTGGGTACTAAAGTTGTGAATGCCCAAGAGGATTAGCAGCTGGGACAGTATCGGTCATGGTATAAAATTTGTCGGAGATCTTTTACGAAAGTGGTCGTGGTAAATTCTGGGATGAGAGAAGTAATACAGGTAACCCTGCAGCGGCACATTGTATCAAACAGTATGTGAAACTTATTCAAGAGGAACAAGCGGCAGCACATGTAGTTTCTAAGCAGGCAAATCCAACTTTTCTGGGTAAAATAAACAGAATTGTCTCGTATATAGATAATGAATTGGGTAGGGTTGATATTTCATCGAAGGAAAGATATGTGCACCTTAAAGATCAAGCTTGGAGGAAATTACAGTTCTTTGGGGGAAATCGGGCAAATGACTAGGGAATGCTTGTAGGACAGGAGGTAAAGAGATTAGATGAATTCTGGTTTAGTTAAAAAACAAACATTTGGGAAAACTTTAAGAGGAAATAAACATAAATCTCATGTTTTTGTTATTAAGAAATGCAGAGACAAGACTGTTTGTCCAGTAACTGGTTTGCACACATATGTTCAAGATTGTAAAAGAATGGGCGTGTATCTTGCAAATGGTTTTCTTTTTCGAGTTGTTTTAGAGAATGGTAGAGTCATAGGTGATAGAGTGACATATTCAGTAATGTATGAAAGACTGATTCGGTATCTTACTTTGTTAGGAATATATGAGGGTGAGACTCCTCATAGTTTTAGAGTCGGGTGTGCAGTGACTCTGGCTTTATCTGGTTCGGTTGCCAATGTAGGACAAATTATGAACCATGTGGGATGGTAGGGAGAGGGAACAGCAGAATATTATAGTAGATTGCCAGCTTTAGTGGAATCTGATTATATTGCAGGTAGATTAGCAGACAGTGCAGGTCAATGTGATTTTGTGGAAGATCAGTTCCAACAGTATGGGGATTTTAATATTTTACAAAAAGCTTTTCATAAACGATAAGGCCTTCAAAATGTCAATACAATTAAGGGAATAAGACAGATCCTTTGTTTTCGAATTTGCAGTAGATTAATTATCCCAAGTCTAATACGTCACCGCGAAAGTGATGACGTCATCTTTCTCAAAGATGTACCAATGATACTGAATATTGAGGATGAGCACGGTAGATGGCATTGATGGTTCAGAGTATCCATGAGTTTACCCCATCCACCCCACCCTTGTATATGTTCTTGTTCTCGGTTCATGATAATGACTACATTTCCCAATACATGAGAAGAACTAGAGCACGAACGTATACACCTGGGATAGGTATAGGTGCAATGTCCATCTCTATTTCATCAATTTAAATTTTGCAATTTCTTACTCTTTGCAGACGTATATGTTGTACATTTGATGTTCAAATGCAACATTTTACATGTAATGATAAAACATACCTATGTCTGGGATCAGAAGAACCAGTAGGAAAAGAATTGCGAAAATCTCCATGAAACATGTCAAGAGTCCCCCAACTACTGCCTCCGACACGGGGAATGCCAACTCAGATCCCATCTCGTAAACCAACGGAAAAGAGCCGTTGATTAACATTCCTCCGATGATAGCTGATATGTAGATTTCAACTGCAAGAAATCATATTTTGTTGAATTTTTACCATCTTTTACATCAAATGTGACAAAGTTTCTATATTTTTATAAAATACTCTACTAAAATACGATAAGGTAAATTTTCAACAACCGCGTCTGTACACTAATTCTAAATCATGGTATCATCTAAATAACAAGTGGTGATCATATCATTGGGAAACGGAAATTCAATCTTTCCAAAAACGTATTGATTAATAAAGAGAATGATAACACTTACTCTTAAATTCATCAATTTGAAACAATGATACCTTTAGAGATAGGTATGATTCCAGAGCTCATGAGAGAAAACCACACATAGCCTAGGACTGCTCCGCCATTCATCAAGATTAAAAAGAATTTAGTGTGTCTCAAAAATATATCTGAAAATCTGCAACAAAATTTAATACAGATAACTATCACAGCTATTTATGAATTGTCATTCTACCCGATAAACATGATTACAAAAGATGTTCGTGTCCAAAATACCTCCCAATAAGAAGTCCAGACACGCCTCCAGCTGCTGTCATGTAGAATCCTAACCATCCGACTTCACTCTTAAGATATTATGGAGAAAACACAGCATTTCGCATATTTTAGTCTAAGTGGACAGATTAATTTTCTTTGCTTGAAGTTCAAGGTCCATAAACTGCTTATAAACTGTCTTTATGCTTTAAGATTTCAGTATGCATGTAATTTGCTAAACCCTGACTACAATTGTAATATTATTGTATTACAGAGAAAATGAATCCTTATAGACATTCGAGATCAATTTGAAAAACAAAACCCAAAAAAATAATCCTTTAACCTTTATTATATTGGTTTTTGCGAGCTAGAAGGCAATCTGGAGACATTTGTACTTACTTGATTCATACCGACTGGGTCTAATAACACATCTAATACAGCCTGCCAGCTTCCAAATACTCCATTAGGTATTGCATTGGCCAAACCCAACAATATGAGAGATTTGTTCCTTTAAATATTTTGAGAATATACTCAATTATTGAATAACACGCACAAAGATGTGTTTCCGCGAATGTTGCATTTTAAACTGCATTAGCTCAATCGTTGCTGTTTCTAAATGAATCTTTTCAACGAAACCCTGGGAATATCGAGAAATCCACATACAGTTCAAGTGCTTGGTTTGATAACAATGATACTCATACCTAGCCACACTTCCAAGAGATTCCTTGTAGTTGGTTCTCTGCACAGAAGCAGACGTACTTGGTGGTGTTGGCGGTTTGGCGGGAAGAT        6920               gi|270063413|gb|GU207406.1|     1       R--G--I--L--E--Y--P--Y--T--*--I--K--Q--K--V--V--P--R--T--K--L--N--F--Y--Q--L--Q--N--E--T--Y--L--Q--Y--I--F--F--T--T--Y--Q--C--Y--N--K--K--I--K--M--Q--S--K--D--*--N--K--T--Y--G--N--G--L--N--*--N--L--S--V--F--Y--W--G--S--I--V--S--N--*--K--L--I--N--V--H--N--R--Y--E--L--E--Y--Y--M--Y--I--M--Y--R--C--L--*--N--T--H--S--F--Q--I--T--T--T--*--N--I--Q--L--Q--H--S--L--L--F--F--N--L--G--*--R--F--I--T--N--K--F--L--N--T--Y--K--F--A--D--*--I--Y--L--Y--Q--P--I--D--L--I--K--T--H--F--R--Q--Y--S--F--I--I--A--C--T--V--S--R--P--L--L--C--F--C--*--Q--S--R--H--I--C--L--R--H--*--H--G--R--R--H--Y--L--C--Q--F--V--Y--S--V--L--E--T--R--R--V--E--Q--I--L--E--V--D--F--L--I--R--T--S--S--S--S--*--F--L--*--L--N--N--K--Y--E--L--H--F--*--Y--Y--L--S--*--N--N--I--L--L--*--I--W--S--*--*--I--V--E--L--Y--L--L--T--D--L--I--L--N--*--L--R--M--S--L--Y--F--V--A--T--T--M--F--G--K--V--I--I--K--Y--L--H--F--S--T--*--K--A--Y--Y--I--Q--I--I--F--L--K--D--L--A--C--I--P--S--V--I--R--H--C--E--S--D--D--V--I--F--L--R--A--T--P--R--R--K--T--Y--*--E--A--A--M--*--Y--I--F--L--Y--*--T--I--E--R--S--V--A--D--E--G--I--A--D--E--C--F--Y--M--A--S--G--S--T--V--I--Y--Q--S--L--R--I--D--F--G--V--K--F--*--K--*--C--G--V--D--E--D--D--H--M--V--W--L--V--L--D--V--P--*--G--P--D--I--H--N--Q--R--G--G--N--S--L--F--T--A--D--V--R--L--I--L--V--M--*--L--N--G--M--I--L--L--R--L--T--M--D--D--C--T--D--L--T--W--I--L--C--R--S--K--I--A--D--K--T--Y--A--T--F--*--K--K--M--Y--R--Q--E--*--I--I--T--S--I--R--V--M--*--I--N--*--T--F--M--I--F--Q--F--C--G--R--N--*--*--I--C--T--D--L--F--V--Y--*--L--F--L--L--S--A--*--C--C--R--W--C--Y--L--L--F--*--K--K--T--F--P--E--R--K--T--I--I--T--S--Y--Q--K--V--E--G--Y--S--R--S--A--W--H--*--T--V--S--*--D--F--F--C--*--A--Y--R--K--Y--V--*--*--G--C--I--E--G--C--*--G--F--N--W--R--K--R--D--*--V--T--G--T--G--R--Y--Y--L--Q--*--N--K--V--L--S--*--M--K--N--*--Y--L--*--Y--Y--S--V--I--A--L--K--S--L--I--N--N--Y--R--S--L--R--T--S--D--A--I--L--L--N--*--I--H--L--K--*--*--G--R--T--Q--I--M--R--I--L--Q--S--Y--Y--S--T--S--A--Y--Y--I--L--I--K--K--D--N--Q--K--D--R--I--C--M--I--Q--N--*--V--I--R--Q--Q--R--L--Q--I--K--R--K--G--L--L--Q--I--Q--G--R--Q--Q--T--K--I--R--Y--S--*--L--T--D--*--V--F--H--Q--K--S--D--*--L--S--V--I--F--N--Y--*--*--L--I--M--Y--C--*--*--T--D--F--*--P--I--S--L--H--*--*--*--L--I--I--L--N--K--W--L--N--N--I--H--F--N--Q--V--Q--Y--Y--Y--L--*--*--*--*--S--L--S--N--D--L--C--I--L--G--F--K--W--W--K--T--*--*--A--T--R--F--N--N--*--S--K--R--Q--G--W--Y--T--Y--S--A--G--E--G--Q--D--L--S--S--L--Y--P--V--P--A--Q--*--S--T--D--L--L--S--L--D--E--L--S--R--L--A--R--K--V--N--H--E--D--M--D--V--Y--E--E--L--Y--R--K--C--T--C--S--R--M--Q--S--K--I--N--I--G--N--F--L--Y--N--G--A--W--G--E--R--C--R--Y--S--D--*--G--H--K--*--M--F--K--G--E--W--D--F--*--S--L--S--W--F--T--Q--F--C--L--L--D--G--R--P--H--H--I--F--I--K--L--L--E--*--Q--L--H--I--F--L--E--N--*--M--*--*--Q--L--S--I--L--M--T--V--W--*--*--L--T--H--Q--K--A--L--L--T--W--K--Q--K--N--I--V--F--G--*--Y--M--L--Y--F--K--F--*--S--V--*--V--T--Q--Y--H--L--K--N--Q--I--L--A--F--L--R--L--V--E--G--L--R--K--V--E--V--R--E--S--*--A--R--K--Y--Q--Y--F--N--R--C--F--W--L--Q--V--W--G--S--S--L--F--S--G--G--K--G--*--T--W--R--F--F--G--D--K--R--Q--*--T--Y--S--F--E--G--S--*--G--S--P--S--G--L--E--V--N--R--G--S--N--*--R--F--K--G--*--L--T--G--R--*--C--C--C--S--*--C--L--E--H--S--R--R--T--G--*--I--F--E--Q--H--N--K--T--D--I--S--V--G--D--F--L--*--L--*--S--A--H--A--I--C--T--L--R--V--N--G--A--D--L--P--V--*--M--H--V--*--I--L--V--H--G--S--V--L--K--G--N--L--D--L--I--Q--W--T--S--W--L--L--I--L--M--*--*--N--I--Q--E--V--Q--H--Y--Q--I--*--P--I--S--Y--P--I--F--I--L--C--K--F--V--C--S--G--F--A--S--G--G--*--C--I--C--L--S--S--F--C--P--Y--T--S--C--T--L--I--F--K--G--T--E--S--W--Y--V--H--L--C--P--T--I--D--Q--A--C--T--C--L--V--A--S--C--S--K--A--C--N--P--E--F--R--V--R--D--Q--G--R--*--G--S--N--K--G--S--L--E--K--V--S--S--L--I--I--R--V--*--D--G--H--*--L--H--S--G--Y--C--L--S--K--L--I--V--F--H--F--V--G--R--K--L--G--S--G--*--D--*--Q--E--D--*--G--I--E--*--Q--I--F--I--I--I--F--I--*--E--K--R--Y--F--E--K--L--S--K--F--I--S--S--T--I--E--E--K--L--H--N--I--*--L--Y--P--*--*--S--K--A--F--L--V--W--K--D--D--F--G--K--T--P--V--H--K--I--N--C--T--F--L--G--T--K--V--V--N--A--Q--E--D--*--Q--L--G--Q--Y--R--S--W--Y--K--I--C--R--R--S--F--T--K--V--V--V--V--N--S--G--M--R--E--V--I--Q--V--T--L--Q--R--H--I--V--S--N--S--M--*--N--L--F--K--R--N--K--R--Q--H--M--*--F--L--S--R--Q--I--Q--L--F--W--V--K--*--T--E--L--S--R--I--*--I--M--N--W--V--G--L--I--F--H--R--R--K--D--M--C--T--L--K--I--K--L--G--G--N--Y--S--S--L--G--E--I--G--Q--M--T--R--E--C--L--*--D--R--R--*--R--D--*--M--N--S--G--L--V--K--K--Q--T--F--G--K--T--L--R--G--N--K--H--K--S--H--V--F--V--I--K--K--C--R--D--K--T--V--C--P--V--T--G--L--H--T--Y--V--Q--D--C--K--R--M--G--V--Y--L--A--N--G--F--L--F--R--V--V--L--E--N--G--R--V--I--G--D--R--V--T--Y--S--V--M--Y--E--R--L--I--R--Y--L--T--L--L--G--I--Y--E--G--E--T--P--H--S--F--R--V--G--C--A--V--T--L--A--L--S--G--S--V--A--N--V--G--Q--I--M--N--H--V--G--W--*--G--E--G--T--A--E--Y--Y--S--R--L--P--A--L--V--E--S--D--Y--I--A--G--R--L--A--D--S--A--G--Q--C--D--F--V--E--D--Q--F--Q--Q--Y--G--D--F--N--I--L--Q--K--A--F--H--K--R--*--G--L--Q--N--V--N--T--I--K--G--I--R--Q--I--L--C--F--R--I--C--S--R--L--I--I--P--S--L--I--R--H--R--E--S--D--D--V--I--F--L--K--D--V--P--M--I--L--N--I--E--D--E--H--G--R--W--H--*--W--F--R--V--S--M--S--L--P--H--P--P--H--P--C--I--C--S--C--S--R--F--M--I--M--T--T--F--P--N--T--*--E--E--L--E--H--E--R--I--H--L--G--*--V--*--V--Q--C--P--S--L--F--H--Q--F--K--F--C--N--F--L--L--F--A--D--V--Y--V--V--H--L--M--F--K--C--N--I--L--H--V--M--I--K--H--T--Y--V--W--D--Q--K--N--Q--*--E--K--N--C--E--N--L--H--E--T--C--Q--E--S--P--N--Y--C--L--R--H--G--E--C--Q--L--R--S--H--L--V--N--Q--R--K--R--A--V--D--*--H--S--S--D--D--S--*--Y--V--D--F--N--C--K--K--S--Y--F--V--E--F--L--P--S--F--T--S--N--V--T--K--F--L--Y--F--Y--K--I--L--Y--*--N--T--I--R--*--I--F--N--N--R--V--C--T--L--I--L--N--H--G--I--I--*--I--T--S--G--D--H--I--I--G--K--R--K--F--N--L--S--K--N--V--L--I--N--K--E--N--D--N--T--Y--S--*--I--H--Q--F--E--T--M--I--P--L--E--I--G--M--I--P--E--L--M--R--E--N--H--T--*--P--R--T--A--P--P--F--I--K--I--K--K--N--L--V--C--L--K--N--I--S--E--N--L--Q--Q--N--L--I--Q--I--T--I--T--A--I--Y--E--L--S--F--Y--P--I--N--M--I--T--K--D--V--R--V--Q--N--T--S--Q--*--E--V--Q--T--R--L--Q--L--L--S--C--R--I--L--T--I--R--L--H--S--*--D--I--M--E--K--T--Q--H--F--A--Y--F--S--L--S--G--Q--I--N--F--L--C--L--K--F--K--V--H--K--L--L--I--N--C--L--Y--A--L--R--F--Q--Y--A--C--N--L--L--N--P--D--Y--N--C--N--I--I--V--L--Q--R--K--*--I--L--I--D--I--R--D--Q--F--E--K--Q--N--P--K--K--*--S--F--N--L--Y--Y--I--G--F--C--E--L--E--G--N--L--E--T--F--V--L--T--*--F--I--P--T--G--S--N--N--T--S--N--T--A--C--Q--L--P--N--T--P--L--G--I--A--L--A--K--P--N--N--M--R--D--L--F--L--*--I--F--*--E--Y--T--Q--L--L--N--N--T--H--K--D--V--F--P--R--M--L--H--F--K--L--H--*--L--N--R--C--C--F--*--M--N--L--F--N--E--T--L--G--I--S--R--N--P--H--T--V--Q--V--L--G--L--I--T--M--I--L--I--P--S--H--T--S--K--R--F--L--V--V--G--S--L--H--R--S--R--R--T--W--W--C--W--R--F--G--G--K----1               gi|270063413|gb|GU207406.1|     2       -E--G--Y--W--N--I--L--I--P--K--S--N--K--R--S--F--R--E--Q--S--*--I--F--I--N--Y--K--M--R--L--T--C--N--I--F--S--L--L--H--I--N--V--I--I--K--K--L--K--C--N--Q--K--T--E--I--K--R--M--E--M--A--*--I--E--I--F--L--Y--F--I--G--A--Q--L--F--Q--T--K--N--*--L--M--S--I--I--V--M--N--*--N--T--T--C--T--*--C--I--D--V--C--K--T--R--T--L--F--K--L--R--P--R--E--I--F--N--Y--S--I--L--Y--Y--F--S--T--*--V--E--G--L--*--P--I--N--F--*--I--H--T--N--L--Q--I--K--S--I--Y--I--N--R--S--T--L--L--R--H--I--L--D--N--I--H--S--S--*--L--A--R--C--P--G--R--Y--C--V--S--V--N--R--V--V--I--F--V--F--G--I--N--M--D--D--D--I--I--Y--V--S--S--F--I--A--C--W--K--Q--E--E--*--N--K--S--*--K--*--T--S--*--*--G--P--V--H--P--A--S--S--Y--N--*--T--T--N--M--N--C--I--F--D--I--I--Y--H--K--T--I--Y--F--Y--R--F--G--H--D--K--L--*--N--C--T--Y--*--Q--I--*--F--L--I--S--L--E--*--V--F--I--L--S--Q--L--L--C--L--V--K--S--S--*--S--I--C--T--L--A--H--R--K--H--I--I--Y--K--*--Y--F--*--R--I--*--H--V--S--Q--V--*--Y--V--T--A--K--V--M--T--S--S--F--S--E--L--H--Q--G--G--R--P--I--E--K--R--Q--C--N--I--F--S--Y--I--R--Q--*--S--G--L--L--P--M--R--A--L--P--M--S--V--F--I--W--P--V--D--L--Q--*--Y--I--N--R--*--G--L--I--L--E--*--N--F--K--N--D--A--A--S--M--R--T--T--T--W--Y--G--W--Y--W--M--Y--H--E--G--Q--I--Y--I--I--S--E--V--V--I--A--C--L--P--L--M--L--D--*--Y--W--*--C--D--*--M--V--*--F--Y--*--D--*--R--W--M--I--A--L--I--*--H--G--F--Y--V--G--R--K--S--Q--I--K--R--M--Q--R--F--K--K--K--C--T--V--K--S--K--*--*--Q--A--Y--V--*--C--E--S--I--K--P--L--*--F--F--N--F--V--A--G--I--S--R--Y--V--R--I--Y--L--Y--I--S--F--F--Y--F--R--L--D--V--V--D--G--V--T--Y--C--F--K--K--K--H--S--L--K--G--K--Q--L--L--P--V--I--R--K--L--R--D--I--Q--D--P--H--G--I--K--Q--L--A--R--T--S--S--A--R--R--T--E--N--M--S--D--E--D--V--L--K--A--V--E--D--L--T--G--E--N--G--I--E--L--L--E--L--V--G--I--I--Y--N--K--T--K--F--C--H--E--*--R--I--D--I--Y--D--I--T--V--*--*--L--L--N--L--*--L--I--T--I--E--V--*--E--Q--V--M--P--F--Y--*--I--K--F--I--*--N--N--R--E--G--P--K--*--*--G--F--C--K--V--T--T--P--L--L--H--I--T--F--*--*--K--R--T--T--R--R--T--G--F--A--*--S--R--I--R--S--F--V--S--N--D--C--R--S--R--E--R--A--Y--F--K--F--K--E--G--N--R--Q--R--S--G--T--L--S--*--R--I--R--F--F--I--K--R--V--I--N--Y--L--*--Y--L--I--I--S--N--*--L--C--I--V--N--K--L--I--S--N--L--L--V--C--I--D--S--D--*--S--F--*--I--N--G--*--T--I--F--T--S--T--R--Y--S--I--T--T--C--D--D--S--N--H--Y--Q--M--I--Y--A--F--*--D--S--N--G--G--K--R--S--K--L--Q--D--S--T--T--E--V--K--D--K--D--G--T--H--I--Q--Q--V--K--D--K--I--*--V--L--C--T--Q--S--Q--P--S--E--A--L--I--Y--*--V--*--M--N--Y--L--G--*--L--E--K--*--T--M--R--T--W--M--C--M--K--N--C--I--E--N--V--H--V--V--G--C--R--A--R--L--T--L--E--I--F--C--I--T--V--L--G--G--K--G--A--G--T--V--T--K--A--I--N--K--C--L--K--E--S--G--I--S--E--V--Y--H--G--L--H--S--F--A--F--W--M--E--G--L--T--I--Y--L--S--N--C--W--N--D--N--Y--I--F--S--*--K--I--E--C--N--N--N--S--V--Y--*--*--P--F--G--D--S--*--P--I--R--R--H--Y--*--L--G--S--K--R--T--L--F--L--G--D--I--C--S--T--S--N--F--N--P--F--R--L--H--S--I--T--*--K--I--K--Y--W--R--F--L--D--S--W--K--G--Y--A--K--W--R--S--E--S--H--K--Q--E--N--T--S--I--S--T--D--A--S--G--Y--K--Y--G--A--V--V--C--F--Q--G--E--K--V--E--L--G--D--F--L--E--T--N--D--S--R--P--I--H--L--K--E--A--E--A--V--L--Q--V--L--K--S--I--E--E--V--I--R--D--S--R--V--D--L--L--V--D--N--V--A--V--L--S--V--W--N--T--Q--G--G--R--D--R--S--L--N--N--I--T--K--Q--I--F--Q--L--V--T--S--C--N--C--D--L--H--M--Q--Y--V--P--S--E--*--M--G--Q--I--Y--L--F--E--C--M--F--E--Y--W--F--M--A--A--C--*--K--A--I--W--T--S--F--N--G--P--H--G--Y--*--F--*--C--D--E--I--F--R--R--Y--S--T--T--K--F--N--P--Y--P--T--P--Y--S--S--C--V--N--L--F--A--Q--D--L--P--V--V--V--N--A--Y--V--Y--P--P--F--A--L--I--H--P--V--L--L--F--L--R--E--Q--K--V--G--M--C--T--F--V--L--P--L--I--K--P--V--P--V--W--W--P--L--V--Q--K--H--V--I--Q--S--L--E--L--G--T--R--G--D--K--A--V--I--R--V--P--*--K--R--F--H--H--*--*--*--G--S--E--M--A--T--S--C--I--Q--A--I--V--Y--L--S--*--L--F--F--I--L--*--G--E--S--L--D--L--V--K--I--D--K--R--I--K--E--L--N--D--K--S--S--S--S--S--L--S--K--R--R--D--T--L--K--N--S--L--N--L--F--L--P--L--L--R--K--N--C--T--I--S--N--C--T--P--D--D--L--K--H--F--W--Y--G--R--M--I--L--V--K--H--Q--F--I--K--*--I--V--H--F--W--V--L--K--L--*--M--P--K--R--I--S--S--W--D--S--I--G--H--G--I--K--F--V--G--D--L--L--R--K--W--S--W--*--I--L--G--*--E--K--*--Y--R--*--P--C--S--G--T--L--Y--Q--T--V--C--E--T--Y--S--R--G--T--S--G--S--T--C--S--F--*--A--G--K--S--N--F--S--G--*--N--K--Q--N--C--L--V--Y--R--*--*--I--G--*--G--*--Y--F--I--E--G--K--I--C--A--P--*--R--S--S--L--E--E--I--T--V--L--W--G--K--S--G--K--*--L--G--N--A--C--R--T--G--G--K--E--I--R--*--I--L--V--*--L--K--N--K--H--L--G--K--L--*--E--E--I--N--I--N--L--M--F--L--L--L--R--N--A--E--T--R--L--F--V--Q--*--L--V--C--T--H--M--F--K--I--V--K--E--W--A--C--I--L--Q--M--V--F--F--F--E--L--F--*--R--M--V--E--S--*--V--I--E--*--H--I--Q--*--C--M--K--D--*--F--G--I--L--L--C--*--E--Y--M--R--V--R--L--L--I--V--L--E--S--G--V--Q--*--L--W--L--Y--L--V--R--L--P--M--*--D--K--L--*--T--M--W--D--G--R--E--R--E--Q--Q--N--I--I--V--D--C--Q--L--*--W--N--L--I--I--L--Q--V--D--*--Q--T--V--Q--V--N--V--I--L--W--K--I--S--S--N--S--M--G--I--L--I--F--Y--K--K--L--F--I--N--D--K--A--F--K--M--S--I--Q--L--R--E--*--D--R--S--F--V--F--E--F--A--V--D--*--L--S--Q--V--*--Y--V--T--A--K--V--M--T--S--S--F--S--K--M--Y--Q--*--Y--*--I--L--R--M--S--T--V--D--G--I--D--G--S--E--Y--P--*--V--Y--P--I--H--P--T--L--V--Y--V--L--V--L--G--S--*--*--*--L--H--F--P--I--H--E--K--N--*--S--T--N--V--Y--T--W--D--R--Y--R--C--N--V--H--L--Y--F--I--N--L--N--F--A--I--S--Y--S--L--Q--T--Y--M--L--Y--I--*--C--S--N--A--T--F--Y--M--*--*--*--N--I--P--M--S--G--I--R--R--T--S--R--K--R--I--A--K--I--S--M--K--H--V--K--S--P--P--T--T--A--S--D--T--G--N--A--N--S--D--P--I--S--*--T--N--G--K--E--P--L--I--N--I--P--P--M--I--A--D--M--*--I--S--T--A--R--N--H--I--L--L--N--F--Y--H--L--L--H--Q--M--*--Q--S--F--Y--I--F--I--K--Y--S--T--K--I--R--*--G--K--F--S--T--T--A--S--V--H--*--F--*--I--M--V--S--S--K--*--Q--V--V--I--I--S--L--G--N--G--N--S--I--F--P--K--T--Y--*--L--I--K--R--M--I--T--L--T--L--K--F--I--N--L--K--Q--*--Y--L--*--R--*--V--*--F--Q--S--S--*--E--K--T--T--H--S--L--G--L--L--R--H--S--S--R--L--K--R--I--*--C--V--S--K--I--Y--L--K--I--C--N--K--I--*--Y--R--*--L--S--Q--L--F--M--N--C--H--S--T--R--*--T--*--L--Q--K--M--F--V--S--K--I--P--P--N--K--K--S--R--H--A--S--S--C--C--H--V--E--S--*--P--S--D--F--T--L--K--I--L--W--R--K--H--S--I--S--H--I--L--V--*--V--D--R--L--I--F--F--A--*--S--S--R--S--I--N--C--L--*--T--V--F--M--L--*--D--F--S--M--H--V--I--C--*--T--L--T--T--I--V--I--L--L--Y--Y--R--E--N--E--S--L--*--T--F--E--I--N--L--K--N--K--T--Q--K--N--N--P--L--T--F--I--I--L--V--F--A--S--*--K--A--I--W--R--H--L--Y--L--L--D--S--Y--R--L--G--L--I--T--H--L--I--Q--P--A--S--F--Q--I--L--H--*--V--L--H--W--P--N--P--T--I--*--E--I--C--S--F--K--Y--F--E--N--I--L--N--Y--*--I--T--R--T--K--M--C--F--R--E--C--C--I--L--N--C--I--S--S--I--V--A--V--S--K--*--I--F--S--T--K--P--W--E--Y--R--E--I--H--I--Q--F--K--C--L--V--*--*--Q--*--Y--S--Y--L--A--T--L--P--R--D--S--L--*--L--V--L--C--T--E--A--D--V--L--G--G--V--G--G--L--A--G--R---2               gi|270063413|gb|GU207406.1|     3       --R--D--I--G--I--S--L--Y--L--N--Q--T--K--G--R--S--E--N--K--V--E--F--L--S--T--T--K--*--D--L--P--A--I--Y--F--L--Y--Y--I--S--M--L--*--*--K--N--*--N--A--I--K--R--L--K--*--N--V--W--K--W--L--E--L--K--S--F--C--I--L--L--G--L--N--C--F--K--L--K--T--D--*--C--P--*--S--L--*--T--R--I--L--H--V--H--N--V--S--M--F--V--K--H--A--L--F--S--N--Y--D--H--V--K--Y--S--T--T--A--F--F--I--I--F--Q--L--R--L--K--V--Y--N--Q--*--I--S--K--Y--I--Q--I--C--R--L--N--L--F--I--S--T--D--R--P--Y--*--D--T--F--*--T--I--F--I--H--H--S--L--H--G--V--P--A--V--I--V--F--L--L--T--E--S--S--Y--L--S--S--A--L--T--W--T--T--T--L--F--M--S--V--R--L--*--R--A--G--N--K--K--S--R--T--N--P--R--S--R--L--P--D--K--D--Q--F--I--Q--L--V--P--I--I--K--Q--Q--I--*--I--A--F--L--I--L--S--I--I--K--Q--Y--T--S--I--D--L--V--M--I--N--C--R--I--V--L--I--N--R--F--N--S--*--L--A--*--N--E--S--L--F--C--R--N--Y--Y--V--W--*--S--H--H--K--V--F--A--L--*--H--I--E--S--I--L--Y--T--N--N--I--S--E--G--F--S--M--Y--P--K--C--N--T--S--L--R--K--*--*--R--H--L--S--Q--S--Y--T--K--E--E--D--L--L--R--S--G--N--V--I--Y--F--L--I--L--D--N--R--A--V--C--C--R--*--G--H--C--R--*--V--F--L--Y--G--Q--W--I--Y--S--D--I--S--I--A--E--D--*--F--W--S--E--I--L--K--M--M--R--R--R--*--G--R--P--H--G--M--A--G--T--G--C--T--M--R--A--R--Y--T--*--S--A--R--W--*--*--L--V--Y--R--*--C--*--I--N--I--G--D--V--I--K--W--Y--D--F--T--E--T--D--D--G--*--L--H--*--F--N--M--D--F--M--*--V--E--N--R--R--*--N--V--C--N--V--L--K--K--N--V--P--S--R--V--N--N--N--K--H--T--C--D--V--N--Q--L--N--L--Y--D--F--S--I--L--W--P--E--L--V--D--M--Y--G--S--I--C--I--L--A--F--F--T--F--G--L--M--L--*--M--V--L--P--T--V--L--K--K--N--I--P--*--K--E--N--N--Y--Y--Q--L--S--E--S--*--G--I--F--K--I--R--M--A--L--N--S--*--L--G--L--L--L--L--G--V--P--K--I--C--L--M--R--M--Y--*--R--L--L--R--I--*--L--E--K--T--G--L--S--Y--W--N--W--*--V--L--F--T--I--K--Q--S--S--V--M--N--E--E--L--I--F--M--I--L--Q--C--N--S--S--*--I--S--N--*--*--L--*--K--F--K--N--K--*--C--H--F--I--E--L--N--S--F--K--I--I--G--K--D--P--N--N--E--D--F--A--K--L--L--L--H--F--C--I--L--H--S--N--K--K--G--Q--P--E--G--Q--D--L--H--D--P--E--L--G--H--S--S--A--T--T--A--D--Q--E--K--G--P--T--S--N--S--R--K--A--T--D--K--D--Q--V--L--L--V--N--G--L--G--F--S--S--K--E--*--L--I--I--C--D--I--*--L--L--V--I--N--Y--V--L--L--I--N--*--F--L--T--Y--*--F--A--L--I--V--I--N--H--F--E--*--M--V--K--Q--Y--S--L--Q--P--G--T--V--L--L--L--V--M--I--V--I--I--I--K--*--F--M--H--F--R--I--Q--M--V--E--N--V--V--S--Y--K--I--Q--Q--L--K--*--K--T--R--M--V--H--I--F--S--R--*--R--T--R--F--K--F--S--V--P--S--P--S--P--V--K--H--*--F--I--K--F--R--*--I--I--*--V--S--*--K--S--E--P--*--G--H--G--C--V--*--R--T--V--*--K--M--Y--M--*--*--D--A--E--Q--D--*--H--W--K--F--F--V--*--R--C--L--G--G--K--V--Q--V--Q--*--L--R--P--*--I--N--V--*--R--R--V--G--F--L--K--F--I--M--V--Y--T--V--L--P--F--G--W--K--A--S--P--Y--I--Y--Q--T--V--G--M--T--I--T--Y--F--L--R--K--L--N--V--I--T--T--Q--Y--I--D--D--R--L--V--I--A--N--P--S--E--G--T--T--D--L--E--A--K--E--H--C--F--W--V--I--Y--A--L--L--Q--I--L--I--R--L--G--Y--T--V--S--L--E--K--S--N--T--G--V--S--*--T--R--G--R--V--T--Q--S--G--G--Q--R--V--I--S--K--K--I--P--V--F--Q--Q--M--L--L--A--T--S--M--G--Q--*--F--V--F--R--G--K--R--L--N--L--E--I--F--W--R--Q--T--T--V--D--L--F--I--*--R--K--L--R--Q--S--F--R--S--*--S--Q--*--R--K--*--L--E--I--Q--G--L--T--Y--W--*--I--M--L--L--F--L--V--F--G--T--L--K--E--D--G--I--D--L--*--T--T--*--Q--N--R--Y--F--S--W--*--L--L--V--I--V--I--C--T--C--N--M--Y--P--Q--S--E--W--G--R--F--T--C--L--N--A--C--L--N--I--G--S--W--Q--R--V--E--R--Q--F--G--P--H--S--M--D--L--M--A--I--D--S--N--V--M--K--Y--S--G--G--T--A--L--P--N--L--T--H--I--L--P--H--I--H--L--V--*--I--C--L--L--R--I--C--Q--W--W--L--M--H--M--F--I--L--L--L--P--L--Y--I--L--Y--S--Y--F--*--G--N--R--K--L--V--C--A--P--L--S--Y--H--*--S--S--L--Y--L--F--G--G--L--L--F--K--S--M--*--S--R--V--*--S--*--G--P--G--E--I--R--Q--*--*--G--F--L--R--K--G--F--I--T--D--N--K--G--L--R--W--P--L--V--A--F--R--L--L--F--I--*--A--N--C--F--S--F--C--R--E--K--A--W--I--W--L--R--L--T--R--G--L--R--N--*--M--T--N--L--H--H--H--L--Y--L--R--E--E--I--L--*--K--T--L--*--I--Y--F--F--H--Y--*--G--K--I--A--Q--Y--L--I--V--P--L--M--I--*--S--I--S--G--M--E--G--*--F--W--*--N--T--S--S--*--N--K--L--Y--I--F--G--Y--*--S--C--E--C--P--R--G--L--A--A--G--T--V--S--V--M--V--*--N--L--S--E--I--F--Y--E--S--G--R--G--K--F--W--D--E--R--S--N--T--G--N--P--A--A--A--H--C--I--K--Q--Y--V--K--L--I--Q--E--E--Q--A--A--A--H--V--V--S--K--Q--A--N--P--T--F--L--G--K--I--N--R--I--V--S--Y--I--D--N--E--L--G--R--V--D--I--S--S--K--E--R--Y--V--H--L--K--D--Q--A--W--R--K--L--Q--F--F--G--G--N--R--A--N--D--*--G--M--L--V--G--Q--E--V--K--R--L--D--E--F--W--F--S--*--K--T--N--I--W--E--N--F--K--R--K--*--T--*--I--S--C--F--C--Y--*--E--M--Q--R--Q--D--C--L--S--S--N--W--F--A--H--I--C--S--R--L--*--K--N--G--R--V--S--C--K--W--F--S--F--S--S--C--F--R--E--W--*--S--H--R--*--*--S--D--I--F--S--N--V--*--K--T--D--S--V--S--Y--F--V--R--N--I--*--G--*--D--S--S--*--F--*--S--R--V--C--S--D--S--G--F--I--W--F--G--C--Q--C--R--T--N--Y--E--P--C--G--M--V--G--R--G--N--S--R--I--L--*--*--I--A--S--F--S--G--I--*--L--Y--C--R--*--I--S--R--Q--C--R--S--M--*--F--C--G--R--S--V--P--T--V--W--G--F--*--Y--F--T--K--S--F--S--*--T--I--R--P--S--K--C--Q--Y--N--*--G--N--K--T--D--P--L--F--S--N--L--Q--*--I--N--Y--P--K--S--N--T--S--P--R--K--*--*--R--H--L--S--Q--R--C--T--N--D--T--E--Y--*--G--*--A--R--*--M--A--L--M--V--Q--S--I--H--E--F--T--P--S--T--P--P--L--Y--M--F--L--F--S--V--H--D--N--D--Y--I--S--Q--Y--M--R--R--T--R--A--R--T--Y--T--P--G--I--G--I--G--A--M--S--I--S--I--S--S--I--*--I--L--Q--F--L--T--L--C--R--R--I--C--C--T--F--D--V--Q--M--Q--H--F--T--C--N--D--K--T--Y--L--C--L--G--S--E--E--P--V--G--K--E--L--R--K--S--P--*--N--M--S--R--V--P--Q--L--L--P--P--T--R--G--M--P--T--Q--I--P--S--R--K--P--T--E--K--S--R--*--L--T--F--L--R--*--*--L--I--C--R--F--Q--L--Q--E--I--I--F--C--*--I--F--T--I--F--Y--I--K--C--D--K--V--S--I--F--L--*--N--T--L--L--K--Y--D--K--V--N--F--Q--Q--P--R--L--Y--T--N--S--K--S--W--Y--H--L--N--N--K--W--*--S--Y--H--W--E--T--E--I--Q--S--F--Q--K--R--I--D--*--*--R--E--*--*--H--L--L--L--N--S--S--I--*--N--N--D--T--F--R--D--R--Y--D--S--R--A--H--E--R--K--P--H--I--A--*--D--C--S--A--I--H--Q--D--*--K--E--F--S--V--S--Q--K--Y--I--*--K--S--A--T--K--F--N--T--D--N--Y--H--S--Y--L--*--I--V--I--L--P--D--K--H--D--Y--K--R--C--S--C--P--K--Y--L--P--I--R--S--P--D--T--P--P--A--A--V--M--*--N--P--N--H--P--T--S--L--L--R--Y--Y--G--E--N--T--A--F--R--I--F--*--S--K--W--T--D--*--F--S--L--L--E--V--Q--G--P--*--T--A--Y--K--L--S--L--C--F--K--I--S--V--C--M--*--F--A--K--P--*--L--Q--L--*--Y--Y--C--I--T--E--K--M--N--P--Y--R--H--S--R--S--I--*--K--T--K--P--K--K--I--I--L--*--P--L--L--Y--W--F--L--R--A--R--R--Q--S--G--D--I--C--T--Y--L--I--H--T--D--W--V--*--*--H--I--*--Y--S--L--P--A--S--K--Y--S--I--R--Y--C--I--G--Q--T--Q--Q--Y--E--R--F--V--P--L--N--I--L--R--I--Y--S--I--I--E--*--H--A--Q--R--C--V--S--A--N--V--A--F--*--T--A--L--A--Q--S--L--L--F--L--N--E--S--F--Q--R--N--P--G--N--I--E--K--S--T--Y--S--S--S--A--W--F--D--N--N--D--T--H--T--*--P--H--F--Q--E--I--P--C--S--W--F--S--A--Q--K--Q--T--Y--L--V--V--L--A--V--W--R--E--D--3                   ---                  - Fasta sequences of sequences that were incomplete in genbank, cDNA sequences, and sequences from unfinished genomes assigned a temporary id can be accessed here.                                ```               >BRAFLDRAFT_68991_Bflo_260795011               MLSNAKSGKATSLDSPPFYTSRTGYKMCARIYLNGDGMGKGTHVSLFFVVMRGHYDGLLRWSFRQKGSVVPVWTFGTLYYPEMLCRGKGLGIEHTMTCVLTRVSTPDQNGVMSSPPVLRADATLVNRSILPLLRELRKPDGPPEEDQLARFEAETPLSAYNAGEIAARIGALTGEPPEDLMEMAGMDPKAEASMRKLAHCAILAANNITAAQPIKQDKASRDAAQAAVGLVEDDPSTSEDGPSTSGLGRKDGKRPATAPPKAAPGRQGKKSKRDPASSSESDSDDDDMEDDEVECQLRRVKRVCQDPFIHLYPARLQNAVRRLVRAAKEAGHKRLSRFKPLPAVPRLWKPAVACPECHHPNDQDFRHCQMCNYVRRPCPPPRKCVEIDKAAIDNRLDEINRMAKSTDYGKKKTALENELVDFLGNSAPPKDLVTASPKDVCAFLVWKDKGGKTVVHKVNCKYFGEKRKTGCGCPKRLAAGTVDSIIGQLRAIFTISGRGRDWTEAVCAGNPAAAPVVRQYLKVTKVEQANAMIQPKQAQPVFFDKLTAVCTHITKKMKEKEVRHTTLFALARDQLQAFLKIMFFAADRASDLARCKSEELAWLPNEEGILFNHTFGKTLRDGTVNAFPILAGKNKSMCPIQGLRLYIRVAQSLKICLDKGYLFRAINKAQEVTNDPFTYDAAQYRFKSVILPLFGPLALASIYCPYLNPNRGKVTWD               >NEMVEDRAFT_v1g218021_Nvec_156358300               MPNKRLLRRELEEFLEALPGYVSIATVTPRDICRFLVSKDKDGRTQVHRLSCRFIVKKGHFGCGCPTHLSYKTVDSYIGKLRAIFHAMGRDsewdkrlGLGNPAVDKSLKDHLRLITAGQLQARMDKLSQLALHLDSEMNKAKRNIDRFIIVRDQAYHKMAFFSEDRPSDLGQIKVAEMLRFPQNDGFLFNHIWGKTlrgGDGNVFGVRRnpqleiCQIRGIEQYMEVARDigidlRGYLFRPVTpdlgvdsplsssAAEARLKGYLKDLKADEGETLHGFRAGCAITLALTGAELSEIMDHIGWSNRHTALFYMQLE               >NEMVEDRAFT_v1g220156_Nvec_156352960               PYQKRTSSLLKELERFLDSLTPPKNLMSASPRDINRFLVWKDEGGRTKIHKPTCTKYGSAGSARCRCPSRLAAGTVDSIIGKLRAIFAEAGRkgewnemlNIGNPSSHRSAKGYLTSIREEQAMAHVSPKQATPIFFDKLAKLCRFLRnlvfvekatSIQRHIHARDLAFFCLDFFAGDRASGLGRVLTKEALASKDGETIWFRHTYGKTlrggGDTNVFPIKKyaldpvaCPVANLRLYIKLCDImkinlrEGYIFRATDGatkvsdnpfvgsTIANRLKLHLGKADILEGETMHGFRSGCSITLSLLRVSTEDVARHVGWKSTSTADYYSQTG               >ALOG_Sprat_299509744               MGEGTSGEGMHHHHETGKSSSSRYESQKRRDWNTFGQYLKNHRPPIAMNKCTSQHVVEFLRYLDQFGKTKVHNEGCQFYGTSGGNPCGCPMKQAWGSLDSLVWRLRAAYEENGGKSENNPFGTRIVRTFLRDVREHQAKARGIAYEKKKRKRAGAGA               >contig_11092_Esil_242186836               MRHKSHTCSQQHTLVMAKQAQFSDIRDFLLHISCVSQVGSGAALFLAERAARTAHIRTQLVGGDLISASAKQSTVDLSNIRRLQQSHCLPDQARMWVTDDFVPAHLQTATEFWRDEILQDTPVGDRNTLLGWVKGVNVYDFVDTKATGIFHGASYNGADLTSIHLPNHVPDEHVSWVTSEVAKLAATGCVTRWKDVADVSVYAKPQMVLPLGVEPTKPRLIWDARWLNLMCRHHPFSMDGVGKVAQCAWPGAHQVTIDHKAGYHHVALDKGSWQYFGFEWEGELYVFTVLAFGWCSAPFIYASLSEAVARYLRARDIPVLTWIDDFYLTNFRSTRTLSYDEQLKAAQTTAYVALEVLYSAGYFISLKKCELIPTTSLVFLGIICDSDNRRFEVPEDKLAKLEAILVDAIASESITFQMLEKLAGKCTSLSVAVPVAALYTHHMYKSIAVFQRRGGRKPSMTIPVPKNSGLMFELKRWLEVREHFNGASWYRAEHKQLALTGASDASSGGWGGLIRSPGQPIFKAGGDFPLHVAQQHINVQEGYALQQTLRLFSDSQPSQLAGSTLISKVDSKVLHDAFKKGRSSNTLMHEIITDLFWLQVRRDFTLKLKWVSSEENAEADGISRPGSDDFVRLDERMFGDLCAWAGEQVTMDLMATPASVHKRWVDGRCTSEDLPFYSRYHTQGCAGVDVLTQNVRFMPGSTTEECFGFCFPPTSMVGVFLQHLEECRAKALVIVPDRKQYWFPRIADAATRSRTLSIGSGGESPFFRVHHQKGSERFHFKRSGMLAVVWISPGMLSDEEFVRLVLNVSPVHVRWFVGQRILYQALQAVFTALGLIVSILEAIIPKRDRLGEAKLPPPPLVKVSASRARVLCPECHSENDDSFRFCQWCGYSIAQHQPRTTPPLQVDEEAISRRYQQFLNAWAEKASARSRSATWALFSNFLASRRNGSVSIENAQPKDVVEFLCWLDSCGSRRRTIVHAKHCEAVGTKDLTACSTDKGECSLRYAFDSLRTNHVSKLSMVFEKEMGVVTPWSKTMRVGNPVKSELVAQYMAFTTSEQKQAGVLVKQAPVILRSHLEKIIFPMQIRLQYASSDVERVTLARDIAFFSVAFSTTKRGVELTNILIQRILRLPNRSGLMCNFQWGKTQRDGADHILTVPYDEEYVAICPVRAVERFIAVGKQVGWDTTSGYLFPDISESMQGEAQRGKLPVATSRMSEALKRYAAAVGETQGFSLHSFRSGGAVSRALAGDSLSTIMQKAYWKSPKTAWRYMRLMEVVAPGSEGTAMVEGVSEEQYRQLNEFGLSEQSRSLSAFSNKPLL               >gi|Adig1000023598|ref|adi_v1.18287               MAEREEPALPLPPAAAPAAPNPAVIAPVVKPLDIPAPAAEPEPDERLRQLEEKVRLLEQEKAVASCDNALTALRRHLNRPPSLFDRHEAIELLESLVRLARTQARDKVEEYSAALDEVKARQASLETGHLQRLILGLVTRPWKPARRCSACLYPNDSDANYCQACGTLTGPRCSAAPVPPLDETAIQERFDEFQSVFRSKPYERRKSALEQQLFKFLGALSPPRTMTSCTAQDIVKFLISKDRSGRTVVHSLSCSKRGCSCPKRLAAGSVDSTLGRLRAIFNKLGRANDSNPASHPLVKDYLKFVREEQAGLAITPSQAVPIFFGKFQQLIAHLRDLCSSSVFLSSASKYILVRDATFFVVDFLIGDRASDLGRLQSCNVFRLRDREGFLLRFTLTKNLRKGPPRSVALIKFAHSHVCPVAWIQYYITVCQCLKVPLDQGYFFRTAERSGSIGSNPFTGSAVNNRLRKHLSEAKLYAGETPHSFRVEGCQGLTTCGKRPDSVNDLRDPSYAELGTFSFQWLGDGQLTITKVSKKTVNSERSSKPQFGCGKSIEWKKMATVAVPSEFIETVKVGTSDIKTLPPTAQNFQQELSGKLDEAGKNLQLDELQPSG               >gi|Lgig1000000655|ref|jgi|Lotgi1|67844|gw1.188.17.1               TKYVHQKEKLTSQLTLFLMSVYSTLNLDNASPGVMREFLVWKDSTGKTKVHLDSCVFRTQSDKASCKCPIRRAASSLDTLIGQLRAIFRDHGRGSDWNEVLGFGNPMAAPSIKRHLQAVTLEQSK               >gi|Lgig1000014628|ref|jgi|Lotgi1|171385|fgenesh2_pg.C_sca_114000014               MQHSHSEEIWEESNSTLNLDNASPGVMREFLVWKDSTGKTKVHLDSCVFRTQSDKASCKCPIRRAASSLDTLIGQLRAIFRDHGRGSDWNEVFGFGNPMAAPSIKRHLQAVTLEQSKALVQPCQAMPLFFDKIVRMCRVINYELAHKDRLSGKKRYALARDKPYFTLMCFTGDRAGDVGRLKRDQIR               >gi|Lgig1000016045|ref|jgi|Lotgi1|175377|fgenesh2_pg.C_sca_274000004               MSRKAWNEVKKPDMLECLKTFRANGQPPKVMVNWKEAVNIETLKIEELKSGFEKMDVDETEMAQLLGIAETRENLIVMAIATILTALKISGSHNPGAGIDKVSIKERIVELDSLIGSKKYVKQKDRLQTDVEKFLEEYSKSTIDKATPHDMRAFLVYKESCGKTKVHRKDCLVVSGTGCHCECLLDMSANSVDSLIGKLRAIFRDRGRGSKWDADLGTGNPLASLSIKNHLKALKMEQLQADVIPIHAVPLFLDKAAKLDRYLEFYLTRPLLIREEYLVRRDKAFIKFICHSGDRAGDLANLRTDQIKQTEKGLLVRMTQGKTIKQKLEYFV               >DB416162_Apect_93299263               RKPPCSCPRRLAFGTVVTKTAQLKAIFQSMGKQHDWPGDSGNPVCSDIVNAYVKQIKTEQSIGHTSQVQAKPMFTSKLSLVAQYIDRHLSDSADPKSRFILARDQALWKLMFFGGDRAHDLGLMLTQEIRILPNQAGYAIRHTWGKTHRLNRPNVFSIYKNPDELLCPVIGLDAYLLVAHELGINLATGYLFRPVIQNVVRDDPLDYQAIYARFKYYLNELGIDE               >_Cgig_260745145               DLKCVQEEQAKAHILPKQAKPFFLTKLETISKFISRELGRSDLTLKERFVLIRDQALFKLQFFAGDRASDIAITLLQEVKYLRKKDGFAFNHTFGKTLRGNGKTNTFVIKCCPNETICPVRGLERYFAEAKRLGIDMSSGYLFRMVTESGRVINEPMSYSSIYERLKCYLITLGIDEGETPHSLRAGCAVHMCFSNAAHDVHDLMNHVGWATESSAKYYSRYDTLLDASNTAVRLAQSSERAKGIEIMFHDKANFGELPSTTTE*CLVHCIDVQCFVTCFKRMHCVMVTKLKINQL               ```                Back to Contents                 ---                     ---               ``` |
